# Detecting Pediatric Emergency Service Use for Suicide and Self-Harm: Multimodal Analysis of 3828 Encounters

**DOI:** 10.2196/82371

**Published:** 2026-02-04

**Authors:** Juliet Beni Edgcomb, Angshuman Saha, Alexandra Klomhaus, Elyse Tascione, Chrislie G Ponce, Joshua J Lee, Theona Tacorda, Bonnie T Zima

**Affiliations:** 1 Mental Health Informatics and Data Science (MINDS) Hub, Semel Institute for Neuroscience and Human Behavior, University of California, Los Angeles, 760 Westwood Plz, Los Angeles, CA, 90095, United States, 1 310-825-9989; 2 Department of Medicine Statistics Core, David Geffen School of Medicine, University of California, Los Angeles, Los Angeles, CA, United States; 3 Biomedical Informatics Program, Clinical and Translational Science Institute, University of California, Los Angeles, Los Angeles, CA, United States

**Keywords:** children, classification algorithms, electronic medical records, emergency services, mental disorders, natural language processing., text classification

## Abstract

**Background:**

Suicide is the second-leading cause of US childhood mortality after 9 years of age. The accurate measurement of pediatric emergency service use for self-injurious thoughts and behaviors (SITB) remains challenging, as diagnostic codes undercount children. This measurement gap impedes public health and prevention efforts. Current research has not established which combination of electronic health record data elements achieves both high detection accuracy and consistent performance across youth populations.

**Objective:**

This study aims to (1) compare the detection accuracy of electronic health record−based methods for identifying SITB-related pediatric emergency department (ED) visits: basic structured data (*International Classification of Diseases Version 10, Clinical Modification* codes, chief concern), comprehensive structured data, clinical note text with natural language processing, and hybrid approaches combining structured data with notes; and (2) for each method, measure variability in detection by youth demographics and underlying mental health diagnosis.

**Methods:**

Multiple human experts reviewed clinical records of 3828 pediatric mental health emergency visits (28,861 clinical notes) to a large health system with 2 EDs (June 2022-October 2024). The reviewers used the Columbia Classification Algorithm for Suicide Assessment to label the presence of SITB at the visit. Random forest classifiers were developed using 3 data modalities: (1) structured data (low-dimensional [International Classification of Diseases codes and chief concerns], medium-dimensional [adding Columbia Suicide Severity Rating Scale screening or mental health diagnoses], and high-dimensional [all structured data or augmented case surveillance, aCS]); (2) text data (general-purpose natural language processing, medical text-specific trained natural language processing, and Large Language Model Meta AI−derived scores), and (3) hybrid data (combining aCS with each text approach). Model performance was evaluated using area under the receiver operating characteristic curve (AUROC).

**Results:**

Of the 3828 visits, 1760 (n=1760, 46.0%) were SITB-related. Detection performance improved with dimensionality: low-dimensional (AUROC=0.865), medium-dimensional (AUROC=0.934‐0.935), and high-dimensional (AUROC=0.965). Low-dimensional structured (International Classification of Diseases codes and chief concerns) showed high variability in detection, with lower accuracy among preadolescents (AUROC=0.821 vs 0.880 for adolescents); male participants (AUROC=0.817 vs 0.902 for females); and patients with neurodevelopmental (AUROC=0.568‐0.809), psychotic (AUROC=0.718), and disruptive disorders (AUROC=0.703). Hybrid modality (aCS+Large Language Model Meta AI) achieved optimal performance (AUROC=0.977), with AUROC ≥0.90 for all 20 demographic and 12/15 diagnostic subgroups.

**Conclusions:**

This cross-sectional retrospective study identified that, relative to diagnostic codes and chief concern alone, hybrid structured-text detection methods improved accuracy and mitigated unwanted detection variability. The findings offer a scaffold for future clinical deployment of improved information retrieval of pediatric suicide and self-harm−related emergencies.

## Introduction

Suicide is the second-leading cause of death among US children over 9 years old [1]. The estimated annual cost of suicide- and self-harm−related emergency department (ED) use is $510 billion, and among young people, nearly 75% of costs are attributable to nonfatal self-harm injuries [2]. Self-injurious thoughts and behaviors (SITB)—encompassing suicidal ideation, suicide attempts, and nonsuicidal self-injury—rank among the strongest predictors of future suicidal behavior [3-5]. The accurate detection of ED visits for SITB underpins interventions to improve quality and reduce preventable ED use [6-8]. Detection enables public health surveillance for geographically or temporally clustered events [7,9-11], informs health system staffing [12], mitigates crowding [13], and supports policy measures such as firearm safety regulations [14,15] and crisis hotlines [16]. Yet, among children, detection remains inconsistent [10,17-19] and leaves many instances of SITB care unidentified [20], particularly among younger children [21].

Several challenges impede the detection of pediatric service use for SITB. When, where, and whether clinicians document suicidality in structured data or clinical text may reflect medical record software functionality [22], stigma [23], racial bias [24], and provider training in pediatrics or mental health (MH) [25,26]. Diagnostic codes and chief concern may inconsistently reflect suicidality in school-age children [21] and children with neurodiverse [27] who often present to emergency services with less lethal means, without immediate disclosure of suicidality, or with externalizing symptoms [28]. The assignment of a diagnostic code often occurs under associated psychiatric diagnoses [29], such as major depression or behavioral disturbance in autism. Diagnostic inaccuracy may further obfuscate these patterns: fewer than 16% of children who attempt suicide are evaluated by a MH specialist in the ED [30].

In this context, methods lag to detect SITB-related service use among children. Most work focuses on adults [31,32] and leverages costly locally trained natural language processing (NLP) of clinical text to detect SITB events in a research context [17]. These NLP methodologies include deep learning [33,34], pretrained models (eg, Word2Vec) [31], and Bidirectional Encoder Representations from Transformers–based transformer models [35] and the examination of keyword representation in clinical notes of individuals with and without self-harm events [31]. While large language models demonstrate promising capabilities to accelerate the efficiency of clinical text analysis, fewer than 5% of medical NLP applications evaluate large language models against nonsynthetic clinical notes using large human−labeled datasets to assess sensitivity, hallucinations, and algorithmic bias [36]. Structured data—such as standardized pediatric MH codes [37] and triage screening [38]—offer more readily implementable detection strategies for operational use [20]. Although the NLP of clinical notes yields fair performance in adolescents [27,39-41], current literature lacks systematic head-to-head comparisons of SITB detection accuracy across electronic health record (EHR) data modalities (text alone, structured alone, hybrid combined). Further, despite calls for algorithmic fairness assessment in suicide prevention [42], phenotyping strategies have seldom evaluated unwanted detection accuracy variation across pediatric demographic and diagnostic subgroups [17]. Combined with typically small human−labeled validation samples (≤1000 youth) [17,32], performance variation in detection strategies across demographic subgroups remains largely unknown.

To address these gaps, this study presents the first large-scale comparative evaluation of automated detection approaches for SITB-related emergency service use among children and adolescents. The primary objectives were to (1) compare detection accuracy across 3 EHR data modalities—structured data alone, clinical text alone, and hybrid combinations—for identifying SITB-related pediatric ED visits; and (2) for each data modality, measure variability in detection performance by youth demographics and underlying MH diagnosis. The findings provide strategies for SITB detection in pediatric emergency settings, with particular emphasis on measuring accuracy for population subgroups historically characterized by suboptimal suicide prevention care.

## Methods

### Study Design and Population

This retrospective cross-sectional study utilized EHR data from 4 hospitals within a large academic health care system in Southern California serving 5.1 million members, including approximately 400,000 youth. We included all youth aged 6‐17 years with at least 1 MH-related ED visit between October 2017 and October 2019; this period was selected to capture data following the initial implementation of Columbia Suicide Severity Rating Scale (c-SSRS) screening and *International Classification of Diseases Version 10, Clinical Modification* (*ICD-10-CM*) while excluding pandemic disruptions. MH-ED visits were defined as those associated with (1) a pediatric MH disorder as specified per the Child and Adolescent Mental Health Disorders Classification System (CAMHD-CS), a comprehensive taxonomy organizing pediatric MH-related *ICD-10-CM* codes into diagnostic categories based on *DSM-5* criteria [37]; (2) an MH-related chief concern; (3) involuntary psychiatric detainment; or (4) a positive response to ED nursing triage screening for psychiatric complaints. The flowchart for study inclusion is presented in Multimedia Appendix 1.

To ensure the dataset included unique individuals, we analyzed each child’s most recent visit. The multiexpert annotation of all eligible encounters (N=3828 visits) occurred in June 2022-October 2024, with analyses conducted in November 2024-February 2025. We compared 3 data modalities to identify optimal approaches for SITB detection: (1) structured data from discrete EHR fields, (2) text data from clinical narratives, and (3) hybrid combinations integrating all available structured data with NLP of clinical notes. Performance was evaluated against expert classifications using area under the receiver operating characteristic (AUROC) curve metrics for overall cohort and subgroup analyses.

### Ethical Considerations

Data, including clinical note text, were deidentified. Analyses were conducted in secure computing environments. The study followed Strengthening the Reporting of Observational Studies in Epidemiology (STROBE) [43] statement guidelines, as well as the Transparent Reporting of a Multivariable Prediction Model for Individual Prognosis or Diagnosis (TRIPOD) guidelines, TRIPOD-AI [44], and TRIPOD-LLM [45] (Checklist 1). The University of California Los Angeles Institutional Review Board approved this study with the informed consent waiver due to the retrospective study nature and minimal risk (IRB #20-001512).

### Demographic and Clinical Variables

Participants were classified as children (6‐12 y) or adolescents (13‐17 y), with race, ethnicity, and legal sex from patient- or family-reported EHR. Racial and ethnic categories aligned with federal standards [46]: American Indian or Alaska Native, Asian, Black or African American, Hispanic or Latino, Native Hawaiian or Pacific Islander, White, plus other or missing or unknown. We incorporated 2 area-based socioeconomic measures linked by census tract: the social vulnerability index [47], a Centers for Disease Control and Prevention measure ranking communities’ resilience to external stresses on human health (ranging 0‐1, higher indicating greater vulnerability) derived from the 2018 5-year American Community Survey, and the area deprivation index [48], a composite measure of neighborhood socioeconomic disadvantage based on income, education, employment, and housing quality (national percentile 1‐100, higher indicating greater deprivation) derived from the 2019 Block Group ADI files v. 3.0. Further details are provided in Multimedia Appendix 2.

We extracted all available EHR data from index ED visits, restricting to the time window between arrival and discharge, transfer, or inpatient hospitalization. We included all verbatim clinical notes (excluding surgical procedures and medical student notes) across disciplines, including notes authored by ED physicians, psychiatrists, psychologists, other medical consultants, social workers, and nurses. We categorized suicide- and self-harm−related diagnoses by the Centers for Disease Control and Prevention surveillance definition of nonfatal suicide attempt and intentional self-harm using *ICD-10-CM* [29]. Youth MH diagnoses were classified via CAMHD-CS, with psychiatric comorbidity defined as ≥2 categories. Chief concerns were categorized as MH or non-MH and SITB or non-SITB related (Multimedia Appendix 3). We included c-SSRS screening scores, ED-administered psychotropic medications, homicidal ideation screening, overdose-related laboratory tests, urine drug screen results, and discharge disposition. Missing data occurred in ≤6% cases for most variables, except for insurance status (~30% missing) and c-SSRS scores (Multimedia Appendix 4). c-SSRS is asked with gatekeeping question structure where subsequent items are only administered if initial screening questions indicate risk. Thus, missingness was recoded as a separate binary variable for each c-SSRS item to preserve this clinical decision pattern. To estimate prior care use, we included number of ED visits and psychiatric and general medical hospitalizations in the past 30, 90, and 365 days.

### Ground-Truth Labeling

A total of 2 trained staff research associate annotators reviewed structured data and verbatim notes from each visit. Annotators labeled visits for SITB presence or type using a modified Columbia Classification Algorithm for Suicide Assessment [49] (Multimedia Appendix 5). Interannotator agreement was assessed via Cohen kappa. When annotators disagreed, 2 board-certified child psychiatrists reviewed records independently. Consensus discussion resolved clinician disagreements. All encounters (N=3828) received binary SITB−related and categorical SITB-type classifications. For a random 724-encounter subsample, annotators assigned phrase-level labels indicating SITB-related (any), the SITB type (ideation, attempt, preparatory act, or nonsuicidal self-injury), and if the phrase referred to the patient (vs other), present (vs past), and was affirmed (vs negated).

### Text Processing Methods

We developed 3 distinct approaches to assign clinical text scores for SITB detection. We provide complete technical specifications, Community Advisory Board consultation details, and prompt engineering protocols in MultimediaAppendices 6  7.

The first approach (general-purpose natural language processing [NLP-general]) adapted a semisupervised methodology from common semisupervised approach (PheCAP) [50] through the following sequential steps. All sentences from the 724 held-out encounters were segmented using spaCy, then embedded using the Universal Sentence Encoder CMLM-en-base and indexed using the Annoy approximate nearest neighbor algorithm [51] with angular distance metrics. Then, for each sentence from the remaining 3104 encounters, the K=5 nearest neighbor sentences were retrieved from the labeled training set, and a sentence-level score was computed as the mean from these neighbors. We determined encounter-level scores by averaging the sentence-level scores (k-normalized votes per sentence) across all sentences within the encounter [51].

The second approach (medical text-specific trained natural language processing) employed identical methodology but substituted MedEmbed-small-v0.146 for sentence vectorization to leverage domain-specific medical embeddings.

The third approach (Large Language Model Meta AI [LLaMA]) utilized large language model processing through a multistage implementation. We leveraged the 724 held-out encounters to iteratively develop and improve upon a condition-specific prompt. The prompt includes instructions to output a Likert-type score ranging from −3 (definitely does not contain SITB) to +3 (definitely contains SITB) along with explanatory text as JSON objects. We tested iterations of this prompt using LLaMA-3.2-1B (selected for computational efficiency with 10× faster processing speed) by comparing the Likert scores against note-level labels from human reviewers. Once preliminary accuracy was established, we presented the prompt to the study Community Advisory Board that suggested additional revisions. Once the prompt was finalized, we conducted final scoring on the remaining 3104 encounters’ notes using LLaMA-3.3-70B. We then determined encounter-level scores by selecting the maximum score across all clinical notes within each encounter.

### Classification Models

#### Feature Set Definitions

We define 3 data modalities based on the fundamental data type: (1) structured modality used discrete EHR fields, (2) text modality used clinical narratives processed through NLP, and (3) hybrid modality combined both data types. We compared a total of 10 feature sets against multiexpert chart annotation—4 structured modality, 3 text modality, and 3 hybrid modality.

We categorized structured feature sets by dimensionality based on the number of input features: low (<10 features), medium (10‐50 features), and high (>50 features). The 4 structured data feature sets were as follows: *Low*: (1) SITB-related *ICD-10-CM* codes and chief concerns (International Classification of Diseases codes and chief concerns [ICD/CC]); *Medium*: either (2) low plus c-SSRS scores from ED nursing evaluation (c-SSRS+ICD/CC) or (3) low plus MH diagnoses from primary treating ED physician evaluation (MH dx+ICD/CC); and *High*: (4) augmented case surveillance (aCS), which includes all available structured clinical data from the EHR. We categorized feature sets by dimensionality to understand the trade-off between model complexity and performance, where low-dimensional models are easier to implement but may miss important signals.

The 3 text feature sets were (5) NLP-general, (6) NLP-med, and (7) an open-source large language model (LLaMA). We selected these text approaches to evaluate detection gains while accounting for key trade-offs—dependency on sentence-labeled data (yes: NLP-general and NLP-med, no: LLaMA), computational resource requirements (higher graphics processing unit requirements: NLP-med, LLaMA; higher central processing unit requirements: NLP-general), and medical-specific versus light-weight embeddings (NLP-med vs NLP-general).

The 3 hybrid structured-text feature sets (8-10) combined aCS with each text approach.

#### Model Development and Validation

Encounters allocated to develop text processing methodology (n=724) were excluded. To assign the probabilities of SITB presence to the remaining encounters (n=3104), we developed 10 random forest classifiers [52], using 10-fold cross-validation with nested hyperparameter optimization [53]. A probability threshold of 0.5 was applied to convert random forest predictions into binary encounter-level classifications. For each outer fold, training data was split 50/50 for inner cross-validation. Hyperparameters were selected from the grid based on the highest classification accuracy in the inner CV. Each outer fold could select different optimal hyperparameters independently. The AUROC was calculated separately for each outer fold using the selected hyperparameters. The reported AUROC values represent the mean across all 10 outer folds with 95% CIs. The mean receiver operating characteristic (ROC) curve was created by interpolating individual fold ROC curves onto a common false positive rate grid and averaging the true positive rates. We selected this approach to maintain the integrity of the validation process and prevent data leakage by ensuring that hyperparameter tuning occurs only on training folds, with performance evaluation conducted on completely unseen validation data within each fold. Each encounter classifier’s individual features are specified in Multimedia Appendix 8.

### Statistical Analysis

#### Overall Classification Performance

We evaluated performance using AUROC, accuracy, sensitivity, specificity, positive predictive value, and negative predictive value. Shapley Additive Explanation values quantified feature importance, while permutation importance provided complementary ranking. Cross-validation variability was used to construct asymptotically exact CIs for test error [54]. Classifier performance was compared using DeLong tests [55].

#### Subgroup Performance

We assessed subgroup variation [56] by stratifying performance across demographic (age group, sex, race or ethnicity) and MH diagnosis (CAMHD-CS groups) subgroups. Each patient was assigned to 1 demographic subgroup but could belong to multiple diagnostic subgroups. For each subgroup, we calculated performance metrics with 95% CIs and generated ROC curves.

Analyses used Python 3.13.0 with scikit-learn, pytorch 1.9.0, and spacy 3.2.0. LLaMA inferencing used Hugging Face Transformers v4.49.0. The code is available upon request.

## Results

### Sample Characteristics

Our study sample included 3828 pediatric ED visits by unique youth ages 6‐17 and comprised 28,861 notes with 619,827 sentences. The sample included 1963 (51.3%) female and 1865 (48.7%) male youth, with the racial and ethnic composition of White non-Hispanic (n=1894, 49.5%), Hispanic or Latino (n=1017, 26.6%), Black (n=363, 9.5%), and Asian (n=178, 4.6%; Table 1). Adolescents (ages 13‐17 y) constituted most of the sample (n=2819, 73.6%), while children (ages 6‐12 y) represented 26.4% (n=1009). The median age was 15 (IQR 12‐16) years. Common psychiatric diagnoses included depressive disorders (n=1387, 36.2%), anxiety disorders (n=1161, 30.3%), suicide or self-injury coded diagnoses (n=1282, 33.5%), and attention-deficit or hyperactivity disorder (ADHD) (n=840, 21.9%). Suicide-related concerns comprised 18.5% (n=708) of the chief concerns.

**Table 1.** Sample characteristics^a^.

Sample characteristic	Value
Gold-standard, n (%)	3828 (100)
Any SITB^b^	1760 (46.0)
Suicide attempt	301 (7.9)
Preparatory acts	261 (6.8)
Suicidal ideation	1014 (26.5)
NSSI^c^	762 (19.9)
Other reason for visit	2036 (53.2)
Not enough information	33 (0.9)
Sex, n (%)	
Female	1963 (51.3)
Male	1865 (48.7)
Race and ethnicity, n (%)	
Not Hispanic or Latino	2774 (72.5)
American Indian or Alaska Native	10 (0.3)
Asian	178 (4.6)
Black or African American	363 (9.5)
Multiple races	88 (2.3)
Native Hawaiian or Other Pacific Islander	5 (0.1)
White	1894 (49.5)
Other race	235 (6.1)
Hispanic or Latino	1017 (26.6)
Unknown race or ethnicity	37 (1.0)
Site, n (%)	
Academic medical center	2858 (74.7)
Community hospital	970 (25.3)
Disposition, n (%)	
Discharged without hospitalization	2277 (59.5)
Hospitalized	1452 (37.9)
General medical hospitalization	390 (10.2)
Psychiatric hospitalization	1062 (27.7)
Other disposition	99 (2.6)
Chief concern, n (%)	
Psychiatric (including suicide-related)	2108 (55.1)
Suicide-related	708 (18.5)
ED^d^ Diagnostic code category (CAMHD-CS^e^), n (%)	
ADHD^f^	840 (21.9)
Anxiety disorders	1161 (30.3)
Autism spectrum disorder	468 (12.2)
Bipolar and related disorders	176 (4.6)
Depressive disorders	1387 (36.2)
Developmental disorder	81 (2.1)
Disruptive, impulse control, and conduct disorders	269 (7.0)
Feeding and eating disorders	106 (2.8)
Intellectual disability	66 (1.7)
Mental health symptom	535 (14.0)
Miscellaneous	202 (5.3)
Neurocognitive disorders	66 (1.7)
Obsessive-compulsive and related disorders	172 (4.5)
Schizophrenia and other psychotic disorders	145 (3.8)
Substance-related and addictive disorders	475 (12.4)
Suicide or self-injury	1282 (33.5)
Trauma and stressor-related disorders	246 (6.4)
≥2 CAMHD-CS^e^ diagnoses	2345 (61.3)
Age (y), median (IQR)	15 (12‐16)
Social vulnerability index, total, median (IQR)	0.38 (0.19‐0.65)
Area deprivation index, median (IQR)	
State ranking	2 (1-5)
National ranking	5 (2-12)

a Percentages do not sum to 100% as children may present with more than one chief concern or mental health diagnosis.

b SITB: self-injurious thoughts and behaviors.

c NSSI: nonsuicidal self-injury.

d ED: emergency department.

e CAMHD-CS: Child and Adolescent Mental Health Disorders Classification System.

f ADHD: attention-deficit or hyperactivity disorder.

### Ground-Truth Agreement

The raters agreed on SITB classification (3695/3828 [96.5% agreement]; Cohen κ=0.93). Nearly half (n=1760, 46.0%) of the encounters involved SITB, with similar prevalence in children (n=455, 45.1%) and adolescents (n=1305, 46.3%).

### Performance Metrics

#### Overview

**Figure 1.**
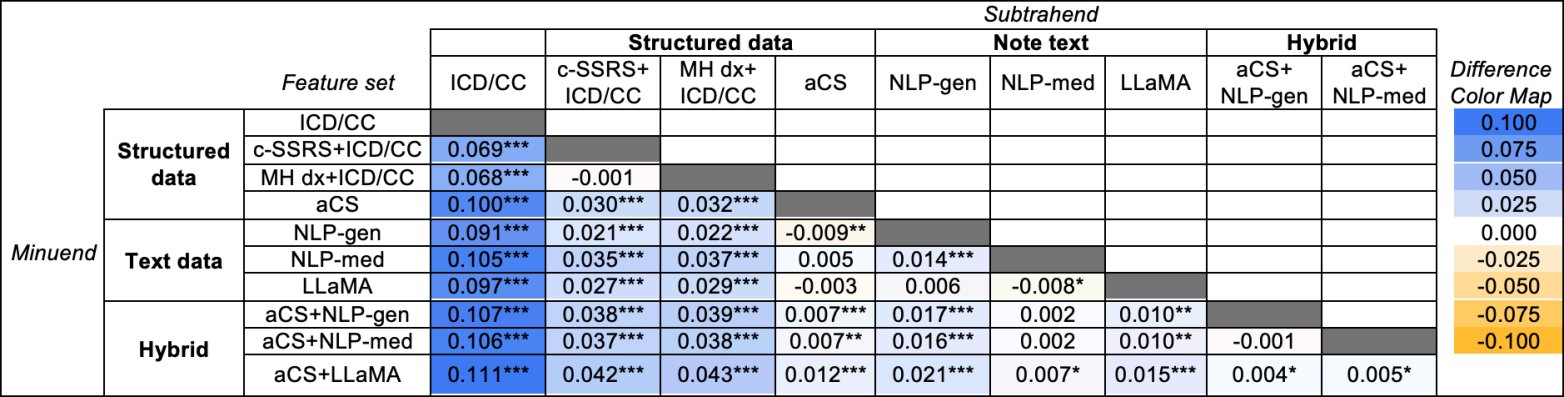
Comparison of detection classifiers for self-injurious thoughts and behaviors: pairwise analysis of area under the receiver operating curve (AUROC) by feature type. This heatmap illustrates the differences in AUROC values among 10 feature sets used for encounter classification. The matrix compares each pair of feature sets, with subtrahend feature sets listed in columns and minuend feature sets listed in rows. Cells shaded blue indicate that the row feature set outperformed the column feature set, while cells shaded yellow indicate inferior performance. The feature sets are categorized into 3 groups: structured (including International Classification of Diseases codes and chief concerns [ICD/CC], c-SSRS+ICD/CC, MH dx+ICD/CC, and augmented case surveillance [aCS]), text (including general-purpose natural language processing [NLP-general], medical text-specific trained natural language processing [NLP-med], and Large Language Model Meta AI [LLaMA]), and hybrid (including aCS+NLP, aCS+NLP-med, and aCS+LLaMA). Asterisks denote statistical significance (*P<.05, **P<.01, ***P<.001). The largest improvement in AUROC (0.111) occurred when high-dimensional structured data (aCS) were combined with open-source large language model scores (LLaMA), compared to the baseline ICD/CC feature set. c-SSRS: Columbia Suicide Severity Rating Scale.

**Figure 2.**
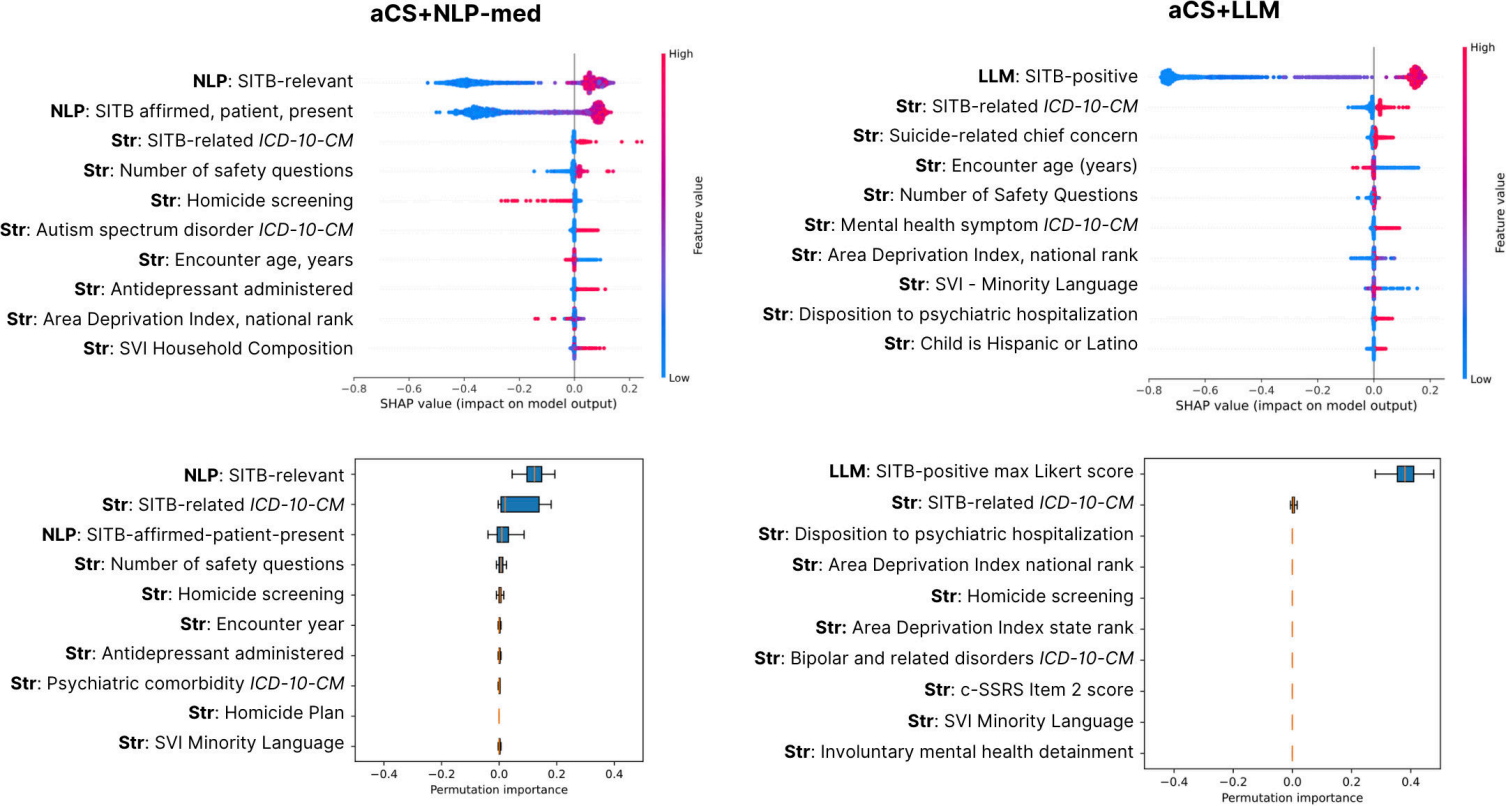
Comparison of feature and permutation importance between aCS+NLP-med and aCS+LLaMA for encounter classification of self-injurious thoughts and behaviors. This figure presents importance analyses for the 2 top-performing classification approaches, aCS+NLP-med (left panels) and aCS+LLaMA (right panels), which achieved the highest area under the receiver operating characteristic curve. The upper panels display Shapley Additive Explanation (SHAP) values, where negative values indicate the decreased detection of self-injurious thoughts and behaviors (SITB), and positive values indicate increased detection. The lower panels show permutation importance scores, which quantify the contribution of each feature to the classifier’s performance. In both classifiers, text features dominated the feature importance rankings, outperforming structured data features. Notable exceptions among structured data features included Columbia Suicide Severity Rating Scale (c-SSRS) items, homicide screening, area deprivation indices, encounter age, and psychiatric hospitalization disposition. For reference, the notation used in this figure is as follows: aCS represents all available structured data; NLP-med refers to note scores derived using MedEmbed-small-v0.1 embeddings with nearest-neighbor approximation; LLM refers to note scores generated by the open-source language model llama-3.3-70B. The "+" symbol indicates combinations of aCS with the corresponding text-based feature set (NLP-med or Large Language Model Meta AI [LLaMA]). aCS: augmented case surveillance; *ICD-10-CM*: *International Classification of Diseases Clinical Modification Version 10*; NLP-med: medical text-specific trained natural language processing; Str: structured data; SVI: social vulnerability index.

The detection of SITB varied across EHR data representations (Figure 1). Complete fit metrics, failure mode characterization including the number of false positives and false negatives, exact DeLong test *P* values, and ROC curves are provided in MultimediaAppendices 9  10. AUROC stability across folds is visualized in Multimedia Appendix 11. The examination of Shapley Additive Explanations and permutation importances for the best-performing representations revealed that text-derived features provided the strongest contribution to classifier accuracy (Figure 2; MultimediaAppendices 12  13).

#### Structured Data Classification

Low-dimensional structured (ICD/CC) yielded the lowest accuracy detection (AUROC 0.865, 95% CI 0.852‐0.879). Both medium-dimensional structured (c-SSRS+ICD/CC; MH dx+ICD/CC) feature sets outperformed ICD/CC (both *P*<.001). The high-dimensional structured feature set (aCS) (AUROC 0.965, 95% CI 0.958‐0.972) further outperformed c-SSRS+ICD/CC (AUROC 0.935, 95% CI 0.925‐0.944) and MH dx+ICD/CC (AUROC 0.934, 95% CI 0.924‐0.943; *P*<.001).

#### Text-Based Classification

Among text modalities, NLP-med (AUROC 0.970, 95% CI 0.964‐0.977) marginally outperformed NLP-general (AUROC 0.956, 95% CI 0.948‐0.964; *P*<.001) and LLaMA (AUROC 0.962, 95% CI 0.955‐0.969; *P*=.03). The text-only modalities all surpassed ICD/CC as well as c-SSRS+ICD/CC and MH dx+ICD/CC (all *P*<.001), but NLP-general was slightly inferior to aCS (*P*=.005). NLP-med and LLaMA did not significantly exceed aCS.

#### Hybrid Classification

Combining text with aCS exceeded aCS alone (*P*<.001). Combining text with aCS also exceeded NLP-general alone (*P*<.001) and LLaMA alone (*P*<.01). However, adding aCS to NLP-med did not improve detection compared with NLP-med alone (*P*=.633). The hybrid representation combining aCS with LLaMA classification (aCS+LLaMA) achieved the highest overall AUROC (0.977, 95% CI 0.971‐0.982), narrowly exceeding aCS with NLP-med (AUROC 0.970, 95% CI 0.964‐0.977; *P*=.04).

### Subgroup Performance

#### Demographic Subgroups

**Figure 3.**
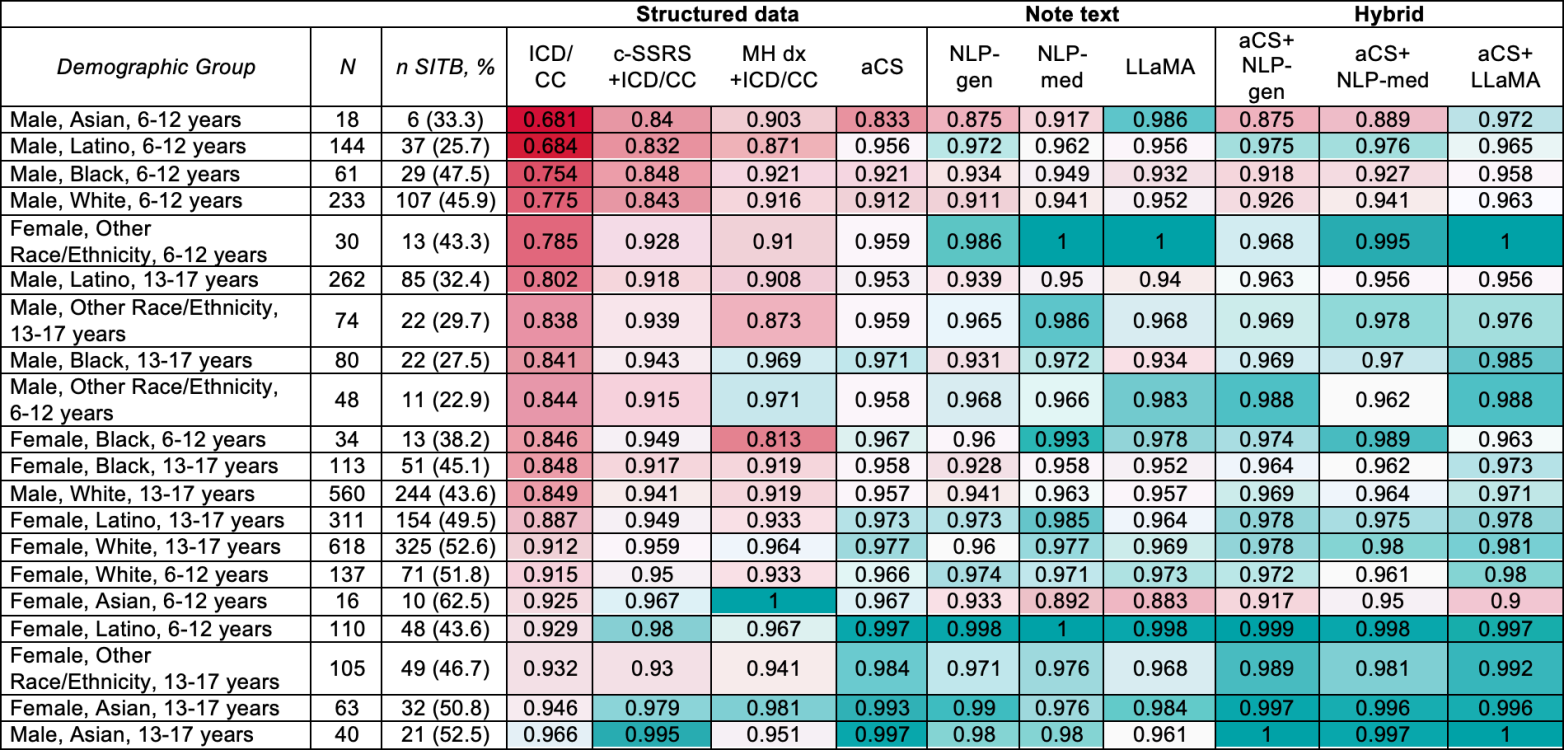
Stratified performance of detection classifiers by demographics. This figure presents the area under the receiver operating characteristic curve (AUROC) values for various encounter classification feature sets, stratified by age (6-12 and 13-17 y), sex (male and female), and race or ethnicity (Asian, Black, Hispanic or Latino, White, and Other), with the number of cases with self-injurious thoughts and behaviors (SITB Pos) shown for each subgroup. The feature sets are categorized into 3 groups: structured (including International Classification of Diseases codes and chief concerns [ICD/CC], c-SSRS+ICD/CC, MH dx+ICD/CC, and augmented case surveillance [aCS]), text (including general-purpose natural language processing [NLP-gen], medical text-specific trained natural language processing [NLP-med], and Large Language Model Meta AI [LLaMA]), and hybrid (including aCS+NLP, aCS+NLP-med, and aCS+LLaMA). The results show that baseline classifiers using only ICD codes or chief concerns had lower performance (AUROC range: 0.681-0.966), whereas more comprehensive classifiers, particularly those combining structured data (aCS) with natural language processing (NLP or LLaMA), achieved higher performance across all demographic subgroups (AUROC range: 0.900-1.000), as indicated by the color gradient from teal (higher performance) to red (lower performance), with the "Other" race or ethnicity category including individuals who identify as multiple races, Native Hawaiian or Pacific Islander, Native American or Alaska Native, or have an unknown race or ethnicity.

Detection varied considerably across age, sex, and race or ethnicity subgroups (Figure 3; Multimedia Appendix 14). Low-dimensional structured data (ICD/CC) achieved AUROC values ≥0.950 for only 2/20 demographic groups. ICD/CC performed less well for children (AUROC 0.821, 95% CI 0.791‐0.851) compared to adolescents (AUROC 0.880, 95% CI 0.865‐0.895) and for male (AUROC 0.817, 95% CI 0.794‐0.840) compared to female (AUROC 0.902; 95% CI 0.886‐0.918) youth, with nonoverlapping CIs. Detection was similar between female children and female adolescents (AUROC 0.903, 95% CI 0.867‐0.939 vs AUROC 0.902, 95% CI 0.884‐0.920) but differed between male children and male adolescents (AUROC 0.753, 95% CI 0.708‐0.798 vs AUROC 0.847, 95% CI 0.821‐0.873). Using ICD/CC alone, detection was lower among Hispanic male children (AUROC 0.684, 95% CI 0.579‐0.789) and Black male children (AUROC 0.754, 95% CI 0.630‐0.877), with a similar trend among Asian male children. Multimedia Appendices 16-19 present ROC curves stratified by feature set and demographic groups.

Hybrid feature sets achieved the greatest subgroup consistency, with AUROC values ≥0.950 for aCS+NLP-general (15/20 demographic groups), aCS+NLP-med (17/20 demographic groups), and aCS+LLaMA (19/20 demographic groups). aCS+LLaMA yielded the most consistent performance, reducing the AUROC gap between the highest and lowest performing groups from 0.285 (ICD/CC) to 0.100 (aCS+LLaMA). Notably, aCS+LLaMA achieved strong detection performance for groups with the lowest detection performance using ICD/CC alone, including Hispanic male children and Black male children, with AUROC improvements of 0.281 and 0.205, respectively.

#### Diagnostic Subgroups

**Figure 4.**
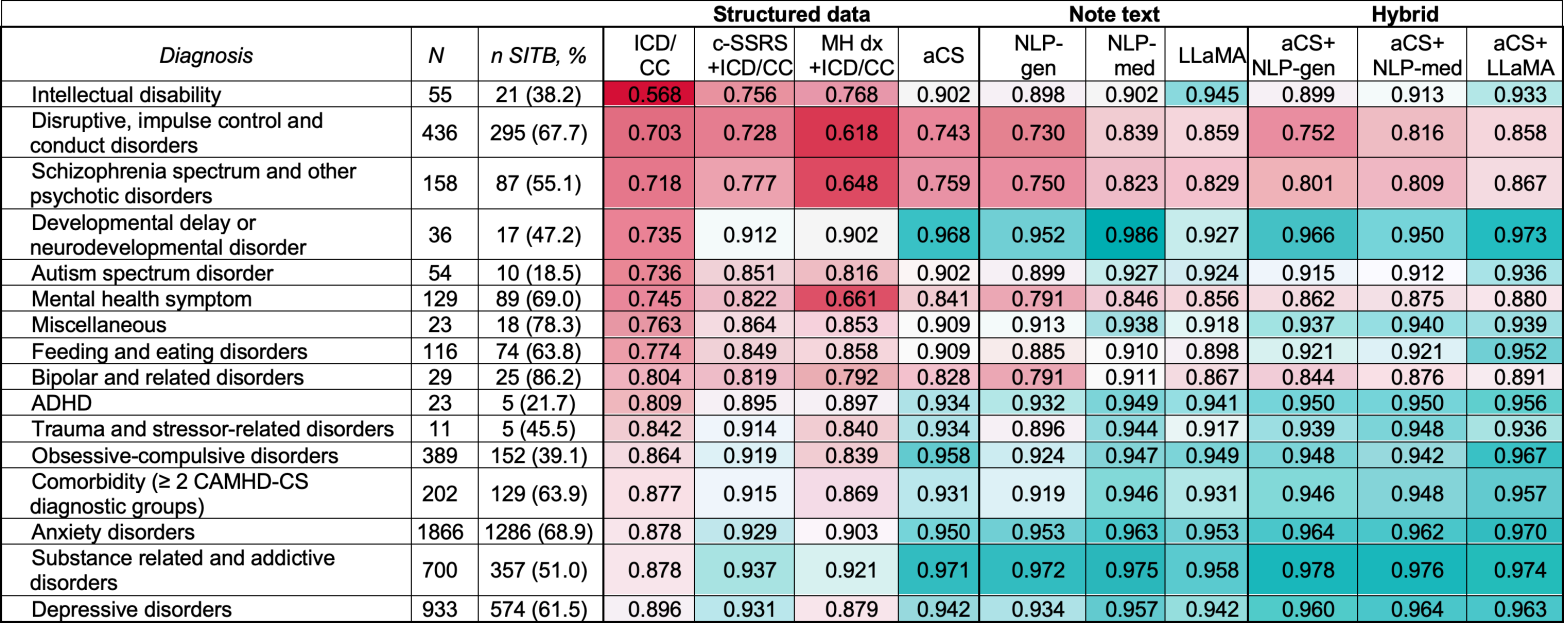
Stratified performance of detection classifiers by mental health diagnosis. This figure presents the area under the receiver operating characteristic curve (AUROC) values for various encounter classification feature sets, stratified by the 15 most prevalent diagnostic categories of the Child and Adolescent Mental Health Disorders Classification System (CAMHD-CS), with the number of self-injurious thoughts and behaviors (SITB)−positive cases shown for each subgroup. For performance by all 23 categories, see Multimedia Appendix 13. The feature sets are categorized into 3 groups: structured (including International Classification of Diseases codes and chief concerns [ICD/CC], c-SSRS+ICD/CC, MH dx+ICD/CC, and augmented case surveillance [aCS]), text (including general-purpose natural language processing [NLP-gen], medical text-specific trained natural language processing [NLP-med], and Large Language Model Meta AI [LLaMA]), and hybrid (including aCS+NLP, aCS+NLP-med, and aCS+LLaMA). The results show that classifiers performed best in identifying SITB risk for substance-related and addictive disorders, anxiety disorders, and developmental delay disorders across most feature sets. Notably, classifiers that integrated structured data (aCS) with natural language processing (NLP) or large language model (LLaMA) approaches generally achieved higher performance compared to individual feature sets alone, as indicated by the color gradient from teal (higher performance) to red (lower performance). ADHD: attention-deficit or hyperactivity disorder.

Detection further varied by MH diagnostic categories (Figure 4; Multimedia Appendix 19). ICD/CC achieved AUROC values ≥0.90 for 0/15 diagnostic groups. ICD/CC achieved lower SITB detection performance among youth with neurodevelopmental (eg, intellectual disability: AUROC 0.568, 95% CI 0.410‐0.726; autism spectrum disorder: AUROC 0.736, 95% CI 0.686‐0.787; ADHD: AUROC 0.809, 95% CI 0.777‐0.841), externalizing (disruptive or impulse control disorders: AUROC 0.703, 95% CI 0.635‐0.772), and psychotic (AUROC 0.718, 95% CI 0.626‐0.811) disorders. In contrast, ICD/CC achieved higher SITB detection performance among youth with internalizing (eg, depressive disorders: AUROC 0.896, 95% CI 0.878‐0.915; anxiety disorders: AUROC 0.878, 95% CI 0.856‐0.899; trauma- or stressor-related disorders: AUROC 0.842, 95% CI 0.789‐0.895) and substance-related disorders (AUROC 0.878, 95% CI 0.840‐0.917).

Hybrid feature sets achieved the greatest subgroup consistency among diagnostic groups, with AUROC exceeding 0.950 for aCS+NLP-general (5/15 diagnostic groups), aCS+NLP-med (5/15 diagnostic groups), and aCS+LLaMA (8/15 diagnostic groups). aCS+LLaMA yielded the most consistent performance, reducing the AUROC gap between the highest and lowest performing groups from 0.328 (ICD/CC classifier) to 0.115. Notably, aCS+ LLaMA achieved strong detection performance for the groups with lower detection performance using ICD/CC alone, including neurodevelopmental problems (intellectual disability: AUROC 0.933, 95% CI 0.854‐1.000; developmental delay: AUROC 0.973, 95% CI 0.910‐1.000; autism spectrum disorder: AUROC 0.936, 95% CI 0.909‐0.963; ADHD: AUROC 0.956, 95% CI 0.941‐0.972), externalizing problems (disruptive or impulse control disorders: AUROC 0.858, 95% CI 0.809‐0.908), and psychotic disorders (AUROC 0.867, 95% CI 0.803‐0.931). However, the detection of SITB among children with externalizing and psychotic disorders remained lower for internalizing disorders (eg, depression: AUROC 0.963, 95% CI 0.953‐0.973).

## Discussion

### Principal Findings

In this cross-sectional study, integrating comprehensive structured data with clinical notes substantially improved the detection of pediatric ED service use for suicide and self-harm. Hybrid modality classifiers combining high-dimensional structured data with an open-source language model scores achieved the highest performance across nearly all subgroups—advancing detection accuracy beyond prior efforts relying on *ICD-10-CM* codes or clinical text alone [17,35,57,58]. Surprisingly, detection using high-dimensional structured data approximated text-based approaches, providing a resource-efficient alternative to improve detection while simplifying anonymization and computational requirements.

Our findings challenge the widespread reliance on suicide- and self-harm−related *ICD-10-CM* codes and chief concern for identifying SITB emergency service use among children. While epidemiologic studies report female adolescents account for the surge in emergency service use for suicidality [59,60], the misclassification of SITB among male children may distort observed patterns of pediatric ED utilization. This detection gap raises particular concern given the annual 8.2% rise in suicide death rates among preteens [59] and the highest age-standardized suicide death rates among male US youth aged 10‐24 years across 52 countries [61]. Youth with psychotic disorders or neurodevelopmental disorders also presented detection challenges despite their markedly elevated risk—70-fold elevated risk of suicide attempts [62] and 3-fold elevated risk of suicide death [63,64], respectively. For these populations, clinical text analysis offers advantages, possibly by capturing subtle manifestations of distress such as irritability, perceptual disturbances, and aggression. Future phenotyping studies should implement systematic bias auditing protocols that regularly evaluate detection accuracy across demographic and diagnostic subgroups to identify and remediate performance disparities before clinical deployment.

The detection of pediatric ED service use for suicide and self-harm has key implications for clinical practice, health system operations, and public health surveillance. Better detection underpins the development of clinical decision support tools to guide clinician awareness of suicide risk and promote delivery of evidence-based suicide prevention interventions such as safety planning [65,66] and lethal means safety counseling [67,68]. Youth with serious mental illness and developmental disorders—among the most frequently undetected groups—are also the highest ED utilizers [59] and experience extended boarding times [69]. Investment in sensitive, efficient SITB detection methods is likely to yield significant returns through forecasting resources, alleviating ED crowding, and reducing ED recidivism. Further, narrowing detection gaps could enable more precise monitoring during crisis periods, such as natural disasters and suicide clusters.

### Limitations

While this study included 2 EDs in a single health system, generalizability requires external validation in other health systems, particularly in low-resourced community-based settings. Some children with SITB present to health care settings for non-MH reasons. To balance maximizing unique individuals while maintaining feasibility for human annotation, we analyzed only the most recent MH-related visit per child. Model performance was evaluated using retrospective data that may not reflect evolving clinical documentation practices, changes in suicide screening protocols, or shifts in patient presentation patterns over time. While feature dimensions are invariant to note length, it is possible extreme differences in documentation volume could influence the accuracy of note-derived scores; however, our observation of decreased heterogeneity with the use of text suggests that note-derived features are capturing clinical patterns across subgroups despite any unmeasured documentation differences. Calibration, while essential for clinical deployment, was outside our scope of comparing data modalities’ relative discriminative power. There are numerous practical challenges involved in deploying NLP methodologies in real-time clinical settings, including the computational cost and necessary implementation infrastructure. Future research should focus on prospective validation in diverse clinical settings, implementation studies examining workflow integration and clinician acceptance, cost-effectiveness analyses, and evaluation of model degradation over time. In the interim, this study offers actionable approaches to strengthening retrospective surveillance of pediatric suicide−related ED use. Real-time EHR integration would require robust model maintenance protocols, comprehensive staff training on result interpretation, patient and family input on automated screening approaches, and ongoing bias monitoring.

### Conclusions

This study developed a cross-disciplinary and multimodal machine learning approach for automating the detection of pediatric SITB−related emergency care using integrated EHR data representations. The hybrid modality achieved high accuracy while demonstrating reduced variation across demographic and diagnostic subgroups compared with basic structured data alone. The findings indicate that, alone, *ICD-10-CM* codes and chief concerns yield suboptimal and variable detection accuracy. Study methods provide computationally efficient alternatives to improve detection accuracy beyond traditional approaches. The findings suggest that systematic detection gaps exist and can be efficiently mitigated: focused efforts to augment information retrieval on suicide risk factors at bedside are needed to stymie decision bias and bolster pediatric MH care quality.

## Supplementary material

10.2196/82371 Multimedia Appendix 1 Flow diagram for study inclusion.

10.2196/82371 Multimedia Appendix 2 Variable construction.

10.2196/82371 Multimedia Appendix 3 Mental health−related chief complaints in structured data fields.

10.2196/82371 Multimedia Appendix 4 Methods to handle missingness.

10.2196/82371 Multimedia Appendix 5 Chart annotation guide.

10.2196/82371 Multimedia Appendix 6 Software implementation.

10.2196/82371 Multimedia Appendix 7 Community advisory board.

10.2196/82371 Multimedia Appendix 8 Variables comprising feature sets.

10.2196/82371 Multimedia Appendix 9 Detection performance by classifier.

10.2196/82371 Multimedia Appendix 10 DeLong tests (*P* value) by feature set.

10.2196/82371 Multimedia Appendix 11 Area under the receiver operating characteristic (AUROC) curves by feature set.

10.2196/82371 Multimedia Appendix 12 Shapley Additive Explanations plots by feature set.

10.2196/82371 Multimedia Appendix 13 Permutation feature importance by feature set.

10.2196/82371 Multimedia Appendix 14 Detection performance by classifier and age group and by sex and race or ethnicity.

10.2196/82371 Multimedia Appendix 15 Area under the receiver operating characteristic curves (AUROC) by feature set by sex and age group (children).

10.2196/82371 Multimedia Appendix 16 Area under the receiver operating characteristic (AUROC) curves by feature set by sex and age group (adolescents).

10.2196/82371 Multimedia Appendix 17 Area under the receiver operating characteristic (AUROC) curves by feature set by race or ethnicity (children).

10.2196/82371 Multimedia Appendix 18 Area under the receiver operating characteristic (AUROC) curves by feature set by race or ethnicity (adolescents).

10.2196/82371 Multimedia Appendix 19 Detection performance by classifier and emergency department mental health diagnosis.

10.2196/82371 Checklist 1 STROBE and TRIPOD checklists.

## Abbreviations:

aCS: augmented case surveillance
ADHD: attention-deficit or hyperactivity disorder
AUROC: area under the receiver operating characteristic curve
c-SSRS: Columbia Suicide Severity Rating Scale
CAMHD-CS: Child and Adolescent Mental Health Disorders Classification System
ED: emergency department
EHR: electronic health record
*ICD-10-CM*: *International Classification of Diseases, 10th Revision, Clinical Modification*
ICD/CC: International Classification of Diseases codes and chief concerns
LLaMA: Large Language Model Meta AI
MH: mental health
NLP: natural language processing
ROC: receiver operating characteristic
SITB: self-injurious thoughts and behaviors
STROBE: Strengthening the Reporting of Observational Studies in Epidemiology
TRIPOD: Transparent Reporting of a Multivariable Prediction Model for Individual Prognosis or Diagnosis

## References

[1] Centers for disease control and prevention. WISQARS Data Visualization.

[2] Peterson C, Haileyesus T, Stone DM (2024). Economic cost of US suicide and nonfatal self-harm. Am J Prev Med.

[3] Tsui FR, Shi L, Ruiz V (2021). Natural language processing and machine learning of electronic health records for prediction of first-time suicide attempts. JAMIA Open.

[4] Yoshimasu K, Kiyohara C, Miyashita K, Stress Research Group of the Japanese Society for Hygiene (2008). Suicidal risk factors and completed suicide: meta-analyses based on psychological autopsy studies. Environ Health Prev Med.

[5] Ribeiro JD, Franklin JC, Fox KR (2016). Self-injurious thoughts and behaviors as risk factors for future suicide ideation, attempts, and death: a meta-analysis of longitudinal studies. Psychol Med.

[6] Stang PE, Ryan PB, Racoosin JA (2010). Advancing the science for active surveillance: rationale and design for the observational medical outcomes partnership. Ann Intern Med.

[7] (2018). Practice Manual for Establishing and Maintaining Surveillance Systems for Suicide Attempts and Self-Harm.

[8] Gahm GA, Reger MA, Kinn JT, Luxton DD, Skopp NA, Bush NE (2012). Addressing the surveillance goal in the national strategy for suicide prevention: the department of defense suicide event report. Am J Public Health.

[9] Metzger MH, Tvardik N, Gicquel Q, Bouvry C, Poulet E, Potinet-Pagliaroli V (2017). Use of emergency department electronic medical records for automated epidemiological surveillance of suicide attempts: a French pilot study. Int J Methods Psychiatr Res.

[10] Emergency Department Surveillance of Nonfatal Suicide-Related Outcomes (ED-SNRO). Centers for Disease Control and Prevention.

[11] (2024). 2024 National Strategy for Suicide Prevention. Centers for Disease Control.

[12] Bonilla AG, Pourat N, Chuang E (2021). Mental health staffing at HRSA-funded health centers may improve access to care. Psychiatr Serv.

[13] Gross TK, Lane NE, Timm NL, Committee on Pediatric Emergency Medicine (2023). Crowding in the emergency department: challenges and best practices for the care of children. Pediatrics.

[14] Roberts BK, Nofi CP, Cornell E, Kapoor S, Harrison L, Sathya C (2023). Trends and disparities in firearm deaths among children. Pediatrics.

[15] Naik-Mathuria BJ, Cain CM, Alore EA, Chen L, Pompeii LA (2023). Defining the full spectrum of pediatric firearm injury and death in the United States: it is even worse than we think. Ann Surg.

[16] Cantor J, Schuler MS, Kerber R, Purtle J, McBain RK (2025). Changes in specialty crisis services offered before and after the launch of the 988 suicide and crisis lifeline. JAMA Psychiatry.

[17] Boggs JM, Kafka JM (2022). A critical review of text mining applications for suicide research. Curr Epidemiol Rep.

[18] Zwald ML, Holland KM, Annor F (2019). Monitoring suicide-related events using National Syndromic Surveillance Program data. Online J Public Health Inform.

[19] Bey R, Cohen A, Trebossen V (2024). Natural language processing of multi-hospital electronic health records for public health surveillance of suicidality. NPJ Mental Health Res.

[20] Edgcomb JB, Tseng CH, Pan M, Klomhaus A, Zima BT (2023). Assessing detection of children with suicide-related emergencies: evaluation and development of computable phenotyping approaches. JMIR Ment Health.

[21] Edgcomb JB, Olde Loohuis L, Tseng CH (2024). Electronic health record phenotyping of pediatric suicide-related emergency department visits. JAMA Netw Open.

[22] Rossom RC, Richards JE, Sterling S (2022). Connecting research and practice: implementation of suicide prevention strategies in learning health care systems. Psychiatr Serv.

[23] Oexle N, Feigelman W, Sheehan L (2020). Perceived suicide stigma, secrecy about suicide loss and mental health outcomes. Death Stud.

[24] Rockett IRH, Wang S, Stack S (2010). Race/ethnicity and potential suicide misclassification: window on a minority suicide paradox?. BMC Psychiatry.

[25] Green C, Gottschlich EA, Burr WH (2023). A national survey of pediatricians' experiences and practices with suicide prevention. Acad Pediatr.

[26] Horowitz LM, Bridge JA, Tipton MV (2022). Implementing suicide risk screening in a pediatric primary care setting: from research to practice. Acad Pediatr.

[27] Downs J, Velupillai S, George G (2018). Detection of suicidality in adolescents with autism spectrum disorders: developing a natural language processing approach for use in electronic health records. AMIA Annu Symp Proc.

[28] Oquendo MA, Mann JJ (2008). Suicidal behavior: a developmental perspective. Psychiatr Clin North Am.

[29] Hedegaard H, Schoenbaum M, Claassen C, Crosby A, Holland K, Proescholdbell S (2018). Issues in developing a surveillance case definition for nonfatal suicide attempt and intentional self-harm using International Classification of Diseases, Tenth Revision, Clinical Modification (ICD-10-CM) coded data. Natl Health Stat Report.

[30] Hoge MA, Vanderploeg J, Paris M, Lang JM, Olezeski C (2022). Emergency department use by children and youth with mental health conditions: a health equity agenda. Community Ment Health J.

[31] Obeid JS, Dahne J, Christensen S (2020). Identifying and predicting intentional self-harm in electronic health record clinical notes: deep learning approach. JMIR Med Inform.

[32] Ji S, Pan S, Li X, Cambria E, Long G, Huang Z (2021). Suicidal ideation detection: a review of machine learning methods and applications. IEEE Trans Comput Soc Syst.

[33] Adekkanattu P, Furmanchuk A, Wu Y (2024). Deep learning for identifying personal and family history of suicidal thoughts and behaviors from EHRs. NPJ Digit Med.

[34] Bunnell BE, Tsalatsanis A, Chaphalkar C (2025). Automated detection and prediction of suicidal behavior from clinical notes using deep learning. PLoS ONE.

[35] Martinez-Romo J, Araujo L, Reneses B (2025). Guardian-BERT: early detection of self-injury and suicidal signs with language technologies in electronic health reports. Comput Biol Med.

[36] Bedi S, Liu Y, Orr-Ewing L (2025). Testing and evaluation of health care applications of large language models: a systematic review. JAMA.

[37] Zima BT, Gay JC, Rodean J (2020). Classification system for International Classification of Diseases, Ninth Revision, Clinical Modification and Tenth Revision pediatric mental health disorders. JAMA Pediatr.

[38] Posner K, Brown GK, Stanley B (2011). The Columbia-Suicide Severity Rating Scale: initial validity and internal consistency findings from three multisite studies with adolescents and adults. Am J Psychiatry.

[39] Carson NJ, Mullin B, Sanchez MJ (2019). Identification of suicidal behavior among psychiatrically hospitalized adolescents using natural language processing and machine learning of electronic health records. PLoS ONE.

[40] Velupillai S, Epstein S, Bittar A, Stephenson T, Dutta R, Downs J (2019). Identifying suicidal adolescents from mental health records using natural language processing. Stud Health Technol Inform.

[41] Cusick M, Velupillai S, Downs J (2022). Portability of natural language processing methods to detect suicidality from clinical text in US and UK electronic health records. J Affect Disord Rep.

[42] Coley RY, Johnson E, Simon GE, Cruz M, Shortreed SM (2021). Racial/ethnic disparities in the performance of prediction models for death by suicide after mental health visits. JAMA Psychiatry.

[43] von Elm E, Altman DG, Egger M (2014). The Strengthening the Reporting of Observational Studies in Epidemiology (STROBE) Statement: guidelines for reporting observational studies. Int J Surg.

[44] Collins GS, Moons KGM, Dhiman P (2024). TRIPOD+AI statement: updated guidance for reporting clinical prediction models that use regression or machine learning methods. BMJ.

[45] Gallifant J, Afshar M, Ameen S (2025). The TRIPOD-LLM reporting guideline for studies using large language models. Nat Med.

[46] NOT-OD-15-089: racial and ethnic categories and definitions for NIH diversity programs and for other reporting purposes. National Institutes of Health.

[47] (2020). Social vulnerability index. Agency for Toxic Substances and Disease Registry.

[48] Maroko AR, Doan TM, Arno PS, Hubel M, Yi S, Viola D (2016). Integrating social determinants of health with treatment and prevention: a new tool to assess local area deprivation. Prev Chronic Dis.

[49] Posner K, Oquendo MA, Gould M, Stanley B, Davies M (2007). Columbia Classification Algorithm of Suicide Assessment (C-CASA): classification of suicidal events in the FDA’s pediatric suicidal risk analysis of antidepressants. Am J Psychiatry.

[50] Zhang Y, Cai T, Yu S (2019). High-throughput phenotyping with electronic medical record data using a common semi-supervised approach (PheCAP). Nat Protoc.

[51] Aumüller M, Bernhardsson E, Faithfull A (2020). ANN-Benchmarks: a benchmarking tool for approximate nearest neighbor algorithms. Inf Syst.

[52] Breiman L (2001). Random forests. Mach Learn.

[53] Probst P, Wright MN, Boulesteix AL (2019). Hyperparameters and tuning strategies for random forest. WIREs Data Min Knowl.

[54] Bayle P, Bayle A, Janson L, Mackey L (2020). Proceedings of the 34th International Conference on Neural Information Processing Systems.

[55] DeLong ER, DeLong DM, Clarke-Pearson DL (1988). Comparing the areas under two or more correlated receiver operating characteristic curves: a nonparametric approach. Biometrics.

[56] Kearns M, Neel S, Roth A, Wu ZS (2018). Preventing fairness gerrymandering: auditing and learning for subgroup fairness. https://proceedings.mlr.press/v80/kearns18a/kearns18a.pdf.

[57] Sveticic J, Stapelberg NCJ, Turner K (2020). Suicidal and self-harm presentations to emergency departments: the challenges of identification through diagnostic codes and presenting complaints. Health Inf Manag.

[58] Arias SA, Boudreaux ED, Chen E (2019). Which chart elements accurately identify emergency department visits for suicidal ideation or behavior?. Arch Suicide Res.

[59] Bommersbach TJ, McKean AJ, Olfson M, Rhee TG (2023). National trends in mental health–related emergency department visits among youth, 2011-2020. JAMA.

[60] Anderson KN, Johns D, Holland KM (2023). Emergency department visits involving mental health conditions, suicide-related behaviors, and drug overdoses among adolescents - United States, January 2019-February 2023. MMWR Morb Mortal Wkly Rep.

[61] Bertuccio P, Amerio A, Grande E (2024). Global trends in youth suicide from 1990 to 2020: an analysis of data from the WHO mortality database. EClinicalMedicine.

[62] Pompili M, Serafini G, Innamorati M (2011). Suicide risk in first episode psychosis: a selective review of the current literature. Schizophr Res.

[63] Kirby AV, Bakian AV, Zhang Y, Bilder DA, Keeshin BR, Coon H (2019). A 20-year study of suicide death in a statewide autism population. Autism Res.

[64] Kõlves K, Fitzgerald C, Nordentoft M, Wood SJ, Erlangsen A (2021). Assessment of suicidal behaviors among individuals with autism spectrum disorder in Denmark. JAMA Netw Open.

[65] Boggs JM, Yarborough BJH, Clarke G (2025). Development and validation of electronic health record measures of safety planning practices as part of zero suicide implementation. Arch Suicide Res.

[66] Reyes-Portillo JA, Chin EM, Toso-Salman J, Blake Turner J, Vawdrey D, Mufson L (2018). Using electronic health record alerts to increase safety planning with youth at-risk for suicide: a non-randomized trial. Child Youth Care Forum.

[67] Bandealy A, Herrera N, Weissman M, Scheidt P (2020). Use of lethal means restriction counseling for suicide prevention in pediatric primary care. Prev Med.

[68] Sisler SM, Hart S, Hamilton J, Schapiro NA (2025). Preventing suicide through lethal means restriction in pediatric care. J Pediatr Health Care.

[69] Hoffmann JA, Stack AM, Monuteaux MC, Levin R, Lee LK (2019). Factors associated with boarding and length of stay for pediatric mental health emergency visits. Am J Emerg Med.

