# Supplementary material for: Detecting Pediatric Emergency Service Use for Suicide and Self-Harm: Multimodal Analysis of 3828 Encounters
Source: JMIR Ment Health. 2026 Feb 4;13:e82371. doi: 10.2196/82371 (PMC12871580; doi:10.2196/82371)
Supplement: Multimedia Appendix 1 [file mental-v13-e82371-s001.docx]

**
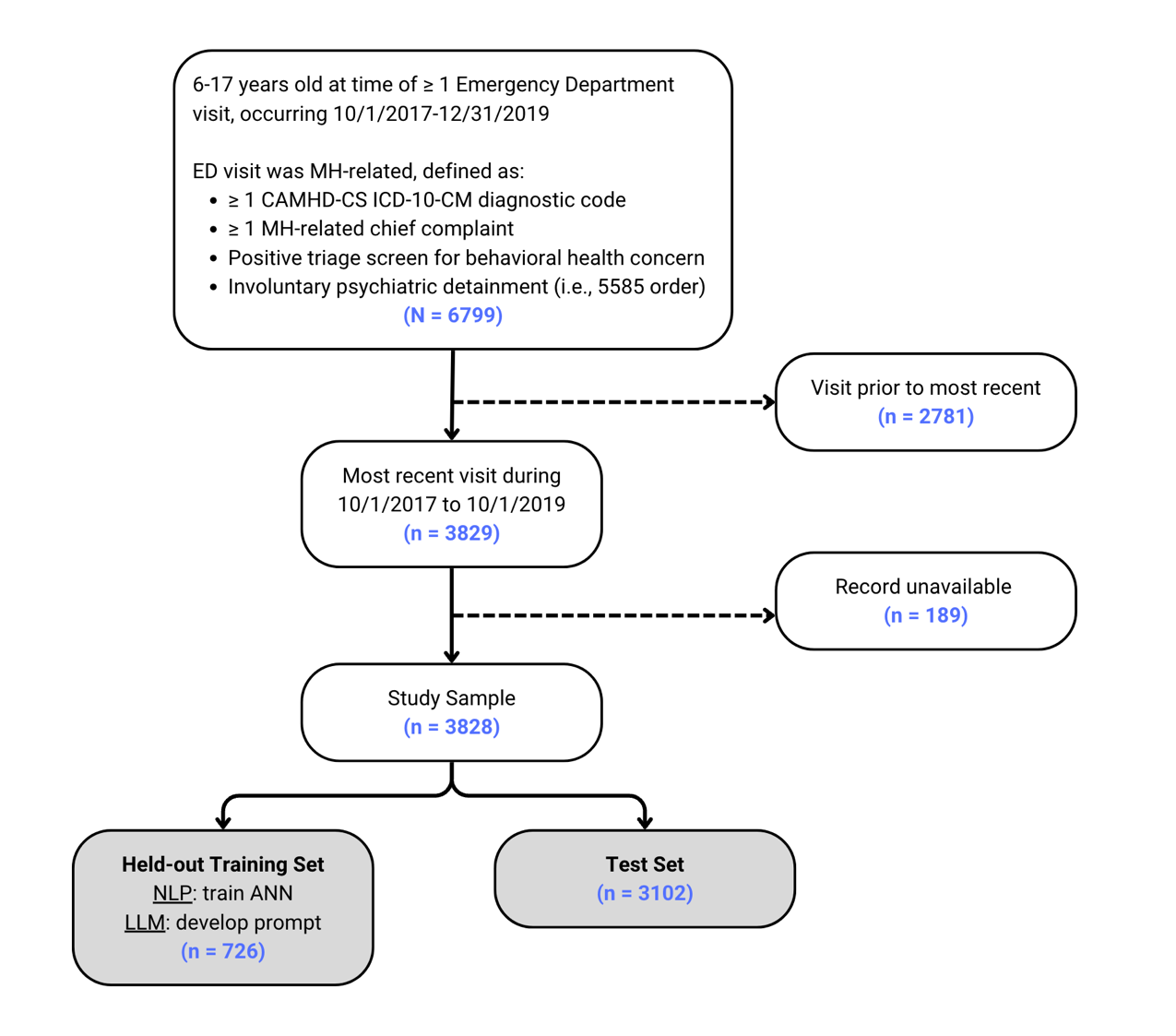
**

**Multimedia Appendix 1. Flow Diagram for Study Inclusion**

*ED: Emergency Department, CAMDH-CS: Child and Adolescent Mental Health Disorders Classification System, ICD-10-CM: International Classification of Diseases, Version 10, Clinical Modification*, *MH: Mental Health.*
