# Supplementary material for: Detecting Pediatric Emergency Service Use for Suicide and Self-Harm: Multimodal Analysis of 3828 Encounters
Source: JMIR Ment Health. 2026 Feb 4;13:e82371. doi: 10.2196/82371 (PMC12871580; doi:10.2196/82371)
Supplement: Multimedia Appendix 2 [file mental-v13-e82371-s002.docx]

**Multimedia Appendix 2: Variable Construction**

**Youth Characteristics:**

*Race and ethnicity:* Patient or parent-reported racial and ethnic categories aligned with OMB Revisions to the Standards for the Classification of Federal Data on Race and Ethnicity (NOT-OD-15-053): American Indian or Alaska Native, Asian, Black or African American, Hispanic or Latino, Native Hawaiian or Other Pacific Islander, and White. Due to low sample size, during comparison of model performance across demographic groups, the category "other race and ethnicity" included American Indian or Alaska Native, Native Hawaiian or Other Pacific Islander, and individuals identifying with multiple races and ethnicities.

*Sex:* Patient or caregiver-reported legal sex.

*Language*: Preferred language (English vs. Other), as reported by the youth or caregiver.

**Encounter Characteristics:**

*Site:* Site was encoded as a binary variable representing primary location of ED visit: quaternary care academic medical center or community hospital.

*Disposition:* ED disposition was categorized as discharged, general medical hospitalization, psychiatric hospitalization (any, within, and outside of health system), transferred, eloped, left without being seen, deceased, and sent to labor and delivery.

*Chief concern:* Patient-reported reason for presentation to the ED, as entered by ED triage nurse. Chief concerns considered mental health-related are defined in eTable 1.

*Diagnoses:* International Classification of Diseases Version 10 Clinical Modification (ICD-10-CM) codes in any diagnostic field associated with the ED encounter were encoded into categories using the Child and Adolescent Mental Health Disorders Classification System (CAMHD-CS). Additional categories included presence of comorbidity (≥2 CAMHD-CS categories), and presence of medical diagnoses not listed in CAMHD-CS. The CAMHD-CS category for suicide and self-harm aligns with the ICD-10-CM list described by Hedegaard et al. in their 2018 National health statistics report on developing surveillance case definitions for nonfatal suicide attempts and intentional self-harm.

*Legal status*: Legal status was defined as presence of a 5585 mental health detainment order (any, none) and an additional binary variable coded whether the detainment was continued following mental health evaluation.

*Columbia Suicide Severity Rating Scale (c-SSRS) Screening*. The c-SSRS was administered to all youth with a behavioral health concern beginning in 2016 to assess suicide risk, with items 1-6 measuring escalating severity of suicidal thoughts and behaviors. The c-SSRS is only asked for youth with a presenting behavioral health concern. The assessment follows a gated structure, where items 3-6 are only asked if responses to items 1-2 are positive. For each item, we encoded both the score and a separate binary variable representing missingness (0=missing, 1=not missing) to distinguish between negative responses and unadministered items. Following completion of the c-SSRS, the nurse was asked to indicate whether the patient is “At-Risk” based on the c-SSRS score or clinical judgment. We included this *At-risk* determination as a binary variable for each c-SSRS administration. Since youth may have been asked the C-SSRS multiple times during an ED visit, we calculated the mean score across all administrations within the visit to create a single value for each item and At-Risk determination. Thus, the scale is encoded as six item scores (mean value for visit), six item missingness scores, and one At-risk determination score.

*Homicidality Screening*. All youth with a behavioral health concern were screened by nursing triage with the question, “Does the patient report homicidal ideation?” If yes is selected, the nurse was also prompted to indicate “Does the patient have a plan?”. Binary affirmed/negated responses to these two questions and variables corresponding to item missingness were included.

*Medications:* Psychotropic medications administered during the visit were categorized using Anatomical Therapeutic Chemical (ATC) classes into 8 groups: antidepressants (amitriptyline, bupropion, citalopram, clomipramine, desvenlafaxine, doxepin, duloxetine, escitalopram, fluoxetine, fluvoxamine, imipramine, mirtazapine, paroxetine, sertraline, trazodone, venlafaxine, vilazodone, vortioxetine), antiepileptics (carbamazepine, divalproex, ethosuximide, lamotrigine, levetiracetam, oxcarbazepine, phenobarbital, phenytoin, valproate, valproic), antihistamines, antipsychotics (aripiprazole, asenapine, brexpiprazole, cariprazine, chlorpromazine, chlorpromazine, fluphenazine, haloperidol, lurasidone, olanzapine, paliperidone, prochlorperazine, promethazine, quetiapine, risperidone, ziprasidone), anxiolytics (alprazolam, buspirone, chlordiazepoxide, clobazam, clonazepam, diazepam, hydroxyzine, lorazepam, temazepam, triazolam), hypnotics and sedatives (melatonin, zaleplon, zolpidem, zonisamide), lithium, and psychostimulants (amphetamine, amphetamine-dextroamphetamine, dexmethylphenidate, dextroamphetamine, guanfacine, lisdexamfetamine, methylphenidate, modafinil), and injectable medications with high probability of psychiatric indication (chlorpromazine, diazepam, haloperidol, lorazepam, olanzapine, and prochlorperazine)

*Laboratory tests* Laboratory tests related to overdose (serum acetaminophen >10 mcg/mL, salicylates >3.0 mg/dL, benzodiazepines >0 ng/L, and tricyclics >3.0 ng/mL), urine drug screen results (positive, negative), and serum alcohol (>15 mg/dL) were encoded as binary variables.

*Insurance status:* Insurance status at time of ED visit was grouped as public, private, or none/unknown.

*Prior care use:* As most children receive outpatient mental health care in the community, we limited information on prior encounters to acute care. We coded the number of ED visits, general medical hospitalizations, and psychiatric hospitalizations occurring within the past 90, 180, and 365 days within the health system.

*Age*. Age in years, at time of ED visit.

*Number of Safety Questions Asked*. The total number of c-SSRS and Homicide screening questions asked, where in max score is 6 (c-SSRS items) + 2 (homicidal ideation, plan) = 8 and minimum is 0.

*Social Vulnerability Index:* The Social Vulnerability Index (SVI) utilizes 16 variables from the U.S. Census Bureau's American Community Survey (ACS) to determine the social vulnerability of each census tract, linked to the child's home address. Separate variables encoded the overall SVI score and four subdomains: socioeconomic status, household composition and disability, minority status and language, and housing type and transportation. Each is scored on a scale of 0 to 1, with higher scores indicating greater vulnerability.

*Area Deprivation Index:* State decile and national percentile rankings were linked by census tract of the child's home address.

**Missingness:** We employed median imputation to handle missing values. Across variables, electronic health information was missing in 0-10% of cases. Insurance data was absent for 28% of individuals. We considered, but ultimately omitted, variables related to sexual orientation and gender identity due to high missingness and skew toward children receiving outpatient care within the health system.
