# Supplementary material for: Detecting Pediatric Emergency Service Use for Suicide and Self-Harm: Multimodal Analysis of 3828 Encounters
Source: JMIR Ment Health. 2026 Feb 4;13:e82371. doi: 10.2196/82371 (PMC12871580; doi:10.2196/82371)
Supplement: Multimedia Appendix 3 [file mental-v13-e82371-s003.docx]

| **Multimedia Appendix 3. Mental Health-related Chief Complaints in Structured Data Fields** |
| --- |
| Agitation |
| Altered Mental Status |
| Psychiatric Evaluation |
| Aggressive Behavior |
| Alcohol Intoxication |
| Depression |
| Agitation |
| Manic Behavior |
| Hallucinations |
| Homicidal |
| Panic Attack |
| Behavior Problem |
| Anxiety |
| Delusional |
| Eating Disorder |
| Support For Psychological Distress |
| Suicidal |
| Suicide Attempt |
| Poisoning |
| Drug Overdose |
| Ingestion |
