# Supplementary material for: Detecting Pediatric Emergency Service Use for Suicide and Self-Harm: Multimodal Analysis of 3828 Encounters
Source: JMIR Ment Health. 2026 Feb 4;13:e82371. doi: 10.2196/82371 (PMC12871580; doi:10.2196/82371)
Supplement: Multimedia Appendix 4 [file mental-v13-e82371-s004.docx]

**Multimedia Appendix 4: Missingness**

We employed median imputation to handle missing values. Across variables, electronic health information was missing in 0-10% of cases. Insurance data was absent for 28% of individuals. We considered, but ultimately omitted, variables related to sexual orientation and gender identity due to high missingness and skew toward children receiving outpatient care within the health system.
