# Supplementary material for: Detecting Pediatric Emergency Service Use for Suicide and Self-Harm: Multimodal Analysis of 3828 Encounters
Source: JMIR Ment Health. 2026 Feb 4;13:e82371. doi: 10.2196/82371 (PMC12871580; doi:10.2196/82371)
Supplement: Multimedia Appendix 5 [file mental-v13-e82371-s005.docx]

**Multimedia Appendix 5 *– Chart Annotation Guide***

**Child Suicidality EHR Phenotyping (CSEP) Project**

**Abstractor Methods Guide**

**February 24^th^, 2025**

**Version 3.0**

Manual chart review and coding for presence and type of SITB was conducted using an adapted form of the Columbia Classification Algorithm for Suicide Assessment (C-CASA). We adapted the C-CASA in the following ways: First, we recorded NSSI in a separate field to avoid obfuscating co-occurring NSSI and suicidality. Second, we combined ‘Self-injurious behavior, suicidal intent unknown’ and ‘Self-injurious behavior, no suicidal intent’ into a single category given previous report (Posner, 2007) of poor inter-rater reliability in distinguishing these (Intraclass Correlation [ICC] 0.59-0.67). Using these adaptations to the C-CASA categories, we assigned each encounter one of the following primary classifications: (1) completed suicide, (2) suicide attempt, (3) preparatory acts toward imminent suicidal behavior, (4) suicidal ideation, (5) non-suicidal self-injury, (6) other, no deliberate self-harm, (7) not enough information. We also assigned each encounter one of the following secondary classifications: (1) non-suicidal self-injury present, or (2) non-suicidal self-injury absence.

Section 1: Overview

**Overview**

- The verbatim text of the following medical record notes is extracted: progress, transfer of care, consult, nursing, social work, significant event, observation, and history and physical exam notes, assessments, diagnostic reports, legal notes and discharge summaries.
- Notes are de-identified by automated removal of Protected Health Information (PHI) using de-id. This software replaces instances of PHI with “***”.
- The goal of manual classification (also called chart abstraction) is to establish a gold-standard classification for the ED visit. These classifications are used to train a machine learning classifier as to the correct classification.
  - For all encounters: An **Encounter-level classification** assigns a single classification at the level of the encounter for the presence and type of SITB.
  - For a subset of encounters: **Concept-level classification** labels all words and phrases related to SITB, including negations, along with associated qualifiers.
- **Note:** As the objective is to develop a gold-standard classification for the ED visit, all notes that are written after a child is admitted to the hospital are not reviewed and not considered when making a classification. Abstractors are instructed to stop reading when it is apparent from the clinical documentation that the child is in transit to an inpatient psychiatric or general pediatric hospitalization.
  - Tips for coding ED to inpatient encounters: These cases are typically very long so do your best to filter out inpatient notes, look for a change in room number, and exclude specific note types such as op notes
- Abstractors are instructed to read all notes from the ED visit prior to making classifications.

Section 2: Encounter-level classification

- Trained chart abstractors review all verbatim notes and assign each emergency department (ED) visit a single classification using the Columbia Classification Algorithm of Suicide Assessment (C-CASA).
- If more than one C-CASA category is present, the abstractor will code the visit as consistent with the most severe category: suicide death>non-fatal suicide attempt>preparatory behavior>suicidal ideation>self-injurious behavior intent unknown>not enough information>self-injurious behavior, no suicidal intent.
- Classification will occur at the level of the encounter, thus hospitalizations and ED visits spanning multiple days will receive a single classification.
- Types of encounter-level classifications
- Suicide death
  - A self-injurious behavior that resulted in fatality and was associated with at least some intent to die as a result of the act.
- Suicide attempt
  - A potentially self-injurious behavior, associated with at least some intent to die, as a result of the act. Evidence that the individual intended to kill him/herself, at least to some degree, can be explicit or inferred from the behavior or circumstance. A suicide attempt may or may not result in actual injury.
- Preparatory acts
  - The individual takes steps to injure him- or herself, but is stopped by self or others from starting the self-injurious act before the potential for harm has begun.
- Suicidal ideation
  - Passive thoughts about wanting to be dead or active thoughts about killing oneself, not accompanied by preparatory behavior.
- Self-injurious behavior, no suicidal intent
  - Self-injurious behavior associated with no intent to die. The behavior is intended purely for other reasons, either to relieve distress (often referred to as “self-mutilation,” e.g., superficial cuts or scratches, hitting/banging, or burns) or to effect change in others or the environment.
- Other, no deliberate self-harm
  - No evidence of any suicidality or deliberate self-injurious behavior associated with the event. The event is characterized as an accidental injury, psychiatric or behavioral symptoms only, or medical symptoms or procedure only.
- Self-injurious behavior, intent unknown
  - Self-injurious behavior where associated intent to die is unknown and cannot be inferred. The injury or potential for injury is clear, but why the individual engaged in that behavior is unclear.
- Not enough information
  - Insufficient information to determine whether the event involved deliberate suicidal behavior or ideation. There is reason to suspect the possibility of suicidality but not enough to be confident that the event was not something other, such as an accident or psychiatric symptom. An injury sustained on a place on the body consistent with deliberate self-harm or suicidal behavior (e.g., wrists), with- out any information as to how the injury was received, would warrant placement in this category.
- **Note:** If self-injurious behavior is present *in addition to* suicidal ideation, preparatory acts, or a suicide attempt, abstractors are instructed to include this label in a secondary classification field.
  - Self-injurious behavior, intent unknown
  - Self-injurious behavior, no suicidal intent

**Table 1. Common and uncommon circumstances arising when classifying encounters, along with examples and the recommended approach.**

| **Circumstance** | **Example** | **Approach** |
| --- | --- | --- |
| Classifying case as SI vs preparatory behavior | e.g., Patient thinks about shooting himself with gun vs patient trying to obtain gun from parents’ gun case | Preparatory involves any ACTION toward self-harm, SI only includes thoughts (no action). |
| Notes include information on patient communicating suicidality in written form | e.g., Parent discovers a note where patient expresses thoughts of SI vs parent finds note where patient apologizes to family for a future SA attempt | Distinguish between writing of suicidal ideation vs. preparatory behavior in which the individual documents a specific plan to harm themselves or otherwise indicates premeditation on suicidal act.  Written forms of preparatory behavior include goodbye letters, funeral invitations, and journal entries and social media posts where a plan is expressed. |
| Cases where child is experiencing CAH/AH involving suicide or self-harming behaviors | e.g., Patient is experiencing CAH telling him that he needs to hang himself | If patient has been recently engaging in SITB code as SI.  If CAH are ego dystonic (patient expresses these thoughts do not belong to them and they do not wish to die) and patient has not been recently engaging in SITB, code as other. |
| Patient is brought to ED for a one-time statement of SI but in ED continuously denies SI | e.g., Patient is overheard saying they want to die in school but in states they were joking and denies SI | If it is a one-off statement, we can code this encounter as negative. |
| Cases where the child is brought in for concern for possible suicidality and is then discharged | e.g., Parent suspects SI because but patient has never admitted to SI and negates SI in ED | Other, no deliberate self-harm and add RULED OUT SI in notes. |
| Patient expresses SI under the influence of drugs or alcohol | e.g., Patient says they want to tie while visiting ED for alcohol intoxication | Code as SI since people under the influence since are more likely to act on thoughts impulsively. |
| Discordant reports when narrative (from humans) differs from objective signs (e.g., UDS, serum toxicology). | e.g., Patient with hx of SA denies SI and SA but is positive for benzodiazepines | Cases that are only positive for UDS but otherwise no documented suicidality would be coded as negative, however, serum toxicology results with high post-test probability (e.g., elevated serum acetaminophen level) would be positive. |
| Choosing between the secondary classifications Self-injurious behavior no suicidal intent and Self-injurious behavior intent unknown | e.g., Patient has engaged in recent cutting behaviors but note is unclear regarding patient’s intent | Choose the secondary classification that you find is most appropriate but keep in mind that they have low validity and will be combined when analyzed. |
| Classifying case as preparatory behavior vs suicide attempt | e.g. Patient goes to a cliff to die and found my treatment staff. Patient is found trying to dangle legs over balcony. | Choose preparatory when the potential for harm has not begun. |

# Section 3: Concept-level classification

Concepts are words and phrases (including negations) related to SITB. All words and phrases related to SITB are included. For example, “the patient denies suicidal ideation” (negation), “the patient states they want to die” (affirmed), “she states she cut herself to feel better” (self-injurious behavior without suicidality), and “his father attempted suicide” (refers to other) are all captured. Words and phrases in the note that are not SITB-related are not captured (e.g., “she reports low back pain”).

Abstractors are instructed to

1. Copy the verbatim word or phrase (hereafter, “SITB-related concept”) into a classification field labeled with the patient, encounter, note, and line ID.

2. Label the SITB-related concept with three qualifiers.

- - Affirmed/Negated
    - Affirmed - The concept affirms the presence of SITB (e.g., “reports suicidal ideation”)
    - Negated- The concept negates the presence of SITB (e.g., “denies suicidal ideation”)
  - Past/Present
    - Past- The concept describes SITB-related events that occurred in the past that are not directly related to the current emergency department visit
    - Present- The concept describes SITB-related events that happen that directly led to the current emergency department visit
  - Patient/Other
    - Patient – The SITB-related concept refers to the child being treated
    - Other – The SITB-related concept refers to another person (e.g., parent, sibling, peer)
  - **Note:** abstractors are encouraged to use surrounding context to assign appropriate qualifier when uncertain. For example, early in the note there may be a statement, “the patient’s mother attempted suicide when he was three years old” and later in the note “he states he is worried she will try again”. The latter statement would be captured based on the context of the former statement.

3. **Note:** Abstractors are encouraged to document in a separate note field any questions or uncertainty about the classification and qualifiers.

**Table 2. Guidelines for Concept-level Coding. Common and uncommon circumstances arising when classifying concepts, along with examples and the recommended approach, organized by qualifier type (affirmed/negated, past/present, and patient/other).**

**Affirmed or Negated**

| **Circumstance** | **Example** | **Approach** |
| --- | --- | --- |
| The SITB related word is in a sentence that describes a treatment or intervention | e.g., "Patient was made aware of suicide hotline", "parent agrees to remove sharp objects from home" | The affirmed/negated column will be left blank and will be flagged as intervention, prevention, or safety-planning related in the notes column |
| Concept contains insufficient information to affirm/negate an SITB related statement | e.g., “Patient refuses to answer a question about suicide” | For statements about ambivalence, leave affirmed/negated blank and add flag "not enough information" |
| Concept contains SITB language related to skin picking and other body focused repetitive behaviors | e.g., Patient has repetitive behaviors such as hair pulling, nail biting, picking at scabs/wounds, and etc. | Code these concepts as affirmed if INTENTIONAL to harm self.  If intentional self-harm is not documented/unknown, then label indeterminate and add note "Body focused repetitive behavior" |
| Patient verbalizes a wish or threat to harm self | e.g., “If I don’t get the candy that I want, I will cut myself with a knife” | Code this as affirmed self-injurious thought and behavior |
| Medical expert explains the baseline risk for suicide in the notes | e.g., Patient has an elevated baseline risk for suicide | For now, anything above baseline is affirmed |
| Patient describes thoughts of death in the context of grief | e.g., Reuniting with dead friend | If the concept you are capturing is vague and you are feeling it slightly related in wishing to be dead code it as affirmed |
| When a patient has an unclear intent for suicide | e.g., Patient states that she wasn't trying to kill herself, although she said she wouldn't have minded if she died after eating glass) | It is safe to say patient is aware that eating glass is self-harm, so the primary classification is “Self-injurious behavior, intent unknown” |
| Capturing concepts about a patient having dreams about self-harm/suicide | e.g., Patient recalls dreaming about swallowing bleach | Reported dreams that are remembered and said out loud it should be coded as “Affirmed” |
| For vague notes that are related to suicidality but has an unclear origin | e.g., Patient made a suicidal statement but denies meaning it seriously or negates SI | Use surrounding context to assign appropriate qualifier |
| When patients make vague statements alluding to SI | e.g., " I don’t trust myself", “I want to end it all”, “I don’t know if I’ll make it past this year” | Use context around sentence to qualify affirm or negated,  If there’s not enough info code as indeterminate and add “contextual factors” to notes |

**Past or Present**

| **Circumstance** | **Example** | **Approach** |
| --- | --- | --- |
| A concept describing when an event related to SITB may be unclear in terms of differentiating past vs present. | e.g., The patient stated she had not cut herself for a long time | For events that happen that led to presentation = label as present (otherwise past) |
| Concepts may indicate a change in clinical status | e.g., Thoughts of suicide have decreased | Label as “negated” and add “change” to the note's column |
| Medical expert explains the baseline Compared to the general population risk for suicide in the notes | e.g., Baseline chronic risk Compared to the general population the patient's baseline chronic suicide risk is estimated to be: Minimal baseline risk equivalent to general population | Code concepts related to baseline chronic risk as “present” |
| Previously attempted SA’s may be coded differently depending the timeline | e.g., Patient attempted to hang himself 10 days ago | If the SA occurred in the last 30 days label as “present” (otherwise label as past) |
| The patient received previous care that is related to the current visit | e.g., Patient with an attempt 7 days ago, seeks outpatient care, then referred to ED, is coded as an attempt; a child with an attempt 14 days ago, hospitalized, then back in the ED with ideation is coded as ideation | Past thoughts/behaviors are used to assign a primary classification if the thoughts/behaviors occurred in the past and there is no documentation of presentation to appropriate care since the thoughts/behaviors last occurred. "Appropriate care" qualifier used because frequently a child with an attempt is seen in an outpatient setting, then referred to the ED. (Did not receive prior care = present) |
| Past suicide attempts that were not treated or documented | e.g., Patient attempted suicide 2 months ago and was never received any care or treatment | Past suicide attempts (longer than 30 days) should not be considered when assigning current primary classification |

**SITB-related Concepts**

| **Circumstance** | **Example** | **Approach** |
| --- | --- | --- |
| Split sentences into concepts in order to justify qualifiers | e.g., Patient has been experiencing SI for the last few weeks but no self-injury or SA | If a concept contains an affirmation of one C-CASA category (e.g., ideation) but also negates a different category (e.g., attempt), then code as indeterminate |
| Include as much of the concept/sentence as needed to justify the qualifier | e.g., Past self-injurious behavior or suicide attempts: Aborted suicide attempt | Preference is to split the concepts if concepts make sense alone |
| When harm happens, capture statements related to intentionality | e.g., Picked a physical fight with his father, so that his father would hit him; or intentionally took 10 pills | Note suspected malingering if patient looking for an outcome that results in harm |
| The concept or statement you are capturing related to SITB may be vague but can have contextual factors in the sentence | e.g., "Pt told a counselor at school this morning what she did." | Flag this as "contextual factors" |
| Concepts can have a scenario which a peer tells a child to attempt suicide or engage in self-harm | e.g., “My friend told me if I didn’t hurt myself, he would beat me up” | Flag as "contextual factors" perceived to be directly related to suicide |
| Concepts containing statements about others' knowledge of the suicidal behavior | e.g., Prior to today, parents were not aware of SI | Flag as "miscellaneous" |
| SITB-related concepts can have thoughts about non-suicidal self-harm that do not result in self-harm | e.g., “I am having thought of cutting myself in order to relive stress” | For mixed phrases leave as indeterminate and flag as negated SI affirmed self-harm |
| Parents and providers may express additional concern for patient in a matter that presents as an opinion | e.g., “Patient seemed to be on edge and dramatic when asked about suicide” | Flag as an "opinion" in the affirmed/negated column |
| Circumstances where a family member prevents the patient from committing suicide | e.g., brother removing belt from sisters' neck who was attempting suicide | Write as affirmed since potential for harm was still there but add to notes as "intervention" when self or other force prevents an SA |
| Patient states they have passive SI but denies plan or intent | e.g., Patient thinks about attempting suicide but states they would never do it | Don’t need to distinguish "plan" or "intent" but capture as part of the concept |
| Eating disorders can be described as a form of self-harm | e.g., Patient is refusing to eat in a SA attempt | Add to notes and distinguish whether it's to lose weight to related to SI/self-harm |
| Capturing medical-related language that is linked to the statement of self-harm | e.g., Note includes documentation of “lacerations” but there is surrounding text about possible SIB | Capture clinical language but the concept must include SITB related terms that support that the injury was self-inflicted |
| Capturing concepts may include part of the providers notes where the “concern for toxidrome” | e.g., “Patient has low probability for an OD, low concern for overt toxidrome” | If patient is there for reasons unrelated to a potential intentional OD or SI then statements with “toxidrome” are not important to capture |
| Patient calling the suicide hotline | e.g., Patient does not admit to SI directly but calls the suicide hotline | Code as “affirmed” and add “intervention” to notes |
| Concepts about 5150s or prior psychiatric hospitalizations that are related SI but don’t mention SI directly | e.g., 5150s related to homicidal ideation, aggression, lack of self-care | Do not add a qualifier (affirmed/negated), but can capture and flag as intervention when there are sentences related to treatment/prevention of SI/SA |
| AVS templates related to SI | e.g., Summaries and handouts related to suicide, depression, self-injury, etc. | Do not capture or pull concepts |

**Patient or Other**

| **Circumstance** | **Example** | **Approach** |
| --- | --- | --- |
| Capture suicidal behavior by others and qualify as other | e.g., Maternal grandmother completed suicide | Include self-injurious behavior of suicide attempts by friends and family members |
| Capture all concepts related to suicide pacts | e.g., Patient made a promise to a friend that they would complete suicide if friend completes suicide first | If self-involved, code as "patient"  If only others are involved, code as "other" |

# Section 4: Common acronyms and terminology

| NSSI | Non-suicidal self-injury |
| --- | --- |
| SIB | Self-injurious behavior |
| SI | Suicidal ideation |
| c-SSRS | Columbia Suicide Severity Rating Scale |
| SH/I | Self-harm/suicidal ideation |
| r/o | Rule out |
| DTS | Danger to Self |
| DTO | Danger to Others |
| HI | Homicidal Ideation |
| 5585 | Involuntary Mental Health (72-hour) Detainment for children |
| 5150 | Involuntary Mental Health (72-hour) Detainment for adults |
| NOS | Not otherwise specified |
| IOP | Intensive outpatient program |
| AVH | Auditory and visual hallucinations |
| SA | Suicide attempt |
| h/o | History of […] |
| AMA | Discharge against medical advice |

# Section 5: Note on Involuntary Mental Health Detainment (5585) orders

Each involuntary mental health detainment (5585) includes two documents: (1) an advisement (goes to the patient) and (2) an application (goes into the record)

Information that needs to be captured as a concept is in the following five places:

*Patient advisement*
*(1) Harm yourself.* ***[check box or no check box in front of "Harm yourself"]***

*(2) We believe this is true because **** ***[capture specific text]***

*Application for detainment*
*(3) I have probable cause to believe that the person is, as a result of a mental health disorder, a danger to others or to himself/herself, or gravely disabled because: (state specific facts):*
****** [capture specific text]***
*(4) A danger to himself/hersel****f. [check box or no check box in front of "Harm yourself"]***

*(5) Historical course of the person’s mental disorder: I have considered the historical course of the person’s mental disorder:* ***[Includes evidence presented by service/support provider, family member(s), and person subject to probable cause determination or designee.]***

Section 6: Consensus Review

For the criterion standard development, annotators underwent a structured training process involving: (1) Initial training with standardized case examples, (2) Independent coding of 50 practice cases, (3) Review and reconciliation of disagreements with senior clinicians, (4) Continuous quality monitoring with periodic recalibration sessions

When staff research abstractors selected differing primary or secondary classifications for presence or type of self-injurious thoughts or behaviors (SITB), these visits were flagged for further review. These flagged visits were blindly reviewed by the two board-certified child psychiatrists and a psychiatric nurse practitioner who each separately assigned a classification. When ≥2 clinicians agreed on a classification, this was used as the final classification. When all clinicians disagreed, the three clinicians and two staff research abstractors met for four one-hour deliberation meetings to present justification for their classification and areas of uncertainty. In all cases, this discussion yielded consensus determination of a final classification.
