# Supplementary material for: Detecting Pediatric Emergency Service Use for Suicide and Self-Harm: Multimodal Analysis of 3828 Encounters
Source: JMIR Ment Health. 2026 Feb 4;13:e82371. doi: 10.2196/82371 (PMC12871580; doi:10.2196/82371)
Supplement: Multimedia Appendix 6 [file mental-v13-e82371-s006.docx]

**Multimedia Appendix 6 – Software Implementation**

## **Step 0: Convert to Pipeline Input**

This step prepares input data for the pipeline by organizing it into a standardized format.

**Purpose**: Transform source data (Excel files) into standardized CSV files for subsequent pipeline steps.

**Key operations**:

1. Reads from an Excel file (Main 12.10.24.xlsx) containing multiple sheets
2. Validates labels against a predefined schema in label_to_valid_values.json
3. Creates four output CSV files:
   - dirty_lines.csv: Raw note lines with their IDs
   - dirty_phrases.csv: SITB-related concepts with labels
   - note_metadata.csv: Mapping between notes and encounters
   - structured_data.csv: Encounter-level structured features and outcomes

**Technical Implementation Details:**

1. **File Structure Management:**
   - Creates directory structure using Path(output_folder).mkdir(parents=True, exist_ok=True) to ensure output folders exist
   - Uses relative paths for consistent execution across environments
2. **Label Validation:**
   - Loads validation schema from label_to_valid_values.json using json.loads(open('label_to_valid_values.json').read())
   - Implements strict validation with explicit error raising: raise ValueError(f'Label "{label}" has invalid value: {value}\nValid values: {valid_values}')
   - Uses dynamic label collection for flexibility: labels.append(value) for each validated label
3. **Data Type Handling:**
   - Uses isinstance(phrase, str) to verify data types before processing
   - Converts numeric IDs to consistent string format: str(int(row['HNO'])) to handle potential floating-point representations
   - Implements explicit string type conversion for label values: row[label].strip().lower() if isinstance(row[label], str) else ''
4. **Line Processing:**
   - Implements deduplication tracking with note_ids = set() to prevent duplicate entries
   - Handles extraneous quotes with conditional logic: line = line[:-1] if line[-1] == '"' else line
   - Uses CSV DictWriter for consistent output formatting
5. **Missing Value Handling:**
   - Custom function handle_nan transforms missing values: if (value == '-') or math.isnan(value): return 'NA'
   - Preserves numeric values when present: return value for non-missing data

## **Step 1: Cleaning**

This step normalizes the text data by handling common inconsistencies.

**Purpose**: Clean and standardize the note text and phrases to improve matching accuracy.

**Key operations**:

1. Processes both notes and SITB-related phrases
2. Normalizes text by removing duplicate spaces
3. Standardizes character formatting (lowercase, special symbols)
4. Concatenates individual lines into complete notes

**Technical Implementation Details:**

1. **Text Normalization Pipeline:**
   - Implements a multi-stage normalization process applied consistently to both phrases and notes
   - Order of operations is significant: spacing, character replacements, case normalization, then trimming
2. **Whitespace Normalization:**
   - Uses Python's split() with no arguments to handle multiple space types (spaces, tabs, newlines)
   - Rejoins with single spaces: ' '.join(phrase.split())
   - Applies at multiple levels (individual phrases, lines, and whole notes)
3. **Character Replacement:**
   - Targeted replacements for known data inconsistencies: phrase.replace('-@', '-') and phrase.replace('+@', '+')
   - Implementation minimizes string operations by directly replacing instead of using regex
4. **Note Construction:**
   - Notes are built incrementally using dictionary accumulation: note_id_to_text[note_id] += line
   - Avoids repeated concatenation operations with large strings
   - Final whitespace normalization performed after full note construction to handle inter-line spacing issues
5. **Case Normalization:**
   - Consistent lowercase application: phrase.strip().lower() and line.lower()
   - Applied before matching to ensure case-insensitive operations

## **Step 2 (NLP Method): Split and Annotate Sentences**

This step segments notes into sentences and labels them based on phrase presence. Clinical notes were segmented into sentences using spaCy's clinical language processing model (en_core_web_sm). Sentences containing manually abstracted SITB phrases were matched to human annotator labels according to a predefined ontology. Character-level indices tracked phrase locations to handle overlapping sentence boundaries. Each sentence maintained mappings to its source note ID and encounter ID for downstream aggregation.

**Purpose**: Divide notes into analyzable units (sentences) and annotate each with relevant SITB labels.

**Key operations**:

1. Locates each SITB phrase within the note text
2. Splits notes into sentences; testing multiple methods (NLTK, regex, spaCy)
3. Labels sentences as SITB-relevant if they contain relevant phrases

**Technical Implementation Details:**

1. **Sentence Tokenizer Implementation:**
   - Dictionary-based approach for tokenizer selection: splitter_to_sentencizer = {...}
   - NLTK implementation: nltk.tokenize.sent_tokenize(note)
   - Regex implementation: re.split(r'\.|•|\|', note) targeting specific delimiter characters
   - spaCy implementation: [str(sentence) for sentence in spacy_tokenizer(note).sents] with type conversion
2. **Phrase Location Tracking:**
   - Tracks each phrase occurrence with character-level indices: start_index = note.index(phrase, start_index + 1)
   - Handles potential multiple occurrences with iteration: for _ in range(note.count(phrase))
   - Stores triples of (start_index, end_index, labels) for efficient processing
3. **Sentence Boundary Management:**
   - Maintains sentence indices: sentence_start_and_end_index_trio.append((sentence, start_index, end_index))
   - Carefully handles sentence end calculation: end_index = start_index + len(sentence)
   - Adjusts index for next sentence: start_index = end_index + 1
4. **Overlap Detection Algorithm:**
   - Implements a precise overlap detection algorithm:

phrase_starts_in_sentence = (phrase_start_index >= sentence_start_index) and (phrase_start_index <= sentence_end_index)phrase_end_in_sentence = (phrase_end_index >= sentence_start_index) and (phrase_end_index <= sentence_end_index)has_phrase = phrase_starts_in_sentence or phrase_end_in_sentence

- - Handles phrases that span sentence boundaries

1. **Label Aggregation:**
   - Builds a comprehensive label collection per sentence: label_name_to_values[label_name].append(label)
   - Serializes as JSON for multi-label support: json.dumps(values)
   - Maps labels to sentences via dictionary: {label_name: json.dumps(values) for label_name, values in label_name_to_values.items()}
2. **Acronym Expansion:**
   - Applies acronym expansion as final processing step: expand_acronyms(sentence).strip()
   - Function imported from utilities module for code organization

## **Step 3a (NLP Method): Embed Sentences**

This step converts sentences into numerical vectors (embeddings) for machine learning. Sentences were transformed into 512-dimensional vector representations using both general-purpose (Universal Sentence Encoder) and domain-specific (MedEmbed-small-v0.1) embedding models. To optimize memory usage, processing occurred with gradient computation disabled (torch.no_grad()) and in batches of 100 sentences.

**Purpose**: Transform text sentences into vector representations that capture semantic meaning.

**Key operations**:

1. Groups sentences by encounter
2. Embeds sentences using two different models:
   - MedEmbed: A medical domain-specific embedding model
   - USE (Universal Sentence Encoder): A general-purpose embedding model
3. Caches embeddings by encounter for efficiency

**Technical Implementation Details:**

1. **Model Loading and Configuration:**
   - MedEmbed setup:

medembed_tokenizer = AutoTokenizer.from_pretrained('abhinand/MedEmbed-small-v0.1') and medembed_model = AutoModel.from_pretrained('abhinand/MedEmbed-small-v0.1')

- - USE setup:

use_preprocessor = hub.KerasLayer('https://kaggle.com/models/tensorflow/bert/TensorFlow2/en-uncased-preprocess/3') and use_embed = hub.KerasLayer('https://www.kaggle.com/models/google/universal-sentence-encoder/TensorFlow2/cmlm-en-base/1')

- - Model-specific embedding functions defined as standalone functions

1. **Memory Optimization:**
   - Explicit gradient disabling: with torch.no_grad(): for MedEmbed to reduce memory usage
   - Chunking mechanism: make_chunks(lst, n) generator for processing batches of 100 sentences
   - Comment notes memory impact: "Embedding via MedEmbed + nltk + encounter... with gradient >128GB RAM vs without gradient ~3GB"
2. **Model-Specific Embedding Functions:**
   - MedEmbed embedding function:

def embed_med_embed(lst): with torch.no_grad(): return medembed_model(**medembed_tokenizer(lst, return_tensors='pt', padding=True, truncation=True, max_length=512)).last_hidden_state.mean(dim=1)

- - USE embedding function:

lambda lst: use_embed(use_preprocessor(lst))['default']

- - Both mapped in a dictionary for consistent access:

embedder_to_embed = {...}

1. **Framework-Specific I/O Operations:**
   - PyTorch saving: torch.save(torch.cat(chunks, dim=0), bin)
   - TensorFlow saving: tf.io.write_file(bin, tf.io.serialize_tensor(tf.concat(chunks, axis=0)))
   - PyTorch loading: torch.load(bin)
   - TensorFlow loading: tf.io.parse_tensor(tf.io.read_file(bin), out_type=tf.float32)
   - Consistent interfaces maintained via dictionaries: embedder_to_load = {...} and embedder_to_save = {...}
2. **Embedding Verification and Caching:**
   - Verifies embedding dimensions match expected count: if len(sentences) != len(embedded_sentences): os.remove(encounter_bin)
   - Implements cache hit checking: if not os.path.isfile(encounter_bin):
   - Uses timestamp logging for progress monitoring: print(f'{index + 1}/{len(encounter_to_sentences)} Embedding {splitter}\'s {encounter} {datetime.datetime.now()}')

## **Step 3b (NLP Method): Train Sentence Classifier**

This step builds a classifier to identify SITB-relevant sentences based on embeddings. For each embedding type, we trained an approximate nearest neighbors (ANN) model using angular distance with a held-out set of 724 labeled encounters containing expert-annotated sentences (k neighbors = 5, 10 trees). This training subset, comprising approximately 500,000-800,000 labeled sentences, allowed optimization of the ANN before application to unlabeled data. The remaining 3,104 encounters were classified using k-normalized voting, with scores representing the proportion of sentence neighbors containing SITB content. This approach processed six distinct SITB classification features, including time context (present vs. past) and attribution (patient vs. other) dimensions.

**Purpose**: Develop a classifier that can detect SITB-related content in sentences.

**Key operations**:

1. Trains an Approximate Nearest Neighbor (ANN) model on labeled encounters
2. Uses k-nearest neighbors (k=5) for classification
3. Applies the classifier to unlabeled encounters
4. Outputs normalized vote scores for different SITB features

**Technical Implementation Details:**

1. **Approximate Nearest Neighbors Implementation:**
   - Uses the Annoy library: from annoy import AnnoyIndex
   - Configures with angular distance: ann = AnnoyIndex(dimensions, 'angular')
   - Sets 10 trees for balancing accuracy vs. performance: trees = 10
   - Builds the index with: ann.build(trees) and ann.save(ann_file)
2. **Feature Definition System:**
   - Defines features through lambda functions for flexibility:

FEATURE_TO_DEFINITION = { 'suicide_relevant': lambda row: row['suicide_relevant'] == '1', 'suicide_relevant_affirmed_preset_patient': lambda row: all(value in json.loads(row[category]) for category, value in [('Past or Present?', 'present'), ('Affirmed or Negated?', 'affirmed'), ('Patient or Other?', 'patient')]), # One feature per SITB label **{ label: lambda row: label in json.loads(row['SITB Type Label']) for label in ['attempt', 'ideation', 'non-suicidal self-injury', 'preparatory act'] },}

- - Dynamically generates lambda functions for SITB labels

1. **Parallel Processing:**
   - Implements multiprocessing: from multiprocessing import Pool
   - Uses pool.starmap for parallel execution: pool.starmap(train_ann, splitter_embedder_permutations)
   - Comment notes memory considerations: "Each ANN needs ~3GB RAM. Avoid disk swapping: Change the number of processes"
   - Runtime estimates included: "One permutation takes train_ann() ~7 hours and classify_ann() ~11 hours"
2. **Classification Algorithm:**
   - K-nearest neighbors with K=5:

ann.get_nns_by_vector(sentence['embedding'], K)

- - Vote normalization: mean of nearest neighbor values
  - Feature value calculation via the mean function:

mean([training_sentences[index]['feature'] for sentence in data['sentences'] for index in ann.get_nns_by_vector(sentence['embedding'], K)])

1. **Incremental Processing and Resumption:**
   - Tracks completion status:

num_completed = len([*csv.DictReader(outfile)])

- - Uses append mode for output files:

with open(filename, 'a', newline='', encoding='utf-8') as outfile:

- - Implements row-by-row processing to maintain state:

if num_so_far > num_completed: writer.writerow({...})

## **Step 3 (LLM Method): LLM Classification**

This step uses Large Language Models to classify encounters directly. Clinical notes were evaluated using two large language models (Llama-3.2 [3B parameters] and Llama-3.3 [70B parameters]) with structured prompts incorporating DSM-5 SITB definitions. We iteratively refined these prompts using the same held-out set of 724 annotated encounters, optimizing the prompts based on Spearman correlations with expert annotations.

Models assigned Likert scores (-3 to +3) indicating SITB likelihood. The final prompt and the remaining encounters' (n=3104) were passed to Llama-3.3-70B, to yield a note-level Likert score. Multiple notes within an encounter were aggregated by selecting the maximum score. When tested on the held-out set, the Llama-3.3 model achieved 0.89 correlation with expert-labeled outcomes, outperforming the smaller Llama-3.2 model (0.61).

**Purpose**: Leverage LLMs to analyze notes and assign SITB probabilities.

**Key operations**:

1. Prompts the LLM to evaluate each note using domain-specific criteria
2. Collects Likert scale ratings for SITB likelihood
3. Aggregates ratings across an encounter's notes

**Technical Implementation Details:**

1. **LLM Configuration:**
   - Model selection based on phase: llm_model = 'llama3.2' if TRAINING else 'llama3.3'
   - Configures context window: 'num_ctx': 8192
   - Sets repetition checking for stability: 'repeat_last_n': 512
   - Controls determinism: 'temperature': temperature
2. **Prompt Engineering:**
   - Domain-specific prompts with detailed guidelines
   - Structured task instructions with Likert scale definitions
   - Clear output format specification for consistent parsing
   - sitb_positive prompt:

| Prompt (written December 10, 2024) |
| --- |
| You are assisting a psychiatrist with a research study.  Your task is to evaluate a medical record note for signs of Self-Injurious Thoughts or Behaviors (SITB). SITB includes:  * Suicide attempt: A potentially self-injurious behavior, associated with at least some intent to die, as a result of the act. Evidence that the individual intended to kill him/ herself, at least to some degree, can be explicit or inferred from the behavior or circumstance. A suicide attempt may or may not result in actual injury.  * Preparatory acts toward imminent suicidal behavior: The individual takes steps to injure him- or herself, but is stopped by self or others from starting the self-injurious act before the potential for harm has begun.  * Suicidal ideation: Passive thoughts about wanting to be dead or active thoughts about killing oneself, not accompanied by preparatory behavior.  * Non-suicidal self-injury: Self-injurious behavior associated with no intent to die. The behavior is intended purely for other reasons, either to relieve distress (often referred to as "self-mutilation", e.g., superficial cuts or scratches, hitting/ banging, or burns) or to effect change in others or the environment.  In your evaluation, assign a score from the following Likert Scale to indicate whether the patient currently has SITB:  3: Definitely SITB  2: Very Probably SITB  1: Probably SITB  0: Possibly SITB  -1: Probably Not SITB  -2: Very Probably Not SITB  -3: Definitely Not SITB  More requirements of your evaluation:  * If the medical record note is ambiguous, try to use clues in the medical record note to assign your best guess.  * There may be additional information in the medical record note unrelated to suicide. Ignore such information. Pay attention to any words or abbreviations that may be relevant to suicide.  * Always assign a Likert Scale score.  * In the medical record note, protected health information has been replaced with: ***  Return your evaluation as a JSON object with keys:  * "likert_score": A score on the above Likert Scale  * "likert_score_analysis": A brief explanation of how you decided on the "likert_score"  Evaluate the following medical record note of an emergency department visit:  ------------  {note} |
| Definitions adapted from: *Posner, K., Oquendo, M. A., Gould, M., Stanley, B., & Davies, M. (2007). Columbia Classification Algorithm of Suicide Assessment (C-CASA): classification of suicidal events in the FDA’s pediatric suicidal risk analysis of antidepressants. American journal of psychiatry, 164(7), 1035-1043.* |

1. **Response Validation Architecture:**
   - Uses Pydantic for schema definition and validation:

class Response(BaseModel): likert_score: Annotated[int, Field(ge=-3, le=3)] likert_score_analysis: str

- - Implements temperature-based retry logic for handling validation failures:

for value in range(11): temperature = value / 10 # ... LLM call ... try: response = Response.model_validate_json(content) break except ValidationError: # ... error handling ...

1. **Output Model:**
   - Uses CSV writer for structured output: note_writer.writerow({...})
   - Records detailed response metadata:
     - 'encounter_id': Encounter identifier
     - 'note_id': Note identifier
     - 'likert_score': Model's numerical assessment
     - 'likert_score_analysis': Model's reasoning
     - 'note': Original text
     - 'temperature': Temperature used for generation
2. **Encounter-Level Aggregation:**
   - Tracks scores across an encounter: scores = [] and scores.append(score)
   - Uses max score as encounter value:encounter_writer.writerow({'encounter_id': encounter_id, 'max_likert_score': max(scores)})
   - Prioritizes strongest signal of SITB risk

## **Step 4: Classifier Development and Evaluation**

Models were developed using 10-fold cross-validation with nested hyperparameter optimization across a grid of parameters:

- max_depth: [3, 5, 10, None]
- min_samples_split: [2, 5, 10]
- max_features: ['sqrt', 'log2', None]
- n_estimators: [100, 200, 300]

Hyperparameter tuning occurred only within training folds of each cross-validation iteration using a nested grid search approach with 3-fold inner cross-validation. The best-performing parameter combination from the inner loop was then applied to the full training fold before evaluation on the test fold.

Missing data were handled via median imputation using scikit-learn's SimpleImputer. For categorical variables, missing values were treated as a separate category. For the c-SSRS, which had a higher rate of missingness, we used additional indicator variables to capture the pattern of missingness, as this could be clinically meaningful (e.g., gatekeeping of screener items; inability to complete assessment due to acute distress).

**Purpose**: Create and evaluate machine learning models that predict SITB outcomes.

**Key operations**:

1. Combines NLP-derived features with structured clinical data
2. Trains classification models using cross-validation
3. Performs hyperparameter optimization
4. Evaluates model performance with various metrics and visualizations

**Technical Implementation Details:**

1. **Cross-Validation Implementation:**
   - Uses scikit-learn's KFold:

KFold(n_splits=cv_fold, shuffle=True, random_state=command_args['random_seed'])

- - Implements nested cross-validation for hyperparameter tuning:

half_train_length = len(train_indices) // 2train_hyperparameters_indices = train_indices[:half_train_length]test_hyperparameters_indices = train_indices[half_train_length:]

- - Default to 10-fold cross-validation: --cv_fold', default = 10

1. **Hyperparameter Grid Search:**
   - Comprehensive parameter space exploration:

forest_grid = [{ 'max_depth': [3, 5, 10, None], 'n_estimators': [100, 200, 300], 'min_samples_split': [2, 5, 10], 'max_features': ['sqrt', 'log2', None], 'min_samples_leaf': [1, 2, 3],}]

- - Combinatorial testing with itertools:

permutations = [item for item in itertools.product(*values)]

- - Optimal parameter selection based on accuracy:

if (highest_accuracy is None) or (accuracy > highest_accuracy): best_hyperparameters = hyperparameters

1. **Missing Data Handling:**
   - Median imputation:

imputer = SimpleImputer(strategy='median')

- - Application to feature matrix:

predictors = imputer.fit_transform(predictors)

- - NaN detection with isnan: if isnan(p): return [np.nan] * 6

1. **Evaluation Metrics Calculation:**
   - Confusion matrix derivation:

confusion_matrix(result['y_true'], result['y_pred'], labels=result['classes'])

- - ROC curve computation:

false_positive_rate, true_positive_rate, _ = roc_curve(y_true, y_proba)

- - AUC calculation:

auc(false_positive_rate, true_positive_rate)

- - Various metric implementations: sensitivity, specificity, PPV, NPV, accuracy, F-statistic

1. **Feature Importance Analysis:**
   - SHAP implementation:

shap_values = TreeExplainer(classifier).shap_values(predictors_test)[:,:,1]

- - Permutation importance:

permutation_importance(classifier, predictors_test, outcomes_test, n_repeats=30, random_state=command_args['random_seed'])

- - Visualizations via matplotlib:

summary_plot(shap_values, predictors_reordered, [*predictors_labeled[0].keys()], 20, show=False)

1. **Model Configuration:**
   - Random Forest setup:

RandomForestClassifier( class_weight=command_args['class_weight'], max_depth=command_args['max_depth'], max_features=command_args['max_features'], min_samples_leaf=command_args['min_samples_leaf'], min_samples_split=command_args['min_samples_split'], n_estimators=command_args['n_estimators'], oob_score=True, random_state=command_args['random_seed'])

- - Logistic Regression setup:

LogisticRegression( l1_ratio=l1_ratio, max_iter=max_iter, penalty=penalty, solver=solver, C=C)

## **Step 5: Classifier Performance Comparisons**

This step analyzes classifier performance across different patient subgroups.

**Purpose**: Assess model fairness and performance across demographic groups and diagnostic categories.

**Key operations**:

1. Stratifies results by age ranges, sex, race/ethnicity, and diagnosis
2. Computes performance metrics for each subgroup
3. Generates confidence intervals for key metrics
4. Creates comparative visualizations

**Technical Implementation Details:**

1. **Subgroup Definition System:**
   - Age ranges:

AGE_RANGES = [(6, 17), (6, 12), (13, 17)]

- - Sex groupings:

SEX_SUBGROUPS = [('Females', [('Female', 1)]), ('Males', [('Female', 0)])]

- - Race/ethnicity groupings: Complex nested structure for various racial/ethnic categories
  - Diagnostic groupings:

[('bipolar_and_related_dos', [('bipolar_and_related_dos', 1)]), ...]

1. **Statistical Confidence Interval Implementation:**
   - Beta distribution approach for binomial CIs:

def binomial_ci(x, n, alpha=0.05): c1 = 0 if x == 0 else beta.interval(1 - alpha, x, n - x + 1)[0] c2 = 1 if x == n else beta.interval(1 - alpha, x + 1, n - x)[1] return c1, c2

- - Confidence interval calculation for each metric:

def make_confusion_matrix_ci(name, numerator, denominator, cm_dict): lower, upper = binomial_ci(sum(cm_dict[item] for item in numerator), sum(cm_dict[item] for item in denominator)) return {f'{name} LL 95% CI': lower, f'{name} UL 95% CI': upper}

1. **Subgroup Filtering Algorithm:**
   - Filters encounters based on age and group criteria:

subgroup_encounters = [ encounter for encounter in encounters if (age_upper >= float(encounter['data']['encounter_age']) >= age_lower) and any(float(encounter['data'][key]) == value for key, value in subgroup_key_and_value_pairs)]

- - Extracts relevant properties with list comprehensions

1. **Visualization for Subgroup Comparison:**
   - Generates ROC curves for each subgroup:

false_positive_rate, true_positive_rate, _ = roc_curve(expected, actual_probability)roc_auc = auc(false_positive_rate, true_positive_rate)plt.plot(false_positive_rate, true_positive_rate, alpha=1.0, lw=2 if subgroup_name == EVERYONE[0] else 1, label='%s: AUC = %0.2f' % (subgroup_name, roc_auc))

- - Outputs PDF visualizations:

f.savefig(os.path.join(permutation_folder, f'{input_filename[:-4]}_ages{age_lower}-{age_upper}_{subgroups_name}.pdf'), bbox_inches='tight')

1. **Multi-class Handling:**
   - Special handling for multi-class scenarios:

if len(classes) <= 2: ... else: ...

- - Label binarization for multi-class metrics:

onehot = LabelBinarizer().fit(actual).transform(expected)[:, class_index]

- - Class-specific probability extraction:

proba = [ap[class_index] for ap in actual_probability]
