# Supplementary material for: Detecting Pediatric Emergency Service Use for Suicide and Self-Harm: Multimodal Analysis of 3828 Encounters
Source: JMIR Ment Health. 2026 Feb 4;13:e82371. doi: 10.2196/82371 (PMC12871580; doi:10.2196/82371)
Supplement: Multimedia Appendix 7 [file mental-v13-e82371-s007.docx]

**Multimedia Appendix 7 - Community Advisory Board**

The project Community Advisory Board comprised six members, including parents of youth with mental health conditions (n=2), young adults with lived experience of suicidality (n=2), and mental health advocates (n=2). Board members were recruited through existing research partnerships and community organizations, with attention to diverse perspectives and backgrounds.

The Board met quarterly during the prompt development period (June-December 2024) to review preliminary LLM outputs and provide feedback on:

1. Clinical relevance of identified SITB patterns
2. Potential linguistic biases in the prompt or model responses
3. Edge cases and potential misclassifications
4. Ethical considerations

Board members reviewed anonymized examples of model outputs alongside the synthetic clinical text to evaluate concordance with clinical judgment. Their feedback informed iterations of prompt refinement, with particular attention to reducing false positives in ambiguous clinical scenarios and ensuring cultural sensitivity in the detection process.
