# Supplementary material for: Detecting Pediatric Emergency Service Use for Suicide and Self-Harm: Multimodal Analysis of 3828 Encounters
Source: JMIR Ment Health. 2026 Feb 4;13:e82371. doi: 10.2196/82371 (PMC12871580; doi:10.2196/82371)
Supplement: Multimedia Appendix 8 [file mental-v13-e82371-s008.docx]

## **Multimedia Appendix 8. Variables Comprising Feature Sets**

## Base Classifier

| **Features** | **Description** |
| --- | --- |
| CC_SI | Chief Complaint of SITB |
| suicide_or_self_injury | ICD-10-CM code for suicide or self-injury |

## c-SSRS + ICD/CC Classifier

| **Features** | **Description** |
| --- | --- |
| CC_SI | Chief Complaint of Suicidal Ideation |
| suicide_or_self_injury | ICD-10-CM code for suicide or self-injury |
| cssrs_item1_new | Columbia Suicide Severity Rating Scale item 1 (wish to be dead) |
| cssrs_item2_new | Columbia Suicide Severity Rating Scale item 2 (suicidal thoughts) |
| cssrs_item3_new | Columbia Suicide Severity Rating Scale item 3 (suicidal thoughts with method) |
| cssrs_item4_new | Columbia Suicide Severity Rating Scale item 4 (suicidal intent) |
| cssrs_item5_new | Columbia Suicide Severity Rating Scale item 5 (suicidal intent with plan) |
| cssrs_item6_new | Columbia Suicide Severity Rating Scale item 6 (suicide behavior) |
| at_risk | Patient identified as being at risk based on C-SSRS |
| missing_cssrs_item1_new | Missing data for C-SSRS item 1 |
| missing_cssrs_item2_new | Missing data for C-SSRS item 2 |
| missing_cssrs_item3_new | Missing data for C-SSRS item 3 |
| missing_cssrs_item4_new | Missing data for C-SSRS item 4 |
| missing_cssrs_item5_new | Missing data for C-SSRS item 5 |
| missing_cssrs_item6_new | Missing data for C-SSRS item 6 |
| missing_at_risk | Indicator for missing risk assessment |

## Mental Health Diagnosis + ICD/CC Classifier

| **Features** | **Description** |
| --- | --- |
| CC_SI | Chief Complaint of Suicidal Ideation |
| suicide_or_self_injury | ICD-10-CM code for suicide or self-injury |
| acc_undeter_poisoning | Accidental or undetermined poisoning |
| adhd | Attention Deficit Hyperactivity Disorder |
| anxiety_dos | Anxiety disorders |
| autism_spectrum_disorder | Autism Spectrum Disorder |
| bipolar_and_related_dos | Bipolar and related disorders |
| communication_dos | Communication disorders |
| depressive_dos | Depressive disorders |
| developmental_delay_or_unspec | Developmental delay or unspecified |
| disruptive_impulse_conduct | Disruptive, impulse-control, and conduct disorders |
| dissociative_dos | Dissociative disorders |
| elimination_dos | Elimination disorders |
| feeding_and_eating_dos | Feeding and eating disorders |
| fetal_damage_maternal_sud | Fetal damage from maternal substance use disorder |
| intellectual_disability | Intellectual disability |
| maternal_mh_or_sud_preg | Maternal mental health or substance use disorder during pregnancy |
| mental_health_symptom | Mental health symptom |
| miscellaneous | Miscellaneous mental health diagnoses |
| motor_dos | Motor disorders |
| neurocognitive_dos | Neurocognitive disorders |
| ocd_and_related_dos | Obsessive-Compulsive and related disorders |
| personality_dos | Personality disorders |
| schizophrenia_psychotic_dos | Schizophrenia and other psychotic disorders |
| sexuality_and_gender_identity_dos | Sexuality and gender identity disorders |
| sleep_wake_dos | Sleep-wake disorders |
| somatic_symptom_and_related_dos | Somatic symptom and related disorders |
| specific_learning_dos | Specific learning disorders |
| substance_abuse_related_medical_illness | Medical illness related to substance abuse |
| substance_related_and_addictive_dos | Substance-related and addictive disorders |
| trauma_and_stressor_related_dos | Trauma and stressor-related disorders |
| psych_comorbidity | Psychiatric comorbidity (presence of multiple psychiatric conditions) |

## aCS Classifier

| **Feature** | **Description** |
| --- | --- |
| encounter_year | Year of the healthcare encounter |
| site | Healthcare facility location |
| Admission | Patient admitted to hospital |
| Observation | Patient placed under observation status |
| Discharge | Patient discharged from facility |
| Transferred | Patient transferred to another facility |
| AMA_or_Eloped | Left against medical advice or eloped (left without notice) |
| LWBS | Left without being seen |
| Deceased | Patient died |
| Sent_to_LD | Sent to labor and delivery |
| Other_ED_Dispo | Other emergency department disposition |
| any_psych_hospitalization | Any psychiatric hospitalization |
| psych_at_xxxx | Psychiatric care within health system |
| psych_outside_xxxx | Psychiatric care outside health system |
| psych_unknown_where | Psychiatric care at unknown location |
| Med_Admission | Medical admission |
| CC_SI | Chief Complaint of Suicidal Ideation |
| CC_Psych | Chief Complaint related to psychiatric issues |
| CC_Missing | Missing chief complaint |
| arrived_5585 | Patient arrived on California 5585 involuntary psychiatric hold |
| put_on_5585 | Patient placed on California 5585 involuntary psychiatric hold |
| any_5585 | Any California 5585 involuntary psychiatric hold |
| acc_undeter_poisoning | ICD-10-CM code for Accidental or undetermined poisoning |
| adhd | ICD-10-CM code for Attention Deficit Hyperactivity Disorder |
| anxiety_dos | ICD-10-CM code for Anxiety disorders |
| autism_spectrum_disorder | ICD-10-CM code for Autism Spectrum Disorder |
| bipolar_and_related_dos | ICD-10-CM code for Bipolar and related disorders |
| communication_dos | ICD-10-CM code for Communication disorders |
| depressive_dos | ICD-10-CM code for Depressive disorders |
| developmental_delay_or_unspec | ICD-10-CM code for Developmental delay or unspecified |
| disruptive_impulse_conduct | ICD-10-CM code for Disruptive, impulse-control, and conduct disorders |
| dissociative_dos | ICD-10-CM code for Dissociative disorders |
| elimination_dos | ICD-10-CM code for Elimination disorders |
| feeding_and_eating_dos | ICD-10-CM code for Feeding and eating disorders |
| fetal_damage_maternal_sud | ICD-10-CM code for Fetal damage from maternal substance use disorder |
| intellectual_disability | ICD-10-CM code for Intellectual disability |
| maternal_mh_or_sud_preg | ICD-10-CM code for Maternal mental health or substance use disorder during pregnancy |
| mental_health_symptom | ICD-10-CM code for Mental health symptom |
| miscellaneous | ICD-10-CM code for Miscellaneous mental health diagnoses |
| motor_dos | ICD-10-CM code for Motor disorders |
| neurocognitive_dos | ICD-10-CM code for Neurocognitive disorders |
| ocd_and_related_dos | ICD-10-CM code for Obsessive-Compulsive and related disorders |
| personality_dos | ICD-10-CM code for Personality disorders |
| schizophrenia_psychotic_dos | ICD-10-CM code for Schizophrenia and other psychotic disorders |
| sexuality_and_gender_identity_dos | ICD-10-CM code for Sexuality and gender identity disorders |
| sleep_wake_dos | ICD-10-CM code for Sleep-wake disorders |
| somatic_symptom_and_related_dos | ICD-10-CM code for Somatic symptom and related disorders |
| specific_learning_dos | ICD-10-CM code for Specific learning disorders |
| substance_abuse_related_medical_illness | ICD-10-CM code for Medical illness related to substance abuse |
| substance_related_and_addictive_dos | ICD-10-CM code for Substance-related and addictive disorders |
| suicide_or_self_injury | ICD-10-CM code for suicide or self-injury |
| trauma_and_stressor_related_dos | ICD-10-CM code for Trauma and stressor-related disorders |
| psych_comorbidity | Psychiatric comorbidity (≥ 2 CAMDHS categories) |
|  |  |
| Female | Patient identified as female |
| Male | Patient identified as male |
| Asian_Not_Hispanic | Asian, not Hispanic ethnicity |
| White_Not_Hispanic | White, not Hispanic ethnicity |
| Multiple_races_not_Hispanic | Multiple races, not Hispanic ethnicity |
| Black_Not_Hispanic | Black, not Hispanic ethnicity |
| American_Indian_Not_Hispanic | American Indian, not Hispanic ethnicity |
| NA+Other_Not_Hispanic | Not available or other, not Hispanic ethnicity |
| Native_Hawaiian_Not_Hispanic | Native Hawaiian or Pacific Islander, not Hispanic ethnicity |
| Hispanic_or_Latino | Hispanic or Latino ethnicity |
| Spanish | Spanish language preference |
| encounter_age | Age at the time of healthcare encounter |
| Missing_prior_Use | Missing information on prior healthcare utilization |
| prior_90d_ed | Emergency department visit within previous 90 days |
| prior_180d_ed | Emergency department visit within previous 180 days |
| prior_365d_ed | Emergency department visit within previous 365 days |
| prior_90d_hosp | Hospitalization within previous 90 days |
| prior_180d_hosp | Hospitalization within previous 180 days |
| prior_365d_hosp | Hospitalization within previous 365 days |
| prior_90d_psych | Psychiatric hospitalization within previous 90 days |
| prior_180d_psych | Psychiatric hospitalization within previous 180 days |
| prior_365d_psych | Psychiatric hospitalization within previous 365 days |
|  |  |
|  |  |
| cssrs_primarycomplaint | Primary complaint on Columbia Suicide Severity Rating Scale |
| homicide_screening | Screening for homicidal ideation |
| homicide_plan | Assessment for homicide plan |
| cssrs_item1_new | Columbia Suicide Severity Rating Scale item 1 (wish to be dead) |
| cssrs_item2_new | Columbia Suicide Severity Rating Scale item 2 (suicidal thoughts) |
| cssrs_item3_new | Columbia Suicide Severity Rating Scale item 3 (suicidal thoughts with method) |
| cssrs_item4_new | Columbia Suicide Severity Rating Scale item 4 (suicidal intent) |
| cssrs_item5_new | Columbia Suicide Severity Rating Scale item 5 (suicidal intent with plan) |
| cssrs_item6_new | Columbia Suicide Severity Rating Scale item 6 (suicide behavior) |
| at_risk | Patient identified as being at risk based on assessment |
| security_bedside | Security stationed at patient's bedside |
| detainment_initiated | Legal detainment process initiated |
| charge_nurse_notified | Charge nurse notified of patient status |
| md_notified | Physician notified of patient status |
| primary_nurse_notified | Primary nurse notified of patient status |
| missing_cssrs_primarycomplaint | Missing data for C-SSRS primary complaint |
| missing_homicide_screening | Missing data for homicide screening |
| missing_homicide_plan | Missing data for homicide plan |
| missing_cssrs_item1_new | Missing data for C-SSRS item 1 |
| missing_cssrs_item2_new | Missing data for C-SSRS item 2 |
| missing_cssrs_item3_new | Missing data for C-SSRS item 3 |
| missing_cssrs_item4_new | Missing data for C-SSRS item 4 |
| missing_cssrs_item5_new | Missing data for C-SSRS item 5 |
| missing_cssrs_item6_new | Missing data for C-SSRS item 6 |
| missing_at_risk | Indicator for missing risk assessment |
| num_safety_questions | Number of safety assessment questions completed |
|  |  |
| antidepressant | Patient on antidepressant medication |
| Antiepileptics | Patient on antiepileptic medication |
| Antihistamines | Patient on antihistamine medication |
| Antipsychotics | Patient on antipsychotic medication |
| Anxiolytics | Patient on anxiolytic medication |
| Hypnotics_and_Sedatives | Patient on hypnotic or sedative medication |
| Lithium | Patient on lithium |
| Psychostimulants | Patient on psychostimulant medication |
| Injectables | Patient on injectable psychiatric medications |
| acetaminophen | Acetaminophen detected in toxicology |
| salicylates | Salicylates detected in toxicology |
| benzodiazepines | Benzodiazepines detected in toxicology |
| tricyclics | Tricyclic antidepressants detected in toxicology |
| alcohol | Alcohol detected in toxicology |
| uds_amphetamine_meth | Amphetamine/methamphetamine detected in urine drug screen |
| uds_barbiturates | Barbiturates detected in urine drug screen |
| uds_benzodiazepines | Benzodiazepines detected in urine drug screen |
| uds_cannabinoids | Cannabinoids detected in urine drug screen |
| uds_cocaine | Cocaine detected in urine drug screen |
| uds_methadone | Methadone detected in urine drug screen |
| uds_opiates | Opiates detected in urine drug screen |
| uds_ethanol | Ethanol detected in urine drug screen |
| uds_oxycodone | Oxycodone detected in urine drug screen |
|  |  |
| provider_female | Provider identified as female |
| provider_male | Provider identified as male |
| Insurance_Private | Patient has private insurance |
| Insurance_Public | Patient has public insurance |
| Insurance_NA_Other | Patient insurance status not available or other type |
| adi_natrank | Area Deprivation Index national ranking |
| adi_staternk | Area Deprivation Index state ranking |
| education | Education level |
| income | Income level |
| svi_socio_econ | Social Vulnerability Index - socioeconomic status |
| svi_hcomp | Social Vulnerability Index - household composition |
| svi_mino_lang | Social Vulnerability Index - minority status and language |
| svi_htype_trans | Social Vulnerability Index - housing type and transportation |
| svi_total | Social Vulnerability Index - total score |

## NLP-gen Classifier

| **Features** | **Description** |
| --- | --- |
| unstructured_attempt | Suicide attempt mentioned in clinical notes |
| unstructured_ideation | Suicidal ideation mentioned in clinical notes |
| unstructured_non-suicidal self-injury | Non-suicidal self-injury mentioned in clinical notes |
| unstructured_preparatory act | Preparation for suicide mentioned in clinical notes |
| unstructured_suicide_relevant | Suicide-relevant information in clinical notes |
| unstructured_suicide_relevant_affirmed_present_patient | Suicide-relevant information affirmed by patient in preset forms |

## NLP-med Classifier

| **Features** | **Description** |
| --- | --- |
| unstructured_attempt | Suicide attempt mentioned in clinical notes (medical model) |
| unstructured_ideation | Suicidal ideation mentioned in clinical notes (medical model) |
| unstructured_non-suicidal self-injury | Non-suicidal self-injury mentioned in clinical notes (medical model) |
| unstructured_preparatory act | Preparation for suicide mentioned in clinical notes (medical model) |
| unstructured_suicide_relevant | Suicide-relevant information in clinical notes (medical model) |
| unstructured_suicide_relevant_affirmed_preset_patient | Suicide-relevant information affirmed by patient in preset forms (medical model) |

## LLM Classifier

| **Features** | **Description** |
| --- | --- |
| unstructured_sitb_positive | Self-Injurious Thoughts and Behaviors identified as positive through large language model analysis of clinical notes |

## Combined Classifiers

| Classifier | **Features** | **Description** |
| --- | --- | --- |
| **aCS + NLP-gen** | All aCS features plus NLP-gen features | Combines all features from the aCS classifier with the NLP-gen features |
| **aCS + NLP-med** | All aCS features plus NLP-med features | Combines all features from the aCS classifier with the NLP-med features |
| **aCS + LLM** | All aCS features plus LLM features | Combines all features from the aCS classifier with the LLM feature |
