# Supplementary material for: Detecting Pediatric Emergency Service Use for Suicide and Self-Harm: Multimodal Analysis of 3828 Encounters
Source: JMIR Ment Health. 2026 Feb 4;13:e82371. doi: 10.2196/82371 (PMC12871580; doi:10.2196/82371)
Supplement: Multimedia Appendix 9 [file mental-v13-e82371-s009.docx]

**Multimedia Appendix 9 - Detection Performance by Classifier**

| **Detection Performance by Classifier** | | | | | | | | | | | | | |
| --- | --- | --- | --- | --- | --- | --- | --- | --- | --- | --- | --- | --- | --- |
| **Classifier** | **Age range** | **N** | **TN** | **FP** | **FN** | **TP** | **Sensitivity 95%CI** | **Specificity**  **95%CI** | **Accuracy**  **95%CI** | **AUROC**  **95%CI** |  | **PPV**  **95%CI** | **NPV**  **95%CI** |
| *ICD/CC (Base)* | 6 to 17 | 3102 | 497 | 1233 | 106 | 1266 | 0.923 (0.907 - 0.936) | 0.287 (0.266 - 0.309) | 0.568 (0.551 - 0.586) | 0.865 (0.852 - 0.879) |  | 0.824 (0.791 - 0.854) | 0.551 (0.586 - 0.865) |
| *c-SSRS + ICD/CC* | 6 to 17 | 3102 | 1507 | 223 | 131 | 1241 | 0.905 (0.888 - 0.920) | 0.871 (0.854 - 0.887) | 0.886 (0.874 - 0.897) | 0.935 (0.925 - 0.944) |  | 0.920 (0.906 - 0.933) | 0.874 (0.897 - 0.935) |
| *MH dx + ICD/CC* | 6 to 17 | 3102 | 1208 | 522 | 78 | 1294 | 0.943 (0.930 - 0.955) | 0.698 (0.676 - 0.720) | 0.807 (0.792 - 0.820) | 0.934 (0.924 - 0.943) |  | 0.939 (0.925 - 0.952) | 0.792 (0.820 - 0.934) |
| *aCS* | 6 to 17 | 3102 | 1328 | 402 | 26 | 1346 | 0.981 (0.972 - 0.988) | 0.768 (0.747 - 0.787) | 0.862 (0.849 - 0.874) | 0.965 (0.958 - 0.972) |  | 0.981 (0.972 - 0.987) | 0.849 (0.874 - 0.965) |
| *NLP-gen* | 6 to 17 | 3102 | 1341 | 389 | 27 | 1345 | 0.980 (0.971 - 0.987) | 0.775 (0.755 - 0.795) | 0.866 (0.853 - 0.878) | 0.956 (0.948 - 0.964) |  | 0.980 (0.971 - 0.987) | 0.853 (0.878 - 0.956) |
| *NLP-med* | 6 to 17 | 3102 | 1399 | 331 | 37 | 1335 | 0.973 (0.963 - 0.981) | 0.809 (0.789 - 0.827) | 0.881 (0.869 - 0.893) | 0.970 (0.964 - 0.977) |  | 0.974 (0.965 - 0.982) | 0.869 (0.893 - 0.970) |
| *LLM* | 6 to 17 | 3102 | 1328 | 402 | 26 | 1346 | 0.979 (0.970 - 0.986) | 0.855 (0.838 - 0.872) | 0.910 (0.899 - 0.920) | 0.962 (0.955 - 0.969) |  | 0.843 (0.824 - 0.861) | 0.981 (0.973 - 0.987) |
| *aCS + NLP-gen* | 6 to 17 | 3102 | 1361 | 369 | 20 | 1352 | 0.985 (0.978 - 0.991) | 0.787 (0.767 - 0.806) | 0.875 (0.862 - 0.886) | 0.973 (0.966 - 0.979) |  | 0.986 (0.978 - 0.991) | 0.862 (0.886 - 0.973) |
| *aCS + NLP-med* | 6 to 17 | 3102 | 1392 | 338 | 26 | 1346 | 0.981 (0.972 - 0.988) | 0.805 (0.785 - 0.823) | 0.883 (0.871 - 0.894) | 0.972 (0.965 - 0.978) |  | 0.982 (0.973 - 0.988) | 0.871 (0.894 - 0.972) |
| *aCS + LLM* | 6 to 17 | 3102 | 1479 | 251 | 33 | 1339 | 0.976 (0.966 - 0.983) | 0.855 (0.837 - 0.871) | 0.908 (0.898 - 0.918) | 0.977 (0.971 - 0.982) |  | 0.978 (0.969 - 0.985) | 0.898 (0.918 - 0.977) |

*Abbreviations: 95% CI: 95% Confidence Interval. Cross-validation variability was used to construct asymptotically exact confidence intervals (CIs) for test error. TN: True Negative; FP: False Positive; FN: False Negative; TP: True Positive; AUROC: Area Under the Receiver Operating Characteristic curve; PPV: Positive Predictive Value; NPV: Negative Predictive Value.*

*The feature set notation is as follows: ICD/CC refers to a feature set based on International Classification of Diseases, Clinical Modification, Version 10, codes for non-fatal suicide attempt and intentional self-harm, as defined by the Centers for Disease Control and Prevention Case Surveillance definition list, plus suicide-related chief concern; c-SSRS+ICD/CC combines ICD/CC with c-SSRS item scores; MH dx+ICD/CC combines ICD/CC with Child and Adolescent Mental Health Disorders Classification System ICD-10-CM code categories; aCS represents all available structured data; NLP-gen and NLP-med are feature sets based on vectorized text features with embeddings derived from the Universal Sentence Encoder and MedEmbed, respectively; LLM refers to Likert-type scores generated by the open-source language model llama-3.3-70B. Feature sets denoted by (aCS+) indicate combinations of aCS with the corresponding text-based feature set (NLP-gen, NLP-med, or LLM*
