# Supplementary material for: Detecting Pediatric Emergency Service Use for Suicide and Self-Harm: Multimodal Analysis of 3828 Encounters
Source: JMIR Ment Health. 2026 Feb 4;13:e82371. doi: 10.2196/82371 (PMC12871580; doi:10.2196/82371)
Supplement: Multimedia Appendix 10 [file mental-v13-e82371-s010.docx]

**Multimedia Appendix 10 - DeLong Tests (p-value) by Feature Set**

| **DeLong Tests (p-value) by Feature Set** | | | | | | | | | | | |
| --- | --- | --- | --- | --- | --- | --- | --- | --- | --- | --- | --- |
|  |  | **Structured Data** | | | | **Text Data** | | | **Combination** | | |
|  | Feature Set | *ICD/CC (base)* | *c-SSRS + ICD/CC* | *MH dx + ICD/CC* | *aCS* | *NLP-gen* | *NLP-med* | *LLM* | *aCS + NLP-gen* | *aCS + NLP-med* | *aCS + LLM_p* |
| **Structured Data** | *ICD/CC (base)* | - |  |  |  |  |  |  |  |  |  |
|  | *c-SSRS + ICD/CC* | 3.6154E-28 | - |  |  |  |  |  |  |  |  |
|  | *MH dx + ICD/CC* | 5.7849E-25 | 0.8349 | - |  |  |  |  |  |  |  |
|  | *aCS* | 2.2798E-46 | 2.854E-15 | 3.4006E-16 | - |  |  |  |  |  |  |
| **Text Data** | *NLP-gen* | 3.5284E-33 | 4.8593E-05 | 1.4848E-06 | 0.0049 | - |  |  |  |  |  |
|  | *NLP-med* | 5.0972E-47 | 6.5816E-13 | 9.8951E-18 | 0.0763 | 1.434E-07 | - |  |  |  |  |
|  | *LLM* | 3.1943E-37 | 1.6168E-07 | 3.5831E-10 | 0.3694 | 0.1051 | 0.0194 | - |  |  |  |
| **Combination** | *aCS + NLP* | 7.6601E-53 | 8.7622E-19 | 2.2505E-24 | 1.19592E-07 | 7.4928E-10 | 0.2850 | 0.0010 | - |  |  |
|  | *aCS + NLP-med* | 4.2621E-50 | 5.7212E-15 | 9.1318E-21 | 0.0062 | 9.8363E-09 | 0.3352 | 0.0031 | 0.6336 | - |  |
|  | *aCS + LLM* | 1.6873E-55 | 3.0966E-19 | 5.056E-28 | 5.70206E-07 | 1.0698E-10 | 0.0113 | 1.5023E-10 | 0.04165 | 0.0272 | - |

*Shaded = p < 0.05; White = p > 0.05*

*The feature set notation is as follows: ICD/CC refers to a feature set based on International Classification of Diseases, Clinical Modification, Version 10, codes for non-fatal suicide attempt and intentional self-harm, as defined by the Centers for Disease Control and Prevention Case Surveillance definition list, plus suicide-related chief concern; c-SSRS+ICD/CC combines ICD/CC with c-SSRS item scores; MH dx+ICD/CC combines ICD/CC with Child and Adolescent Mental Health Disorders Classification System ICD-10-CM code categories; aCS represents all available structured data; NLP-gen and NLP-med are feature sets based on vectorized text features with embeddings derived from the Universal Sentence Encoder and MedEmbed, respectively; LLM refers to Likert-type scores generated by the open-source language model llama-3.3-70B. Feature sets denoted by (aCS+) indicate combinations of aCS with the corresponding text-based feature set (NLP-gen, NLP-med, or LLM).*
