# Supplementary material for: Detecting Pediatric Emergency Service Use for Suicide and Self-Harm: Multimodal Analysis of 3828 Encounters
Source: JMIR Ment Health. 2026 Feb 4;13:e82371. doi: 10.2196/82371 (PMC12871580; doi:10.2196/82371)
Supplement: Multimedia Appendix 12 [file mental-v13-e82371-s012.docx]

**
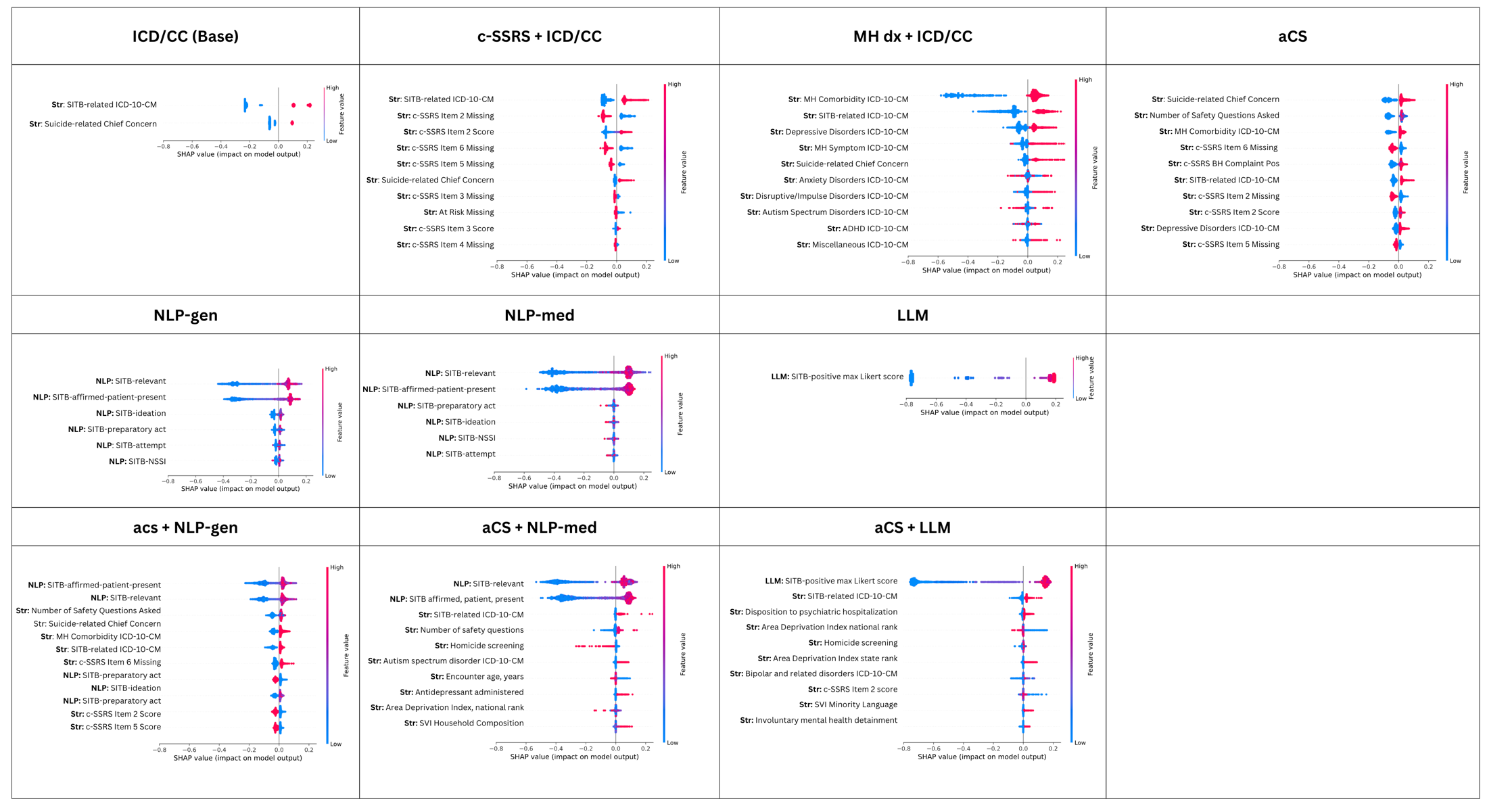
**

**Multimedia Appendix 12. SHapley** **Additive exPlanations (SHAP) Plots by Feature Set**

The diagram is a composite of SHAP plots across 10 model types: 4 structured data models (ICD/CC Base, c-SSRS + ICD/CC, MH dx + ICD/CC, aCS), 3 text models (NLP-gen, NLP-med, LLM), and 3 combined models (aCS + NLP-gen, aCS + NLP-med, aCS + LLM). Each panel displays the most influential features for a specific model, with blue dots indicating negative impact on SITB prediction and red dots indicating positive impact. SHAP values represent the contribution of each feature to the model output, with larger absolute values indicating stronger influence. The single-feature LLM model shows the strongest overall feature importance, while combined models demonstrate integration of both structured and text-derived features. *The feature set notation is as follows: ICD/CC refers to a feature set based on International Classification of Diseases, Clinical Modification, Version 10, codes for non-fatal suicide attempt and intentional self-harm, as defined by the Centers for Disease Control and Prevention Case Surveillance definition list, plus suicide-related chief concern; c-SSRS+ICD/CC combines ICD/CC with c-SSRS item scores; MH dx+ICD/CC combines ICD/CC with Child and Adolescent Mental Health Disorders Classification System ICD-10-CM code categories; aCS represents all available structured data; NLP-gen and NLP-med are feature sets based on vectorized text features with embeddings derived from the Universal Sentence Encoder and MedEmbed, respectively; LLM refers to Likert-type scores generated by the open-source language model llama-3.3-70B. Feature sets denoted by (aCS+) indicate combinations of aCS with the corresponding text-based feature set (NLP-gen, NLP-med, or LLM).*
