# Supplementary material for: Detecting Pediatric Emergency Service Use for Suicide and Self-Harm: Multimodal Analysis of 3828 Encounters
Source: JMIR Ment Health. 2026 Feb 4;13:e82371. doi: 10.2196/82371 (PMC12871580; doi:10.2196/82371)
Supplement: Multimedia Appendix 14 [file mental-v13-e82371-s014.docx]

**Multimedia Appendix 14. Detection Performance by Classifier and Age Group, by Sex and Race/Ethnicity**

|  | Age range | Subgroup | N | TN | FP | FN | TP | **Sensitivity** | **Specificity** | **Accuracy** | **ROC-AUC** |  | **PPV** | **NPV** |
| --- | --- | --- | --- | --- | --- | --- | --- | --- | --- | --- | --- | --- | --- | --- |
| ICD/CC | 6 to 17 | Total | 3102 | 497 | 1233 | 106 | 1266 | 0.923 (0.907 - 0.936) | 0.287 (0.266 - 0.309) | 0.568 (0.551 - 0.586) | 0.865 (0.852 - 0.879) |  | 0.507 (0.487 - 0.526) | 0.824 (0.791 - 0.854) |
|  |  | Female | 1564 | 242 | 542 | 43 | 737 | 0.945 (0.926 - 0.960) | 0.309 (0.276 - 0.342) | 0.626 (0.601 - 0.650) | 0.902 (0.887 - 0.918) |  | 0.576 (0.549 - 0.604) | 0.849 (0.802 - 0.889) |
|  |  | Male | 1538 | 255 | 691 | 63 | 529 | 0.894 (0.866 - 0.917) | 0.270 (0.242 - 0.299) | 0.510 (0.484 - 0.535) | 0.817 (0.794 - 0.840) |  | 0.434 (0.406 - 0.462) | 0.802 (0.754 - 0.844) |
|  |  | Asian | 137 | 26 | 42 | 3 | 66 | 0.957 (0.878 - 0.991) | 0.382 (0.267 - 0.508) | 0.672 (0.586 - 0.749) | 0.928 (0.883 - 0.974) |  | 0.611 (0.513 - 0.703) | 0.897 (0.726 - 0.978) |
|  |  | Black | 288 | 54 | 119 | 14 | 101 | 0.878 (0.804 - 0.932) | 0.312 (0.244 - 0.387) | 0.538 (0.479 - 0.597) | 0.822 (0.770 - 0.874) |  | 0.459 (0.392 - 0.527) | 0.794 (0.679 - 0.883) |
|  |  | Hispanic/Latino | 827 | 136 | 367 | 22 | 302 | 0.932 (0.899 - 0.957) | 0.270 (0.232 - 0.311) | 0.530 (0.495 - 0.564) | 0.847 (0.818 - 0.876) |  | 0.451 (0.413 - 0.490) | 0.861 (0.797 - 0.911) |
|  |  | White | 1548 | 222 | 579 | 60 | 687 | 0.920 (0.898 - 0.938) | 0.277 (0.246 - 0.310) | 0.587 (0.562 - 0.612) | 0.872 (0.854 - 0.890) |  | 0.543 (0.515 - 0.570) | 0.787 (0.735 - 0.834) |
|  |  | Other Race/Ethnicity | 257 | 51 | 111 | 4 | 91 | 0.958 (0.896 - 0.988) | 0.315 (0.244 - 0.392) | 0.553 (0.489 - 0.614) | 0.882 (0.834 - 0.929) |  | 0.450 (0.381 - 0.522) | 0.927 (0.824 - 0.980) |
|  |  | Asian, Female | 79 | 16 | 21 | 2 | 40 | 0.952 (0.838 - 0.994) | 0.432 (0.271 - 0.605) | 0.709 (0.596 - 0.806) | 0.939 (0.884 - 0.993) |  | 0.656 (0.523 - 0.773) | 0.889 (0.653 - 0.986) |
|  |  | Asian, Male | 58 | 10 | 21 | 1 | 26 | 0.963 (0.810 - 0.999) | 0.323 (0.167 - 0.514) | 0.621 (0.484 - 0.745) | 0.902 (0.818 - 0.986) |  | 0.553 (0.401 - 0.698) | 0.909 (0.587 - 0.998) |
|  |  | Black, Female | 147 | 27 | 56 | 7 | 57 | 0.891 (0.788 - 0.955) | 0.325 (0.226 - 0.437) | 0.571 (0.487 - 0.653) | 0.845 (0.778 - 0.912) |  | 0.504 (0.409 - 0.600) | 0.794 (0.621 - 0.913) |
|  |  | Black, Male | 141 | 27 | 63 | 7 | 44 | 0.863 (0.737 - 0.943) | 0.300 (0.208 - 0.406) | 0.504 (0.418 - 0.589) | 0.795 (0.713 - 0.876) |  | 0.411 (0.317 - 0.510) | 0.794 (0.621 - 0.913) |
|  |  | Hispanic, Female | 421 | 64 | 155 | 10 | 192 | 0.950 (0.911 - 0.976) | 0.292 (0.233 - 0.357) | 0.608 (0.560 - 0.655) | 0.898 (0.866 - 0.929) |  | 0.553 (0.499 - 0.606) | 0.865 (0.765 - 0.933) |
|  |  | Hispanic, Male | 406 | 72 | 212 | 12 | 110 | 0.902 (0.834 - 0.948) | 0.254 (0.204 - 0.308) | 0.448 (0.399 - 0.498) | 0.765 (0.711 - 0.819) |  | 0.342 (0.290 - 0.396) | 0.857 (0.764 - 0.924) |
|  |  | White, Female | 755 | 107 | 252 | 21 | 375 | 0.947 (0.920 - 0.967) | 0.298 (0.251 - 0.348) | 0.638 (0.603 - 0.673) | 0.912 (0.891 - 0.933) |  | 0.598 (0.559 - 0.637) | 0.836 (0.760 - 0.895) |
|  |  | White, Male | 793 | 115 | 327 | 39 | 312 | 0.889 (0.851 - 0.920) | 0.260 (0.220 - 0.304) | 0.538 (0.503 - 0.574) | 0.827 (0.797 - 0.857) |  | 0.488 (0.449 - 0.528) | 0.747 (0.670 - 0.813) |
|  |  | Other Race/Ethnicity, Female | 135 | 23 | 50 | 1 | 61 | 0.984 (0.913 - 1.000) | 0.315 (0.211 - 0.434) | 0.622 (0.535 - 0.704) | 0.904 (0.850 - 0.959) |  | 0.550 (0.452 - 0.644) | 0.958 (0.789 - 0.999) |
|  |  | Other Race/Ethnicity, Male | 122 | 28 | 61 | 3 | 30 | 0.909 (0.757 - 0.981) | 0.315 (0.220 - 0.422) | 0.475 (0.384 - 0.568) | 0.841 (0.752 - 0.930) |  | 0.330 (0.235 - 0.436) | 0.903 (0.742 - 0.980) |
|  | 6 to 12 | Total | 840 | 147 | 345 | 37 | 311 | 0.894 (0.856 - 0.924) | 0.299 (0.259 - 0.341) | 0.545 (0.511 - 0.579) | 0.822 (0.792 - 0.852) |  | 0.474 (0.435 - 0.513) | 0.799 (0.734 - 0.854) |
|  |  | Female | 329 | 60 | 113 | 8 | 148 | 0.949 (0.901 - 0.978) | 0.347 (0.276 - 0.423) | 0.632 (0.578 - 0.684) | 0.903 (0.868 - 0.937) |  | 0.567 (0.505 - 0.628) | 0.882 (0.781 - 0.948) |
|  |  | Male | 511 | 87 | 232 | 29 | 163 | 0.849 (0.790 - 0.896) | 0.273 (0.225 - 0.325) | 0.489 (0.445 - 0.534) | 0.753 (0.708 - 0.798) |  | 0.413 (0.364 - 0.463) | 0.750 (0.661 - 0.826) |
|  |  | Asian | 34 | 6 | 12 | 2 | 14 | 0.875 (0.617 - 0.984) | 0.333 (0.133 - 0.590) | 0.588 (0.407 - 0.754) | 0.837 (0.696 - 0.977) |  | 0.538 (0.334 - 0.734) | 0.750 (0.349 - 0.968) |
|  |  | Black | 95 | 18 | 35 | 7 | 35 | 0.833 (0.686 - 0.930) | 0.340 (0.215 - 0.483) | 0.558 (0.452 - 0.660) | 0.788 (0.694 - 0.883) |  | 0.500 (0.378 - 0.622) | 0.720 (0.506 - 0.879) |
|  |  | Hispanic/Latino | 254 | 51 | 118 | 5 | 80 | 0.941 (0.868 - 0.981) | 0.302 (0.234 - 0.377) | 0.516 (0.452 - 0.579) | 0.822 (0.763 - 0.881) |  | 0.404 (0.335 - 0.476) | 0.911 (0.804 - 0.970) |
|  |  | White | 370 | 58 | 134 | 21 | 157 | 0.882 (0.825 - 0.925) | 0.302 (0.238 - 0.372) | 0.581 (0.529 - 0.632) | 0.833 (0.791 - 0.875) |  | 0.540 (0.480 - 0.598) | 0.734 (0.623 - 0.827) |
|  |  | Other Race/Ethnicity | 78 | 12 | 42 | 1 | 23 | 0.958 (0.789 - 0.999) | 0.222 (0.120 - 0.356) | 0.449 (0.336 - 0.566) | 0.815 (0.702 - 0.927) |  | 0.354 (0.239 - 0.482) | 0.923 (0.640 - 0.998) |
|  |  | Asian, Female | 16 | 2 | 4 | 1 | 9 | 0.900 (0.555 - 0.997) | 0.333 (0.043 - 0.777) | 0.688 (0.413 - 0.890) | 0.925 (0.791 - 1.000) |  | 0.692 (0.386 - 0.909) | 0.667 (0.094 - 0.992) |
|  |  | Asian, Male | 18 | 4 | 8 | 1 | 5 | 0.833 (0.359 - 0.996) | 0.333 (0.099 - 0.651) | 0.500 (0.260 - 0.740) | 0.681 (0.403 - 0.959) |  | 0.385 (0.139 - 0.684) | 0.800 (0.284 - 0.995) |
|  |  | Black, Female | 34 | 11 | 10 | 2 | 11 | 0.846 (0.546 - 0.981) | 0.524 (0.298 - 0.743) | 0.647 (0.465 - 0.803) | 0.846 (0.699 - 0.993) |  | 0.524 (0.298 - 0.743) | 0.846 (0.546 - 0.981) |
|  |  | Black, Male | 61 | 7 | 25 | 5 | 24 | 0.828 (0.642 - 0.942) | 0.219 (0.093 - 0.400) | 0.508 (0.377 - 0.639) | 0.754 (0.630 - 0.877) |  | 0.490 (0.344 - 0.637) | 0.583 (0.277 - 0.848) |
|  |  | Hispanic, Female | 110 | 18 | 44 | 0 | 48 | 1.000 (0.926 - 1.000) | 0.290 (0.182 - 0.419) | 0.600 (0.502 - 0.692) | 0.929 (0.876 - 0.983) |  | 0.522 (0.415 - 0.627) | 1.000 (0.815 - 1.000) |
|  |  | Hispanic, Male | 144 | 33 | 74 | 5 | 32 | 0.865 (0.712 - 0.955) | 0.308 (0.223 - 0.405) | 0.451 (0.368 - 0.536) | 0.684 (0.579 - 0.789) |  | 0.302 (0.217 - 0.399) | 0.868 (0.719 - 0.956) |
|  |  | White, Female | 137 | 26 | 40 | 4 | 67 | 0.944 (0.862 - 0.984) | 0.394 (0.276 - 0.522) | 0.679 (0.594 - 0.756) | 0.915 (0.866 - 0.964) |  | 0.626 (0.527 - 0.718) | 0.867 (0.693 - 0.962) |
|  |  | White, Male | 233 | 32 | 94 | 17 | 90 | 0.841 (0.758 - 0.905) | 0.254 (0.181 - 0.339) | 0.524 (0.457 - 0.589) | 0.775 (0.714 - 0.836) |  | 0.489 (0.415 - 0.564) | 0.653 (0.504 - 0.783) |
|  |  | Other Race/Ethnicity, Female | 30 | 3 | 14 | 1 | 12 | 0.923 (0.640 - 0.998) | 0.176 (0.038 - 0.434) | 0.500 (0.313 - 0.687) | 0.785 (0.612 - 0.958) |  | 0.462 (0.266 - 0.666) | 0.750 (0.194 - 0.994) |
|  |  | Other Race/Ethnicity, Male | 48 | 9 | 28 | 0 | 11 | 1.000 (0.715 - 1.000) | 0.243 (0.118 - 0.412) | 0.417 (0.276 - 0.568) | 0.844 (0.691 - 0.997) |  | 0.282 (0.150 - 0.449) | 1.000 (0.664 - 1.000) |
|  | 13 to 17 | Total | 2262 | 350 | 888 | 69 | 955 | 0.933 (0.915 - 0.947) | 0.283 (0.258 - 0.309) | 0.577 (0.556 - 0.597) | 0.880 (0.866 - 0.895) |  | 0.518 (0.495 - 0.541) | 0.835 (0.796 - 0.870) |
|  |  | Female | 1235 | 182 | 429 | 35 | 589 | 0.944 (0.923 - 0.961) | 0.298 (0.262 - 0.336) | 0.624 (0.597 - 0.651) | 0.902 (0.885 - 0.920) |  | 0.579 (0.548 - 0.609) | 0.839 (0.783 - 0.885) |
|  |  | Male | 1027 | 168 | 459 | 34 | 366 | 0.915 (0.883 - 0.940) | 0.268 (0.234 - 0.304) | 0.520 (0.489 - 0.551) | 0.848 (0.822 - 0.874) |  | 0.444 (0.409 - 0.478) | 0.832 (0.773 - 0.881) |
|  |  | Asian | 103 | 20 | 30 | 1 | 52 | 0.981 (0.899 - 1.000) | 0.400 (0.264 - 0.548) | 0.699 (0.601 - 0.785) | 0.955 (0.914 - 0.996) |  | 0.634 (0.520 - 0.738) | 0.952 (0.762 - 0.999) |
|  |  | Black | 193 | 36 | 84 | 7 | 66 | 0.904 (0.812 - 0.961) | 0.300 (0.220 - 0.390) | 0.528 (0.456 - 0.601) | 0.848 (0.787 - 0.909) |  | 0.440 (0.359 - 0.523) | 0.837 (0.693 - 0.932) |
|  |  | Hispanic/Latino | 573 | 85 | 249 | 17 | 222 | 0.929 (0.889 - 0.958) | 0.254 (0.209 - 0.305) | 0.536 (0.494 - 0.577) | 0.856 (0.823 - 0.889) |  | 0.471 (0.425 - 0.518) | 0.833 (0.747 - 0.900) |
|  |  | White | 1178 | 164 | 445 | 39 | 530 | 0.931 (0.907 - 0.951) | 0.269 (0.234 - 0.306) | 0.589 (0.560 - 0.617) | 0.885 (0.865 - 0.904) |  | 0.544 (0.512 - 0.575) | 0.808 (0.747 - 0.860) |
|  |  | Other Race/Ethnicity | 179 | 39 | 69 | 3 | 68 | 0.958 (0.881 - 0.991) | 0.361 (0.271 - 0.459) | 0.598 (0.522 - 0.670) | 0.904 (0.854 - 0.954) |  | 0.496 (0.410 - 0.583) | 0.929 (0.805 - 0.985) |
|  |  | Asian, Female | 63 | 14 | 17 | 1 | 31 | 0.969 (0.838 - 0.999) | 0.452 (0.273 - 0.640) | 0.714 (0.587 - 0.821) | 0.946 (0.887 - 1.000) |  | 0.646 (0.495 - 0.778) | 0.933 (0.681 - 0.998) |
|  |  | Asian, Male | 40 | 6 | 13 | 0 | 21 | 1.000 (0.839 - 1.000) | 0.316 (0.126 - 0.566) | 0.675 (0.509 - 0.814) | 0.966 (0.909 - 1.000) |  | 0.618 (0.436 - 0.778) | 1.000 (0.541 - 1.000) |
|  |  | Black, Female | 113 | 16 | 46 | 5 | 46 | 0.902 (0.786 - 0.967) | 0.258 (0.155 - 0.385) | 0.549 (0.452 - 0.642) | 0.848 (0.773 - 0.923) |  | 0.500 (0.394 - 0.606) | 0.762 (0.528 - 0.918) |
|  |  | Black, Male | 80 | 20 | 38 | 2 | 20 | 0.909 (0.708 - 0.989) | 0.345 (0.225 - 0.481) | 0.500 (0.386 - 0.614) | 0.841 (0.732 - 0.951) |  | 0.345 (0.225 - 0.481) | 0.909 (0.708 - 0.989) |
|  |  | Hispanic, Female | 311 | 46 | 111 | 10 | 144 | 0.935 (0.884 - 0.968) | 0.293 (0.223 - 0.371) | 0.611 (0.554 - 0.665) | 0.887 (0.849 - 0.925) |  | 0.565 (0.501 - 0.626) | 0.821 (0.696 - 0.911) |
|  |  | Hispanic, Male | 262 | 39 | 138 | 7 | 78 | 0.918 (0.838 - 0.966) | 0.220 (0.162 - 0.289) | 0.447 (0.385 - 0.509) | 0.802 (0.741 - 0.864) |  | 0.361 (0.297 - 0.429) | 0.848 (0.711 - 0.937) |
|  |  | White, Female | 618 | 81 | 212 | 17 | 308 | 0.948 (0.918 - 0.969) | 0.276 (0.226 - 0.331) | 0.629 (0.590 - 0.668) | 0.912 (0.888 - 0.935) |  | 0.592 (0.549 - 0.635) | 0.827 (0.737 - 0.896) |
|  |  | White, Male | 560 | 83 | 233 | 22 | 222 | 0.910 (0.867 - 0.943) | 0.263 (0.215 - 0.315) | 0.545 (0.502 - 0.586) | 0.849 (0.816 - 0.883) |  | 0.488 (0.441 - 0.535) | 0.790 (0.700 - 0.864) |
|  |  | Other Race/Ethnicity, Female | 105 | 20 | 36 | 0 | 49 | 1.000 (0.927 - 1.000) | 0.357 (0.234 - 0.496) | 0.657 (0.558 - 0.747) | 0.932 (0.880 - 0.984) |  | 0.576 (0.464 - 0.683) | 1.000 (0.832 - 1.000) |
|  |  | Other Race/Ethnicity, Male | 74 | 19 | 33 | 3 | 19 | 0.864 (0.651 - 0.971) | 0.365 (0.236 - 0.510) | 0.514 (0.394 - 0.631) | 0.838 (0.726 - 0.949) |  | 0.365 (0.236 - 0.510) | 0.864 (0.651 - 0.971) |
|  |  |  |  |  |  |  |  |  |  |  |  |  |  |  |
| c-SSRS | 6 to 17 | Total | 3102 | 1507 | 223 | 131 | 1241 | 0.905 (0.888 - 0.920) | 0.871 (0.854 - 0.887) | 0.886 (0.874 - 0.897) | 0.935 (0.925 - 0.944) |  | 0.848 (0.828 - 0.866) | 0.920 (0.906 - 0.933) |
|  |  | Female | 1564 | 706 | 78 | 54 | 726 | 0.931 (0.911 - 0.948) | 0.901 (0.877 - 0.921) | 0.916 (0.901 - 0.929) | 0.953 (0.942 - 0.964) |  | 0.903 (0.880 - 0.923) | 0.929 (0.908 - 0.946) |
|  |  | Male | 1538 | 801 | 145 | 77 | 515 | 0.870 (0.840 - 0.896) | 0.847 (0.822 - 0.869) | 0.856 (0.837 - 0.873) | 0.912 (0.895 - 0.928) |  | 0.780 (0.747 - 0.811) | 0.912 (0.892 - 0.930) |
|  |  | Asian | 137 | 58 | 10 | 3 | 66 | 0.957 (0.878 - 0.991) | 0.853 (0.746 - 0.927) | 0.905 (0.843 - 0.949) | 0.969 (0.939 - 0.999) |  | 0.868 (0.771 - 0.935) | 0.951 (0.863 - 0.990) |
|  |  | Black | 288 | 150 | 23 | 15 | 100 | 0.870 (0.794 - 0.925) | 0.867 (0.807 - 0.914) | 0.868 (0.823 - 0.905) | 0.912 (0.874 - 0.950) |  | 0.813 (0.733 - 0.878) | 0.909 (0.854 - 0.948) |
|  |  | Hispanic/Latino | 827 | 454 | 49 | 37 | 287 | 0.886 (0.846 - 0.918) | 0.903 (0.873 - 0.927) | 0.896 (0.873 - 0.916) | 0.932 (0.912 - 0.952) |  | 0.854 (0.812 - 0.890) | 0.925 (0.898 - 0.946) |
|  |  | White | 1548 | 685 | 116 | 64 | 683 | 0.914 (0.892 - 0.933) | 0.855 (0.829 - 0.879) | 0.884 (0.867 - 0.899) | 0.935 (0.922 - 0.948) |  | 0.855 (0.828 - 0.879) | 0.915 (0.892 - 0.934) |
|  |  | Other Race/Ethnicity | 257 | 141 | 21 | 10 | 85 | 0.895 (0.815 - 0.948) | 0.870 (0.809 - 0.918) | 0.879 (0.833 - 0.917) | 0.928 (0.891 - 0.966) |  | 0.802 (0.713 - 0.873) | 0.934 (0.882 - 0.968) |
|  |  | Asian, Female | 79 | 33 | 4 | 2 | 40 | 0.952 (0.838 - 0.994) | 0.892 (0.746 - 0.970) | 0.924 (0.842 - 0.972) | 0.976 (0.941 - 1.000) |  | 0.909 (0.783 - 0.975) | 0.943 (0.808 - 0.993) |
|  |  | Asian, Male | 58 | 25 | 6 | 1 | 26 | 0.963 (0.810 - 0.999) | 0.806 (0.625 - 0.925) | 0.879 (0.767 - 0.950) | 0.961 (0.908 - 1.000) |  | 0.813 (0.636 - 0.928) | 0.962 (0.804 - 0.999) |
|  |  | Black, Female | 147 | 74 | 9 | 6 | 58 | 0.906 (0.807 - 0.965) | 0.892 (0.804 - 0.949) | 0.898 (0.837 - 0.942) | 0.926 (0.879 - 0.973) |  | 0.866 (0.760 - 0.937) | 0.925 (0.844 - 0.972) |
|  |  | Black, Male | 141 | 76 | 14 | 9 | 42 | 0.824 (0.691 - 0.916) | 0.844 (0.753 - 0.912) | 0.837 (0.765 - 0.894) | 0.895 (0.833 - 0.956) |  | 0.750 (0.616 - 0.856) | 0.894 (0.808 - 0.950) |
|  |  | Hispanic, Female | 421 | 204 | 15 | 15 | 187 | 0.926 (0.880 - 0.958) | 0.932 (0.890 - 0.961) | 0.929 (0.900 - 0.951) | 0.958 (0.937 - 0.978) |  | 0.926 (0.880 - 0.958) | 0.932 (0.890 - 0.961) |
|  |  | Hispanic, Male | 406 | 250 | 34 | 22 | 100 | 0.820 (0.740 - 0.883) | 0.880 (0.837 - 0.916) | 0.862 (0.825 - 0.894) | 0.892 (0.852 - 0.931) |  | 0.746 (0.664 - 0.817) | 0.919 (0.880 - 0.949) |
|  |  | White, Female | 755 | 320 | 39 | 23 | 373 | 0.942 (0.914 - 0.963) | 0.891 (0.854 - 0.922) | 0.918 (0.896 - 0.936) | 0.957 (0.942 - 0.972) |  | 0.905 (0.873 - 0.932) | 0.933 (0.901 - 0.957) |
|  |  | White, Male | 793 | 365 | 77 | 41 | 310 | 0.883 (0.845 - 0.915) | 0.826 (0.787 - 0.860) | 0.851 (0.824 - 0.875) | 0.911 (0.889 - 0.933) |  | 0.801 (0.758 - 0.840) | 0.899 (0.866 - 0.927) |
|  |  | Other Race/Ethnicity, Female | 135 | 64 | 9 | 6 | 56 | 0.903 (0.801 - 0.964) | 0.877 (0.779 - 0.942) | 0.889 (0.823 - 0.936) | 0.928 (0.881 - 0.976) |  | 0.862 (0.753 - 0.935) | 0.914 (0.823 - 0.968) |
|  |  | Other Race/Ethnicity, Male | 122 | 77 | 12 | 4 | 29 | 0.879 (0.718 - 0.966) | 0.865 (0.776 - 0.928) | 0.869 (0.796 - 0.923) | 0.930 (0.868 - 0.992) |  | 0.707 (0.545 - 0.839) | 0.951 (0.878 - 0.986) |
|  | 6 to 12 | Total | 840 | 435 | 57 | 57 | 291 | 0.836 (0.793 - 0.874) | 0.884 (0.853 - 0.911) | 0.864 (0.839 - 0.887) | 0.902 (0.879 - 0.925) |  | 0.836 (0.793 - 0.874) | 0.884 (0.853 - 0.911) |
|  |  | Female | 329 | 159 | 14 | 11 | 145 | 0.929 (0.877 - 0.964) | 0.919 (0.868 - 0.955) | 0.924 (0.890 - 0.950) | 0.960 (0.938 - 0.982) |  | 0.912 (0.857 - 0.951) | 0.935 (0.887 - 0.967) |
|  |  | Male | 511 | 276 | 43 | 46 | 146 | 0.760 (0.694 - 0.819) | 0.865 (0.823 - 0.901) | 0.826 (0.790 - 0.858) | 0.852 (0.815 - 0.889) |  | 0.772 (0.706 - 0.830) | 0.857 (0.814 - 0.893) |
|  |  | Asian | 34 | 15 | 3 | 1 | 15 | 0.938 (0.698 - 0.998) | 0.833 (0.586 - 0.964) | 0.882 (0.725 - 0.967) | 0.918 (0.817 - 1.000) |  | 0.833 (0.586 - 0.964) | 0.938 (0.698 - 0.998) |
|  |  | Black | 95 | 45 | 8 | 7 | 35 | 0.833 (0.686 - 0.930) | 0.849 (0.724 - 0.933) | 0.842 (0.753 - 0.909) | 0.888 (0.816 - 0.959) |  | 0.814 (0.666 - 0.916) | 0.865 (0.742 - 0.944) |
|  |  | Hispanic/Latino | 254 | 158 | 11 | 13 | 72 | 0.847 (0.753 - 0.916) | 0.935 (0.887 - 0.967) | 0.906 (0.863 - 0.939) | 0.916 (0.874 - 0.959) |  | 0.867 (0.775 - 0.932) | 0.924 (0.874 - 0.959) |
|  |  | White | 370 | 165 | 27 | 32 | 146 | 0.820 (0.756 - 0.874) | 0.859 (0.802 - 0.905) | 0.841 (0.799 - 0.876) | 0.888 (0.854 - 0.923) |  | 0.844 (0.781 - 0.895) | 0.838 (0.778 - 0.886) |
|  |  | Other Race/Ethnicity | 78 | 48 | 6 | 4 | 20 | 0.833 (0.626 - 0.953) | 0.889 (0.774 - 0.958) | 0.872 (0.777 - 0.937) | 0.917 (0.837 - 0.997) |  | 0.769 (0.564 - 0.910) | 0.923 (0.815 - 0.979) |
|  |  | Asian, Female | 16 | 5 | 1 | 0 | 10 | 1.000 (0.692 - 1.000) | 0.833 (0.359 - 0.996) | 0.938 (0.698 - 0.998) | 0.967 (0.879 - 1.000) |  | 0.909 (0.587 - 0.998) | 1.000 (0.478 - 1.000) |
|  |  | Asian, Male | 18 | 10 | 2 | 1 | 5 | 0.833 (0.359 - 0.996) | 0.833 (0.516 - 0.979) | 0.833 (0.586 - 0.964) | 0.840 (0.621 - 1.000) |  | 0.714 (0.290 - 0.963) | 0.909 (0.587 - 0.998) |
|  |  | Black, Female | 34 | 18 | 3 | 0 | 13 | 1.000 (0.753 - 1.000) | 0.857 (0.637 - 0.970) | 0.912 (0.763 - 0.981) | 0.949 (0.861 - 1.000) |  | 0.813 (0.544 - 0.960) | 1.000 (0.815 - 1.000) |
|  |  | Black, Male | 61 | 27 | 5 | 7 | 22 | 0.759 (0.565 - 0.897) | 0.844 (0.672 - 0.947) | 0.803 (0.682 - 0.894) | 0.848 (0.747 - 0.948) |  | 0.815 (0.619 - 0.937) | 0.794 (0.621 - 0.913) |
|  |  | Hispanic, Female | 110 | 61 | 1 | 2 | 46 | 0.958 (0.857 - 0.995) | 0.984 (0.913 - 1.000) | 0.973 (0.922 - 0.994) | 0.980 (0.952 - 1.000) |  | 0.979 (0.887 - 0.999) | 0.968 (0.890 - 0.996) |
|  |  | Hispanic, Male | 144 | 97 | 10 | 11 | 26 | 0.703 (0.530 - 0.841) | 0.907 (0.835 - 0.954) | 0.854 (0.786 - 0.907) | 0.832 (0.746 - 0.918) |  | 0.722 (0.548 - 0.858) | 0.898 (0.825 - 0.948) |
|  |  | White, Female | 137 | 59 | 7 | 8 | 63 | 0.887 (0.790 - 0.950) | 0.894 (0.794 - 0.956) | 0.891 (0.826 - 0.937) | 0.950 (0.913 - 0.988) |  | 0.900 (0.805 - 0.959) | 0.881 (0.778 - 0.947) |
|  |  | White, Male | 233 | 106 | 20 | 24 | 83 | 0.776 (0.685 - 0.851) | 0.841 (0.766 - 0.900) | 0.811 (0.755 - 0.859) | 0.843 (0.791 - 0.896) |  | 0.806 (0.716 - 0.877) | 0.815 (0.738 - 0.878) |
|  |  | Other Race/Ethnicity, Female | 30 | 15 | 2 | 1 | 12 | 0.923 (0.640 - 0.998) | 0.882 (0.636 - 0.985) | 0.900 (0.735 - 0.979) | 0.928 (0.822 - 1.000) |  | 0.857 (0.572 - 0.982) | 0.938 (0.698 - 0.998) |
|  |  | Other Race/Ethnicity, Male | 48 | 33 | 4 | 3 | 8 | 0.727 (0.390 - 0.940) | 0.892 (0.746 - 0.970) | 0.854 (0.722 - 0.939) | 0.915 (0.797 - 1.000) |  | 0.667 (0.349 - 0.901) | 0.917 (0.775 - 0.982) |
|  | 13 to 17 | Total | 2262 | 1072 | 166 | 74 | 950 | 0.928 (0.910 - 0.943) | 0.866 (0.846 - 0.884) | 0.894 (0.880 - 0.906) | 0.946 (0.936 - 0.956) |  | 0.851 (0.829 - 0.872) | 0.935 (0.920 - 0.949) |
|  |  | Female | 1235 | 547 | 64 | 43 | 581 | 0.931 (0.908 - 0.950) | 0.895 (0.868 - 0.918) | 0.913 (0.896 - 0.928) | 0.951 (0.938 - 0.963) |  | 0.901 (0.875 - 0.923) | 0.927 (0.903 - 0.947) |
|  |  | Male | 1027 | 525 | 102 | 31 | 369 | 0.923 (0.892 - 0.947) | 0.837 (0.806 - 0.865) | 0.870 (0.848 - 0.890) | 0.940 (0.923 - 0.957) |  | 0.783 (0.743 - 0.820) | 0.944 (0.922 - 0.962) |
|  |  | Asian | 103 | 43 | 7 | 2 | 51 | 0.962 (0.870 - 0.995) | 0.860 (0.733 - 0.942) | 0.913 (0.841 - 0.959) | 0.982 (0.957 - 1.000) |  | 0.879 (0.767 - 0.950) | 0.956 (0.849 - 0.995) |
|  |  | Black | 193 | 105 | 15 | 8 | 65 | 0.890 (0.795 - 0.951) | 0.875 (0.802 - 0.928) | 0.881 (0.827 - 0.923) | 0.921 (0.876 - 0.966) |  | 0.813 (0.710 - 0.891) | 0.929 (0.865 - 0.969) |
|  |  | Hispanic/Latino | 573 | 296 | 38 | 24 | 215 | 0.900 (0.854 - 0.935) | 0.886 (0.847 - 0.918) | 0.892 (0.863 - 0.916) | 0.938 (0.915 - 0.960) |  | 0.850 (0.800 - 0.891) | 0.925 (0.890 - 0.951) |
|  |  | White | 1178 | 520 | 89 | 32 | 537 | 0.944 (0.922 - 0.961) | 0.854 (0.823 - 0.881) | 0.897 (0.879 - 0.914) | 0.951 (0.938 - 0.964) |  | 0.858 (0.828 - 0.884) | 0.942 (0.919 - 0.960) |
|  |  | Other Race/Ethnicity | 179 | 93 | 15 | 6 | 65 | 0.915 (0.825 - 0.968) | 0.861 (0.781 - 0.920) | 0.883 (0.826 - 0.926) | 0.932 (0.890 - 0.975) |  | 0.813 (0.710 - 0.891) | 0.939 (0.873 - 0.977) |
|  |  | Asian, Female | 63 | 28 | 3 | 2 | 30 | 0.938 (0.792 - 0.992) | 0.903 (0.742 - 0.980) | 0.921 (0.824 - 0.974) | 0.979 (0.943 - 1.000) |  | 0.909 (0.757 - 0.981) | 0.933 (0.779 - 0.992) |
|  |  | Asian, Male | 40 | 15 | 4 | 0 | 21 | 1.000 (0.839 - 1.000) | 0.789 (0.544 - 0.939) | 0.900 (0.763 - 0.972) | 0.995 (0.973 - 1.000) |  | 0.840 (0.639 - 0.955) | 1.000 (0.782 - 1.000) |
|  |  | Black, Female | 113 | 56 | 6 | 6 | 45 | 0.882 (0.761 - 0.956) | 0.903 (0.801 - 0.964) | 0.894 (0.822 - 0.944) | 0.917 (0.860 - 0.973) |  | 0.882 (0.761 - 0.956) | 0.903 (0.801 - 0.964) |
|  |  | Black, Male | 80 | 49 | 9 | 2 | 20 | 0.909 (0.708 - 0.989) | 0.845 (0.726 - 0.927) | 0.863 (0.767 - 0.929) | 0.943 (0.874 - 1.000) |  | 0.690 (0.492 - 0.847) | 0.961 (0.865 - 0.995) |
|  |  | Hispanic, Female | 311 | 143 | 14 | 13 | 141 | 0.916 (0.860 - 0.954) | 0.911 (0.855 - 0.950) | 0.913 (0.876 - 0.942) | 0.949 (0.924 - 0.975) |  | 0.910 (0.853 - 0.950) | 0.917 (0.862 - 0.955) |
|  |  | Hispanic, Male | 262 | 153 | 24 | 11 | 74 | 0.871 (0.780 - 0.934) | 0.864 (0.805 - 0.911) | 0.866 (0.819 - 0.905) | 0.918 (0.876 - 0.960) |  | 0.755 (0.658 - 0.836) | 0.933 (0.883 - 0.966) |
|  |  | White, Female | 618 | 261 | 32 | 15 | 310 | 0.954 (0.925 - 0.974) | 0.891 (0.849 - 0.924) | 0.924 (0.900 - 0.944) | 0.959 (0.943 - 0.975) |  | 0.906 (0.870 - 0.935) | 0.946 (0.912 - 0.969) |
|  |  | White, Male | 560 | 259 | 57 | 17 | 227 | 0.930 (0.891 - 0.959) | 0.820 (0.773 - 0.860) | 0.868 (0.837 - 0.895) | 0.941 (0.919 - 0.962) |  | 0.799 (0.748 - 0.844) | 0.938 (0.903 - 0.964) |
|  |  | Other Race/Ethnicity, Female | 105 | 49 | 7 | 5 | 44 | 0.898 (0.778 - 0.966) | 0.875 (0.759 - 0.948) | 0.886 (0.809 - 0.940) | 0.930 (0.877 - 0.983) |  | 0.863 (0.737 - 0.943) | 0.907 (0.797 - 0.969) |
|  |  | Other Race/Ethnicity, Male | 74 | 44 | 8 | 1 | 21 | 0.955 (0.772 - 0.999) | 0.846 (0.719 - 0.931) | 0.878 (0.782 - 0.943) | 0.939 (0.867 - 1.000) |  | 0.724 (0.528 - 0.873) | 0.978 (0.882 - 0.999) |
|  |  |  |  |  |  |  |  |  |  |  |  |  |  |  |
| MH Dx | 6 to 17 | Total | 3102 | 1208 | 522 | 78 | 1294 | 0.943 (0.930 - 0.955) | 0.698 (0.676 - 0.720) | 0.807 (0.792 - 0.820) | 0.934 (0.924 - 0.943) |  | 0.713 (0.691 - 0.733) | 0.939 (0.925 - 0.952) |
|  |  | Female | 1564 | 527 | 257 | 41 | 739 | 0.947 (0.929 - 0.962) | 0.672 (0.638 - 0.705) | 0.809 (0.789 - 0.829) | 0.949 (0.938 - 0.960) |  | 0.742 (0.714 - 0.769) | 0.928 (0.903 - 0.948) |
|  |  | Male | 1538 | 681 | 265 | 37 | 555 | 0.938 (0.915 - 0.956) | 0.720 (0.690 - 0.748) | 0.804 (0.783 - 0.823) | 0.917 (0.900 - 0.933) |  | 0.677 (0.644 - 0.709) | 0.948 (0.930 - 0.963) |
|  |  | Asian | 137 | 49 | 19 | 0 | 69 | 1.000 (0.948 - 1.000) | 0.721 (0.599 - 0.823) | 0.861 (0.792 - 0.914) | 0.968 (0.937 - 0.998) |  | 0.784 (0.684 - 0.865) | 1.000 (0.927 - 1.000) |
|  |  | Black | 288 | 114 | 59 | 10 | 105 | 0.913 (0.846 - 0.958) | 0.659 (0.583 - 0.729) | 0.760 (0.707 - 0.809) | 0.922 (0.886 - 0.958) |  | 0.640 (0.562 - 0.714) | 0.919 (0.857 - 0.961) |
|  |  | Hispanic/Latino | 827 | 382 | 121 | 29 | 295 | 0.910 (0.874 - 0.939) | 0.759 (0.720 - 0.796) | 0.819 (0.791 - 0.844) | 0.923 (0.902 - 0.944) |  | 0.709 (0.663 - 0.752) | 0.929 (0.900 - 0.952) |
|  |  | White | 1548 | 532 | 269 | 32 | 715 | 0.957 (0.940 - 0.971) | 0.664 (0.630 - 0.697) | 0.806 (0.785 - 0.825) | 0.938 (0.925 - 0.951) |  | 0.727 (0.698 - 0.754) | 0.943 (0.921 - 0.961) |
|  |  | Other Race/Ethnicity | 257 | 117 | 45 | 7 | 88 | 0.926 (0.854 - 0.970) | 0.722 (0.647 - 0.790) | 0.798 (0.743 - 0.845) | 0.922 (0.883 - 0.961) |  | 0.662 (0.575 - 0.741) | 0.944 (0.887 - 0.977) |
|  |  | Asian, Female | 79 | 28 | 9 | 0 | 42 | 1.000 (0.916 - 1.000) | 0.757 (0.588 - 0.882) | 0.886 (0.795 - 0.947) | 0.984 (0.956 - 1.000) |  | 0.824 (0.691 - 0.916) | 1.000 (0.877 - 1.000) |
|  |  | Asian, Male | 58 | 21 | 10 | 0 | 27 | 1.000 (0.872 - 1.000) | 0.677 (0.486 - 0.833) | 0.828 (0.706 - 0.914) | 0.947 (0.884 - 1.000) |  | 0.730 (0.559 - 0.862) | 1.000 (0.839 - 1.000) |
|  |  | Black, Female | 147 | 44 | 39 | 7 | 57 | 0.891 (0.788 - 0.955) | 0.530 (0.417 - 0.641) | 0.687 (0.605 - 0.761) | 0.903 (0.850 - 0.957) |  | 0.594 (0.489 - 0.693) | 0.863 (0.737 - 0.943) |
|  |  | Black, Male | 141 | 70 | 20 | 3 | 48 | 0.941 (0.838 - 0.988) | 0.778 (0.678 - 0.859) | 0.837 (0.765 - 0.894) | 0.944 (0.898 - 0.989) |  | 0.706 (0.583 - 0.810) | 0.959 (0.885 - 0.991) |
|  |  | Hispanic, Female | 421 | 166 | 53 | 14 | 188 | 0.931 (0.886 - 0.962) | 0.758 (0.696 - 0.813) | 0.841 (0.802 - 0.874) | 0.943 (0.920 - 0.967) |  | 0.780 (0.722 - 0.831) | 0.922 (0.873 - 0.957) |
|  |  | Hispanic, Male | 406 | 216 | 68 | 15 | 107 | 0.877 (0.805 - 0.930) | 0.761 (0.707 - 0.809) | 0.796 (0.753 - 0.834) | 0.899 (0.860 - 0.937) |  | 0.611 (0.535 - 0.684) | 0.935 (0.895 - 0.963) |
|  |  | White, Female | 755 | 231 | 128 | 15 | 381 | 0.962 (0.938 - 0.979) | 0.643 (0.591 - 0.693) | 0.811 (0.781 - 0.838) | 0.959 (0.944 - 0.973) |  | 0.749 (0.708 - 0.786) | 0.939 (0.901 - 0.965) |
|  |  | White, Male | 793 | 301 | 141 | 17 | 334 | 0.952 (0.924 - 0.972) | 0.681 (0.635 - 0.724) | 0.801 (0.771 - 0.828) | 0.917 (0.896 - 0.938) |  | 0.703 (0.660 - 0.744) | 0.947 (0.916 - 0.969) |
|  |  | Other Race/Ethnicity, Female | 135 | 50 | 23 | 5 | 57 | 0.919 (0.822 - 0.973) | 0.685 (0.566 - 0.789) | 0.793 (0.714 - 0.858) | 0.934 (0.888 - 0.979) |  | 0.713 (0.600 - 0.808) | 0.909 (0.800 - 0.970) |
|  |  | Other Race/Ethnicity, Male | 122 | 67 | 22 | 2 | 31 | 0.939 (0.798 - 0.993) | 0.753 (0.650 - 0.838) | 0.803 (0.722 - 0.870) | 0.908 (0.838 - 0.979) |  | 0.585 (0.441 - 0.719) | 0.971 (0.899 - 0.996) |
|  | 6 to 12 | Total | 840 | 352 | 140 | 24 | 324 | 0.931 (0.899 - 0.955) | 0.715 (0.673 - 0.755) | 0.805 (0.776 - 0.831) | 0.924 (0.903 - 0.944) |  | 0.698 (0.654 - 0.740) | 0.936 (0.907 - 0.959) |
|  |  | Female | 329 | 122 | 51 | 11 | 145 | 0.929 (0.877 - 0.964) | 0.705 (0.631 - 0.772) | 0.812 (0.765 - 0.852) | 0.944 (0.918 - 0.971) |  | 0.740 (0.672 - 0.800) | 0.917 (0.857 - 0.958) |
|  |  | Male | 511 | 230 | 89 | 13 | 179 | 0.932 (0.887 - 0.963) | 0.721 (0.668 - 0.770) | 0.800 (0.763 - 0.834) | 0.908 (0.878 - 0.938) |  | 0.668 (0.608 - 0.724) | 0.947 (0.910 - 0.971) |
|  |  | Asian | 34 | 14 | 4 | 0 | 16 | 1.000 (0.794 - 1.000) | 0.778 (0.524 - 0.936) | 0.882 (0.725 - 0.967) | 0.962 (0.892 - 1.000) |  | 0.800 (0.563 - 0.943) | 1.000 (0.768 - 1.000) |
|  |  | Black | 95 | 32 | 21 | 4 | 38 | 0.905 (0.774 - 0.973) | 0.604 (0.460 - 0.735) | 0.737 (0.636 - 0.822) | 0.889 (0.819 - 0.960) |  | 0.644 (0.509 - 0.764) | 0.889 (0.739 - 0.969) |
|  |  | Hispanic/Latino | 254 | 138 | 31 | 9 | 76 | 0.894 (0.808 - 0.950) | 0.817 (0.750 - 0.872) | 0.843 (0.792 - 0.885) | 0.925 (0.884 - 0.965) |  | 0.710 (0.615 - 0.794) | 0.939 (0.887 - 0.972) |
|  |  | White | 370 | 123 | 69 | 9 | 169 | 0.949 (0.906 - 0.977) | 0.641 (0.568 - 0.708) | 0.789 (0.744 - 0.830) | 0.922 (0.892 - 0.951) |  | 0.710 (0.648 - 0.767) | 0.932 (0.875 - 0.968) |
|  |  | Other Race/Ethnicity | 78 | 43 | 11 | 2 | 22 | 0.917 (0.730 - 0.990) | 0.796 (0.665 - 0.894) | 0.833 (0.732 - 0.908) | 0.941 (0.873 - 1.000) |  | 0.667 (0.482 - 0.820) | 0.956 (0.849 - 0.995) |
|  |  | Asian, Female | 16 | 6 | 0 | 0 | 10 | 1.000 (0.692 - 1.000) | 1.000 (0.541 - 1.000) | 1.000 (0.794 - 1.000) | 1.000 (1.000 - 1.000) |  | 1.000 (0.692 - 1.000) | 1.000 (0.541 - 1.000) |
|  |  | Asian, Male | 18 | 8 | 4 | 0 | 6 | 1.000 (0.541 - 1.000) | 0.667 (0.349 - 0.901) | 0.778 (0.524 - 0.936) | 0.903 (0.726 - 1.000) |  | 0.600 (0.262 - 0.878) | 1.000 (0.631 - 1.000) |
|  |  | Black, Female | 34 | 9 | 12 | 2 | 11 | 0.846 (0.546 - 0.981) | 0.429 (0.218 - 0.660) | 0.588 (0.407 - 0.754) | 0.813 (0.654 - 0.972) |  | 0.478 (0.268 - 0.694) | 0.818 (0.482 - 0.977) |
|  |  | Black, Male | 61 | 23 | 9 | 2 | 27 | 0.931 (0.772 - 0.992) | 0.719 (0.533 - 0.863) | 0.820 (0.700 - 0.906) | 0.921 (0.848 - 0.995) |  | 0.750 (0.578 - 0.879) | 0.920 (0.740 - 0.990) |
|  |  | Hispanic, Female | 110 | 55 | 7 | 3 | 45 | 0.938 (0.828 - 0.987) | 0.887 (0.781 - 0.953) | 0.909 (0.839 - 0.956) | 0.967 (0.930 - 1.000) |  | 0.865 (0.742 - 0.944) | 0.948 (0.856 - 0.989) |
|  |  | Hispanic, Male | 144 | 83 | 24 | 6 | 31 | 0.838 (0.680 - 0.938) | 0.776 (0.685 - 0.851) | 0.792 (0.716 - 0.855) | 0.871 (0.794 - 0.948) |  | 0.564 (0.423 - 0.697) | 0.933 (0.859 - 0.975) |
|  |  | White, Female | 137 | 40 | 26 | 4 | 67 | 0.944 (0.862 - 0.984) | 0.606 (0.478 - 0.724) | 0.781 (0.702 - 0.847) | 0.933 (0.890 - 0.977) |  | 0.720 (0.618 - 0.809) | 0.909 (0.783 - 0.975) |
|  |  | White, Male | 233 | 83 | 43 | 5 | 102 | 0.953 (0.894 - 0.985) | 0.659 (0.569 - 0.741) | 0.794 (0.736 - 0.844) | 0.916 (0.878 - 0.955) |  | 0.703 (0.622 - 0.776) | 0.943 (0.872 - 0.981) |
|  |  | Other Race/Ethnicity, Female | 30 | 12 | 5 | 2 | 11 | 0.846 (0.546 - 0.981) | 0.706 (0.440 - 0.897) | 0.767 (0.577 - 0.901) | 0.910 (0.792 - 1.000) |  | 0.688 (0.413 - 0.890) | 0.857 (0.572 - 0.982) |
|  |  | Other Race/Ethnicity, Male | 48 | 31 | 6 | 0 | 11 | 1.000 (0.715 - 1.000) | 0.838 (0.680 - 0.938) | 0.875 (0.748 - 0.953) | 0.971 (0.899 - 1.000) |  | 0.647 (0.383 - 0.858) | 1.000 (0.888 - 1.000) |
|  | 13 to 17 | Total | 2262 | 856 | 382 | 54 | 970 | 0.947 (0.932 - 0.960) | 0.691 (0.665 - 0.717) | 0.807 (0.790 - 0.823) | 0.937 (0.926 - 0.948) |  | 0.717 (0.693 - 0.741) | 0.941 (0.923 - 0.955) |
|  |  | Female | 1235 | 405 | 206 | 30 | 594 | 0.952 (0.932 - 0.967) | 0.663 (0.624 - 0.700) | 0.809 (0.786 - 0.830) | 0.951 (0.939 - 0.963) |  | 0.743 (0.711 - 0.772) | 0.931 (0.903 - 0.953) |
|  |  | Male | 1027 | 451 | 176 | 24 | 376 | 0.940 (0.912 - 0.961) | 0.719 (0.682 - 0.754) | 0.805 (0.780 - 0.829) | 0.921 (0.902 - 0.940) |  | 0.681 (0.640 - 0.720) | 0.949 (0.926 - 0.967) |
|  |  | Asian | 103 | 35 | 15 | 0 | 53 | 1.000 (0.933 - 1.000) | 0.700 (0.554 - 0.821) | 0.854 (0.771 - 0.916) | 0.970 (0.936 - 1.000) |  | 0.779 (0.662 - 0.871) | 1.000 (0.900 - 1.000) |
|  |  | Black | 193 | 82 | 38 | 6 | 67 | 0.918 (0.830 - 0.969) | 0.683 (0.592 - 0.765) | 0.772 (0.706 - 0.829) | 0.939 (0.899 - 0.979) |  | 0.638 (0.539 - 0.730) | 0.932 (0.857 - 0.975) |
|  |  | Hispanic/Latino | 573 | 244 | 90 | 20 | 219 | 0.916 (0.874 - 0.948) | 0.731 (0.680 - 0.777) | 0.808 (0.773 - 0.839) | 0.921 (0.896 - 0.946) |  | 0.709 (0.655 - 0.759) | 0.924 (0.885 - 0.953) |
|  |  | White | 1178 | 409 | 200 | 23 | 546 | 0.960 (0.940 - 0.974) | 0.672 (0.633 - 0.709) | 0.811 (0.787 - 0.833) | 0.943 (0.929 - 0.957) |  | 0.732 (0.699 - 0.763) | 0.947 (0.921 - 0.966) |
|  |  | Other Race/Ethnicity | 179 | 74 | 34 | 5 | 66 | 0.930 (0.843 - 0.977) | 0.685 (0.589 - 0.771) | 0.782 (0.714 - 0.840) | 0.914 (0.867 - 0.962) |  | 0.660 (0.558 - 0.752) | 0.937 (0.858 - 0.979) |
|  |  | Asian, Female | 63 | 22 | 9 | 0 | 32 | 1.000 (0.891 - 1.000) | 0.710 (0.520 - 0.858) | 0.857 (0.746 - 0.933) | 0.981 (0.946 - 1.000) |  | 0.780 (0.624 - 0.894) | 1.000 (0.846 - 1.000) |
|  |  | Asian, Male | 40 | 13 | 6 | 0 | 21 | 1.000 (0.839 - 1.000) | 0.684 (0.434 - 0.874) | 0.850 (0.702 - 0.943) | 0.951 (0.882 - 1.000) |  | 0.778 (0.577 - 0.914) | 1.000 (0.753 - 1.000) |
|  |  | Black, Female | 113 | 35 | 27 | 5 | 46 | 0.902 (0.786 - 0.967) | 0.565 (0.433 - 0.690) | 0.717 (0.624 - 0.798) | 0.919 (0.863 - 0.974) |  | 0.630 (0.509 - 0.740) | 0.875 (0.732 - 0.958) |
|  |  | Black, Male | 80 | 47 | 11 | 1 | 21 | 0.955 (0.772 - 0.999) | 0.810 (0.686 - 0.901) | 0.850 (0.753 - 0.920) | 0.969 (0.917 - 1.000) |  | 0.656 (0.468 - 0.814) | 0.979 (0.889 - 0.999) |
|  |  | Hispanic, Female | 311 | 111 | 46 | 11 | 143 | 0.929 (0.876 - 0.964) | 0.707 (0.629 - 0.777) | 0.817 (0.769 - 0.858) | 0.933 (0.904 - 0.962) |  | 0.757 (0.689 - 0.816) | 0.910 (0.844 - 0.954) |
|  |  | Hispanic, Male | 262 | 133 | 44 | 9 | 76 | 0.894 (0.808 - 0.950) | 0.751 (0.681 - 0.813) | 0.798 (0.744 - 0.845) | 0.908 (0.864 - 0.952) |  | 0.633 (0.541 - 0.719) | 0.937 (0.883 - 0.971) |
|  |  | White, Female | 618 | 191 | 102 | 11 | 314 | 0.966 (0.940 - 0.983) | 0.652 (0.594 - 0.706) | 0.817 (0.784 - 0.847) | 0.964 (0.949 - 0.979) |  | 0.755 (0.711 - 0.795) | 0.946 (0.905 - 0.973) |
|  |  | White, Male | 560 | 218 | 98 | 12 | 232 | 0.951 (0.916 - 0.974) | 0.690 (0.636 - 0.740) | 0.804 (0.768 - 0.836) | 0.919 (0.894 - 0.944) |  | 0.703 (0.651 - 0.752) | 0.948 (0.911 - 0.973) |
|  |  | Other Race/Ethnicity, Female | 105 | 38 | 18 | 3 | 46 | 0.939 (0.831 - 0.987) | 0.679 (0.540 - 0.797) | 0.800 (0.711 - 0.872) | 0.941 (0.892 - 0.990) |  | 0.719 (0.592 - 0.824) | 0.927 (0.801 - 0.985) |
|  |  | Other Race/Ethnicity, Male | 74 | 36 | 16 | 2 | 20 | 0.909 (0.708 - 0.989) | 0.692 (0.549 - 0.813) | 0.757 (0.643 - 0.849) | 0.873 (0.772 - 0.973) |  | 0.556 (0.381 - 0.721) | 0.947 (0.823 - 0.994) |
|  |  |  |  |  |  |  |  |  |  |  |  |  |  |  |
| aCS | 6 to 17 | Total | 3102 | 1328 | 402 | 26 | 1346 | 0.981 (0.972 - 0.988) | 0.768 (0.747 - 0.787) | 0.862 (0.849 - 0.874) | 0.965 (0.958 - 0.972) |  | 0.770 (0.750 - 0.790) | 0.981 (0.972 - 0.987) |
|  |  | Female | 1564 | 633 | 151 | 13 | 767 | 0.983 (0.972 - 0.991) | 0.807 (0.778 - 0.834) | 0.895 (0.879 - 0.910) | 0.976 (0.968 - 0.984) |  | 0.836 (0.810 - 0.859) | 0.980 (0.966 - 0.989) |
|  |  | Male | 1538 | 695 | 251 | 13 | 579 | 0.978 (0.963 - 0.988) | 0.735 (0.705 - 0.763) | 0.828 (0.809 - 0.847) | 0.951 (0.939 - 0.964) |  | 0.698 (0.665 - 0.729) | 0.982 (0.969 - 0.990) |
|  |  | Asian | 137 | 53 | 15 | 1 | 68 | 0.986 (0.922 - 1.000) | 0.779 (0.662 - 0.871) | 0.883 (0.817 - 0.932) | 0.973 (0.946 - 1.000) |  | 0.819 (0.720 - 0.895) | 0.981 (0.901 - 1.000) |
|  |  | Black | 288 | 126 | 47 | 4 | 111 | 0.965 (0.913 - 0.990) | 0.728 (0.656 - 0.793) | 0.823 (0.774 - 0.865) | 0.955 (0.927 - 0.982) |  | 0.703 (0.625 - 0.773) | 0.969 (0.923 - 0.992) |
|  |  | Hispanic/Latino | 827 | 418 | 85 | 10 | 314 | 0.969 (0.944 - 0.985) | 0.831 (0.795 - 0.863) | 0.885 (0.861 - 0.906) | 0.970 (0.956 - 0.983) |  | 0.787 (0.743 - 0.826) | 0.977 (0.957 - 0.989) |
|  |  | White | 1548 | 587 | 214 | 9 | 738 | 0.988 (0.977 - 0.994) | 0.733 (0.701 - 0.763) | 0.856 (0.837 - 0.873) | 0.962 (0.952 - 0.972) |  | 0.775 (0.747 - 0.801) | 0.985 (0.972 - 0.993) |
|  |  | Other Race/Ethnicity | 257 | 127 | 35 | 2 | 93 | 0.979 (0.926 - 0.997) | 0.784 (0.713 - 0.845) | 0.856 (0.807 - 0.897) | 0.972 (0.948 - 0.996) |  | 0.727 (0.641 - 0.802) | 0.984 (0.945 - 0.998) |
|  |  | Asian, Female | 79 | 31 | 6 | 0 | 42 | 1.000 (0.916 - 1.000) | 0.838 (0.680 - 0.938) | 0.924 (0.842 - 0.972) | 0.986 (0.961 - 1.000) |  | 0.875 (0.748 - 0.953) | 1.000 (0.888 - 1.000) |
|  |  | Asian, Male | 58 | 22 | 9 | 1 | 26 | 0.963 (0.810 - 0.999) | 0.710 (0.520 - 0.858) | 0.828 (0.706 - 0.914) | 0.955 (0.897 - 1.000) |  | 0.743 (0.567 - 0.875) | 0.957 (0.781 - 0.999) |
|  |  | Black, Female | 147 | 63 | 20 | 4 | 60 | 0.938 (0.848 - 0.983) | 0.759 (0.653 - 0.846) | 0.837 (0.767 - 0.893) | 0.958 (0.922 - 0.994) |  | 0.750 (0.641 - 0.840) | 0.940 (0.854 - 0.983) |
|  |  | Black, Male | 141 | 63 | 27 | 0 | 51 | 1.000 (0.930 - 1.000) | 0.700 (0.594 - 0.792) | 0.809 (0.734 - 0.870) | 0.950 (0.906 - 0.993) |  | 0.654 (0.538 - 0.758) | 1.000 (0.943 - 1.000) |
|  |  | Hispanic, Female | 421 | 187 | 32 | 4 | 198 | 0.980 (0.950 - 0.995) | 0.854 (0.800 - 0.898) | 0.914 (0.884 - 0.939) | 0.981 (0.967 - 0.994) |  | 0.861 (0.809 - 0.903) | 0.979 (0.947 - 0.994) |
|  |  | Hispanic, Male | 406 | 231 | 53 | 6 | 116 | 0.951 (0.896 - 0.982) | 0.813 (0.763 - 0.857) | 0.855 (0.817 - 0.887) | 0.952 (0.924 - 0.979) |  | 0.686 (0.611 - 0.755) | 0.975 (0.946 - 0.991) |
|  |  | White, Female | 755 | 284 | 75 | 4 | 392 | 0.990 (0.974 - 0.997) | 0.791 (0.745 - 0.832) | 0.895 (0.871 - 0.916) | 0.975 (0.964 - 0.986) |  | 0.839 (0.803 - 0.872) | 0.986 (0.965 - 0.996) |
|  |  | White, Male | 793 | 303 | 139 | 5 | 346 | 0.986 (0.967 - 0.995) | 0.686 (0.640 - 0.729) | 0.818 (0.790 - 0.845) | 0.946 (0.928 - 0.963) |  | 0.713 (0.671 - 0.753) | 0.984 (0.963 - 0.995) |
|  |  | Other Race/Ethnicity, Female | 135 | 59 | 14 | 1 | 61 | 0.984 (0.913 - 1.000) | 0.808 (0.699 - 0.891) | 0.889 (0.823 - 0.936) | 0.981 (0.956 - 1.000) |  | 0.813 (0.707 - 0.894) | 0.983 (0.911 - 1.000) |
|  |  | Other Race/Ethnicity, Male | 122 | 68 | 21 | 1 | 32 | 0.970 (0.842 - 0.999) | 0.764 (0.662 - 0.848) | 0.820 (0.740 - 0.883) | 0.956 (0.907 - 1.000) |  | 0.604 (0.460 - 0.735) | 0.986 (0.922 - 1.000) |
|  | 6 to 12 | Total | 840 | 366 | 126 | 6 | 342 | 0.983 (0.963 - 0.994) | 0.744 (0.703 - 0.782) | 0.843 (0.816 - 0.867) | 0.954 (0.938 - 0.970) |  | 0.731 (0.688 - 0.770) | 0.984 (0.965 - 0.994) |
|  |  | Female | 329 | 138 | 35 | 2 | 154 | 0.987 (0.954 - 0.998) | 0.798 (0.730 - 0.855) | 0.888 (0.848 - 0.920) | 0.979 (0.962 - 0.995) |  | 0.815 (0.752 - 0.867) | 0.986 (0.949 - 0.998) |
|  |  | Male | 511 | 228 | 91 | 4 | 188 | 0.979 (0.948 - 0.994) | 0.715 (0.662 - 0.764) | 0.814 (0.778 - 0.847) | 0.931 (0.905 - 0.957) |  | 0.674 (0.615 - 0.729) | 0.983 (0.956 - 0.995) |
|  |  | Asian | 34 | 14 | 4 | 1 | 15 | 0.938 (0.698 - 0.998) | 0.778 (0.524 - 0.936) | 0.853 (0.689 - 0.950) | 0.917 (0.814 - 1.000) |  | 0.789 (0.544 - 0.939) | 0.933 (0.681 - 0.998) |
|  |  | Black | 95 | 34 | 19 | 0 | 42 | 1.000 (0.916 - 1.000) | 0.642 (0.498 - 0.769) | 0.800 (0.705 - 0.875) | 0.942 (0.890 - 0.994) |  | 0.689 (0.557 - 0.801) | 1.000 (0.897 - 1.000) |
|  |  | Hispanic/Latino | 254 | 143 | 26 | 1 | 84 | 0.988 (0.936 - 1.000) | 0.846 (0.783 - 0.897) | 0.894 (0.849 - 0.929) | 0.981 (0.961 - 1.000) |  | 0.764 (0.673 - 0.839) | 0.993 (0.962 - 1.000) |
|  |  | White | 370 | 130 | 62 | 3 | 175 | 0.983 (0.952 - 0.997) | 0.677 (0.606 - 0.743) | 0.824 (0.782 - 0.862) | 0.936 (0.909 - 0.962) |  | 0.738 (0.678 - 0.793) | 0.977 (0.935 - 0.995) |
|  |  | Other Race/Ethnicity | 78 | 42 | 12 | 1 | 23 | 0.958 (0.789 - 0.999) | 0.778 (0.644 - 0.880) | 0.833 (0.732 - 0.908) | 0.954 (0.895 - 1.000) |  | 0.657 (0.478 - 0.809) | 0.977 (0.877 - 0.999) |
|  |  | Asian, Female | 16 | 4 | 2 | 0 | 10 | 1.000 (0.692 - 1.000) | 0.667 (0.223 - 0.957) | 0.875 (0.617 - 0.984) | 0.967 (0.879 - 1.000) |  | 0.833 (0.516 - 0.979) | 1.000 (0.398 - 1.000) |
|  |  | Asian, Male | 18 | 10 | 2 | 1 | 5 | 0.833 (0.359 - 0.996) | 0.833 (0.516 - 0.979) | 0.833 (0.586 - 0.964) | 0.833 (0.610 - 1.000) |  | 0.714 (0.290 - 0.963) | 0.909 (0.587 - 0.998) |
|  |  | Black, Female | 34 | 14 | 7 | 0 | 13 | 1.000 (0.753 - 1.000) | 0.667 (0.430 - 0.854) | 0.794 (0.621 - 0.913) | 0.967 (0.896 - 1.000) |  | 0.650 (0.408 - 0.846) | 1.000 (0.768 - 1.000) |
|  |  | Black, Male | 61 | 20 | 12 | 0 | 29 | 1.000 (0.881 - 1.000) | 0.625 (0.437 - 0.789) | 0.803 (0.682 - 0.894) | 0.921 (0.848 - 0.995) |  | 0.707 (0.545 - 0.839) | 1.000 (0.832 - 1.000) |
|  |  | Hispanic, Female | 110 | 57 | 5 | 0 | 48 | 1.000 (0.926 - 1.000) | 0.919 (0.822 - 0.973) | 0.955 (0.897 - 0.985) | 0.997 (0.987 - 1.000) |  | 0.906 (0.793 - 0.969) | 1.000 (0.937 - 1.000) |
|  |  | Hispanic, Male | 144 | 86 | 21 | 1 | 36 | 0.973 (0.858 - 0.999) | 0.804 (0.716 - 0.874) | 0.847 (0.778 - 0.902) | 0.956 (0.909 - 1.000) |  | 0.632 (0.493 - 0.756) | 0.989 (0.938 - 1.000) |
|  |  | White, Female | 137 | 49 | 17 | 1 | 70 | 0.986 (0.924 - 1.000) | 0.742 (0.620 - 0.842) | 0.869 (0.800 - 0.920) | 0.966 (0.935 - 0.997) |  | 0.805 (0.706 - 0.882) | 0.980 (0.894 - 0.999) |
|  |  | White, Male | 233 | 81 | 45 | 2 | 105 | 0.981 (0.934 - 0.998) | 0.643 (0.553 - 0.726) | 0.798 (0.741 - 0.848) | 0.912 (0.872 - 0.952) |  | 0.700 (0.620 - 0.772) | 0.976 (0.916 - 0.997) |
|  |  | Other Race/Ethnicity, Female | 30 | 14 | 3 | 1 | 12 | 0.923 (0.640 - 0.998) | 0.824 (0.566 - 0.962) | 0.867 (0.693 - 0.962) | 0.959 (0.880 - 1.000) |  | 0.800 (0.519 - 0.957) | 0.933 (0.681 - 0.998) |
|  |  | Other Race/Ethnicity, Male | 48 | 28 | 9 | 0 | 11 | 1.000 (0.715 - 1.000) | 0.757 (0.588 - 0.882) | 0.813 (0.674 - 0.911) | 0.958 (0.873 - 1.000) |  | 0.550 (0.315 - 0.769) | 1.000 (0.877 - 1.000) |
|  | 13 to 17 | Total | 2262 | 962 | 276 | 20 | 1004 | 0.980 (0.970 - 0.988) | 0.777 (0.753 - 0.800) | 0.869 (0.855 - 0.883) | 0.969 (0.961 - 0.976) |  | 0.784 (0.761 - 0.807) | 0.980 (0.969 - 0.988) |
|  |  | Female | 1235 | 495 | 116 | 11 | 613 | 0.982 (0.969 - 0.991) | 0.810 (0.777 - 0.841) | 0.897 (0.879 - 0.914) | 0.976 (0.967 - 0.984) |  | 0.841 (0.812 - 0.867) | 0.978 (0.961 - 0.989) |
|  |  | Male | 1027 | 467 | 160 | 9 | 391 | 0.978 (0.958 - 0.990) | 0.745 (0.709 - 0.779) | 0.835 (0.811 - 0.858) | 0.960 (0.946 - 0.973) |  | 0.710 (0.670 - 0.747) | 0.981 (0.964 - 0.991) |
|  |  | Asian | 103 | 39 | 11 | 0 | 53 | 1.000 (0.933 - 1.000) | 0.780 (0.640 - 0.885) | 0.893 (0.817 - 0.945) | 0.991 (0.973 - 1.000) |  | 0.828 (0.713 - 0.911) | 1.000 (0.910 - 1.000) |
|  |  | Black | 193 | 92 | 28 | 4 | 69 | 0.945 (0.866 - 0.985) | 0.767 (0.681 - 0.839) | 0.834 (0.774 - 0.884) | 0.959 (0.927 - 0.992) |  | 0.711 (0.610 - 0.799) | 0.958 (0.897 - 0.989) |
|  |  | Hispanic/Latino | 573 | 275 | 59 | 9 | 230 | 0.962 (0.930 - 0.983) | 0.823 (0.778 - 0.863) | 0.881 (0.852 - 0.907) | 0.965 (0.949 - 0.982) |  | 0.796 (0.745 - 0.841) | 0.968 (0.941 - 0.985) |
|  |  | White | 1178 | 457 | 152 | 6 | 563 | 0.989 (0.977 - 0.996) | 0.750 (0.714 - 0.784) | 0.866 (0.845 - 0.885) | 0.968 (0.958 - 0.979) |  | 0.787 (0.756 - 0.817) | 0.987 (0.972 - 0.995) |
|  |  | Other Race/Ethnicity | 179 | 85 | 23 | 1 | 70 | 0.986 (0.924 - 1.000) | 0.787 (0.698 - 0.860) | 0.866 (0.807 - 0.912) | 0.977 (0.951 - 1.000) |  | 0.753 (0.652 - 0.836) | 0.988 (0.937 - 1.000) |
|  |  | Asian, Female | 63 | 27 | 4 | 0 | 32 | 1.000 (0.891 - 1.000) | 0.871 (0.702 - 0.964) | 0.937 (0.845 - 0.982) | 0.993 (0.972 - 1.000) |  | 0.889 (0.739 - 0.969) | 1.000 (0.872 - 1.000) |
|  |  | Asian, Male | 40 | 12 | 7 | 0 | 21 | 1.000 (0.839 - 1.000) | 0.632 (0.384 - 0.837) | 0.825 (0.672 - 0.927) | 0.997 (0.982 - 1.000) |  | 0.750 (0.551 - 0.893) | 1.000 (0.735 - 1.000) |
|  |  | Black, Female | 113 | 49 | 13 | 4 | 47 | 0.922 (0.811 - 0.978) | 0.790 (0.668 - 0.883) | 0.850 (0.770 - 0.910) | 0.958 (0.918 - 0.998) |  | 0.783 (0.658 - 0.879) | 0.925 (0.818 - 0.979) |
|  |  | Black, Male | 80 | 43 | 15 | 0 | 22 | 1.000 (0.846 - 1.000) | 0.741 (0.610 - 0.847) | 0.813 (0.710 - 0.891) | 0.971 (0.921 - 1.000) |  | 0.595 (0.421 - 0.752) | 1.000 (0.918 - 1.000) |
|  |  | Hispanic, Female | 311 | 130 | 27 | 4 | 150 | 0.974 (0.935 - 0.993) | 0.828 (0.760 - 0.884) | 0.900 (0.862 - 0.931) | 0.973 (0.955 - 0.992) |  | 0.847 (0.786 - 0.897) | 0.970 (0.925 - 0.992) |
|  |  | Hispanic, Male | 262 | 145 | 32 | 5 | 80 | 0.941 (0.868 - 0.981) | 0.819 (0.754 - 0.873) | 0.859 (0.811 - 0.899) | 0.953 (0.920 - 0.985) |  | 0.714 (0.621 - 0.796) | 0.967 (0.924 - 0.989) |
|  |  | White, Female | 618 | 235 | 58 | 3 | 322 | 0.991 (0.973 - 0.998) | 0.802 (0.752 - 0.846) | 0.901 (0.875 - 0.924) | 0.977 (0.965 - 0.989) |  | 0.847 (0.807 - 0.882) | 0.987 (0.964 - 0.997) |
|  |  | White, Male | 560 | 222 | 94 | 3 | 241 | 0.988 (0.964 - 0.997) | 0.703 (0.649 - 0.752) | 0.827 (0.793 - 0.857) | 0.957 (0.938 - 0.975) |  | 0.719 (0.668 - 0.767) | 0.987 (0.962 - 0.997) |
|  |  | Other Race/Ethnicity, Female | 105 | 45 | 11 | 0 | 49 | 1.000 (0.927 - 1.000) | 0.804 (0.676 - 0.898) | 0.895 (0.820 - 0.947) | 0.984 (0.959 - 1.000) |  | 0.817 (0.696 - 0.905) | 1.000 (0.921 - 1.000) |
|  |  | Other Race/Ethnicity, Male | 74 | 40 | 12 | 1 | 21 | 0.955 (0.772 - 0.999) | 0.769 (0.632 - 0.875) | 0.824 (0.718 - 0.903) | 0.959 (0.899 - 1.000) |  | 0.636 (0.451 - 0.796) | 0.976 (0.871 - 0.999) |
|  |  |  |  |  |  |  |  |  |  |  |  |  |  |  |
| NLP-gen | 6 to 17 | Total | 3102 | 1341 | 389 | 27 | 1345 | 0.980 (0.971 - 0.987) | 0.775 (0.755 - 0.795) | 0.866 (0.853 - 0.878) | 0.956 (0.948 - 0.964) |  | 0.776 (0.755 - 0.795) | 0.980 (0.971 - 0.987) |
|  |  | Female | 1564 | 631 | 153 | 12 | 768 | 0.985 (0.973 - 0.992) | 0.805 (0.775 - 0.832) | 0.895 (0.878 - 0.909) | 0.968 (0.959 - 0.977) |  | 0.834 (0.808 - 0.857) | 0.981 (0.968 - 0.990) |
|  |  | Male | 1538 | 710 | 236 | 15 | 577 | 0.975 (0.959 - 0.986) | 0.751 (0.722 - 0.778) | 0.837 (0.817 - 0.855) | 0.942 (0.928 - 0.956) |  | 0.710 (0.677 - 0.741) | 0.979 (0.966 - 0.988) |
|  |  | Asian | 137 | 54 | 14 | 1 | 68 | 0.986 (0.922 - 1.000) | 0.794 (0.679 - 0.883) | 0.891 (0.826 - 0.937) | 0.972 (0.944 - 1.000) |  | 0.829 (0.730 - 0.903) | 0.982 (0.903 - 1.000) |
|  |  | Black | 288 | 127 | 46 | 1 | 114 | 0.991 (0.953 - 1.000) | 0.734 (0.662 - 0.798) | 0.837 (0.789 - 0.878) | 0.935 (0.902 - 0.967) |  | 0.713 (0.636 - 0.781) | 0.992 (0.957 - 1.000) |
|  |  | Hispanic/Latino | 827 | 416 | 87 | 6 | 318 | 0.981 (0.960 - 0.993) | 0.827 (0.791 - 0.859) | 0.888 (0.864 - 0.908) | 0.966 (0.952 - 0.980) |  | 0.785 (0.742 - 0.824) | 0.986 (0.969 - 0.995) |
|  |  | White | 1548 | 595 | 206 | 19 | 728 | 0.975 (0.961 - 0.985) | 0.743 (0.711 - 0.773) | 0.855 (0.836 - 0.872) | 0.949 (0.938 - 0.961) |  | 0.779 (0.751 - 0.806) | 0.969 (0.952 - 0.981) |
|  |  | Other Race/Ethnicity | 257 | 134 | 28 | 0 | 95 | 1.000 (0.962 - 1.000) | 0.827 (0.760 - 0.882) | 0.891 (0.846 - 0.926) | 0.971 (0.947 - 0.995) |  | 0.772 (0.688 - 0.843) | 1.000 (0.973 - 1.000) |
|  |  | Asian, Female | 79 | 32 | 5 | 0 | 42 | 1.000 (0.916 - 1.000) | 0.865 (0.712 - 0.955) | 0.937 (0.858 - 0.979) | 0.981 (0.950 - 1.000) |  | 0.894 (0.769 - 0.965) | 1.000 (0.891 - 1.000) |
|  |  | Asian, Male | 58 | 22 | 9 | 1 | 26 | 0.963 (0.810 - 0.999) | 0.710 (0.520 - 0.858) | 0.828 (0.706 - 0.914) | 0.956 (0.899 - 1.000) |  | 0.743 (0.567 - 0.875) | 0.957 (0.781 - 0.999) |
|  |  | Black, Female | 147 | 62 | 21 | 1 | 63 | 0.984 (0.916 - 1.000) | 0.747 (0.640 - 0.836) | 0.850 (0.782 - 0.904) | 0.937 (0.893 - 0.980) |  | 0.750 (0.644 - 0.838) | 0.984 (0.915 - 1.000) |
|  |  | Black, Male | 141 | 65 | 25 | 0 | 51 | 1.000 (0.930 - 1.000) | 0.722 (0.618 - 0.811) | 0.823 (0.749 - 0.882) | 0.930 (0.879 - 0.981) |  | 0.671 (0.554 - 0.775) | 1.000 (0.945 - 1.000) |
|  |  | Hispanic, Female | 421 | 186 | 33 | 3 | 199 | 0.985 (0.957 - 0.997) | 0.849 (0.795 - 0.894) | 0.914 (0.884 - 0.939) | 0.981 (0.968 - 0.995) |  | 0.858 (0.806 - 0.900) | 0.984 (0.954 - 0.997) |
|  |  | Hispanic, Male | 406 | 230 | 54 | 3 | 119 | 0.975 (0.930 - 0.995) | 0.810 (0.759 - 0.854) | 0.860 (0.822 - 0.892) | 0.951 (0.923 - 0.978) |  | 0.688 (0.613 - 0.756) | 0.987 (0.963 - 0.997) |
|  |  | White, Female | 755 | 281 | 78 | 8 | 388 | 0.980 (0.961 - 0.991) | 0.783 (0.736 - 0.824) | 0.886 (0.861 - 0.908) | 0.963 (0.949 - 0.977) |  | 0.833 (0.796 - 0.865) | 0.972 (0.946 - 0.988) |
|  |  | White, Male | 793 | 314 | 128 | 11 | 340 | 0.969 (0.945 - 0.984) | 0.710 (0.666 - 0.752) | 0.825 (0.796 - 0.851) | 0.932 (0.913 - 0.952) |  | 0.726 (0.684 - 0.766) | 0.966 (0.940 - 0.983) |
|  |  | Other Race/Ethnicity, Female | 135 | 61 | 12 | 0 | 62 | 1.000 (0.942 - 1.000) | 0.836 (0.730 - 0.912) | 0.911 (0.850 - 0.953) | 0.975 (0.948 - 1.000) |  | 0.838 (0.734 - 0.913) | 1.000 (0.941 - 1.000) |
|  |  | Other Race/Ethnicity, Male | 122 | 73 | 16 | 0 | 33 | 1.000 (0.894 - 1.000) | 0.820 (0.725 - 0.894) | 0.869 (0.796 - 0.923) | 0.966 (0.922 - 1.000) |  | 0.673 (0.525 - 0.801) | 1.000 (0.951 - 1.000) |
|  | 6 to 12 | Total | 840 | 384 | 108 | 9 | 339 | 0.974 (0.951 - 0.988) | 0.780 (0.741 - 0.816) | 0.861 (0.835 - 0.883) | 0.958 (0.943 - 0.973) |  | 0.758 (0.716 - 0.797) | 0.977 (0.957 - 0.989) |
|  |  | Female | 329 | 143 | 30 | 2 | 154 | 0.987 (0.954 - 0.998) | 0.827 (0.762 - 0.880) | 0.903 (0.865 - 0.933) | 0.983 (0.969 - 0.998) |  | 0.837 (0.775 - 0.887) | 0.986 (0.951 - 0.998) |
|  |  | Male | 511 | 241 | 78 | 7 | 185 | 0.964 (0.926 - 0.985) | 0.755 (0.705 - 0.802) | 0.834 (0.798 - 0.865) | 0.938 (0.913 - 0.962) |  | 0.703 (0.644 - 0.758) | 0.972 (0.943 - 0.989) |
|  |  | Asian | 34 | 14 | 4 | 1 | 15 | 0.938 (0.698 - 0.998) | 0.778 (0.524 - 0.936) | 0.853 (0.689 - 0.950) | 0.920 (0.820 - 1.000) |  | 0.789 (0.544 - 0.939) | 0.933 (0.681 - 0.998) |
|  |  | Black | 95 | 39 | 14 | 0 | 42 | 1.000 (0.916 - 1.000) | 0.736 (0.597 - 0.847) | 0.853 (0.765 - 0.917) | 0.943 (0.892 - 0.995) |  | 0.750 (0.616 - 0.856) | 1.000 (0.910 - 1.000) |
|  |  | Hispanic/Latino | 254 | 146 | 23 | 1 | 84 | 0.988 (0.936 - 1.000) | 0.864 (0.803 - 0.912) | 0.906 (0.863 - 0.939) | 0.987 (0.971 - 1.000) |  | 0.785 (0.695 - 0.859) | 0.993 (0.963 - 1.000) |
|  |  | White | 370 | 136 | 56 | 7 | 171 | 0.961 (0.921 - 0.984) | 0.708 (0.639 - 0.772) | 0.830 (0.787 - 0.867) | 0.937 (0.910 - 0.963) |  | 0.753 (0.692 - 0.808) | 0.951 (0.902 - 0.980) |
|  |  | Other Race/Ethnicity | 78 | 47 | 7 | 0 | 24 | 1.000 (0.858 - 1.000) | 0.870 (0.751 - 0.946) | 0.910 (0.824 - 0.963) | 0.975 (0.929 - 1.000) |  | 0.774 (0.589 - 0.904) | 1.000 (0.925 - 1.000) |
|  |  | Asian, Female | 16 | 5 | 1 | 0 | 10 | 1.000 (0.692 - 1.000) | 0.833 (0.359 - 0.996) | 0.938 (0.698 - 0.998) | 0.933 (0.807 - 1.000) |  | 0.909 (0.587 - 0.998) | 1.000 (0.478 - 1.000) |
|  |  | Asian, Male | 18 | 9 | 3 | 1 | 5 | 0.833 (0.359 - 0.996) | 0.750 (0.428 - 0.945) | 0.778 (0.524 - 0.936) | 0.875 (0.677 - 1.000) |  | 0.625 (0.245 - 0.915) | 0.900 (0.555 - 0.997) |
|  |  | Black, Female | 34 | 17 | 4 | 0 | 13 | 1.000 (0.753 - 1.000) | 0.810 (0.581 - 0.946) | 0.882 (0.725 - 0.967) | 0.960 (0.881 - 1.000) |  | 0.765 (0.501 - 0.932) | 1.000 (0.805 - 1.000) |
|  |  | Black, Male | 61 | 22 | 10 | 0 | 29 | 1.000 (0.881 - 1.000) | 0.688 (0.500 - 0.839) | 0.836 (0.719 - 0.918) | 0.934 (0.867 - 1.000) |  | 0.744 (0.579 - 0.870) | 1.000 (0.846 - 1.000) |
|  |  | Hispanic, Female | 110 | 57 | 5 | 0 | 48 | 1.000 (0.926 - 1.000) | 0.919 (0.822 - 0.973) | 0.955 (0.897 - 0.985) | 0.998 (0.988 - 1.000) |  | 0.906 (0.793 - 0.969) | 1.000 (0.937 - 1.000) |
|  |  | Hispanic, Male | 144 | 89 | 18 | 1 | 36 | 0.973 (0.858 - 0.999) | 0.832 (0.747 - 0.897) | 0.868 (0.802 - 0.919) | 0.972 (0.934 - 1.000) |  | 0.667 (0.525 - 0.789) | 0.989 (0.940 - 1.000) |
|  |  | White, Female | 137 | 49 | 17 | 2 | 69 | 0.972 (0.902 - 0.997) | 0.742 (0.620 - 0.842) | 0.861 (0.792 - 0.914) | 0.974 (0.948 - 1.000) |  | 0.802 (0.702 - 0.880) | 0.961 (0.865 - 0.995) |
|  |  | White, Male | 233 | 87 | 39 | 5 | 102 | 0.953 (0.894 - 0.985) | 0.690 (0.602 - 0.770) | 0.811 (0.755 - 0.859) | 0.911 (0.871 - 0.951) |  | 0.723 (0.642 - 0.795) | 0.946 (0.878 - 0.982) |
|  |  | Other Race/Ethnicity, Female | 30 | 15 | 2 | 0 | 13 | 1.000 (0.753 - 1.000) | 0.882 (0.636 - 0.985) | 0.933 (0.779 - 0.992) | 0.986 (0.940 - 1.000) |  | 0.867 (0.595 - 0.983) | 1.000 (0.782 - 1.000) |
|  |  | Other Race/Ethnicity, Male | 48 | 32 | 5 | 0 | 11 | 1.000 (0.715 - 1.000) | 0.865 (0.712 - 0.955) | 0.896 (0.773 - 0.965) | 0.968 (0.894 - 1.000) |  | 0.688 (0.413 - 0.890) | 1.000 (0.891 - 1.000) |
|  | 13 to 17 | Total | 2262 | 957 | 281 | 18 | 1006 | 0.982 (0.972 - 0.990) | 0.773 (0.749 - 0.796) | 0.868 (0.853 - 0.882) | 0.955 (0.946 - 0.964) |  | 0.782 (0.758 - 0.804) | 0.982 (0.971 - 0.989) |
|  |  | Female | 1235 | 488 | 123 | 10 | 614 | 0.984 (0.971 - 0.992) | 0.799 (0.765 - 0.830) | 0.892 (0.874 - 0.909) | 0.963 (0.953 - 0.974) |  | 0.833 (0.804 - 0.859) | 0.980 (0.963 - 0.990) |
|  |  | Male | 1027 | 469 | 158 | 8 | 392 | 0.980 (0.961 - 0.991) | 0.748 (0.712 - 0.782) | 0.838 (0.814 - 0.860) | 0.944 (0.928 - 0.961) |  | 0.713 (0.673 - 0.750) | 0.983 (0.967 - 0.993) |
|  |  | Asian | 103 | 40 | 10 | 0 | 53 | 1.000 (0.933 - 1.000) | 0.800 (0.663 - 0.900) | 0.903 (0.829 - 0.952) | 0.986 (0.963 - 1.000) |  | 0.841 (0.727 - 0.921) | 1.000 (0.912 - 1.000) |
|  |  | Black | 193 | 88 | 32 | 1 | 72 | 0.986 (0.926 - 1.000) | 0.733 (0.645 - 0.810) | 0.829 (0.768 - 0.879) | 0.932 (0.890 - 0.974) |  | 0.692 (0.594 - 0.779) | 0.989 (0.939 - 1.000) |
|  |  | Hispanic/Latino | 573 | 270 | 64 | 5 | 234 | 0.979 (0.952 - 0.993) | 0.808 (0.762 - 0.849) | 0.880 (0.850 - 0.905) | 0.956 (0.937 - 0.975) |  | 0.785 (0.734 - 0.830) | 0.982 (0.958 - 0.994) |
|  |  | White | 1178 | 459 | 150 | 12 | 557 | 0.979 (0.963 - 0.989) | 0.754 (0.717 - 0.787) | 0.862 (0.841 - 0.882) | 0.952 (0.940 - 0.965) |  | 0.788 (0.756 - 0.817) | 0.975 (0.956 - 0.987) |
|  |  | Other Race/Ethnicity | 179 | 87 | 21 | 0 | 71 | 1.000 (0.949 - 1.000) | 0.806 (0.718 - 0.875) | 0.883 (0.826 - 0.926) | 0.970 (0.942 - 0.999) |  | 0.772 (0.672 - 0.853) | 1.000 (0.958 - 1.000) |
|  |  | Asian, Female | 63 | 27 | 4 | 0 | 32 | 1.000 (0.891 - 1.000) | 0.871 (0.702 - 0.964) | 0.937 (0.845 - 0.982) | 0.990 (0.965 - 1.000) |  | 0.889 (0.739 - 0.969) | 1.000 (0.872 - 1.000) |
|  |  | Asian, Male | 40 | 13 | 6 | 0 | 21 | 1.000 (0.839 - 1.000) | 0.684 (0.434 - 0.874) | 0.850 (0.702 - 0.943) | 0.980 (0.936 - 1.000) |  | 0.778 (0.577 - 0.914) | 1.000 (0.753 - 1.000) |
|  |  | Black, Female | 113 | 45 | 17 | 1 | 50 | 0.980 (0.896 - 1.000) | 0.726 (0.598 - 0.831) | 0.841 (0.760 - 0.903) | 0.928 (0.876 - 0.980) |  | 0.746 (0.625 - 0.845) | 0.978 (0.885 - 0.999) |
|  |  | Black, Male | 80 | 43 | 15 | 0 | 22 | 1.000 (0.846 - 1.000) | 0.741 (0.610 - 0.847) | 0.813 (0.710 - 0.891) | 0.931 (0.854 - 1.000) |  | 0.595 (0.421 - 0.752) | 1.000 (0.918 - 1.000) |
|  |  | Hispanic, Female | 311 | 129 | 28 | 3 | 151 | 0.981 (0.944 - 0.996) | 0.822 (0.753 - 0.878) | 0.900 (0.862 - 0.931) | 0.973 (0.954 - 0.991) |  | 0.844 (0.782 - 0.893) | 0.977 (0.935 - 0.995) |
|  |  | Hispanic, Male | 262 | 141 | 36 | 2 | 83 | 0.976 (0.918 - 0.997) | 0.797 (0.730 - 0.853) | 0.855 (0.806 - 0.895) | 0.939 (0.902 - 0.975) |  | 0.697 (0.607 - 0.778) | 0.986 (0.950 - 0.998) |
|  |  | White, Female | 618 | 232 | 61 | 6 | 319 | 0.982 (0.960 - 0.993) | 0.792 (0.741 - 0.837) | 0.892 (0.864 - 0.915) | 0.960 (0.944 - 0.976) |  | 0.839 (0.799 - 0.875) | 0.975 (0.946 - 0.991) |
|  |  | White, Male | 560 | 227 | 89 | 6 | 238 | 0.975 (0.947 - 0.991) | 0.718 (0.665 - 0.767) | 0.830 (0.797 - 0.861) | 0.941 (0.920 - 0.963) |  | 0.728 (0.676 - 0.775) | 0.974 (0.945 - 0.990) |
|  |  | Other Race/Ethnicity, Female | 105 | 46 | 10 | 0 | 49 | 1.000 (0.927 - 1.000) | 0.821 (0.696 - 0.911) | 0.905 (0.832 - 0.953) | 0.971 (0.937 - 1.000) |  | 0.831 (0.710 - 0.916) | 1.000 (0.923 - 1.000) |
|  |  | Other Race/Ethnicity, Male | 74 | 41 | 11 | 0 | 22 | 1.000 (0.846 - 1.000) | 0.788 (0.653 - 0.889) | 0.851 (0.750 - 0.923) | 0.965 (0.910 - 1.000) |  | 0.667 (0.482 - 0.820) | 1.000 (0.914 - 1.000) |
|  |  |  |  |  |  |  |  |  |  |  |  |  |  |  |
| NLP-med | 6 to 17 | Total | 3102 | 1399 | 331 | 37 | 1335 | 0.973 (0.963 - 0.981) | 0.809 (0.789 - 0.827) | 0.881 (0.869 - 0.893) | 0.970 (0.964 - 0.977) |  | 0.801 (0.781 - 0.820) | 0.974 (0.965 - 0.982) |
|  |  | Female | 1564 | 659 | 125 | 16 | 764 | 0.979 (0.967 - 0.988) | 0.841 (0.813 - 0.866) | 0.910 (0.895 - 0.924) | 0.978 (0.971 - 0.986) |  | 0.859 (0.835 - 0.882) | 0.976 (0.962 - 0.986) |
|  |  | Male | 1538 | 740 | 206 | 21 | 571 | 0.965 (0.946 - 0.978) | 0.782 (0.755 - 0.808) | 0.852 (0.834 - 0.870) | 0.959 (0.948 - 0.971) |  | 0.735 (0.702 - 0.766) | 0.972 (0.958 - 0.983) |
|  |  | Asian | 137 | 56 | 12 | 2 | 67 | 0.971 (0.899 - 0.996) | 0.824 (0.712 - 0.905) | 0.898 (0.834 - 0.943) | 0.966 (0.935 - 0.997) |  | 0.848 (0.750 - 0.919) | 0.966 (0.881 - 0.996) |
|  |  | Black | 288 | 132 | 41 | 3 | 112 | 0.974 (0.926 - 0.995) | 0.763 (0.693 - 0.824) | 0.847 (0.800 - 0.887) | 0.962 (0.937 - 0.987) |  | 0.732 (0.655 - 0.800) | 0.978 (0.936 - 0.995) |
|  |  | Hispanic/Latino | 827 | 432 | 71 | 10 | 314 | 0.969 (0.944 - 0.985) | 0.859 (0.825 - 0.888) | 0.902 (0.880 - 0.921) | 0.975 (0.963 - 0.987) |  | 0.816 (0.773 - 0.853) | 0.977 (0.959 - 0.989) |
|  |  | White | 1548 | 624 | 177 | 18 | 729 | 0.976 (0.962 - 0.986) | 0.779 (0.749 - 0.807) | 0.874 (0.856 - 0.890) | 0.967 (0.958 - 0.976) |  | 0.805 (0.777 - 0.830) | 0.972 (0.956 - 0.983) |
|  |  | Other Race/Ethnicity | 257 | 138 | 24 | 4 | 91 | 0.958 (0.896 - 0.988) | 0.852 (0.788 - 0.903) | 0.891 (0.846 - 0.926) | 0.979 (0.959 - 1.000) |  | 0.791 (0.706 - 0.861) | 0.972 (0.929 - 0.992) |
|  |  | Asian, Female | 79 | 35 | 2 | 1 | 41 | 0.976 (0.874 - 0.999) | 0.946 (0.818 - 0.993) | 0.962 (0.893 - 0.992) | 0.965 (0.923 - 1.000) |  | 0.953 (0.842 - 0.994) | 0.972 (0.855 - 0.999) |
|  |  | Asian, Male | 58 | 21 | 10 | 1 | 26 | 0.963 (0.810 - 0.999) | 0.677 (0.486 - 0.833) | 0.810 (0.686 - 0.901) | 0.964 (0.913 - 1.000) |  | 0.722 (0.548 - 0.858) | 0.955 (0.772 - 0.999) |
|  |  | Black, Female | 147 | 66 | 17 | 2 | 62 | 0.969 (0.892 - 0.996) | 0.795 (0.692 - 0.876) | 0.871 (0.806 - 0.920) | 0.964 (0.930 - 0.997) |  | 0.785 (0.678 - 0.869) | 0.971 (0.898 - 0.996) |
|  |  | Black, Male | 141 | 66 | 24 | 1 | 50 | 0.980 (0.896 - 1.000) | 0.733 (0.630 - 0.821) | 0.823 (0.749 - 0.882) | 0.963 (0.925 - 1.000) |  | 0.676 (0.557 - 0.780) | 0.985 (0.920 - 1.000) |
|  |  | Hispanic, Female | 421 | 191 | 28 | 3 | 199 | 0.985 (0.957 - 0.997) | 0.872 (0.821 - 0.913) | 0.926 (0.897 - 0.949) | 0.990 (0.980 - 1.000) |  | 0.877 (0.827 - 0.916) | 0.985 (0.955 - 0.997) |
|  |  | Hispanic, Male | 406 | 241 | 43 | 7 | 115 | 0.943 (0.885 - 0.977) | 0.849 (0.802 - 0.888) | 0.877 (0.841 - 0.907) | 0.954 (0.927 - 0.980) |  | 0.728 (0.651 - 0.796) | 0.972 (0.943 - 0.989) |
|  |  | White, Female | 755 | 294 | 65 | 7 | 389 | 0.982 (0.964 - 0.993) | 0.819 (0.775 - 0.857) | 0.905 (0.881 - 0.925) | 0.976 (0.966 - 0.987) |  | 0.857 (0.821 - 0.888) | 0.977 (0.953 - 0.991) |
|  |  | White, Male | 793 | 330 | 112 | 11 | 340 | 0.969 (0.945 - 0.984) | 0.747 (0.703 - 0.787) | 0.845 (0.818 - 0.869) | 0.957 (0.941 - 0.972) |  | 0.752 (0.710 - 0.791) | 0.968 (0.943 - 0.984) |
|  |  | Other Race/Ethnicity, Female | 135 | 63 | 10 | 3 | 59 | 0.952 (0.865 - 0.990) | 0.863 (0.762 - 0.932) | 0.904 (0.841 - 0.948) | 0.980 (0.954 - 1.000) |  | 0.855 (0.750 - 0.928) | 0.955 (0.873 - 0.991) |
|  |  | Other Race/Ethnicity, Male | 122 | 75 | 14 | 1 | 32 | 0.970 (0.842 - 0.999) | 0.843 (0.750 - 0.911) | 0.877 (0.805 - 0.930) | 0.979 (0.945 - 1.000) |  | 0.696 (0.542 - 0.823) | 0.987 (0.929 - 1.000) |
|  | 6 to 12 | Total | 840 | 393 | 99 | 12 | 336 | 0.966 (0.941 - 0.982) | 0.799 (0.761 - 0.833) | 0.868 (0.843 - 0.890) | 0.966 (0.952 - 0.979) |  | 0.772 (0.730 - 0.811) | 0.970 (0.949 - 0.985) |
|  |  | Female | 329 | 145 | 28 | 1 | 155 | 0.994 (0.965 - 1.000) | 0.838 (0.775 - 0.890) | 0.912 (0.876 - 0.940) | 0.983 (0.969 - 0.998) |  | 0.847 (0.787 - 0.896) | 0.993 (0.962 - 1.000) |
|  |  | Male | 511 | 248 | 71 | 11 | 181 | 0.943 (0.900 - 0.971) | 0.777 (0.728 - 0.822) | 0.840 (0.805 - 0.870) | 0.951 (0.929 - 0.973) |  | 0.718 (0.658 - 0.773) | 0.958 (0.925 - 0.979) |
|  |  | Asian | 34 | 14 | 4 | 1 | 15 | 0.938 (0.698 - 0.998) | 0.778 (0.524 - 0.936) | 0.853 (0.689 - 0.950) | 0.929 (0.834 - 1.000) |  | 0.789 (0.544 - 0.939) | 0.933 (0.681 - 0.998) |
|  |  | Black | 95 | 39 | 14 | 1 | 41 | 0.976 (0.874 - 0.999) | 0.736 (0.597 - 0.847) | 0.842 (0.753 - 0.909) | 0.967 (0.927 - 1.000) |  | 0.745 (0.610 - 0.853) | 0.975 (0.868 - 0.999) |
|  |  | Hispanic/Latino | 254 | 150 | 19 | 2 | 83 | 0.976 (0.918 - 0.997) | 0.888 (0.830 - 0.931) | 0.917 (0.876 - 0.948) | 0.985 (0.966 - 1.000) |  | 0.814 (0.724 - 0.884) | 0.987 (0.953 - 0.998) |
|  |  | White | 370 | 142 | 50 | 7 | 171 | 0.961 (0.921 - 0.984) | 0.740 (0.671 - 0.800) | 0.846 (0.805 - 0.881) | 0.953 (0.930 - 0.975) |  | 0.774 (0.713 - 0.827) | 0.953 (0.906 - 0.981) |
|  |  | Other Race/Ethnicity | 78 | 45 | 9 | 1 | 23 | 0.958 (0.789 - 0.999) | 0.833 (0.707 - 0.921) | 0.872 (0.777 - 0.937) | 0.978 (0.935 - 1.000) |  | 0.719 (0.533 - 0.863) | 0.978 (0.885 - 0.999) |
|  |  | Asian, Female | 16 | 5 | 1 | 0 | 10 | 1.000 (0.692 - 1.000) | 0.833 (0.359 - 0.996) | 0.938 (0.698 - 0.998) | 0.892 (0.730 - 1.000) |  | 0.909 (0.587 - 0.998) | 1.000 (0.478 - 1.000) |
|  |  | Asian, Male | 18 | 9 | 3 | 1 | 5 | 0.833 (0.359 - 0.996) | 0.750 (0.428 - 0.945) | 0.778 (0.524 - 0.936) | 0.917 (0.752 - 1.000) |  | 0.625 (0.245 - 0.915) | 0.900 (0.555 - 0.997) |
|  |  | Black, Female | 34 | 17 | 4 | 0 | 13 | 1.000 (0.753 - 1.000) | 0.810 (0.581 - 0.946) | 0.882 (0.725 - 0.967) | 0.993 (0.959 - 1.000) |  | 0.765 (0.501 - 0.932) | 1.000 (0.805 - 1.000) |
|  |  | Black, Male | 61 | 22 | 10 | 1 | 28 | 0.966 (0.822 - 0.999) | 0.688 (0.500 - 0.839) | 0.820 (0.700 - 0.906) | 0.949 (0.890 - 1.000) |  | 0.737 (0.569 - 0.866) | 0.957 (0.781 - 0.999) |
|  |  | Hispanic, Female | 110 | 58 | 4 | 0 | 48 | 1.000 (0.926 - 1.000) | 0.935 (0.843 - 0.982) | 0.964 (0.910 - 0.990) | 1.000 (1.000 - 1.000) |  | 0.923 (0.815 - 0.979) | 1.000 (0.938 - 1.000) |
|  |  | Hispanic, Male | 144 | 92 | 15 | 2 | 35 | 0.946 (0.818 - 0.993) | 0.860 (0.779 - 0.919) | 0.882 (0.818 - 0.930) | 0.962 (0.918 - 1.000) |  | 0.700 (0.554 - 0.821) | 0.979 (0.925 - 0.997) |
|  |  | White, Female | 137 | 51 | 15 | 1 | 70 | 0.986 (0.924 - 1.000) | 0.773 (0.653 - 0.867) | 0.883 (0.817 - 0.932) | 0.971 (0.943 - 1.000) |  | 0.824 (0.726 - 0.898) | 0.981 (0.897 - 1.000) |
|  |  | White, Male | 233 | 91 | 35 | 6 | 101 | 0.944 (0.882 - 0.979) | 0.722 (0.635 - 0.798) | 0.824 (0.769 - 0.871) | 0.941 (0.908 - 0.973) |  | 0.743 (0.661 - 0.814) | 0.938 (0.870 - 0.977) |
|  |  | Other Race/Ethnicity, Female | 30 | 14 | 3 | 0 | 13 | 1.000 (0.753 - 1.000) | 0.824 (0.566 - 0.962) | 0.900 (0.735 - 0.979) | 1.000 (1.000 - 1.000) |  | 0.813 (0.544 - 0.960) | 1.000 (0.768 - 1.000) |
|  |  | Other Race/Ethnicity, Male | 48 | 31 | 6 | 1 | 10 | 0.909 (0.587 - 0.998) | 0.838 (0.680 - 0.938) | 0.854 (0.722 - 0.939) | 0.966 (0.888 - 1.000) |  | 0.625 (0.354 - 0.848) | 0.969 (0.838 - 0.999) |
|  | 13 to 17 | Total | 2262 | 1006 | 232 | 25 | 999 | 0.976 (0.964 - 0.984) | 0.813 (0.790 - 0.834) | 0.886 (0.873 - 0.899) | 0.972 (0.964 - 0.979) |  | 0.812 (0.789 - 0.833) | 0.976 (0.964 - 0.984) |
|  |  | Female | 1235 | 514 | 97 | 15 | 609 | 0.976 (0.961 - 0.986) | 0.841 (0.810 - 0.869) | 0.909 (0.892 - 0.925) | 0.977 (0.969 - 0.986) |  | 0.863 (0.835 - 0.887) | 0.972 (0.954 - 0.984) |
|  |  | Male | 1027 | 492 | 135 | 10 | 390 | 0.975 (0.955 - 0.988) | 0.785 (0.750 - 0.816) | 0.859 (0.836 - 0.880) | 0.963 (0.950 - 0.977) |  | 0.743 (0.703 - 0.780) | 0.980 (0.964 - 0.990) |
|  |  | Asian | 103 | 42 | 8 | 1 | 52 | 0.981 (0.899 - 1.000) | 0.840 (0.709 - 0.928) | 0.913 (0.841 - 0.959) | 0.978 (0.950 - 1.000) |  | 0.867 (0.754 - 0.941) | 0.977 (0.877 - 0.999) |
|  |  | Black | 193 | 93 | 27 | 2 | 71 | 0.973 (0.905 - 0.997) | 0.775 (0.690 - 0.846) | 0.850 (0.791 - 0.897) | 0.961 (0.929 - 0.993) |  | 0.724 (0.625 - 0.810) | 0.979 (0.926 - 0.997) |
|  |  | Hispanic/Latino | 573 | 282 | 52 | 8 | 231 | 0.967 (0.935 - 0.985) | 0.844 (0.801 - 0.881) | 0.895 (0.867 - 0.919) | 0.971 (0.955 - 0.986) |  | 0.816 (0.766 - 0.860) | 0.972 (0.946 - 0.988) |
|  |  | White | 1178 | 482 | 127 | 11 | 558 | 0.981 (0.966 - 0.990) | 0.791 (0.757 - 0.823) | 0.883 (0.863 - 0.901) | 0.971 (0.961 - 0.981) |  | 0.815 (0.783 - 0.843) | 0.978 (0.960 - 0.989) |
|  |  | Other Race/Ethnicity | 179 | 93 | 15 | 3 | 68 | 0.958 (0.881 - 0.991) | 0.861 (0.781 - 0.920) | 0.899 (0.846 - 0.939) | 0.980 (0.957 - 1.000) |  | 0.819 (0.720 - 0.895) | 0.969 (0.911 - 0.994) |
|  |  | Asian, Female | 63 | 30 | 1 | 1 | 31 | 0.969 (0.838 - 0.999) | 0.968 (0.833 - 0.999) | 0.968 (0.890 - 0.996) | 0.976 (0.938 - 1.000) |  | 0.969 (0.838 - 0.999) | 0.968 (0.833 - 0.999) |
|  |  | Asian, Male | 40 | 12 | 7 | 0 | 21 | 1.000 (0.839 - 1.000) | 0.632 (0.384 - 0.837) | 0.825 (0.672 - 0.927) | 0.980 (0.936 - 1.000) |  | 0.750 (0.551 - 0.893) | 1.000 (0.735 - 1.000) |
|  |  | Black, Female | 113 | 49 | 13 | 2 | 49 | 0.961 (0.865 - 0.995) | 0.790 (0.668 - 0.883) | 0.867 (0.791 - 0.924) | 0.958 (0.917 - 0.998) |  | 0.790 (0.668 - 0.883) | 0.961 (0.865 - 0.995) |
|  |  | Black, Male | 80 | 44 | 14 | 0 | 22 | 1.000 (0.846 - 1.000) | 0.759 (0.628 - 0.861) | 0.825 (0.724 - 0.901) | 0.972 (0.922 - 1.000) |  | 0.611 (0.435 - 0.769) | 1.000 (0.920 - 1.000) |
|  |  | Hispanic, Female | 311 | 133 | 24 | 3 | 151 | 0.981 (0.944 - 0.996) | 0.847 (0.781 - 0.900) | 0.913 (0.876 - 0.942) | 0.985 (0.971 - 0.999) |  | 0.863 (0.803 - 0.910) | 0.978 (0.937 - 0.995) |
|  |  | Hispanic, Male | 262 | 149 | 28 | 5 | 80 | 0.941 (0.868 - 0.981) | 0.842 (0.780 - 0.892) | 0.874 (0.828 - 0.912) | 0.950 (0.917 - 0.983) |  | 0.741 (0.648 - 0.820) | 0.968 (0.926 - 0.989) |
|  |  | White, Female | 618 | 243 | 50 | 6 | 319 | 0.982 (0.960 - 0.993) | 0.829 (0.781 - 0.871) | 0.909 (0.884 - 0.931) | 0.977 (0.965 - 0.989) |  | 0.864 (0.825 - 0.898) | 0.976 (0.948 - 0.991) |
|  |  | White, Male | 560 | 239 | 77 | 5 | 239 | 0.980 (0.953 - 0.993) | 0.756 (0.705 - 0.803) | 0.854 (0.822 - 0.882) | 0.963 (0.946 - 0.980) |  | 0.756 (0.705 - 0.803) | 0.980 (0.953 - 0.993) |
|  |  | Other Race/Ethnicity, Female | 105 | 49 | 7 | 3 | 46 | 0.939 (0.831 - 0.987) | 0.875 (0.759 - 0.948) | 0.905 (0.832 - 0.953) | 0.976 (0.946 - 1.000) |  | 0.868 (0.747 - 0.945) | 0.942 (0.841 - 0.988) |
|  |  | Other Race/Ethnicity, Male | 74 | 44 | 8 | 0 | 22 | 1.000 (0.846 - 1.000) | 0.846 (0.719 - 0.931) | 0.892 (0.798 - 0.952) | 0.986 (0.951 - 1.000) |  | 0.733 (0.541 - 0.877) | 1.000 (0.920 - 1.000) |
|  |  |  |  |  |  |  |  |  |  |  |  |  |  |  |
| LLM | 6 to 17 | Total | 3102 | 1480 | 250 | 29 | 1343 | 0.979 (0.970 - 0.986) | 0.855 (0.838 - 0.872) | 0.910 (0.899 - 0.920) | 0.962 (0.955 - 0.969) |  | 0.843 (0.824 - 0.861) | 0.981 (0.973 - 0.987) |
|  |  | Female | 1564 | 682 | 102 | 10 | 770 | 0.987 (0.977 - 0.994) | 0.870 (0.844 - 0.893) | 0.928 (0.914 - 0.941) | 0.971 (0.962 - 0.979) |  | 0.883 (0.860 - 0.904) | 0.986 (0.974 - 0.993) |
|  |  | Male | 1538 | 798 | 148 | 19 | 573 | 0.968 (0.950 - 0.981) | 0.844 (0.819 - 0.866) | 0.891 (0.875 - 0.907) | 0.953 (0.941 - 0.965) |  | 0.795 (0.763 - 0.824) | 0.977 (0.964 - 0.986) |
|  |  | Asian | 137 | 60 | 8 | 0 | 69 | 1.000 (0.948 - 1.000) | 0.882 (0.781 - 0.948) | 0.942 (0.888 - 0.974) | 0.972 (0.944 - 1.000) |  | 0.896 (0.806 - 0.954) | 1.000 (0.940 - 1.000) |
|  |  | Black | 288 | 144 | 29 | 2 | 113 | 0.983 (0.939 - 0.998) | 0.832 (0.768 - 0.885) | 0.892 (0.851 - 0.926) | 0.947 (0.918 - 0.977) |  | 0.796 (0.720 - 0.859) | 0.986 (0.951 - 0.998) |
|  |  | Hispanic/Latino | 827 | 440 | 63 | 11 | 313 | 0.966 (0.940 - 0.983) | 0.875 (0.843 - 0.902) | 0.911 (0.889 - 0.929) | 0.961 (0.946 - 0.976) |  | 0.832 (0.791 - 0.869) | 0.976 (0.957 - 0.988) |
|  |  | White | 1548 | 675 | 126 | 16 | 731 | 0.979 (0.965 - 0.988) | 0.843 (0.816 - 0.867) | 0.908 (0.893 - 0.922) | 0.963 (0.953 - 0.973) |  | 0.853 (0.827 - 0.876) | 0.977 (0.963 - 0.987) |
|  |  | Other Race/Ethnicity | 257 | 142 | 20 | 0 | 95 | 1.000 (0.962 - 1.000) | 0.877 (0.816 - 0.923) | 0.922 (0.882 - 0.952) | 0.973 (0.949 - 0.996) |  | 0.826 (0.744 - 0.890) | 1.000 (0.974 - 1.000) |
|  |  | Asian, Female | 79 | 33 | 4 | 0 | 42 | 1.000 (0.916 - 1.000) | 0.892 (0.746 - 0.970) | 0.949 (0.875 - 0.986) | 0.972 (0.935 - 1.000) |  | 0.913 (0.792 - 0.976) | 1.000 (0.894 - 1.000) |
|  |  | Asian, Male | 58 | 27 | 4 | 0 | 27 | 1.000 (0.872 - 1.000) | 0.871 (0.702 - 0.964) | 0.931 (0.833 - 0.981) | 0.973 (0.929 - 1.000) |  | 0.871 (0.702 - 0.964) | 1.000 (0.872 - 1.000) |
|  |  | Black, Female | 147 | 69 | 14 | 2 | 62 | 0.969 (0.892 - 0.996) | 0.831 (0.733 - 0.905) | 0.891 (0.829 - 0.936) | 0.960 (0.925 - 0.995) |  | 0.816 (0.710 - 0.895) | 0.972 (0.902 - 0.997) |
|  |  | Black, Male | 141 | 75 | 15 | 0 | 51 | 1.000 (0.930 - 1.000) | 0.833 (0.740 - 0.904) | 0.894 (0.831 - 0.939) | 0.935 (0.886 - 0.984) |  | 0.773 (0.653 - 0.867) | 1.000 (0.952 - 1.000) |
|  |  | Hispanic, Female | 421 | 194 | 25 | 3 | 199 | 0.985 (0.957 - 0.997) | 0.886 (0.836 - 0.925) | 0.933 (0.905 - 0.955) | 0.975 (0.959 - 0.990) |  | 0.888 (0.840 - 0.926) | 0.985 (0.956 - 0.997) |
|  |  | Hispanic, Male | 406 | 246 | 38 | 8 | 114 | 0.934 (0.875 - 0.971) | 0.866 (0.821 - 0.904) | 0.887 (0.852 - 0.916) | 0.944 (0.915 - 0.973) |  | 0.750 (0.673 - 0.817) | 0.969 (0.939 - 0.986) |
|  |  | White, Female | 755 | 311 | 48 | 5 | 391 | 0.987 (0.971 - 0.996) | 0.866 (0.827 - 0.900) | 0.930 (0.909 - 0.947) | 0.970 (0.958 - 0.982) |  | 0.891 (0.858 - 0.918) | 0.984 (0.963 - 0.995) |
|  |  | White, Male | 793 | 364 | 78 | 11 | 340 | 0.969 (0.945 - 0.984) | 0.824 (0.785 - 0.858) | 0.888 (0.864 - 0.909) | 0.955 (0.940 - 0.971) |  | 0.813 (0.773 - 0.850) | 0.971 (0.948 - 0.985) |
|  |  | Other Race/Ethnicity, Female | 135 | 65 | 8 | 0 | 62 | 1.000 (0.942 - 1.000) | 0.890 (0.795 - 0.951) | 0.941 (0.887 - 0.974) | 0.972 (0.942 - 1.000) |  | 0.886 (0.787 - 0.949) | 1.000 (0.945 - 1.000) |
|  |  | Other Race/Ethnicity, Male | 122 | 77 | 12 | 0 | 33 | 1.000 (0.894 - 1.000) | 0.865 (0.776 - 0.928) | 0.902 (0.834 - 0.948) | 0.974 (0.935 - 1.000) |  | 0.733 (0.581 - 0.854) | 1.000 (0.953 - 1.000) |
|  | 6 to 12 | Total | 840 | 438 | 54 | 11 | 337 | 0.968 (0.944 - 0.984) | 0.890 (0.859 - 0.916) | 0.923 (0.902 - 0.940) | 0.967 (0.954 - 0.981) |  | 0.862 (0.824 - 0.895) | 0.976 (0.957 - 0.988) |
|  |  | Female | 329 | 161 | 12 | 2 | 154 | 0.987 (0.954 - 0.998) | 0.931 (0.882 - 0.964) | 0.957 (0.930 - 0.977) | 0.984 (0.969 - 0.998) |  | 0.928 (0.877 - 0.962) | 0.988 (0.956 - 0.999) |
|  |  | Male | 511 | 277 | 42 | 9 | 183 | 0.953 (0.913 - 0.978) | 0.868 (0.826 - 0.903) | 0.900 (0.871 - 0.925) | 0.955 (0.934 - 0.976) |  | 0.813 (0.756 - 0.862) | 0.969 (0.941 - 0.986) |
|  |  | Asian | 34 | 15 | 3 | 0 | 16 | 1.000 (0.794 - 1.000) | 0.833 (0.586 - 0.964) | 0.912 (0.763 - 0.981) | 0.948 (0.867 - 1.000) |  | 0.842 (0.604 - 0.966) | 1.000 (0.782 - 1.000) |
|  |  | Black | 95 | 44 | 9 | 0 | 42 | 1.000 (0.916 - 1.000) | 0.830 (0.702 - 0.919) | 0.905 (0.828 - 0.956) | 0.954 (0.907 - 1.000) |  | 0.824 (0.691 - 0.916) | 1.000 (0.920 - 1.000) |
|  |  | Hispanic/Latino | 254 | 156 | 13 | 3 | 82 | 0.965 (0.900 - 0.993) | 0.923 (0.872 - 0.958) | 0.937 (0.900 - 0.964) | 0.976 (0.953 - 0.999) |  | 0.863 (0.777 - 0.925) | 0.981 (0.946 - 0.996) |
|  |  | White | 370 | 168 | 24 | 8 | 170 | 0.955 (0.913 - 0.980) | 0.875 (0.820 - 0.918) | 0.914 (0.880 - 0.940) | 0.961 (0.940 - 0.981) |  | 0.876 (0.822 - 0.919) | 0.955 (0.912 - 0.980) |
|  |  | Other Race/Ethnicity | 78 | 50 | 4 | 0 | 24 | 1.000 (0.858 - 1.000) | 0.926 (0.821 - 0.979) | 0.949 (0.874 - 0.986) | 0.986 (0.953 - 1.000) |  | 0.857 (0.673 - 0.960) | 1.000 (0.929 - 1.000) |
|  |  | Asian, Female | 16 | 5 | 1 | 0 | 10 | 1.000 (0.692 - 1.000) | 0.833 (0.359 - 0.996) | 0.938 (0.698 - 0.998) | 0.883 (0.715 - 1.000) |  | 0.909 (0.587 - 0.998) | 1.000 (0.478 - 1.000) |
|  |  | Asian, Male | 18 | 10 | 2 | 0 | 6 | 1.000 (0.541 - 1.000) | 0.833 (0.516 - 0.979) | 0.889 (0.653 - 0.986) | 0.986 (0.917 - 1.000) |  | 0.750 (0.349 - 0.968) | 1.000 (0.692 - 1.000) |
|  |  | Black, Female | 34 | 19 | 2 | 0 | 13 | 1.000 (0.753 - 1.000) | 0.905 (0.696 - 0.988) | 0.941 (0.803 - 0.993) | 0.978 (0.920 - 1.000) |  | 0.867 (0.595 - 0.983) | 1.000 (0.824 - 1.000) |
|  |  | Black, Male | 61 | 25 | 7 | 0 | 29 | 1.000 (0.881 - 1.000) | 0.781 (0.600 - 0.907) | 0.885 (0.778 - 0.953) | 0.932 (0.863 - 1.000) |  | 0.806 (0.640 - 0.918) | 1.000 (0.863 - 1.000) |
|  |  | Hispanic, Female | 110 | 59 | 3 | 0 | 48 | 1.000 (0.926 - 1.000) | 0.952 (0.865 - 0.990) | 0.973 (0.922 - 0.994) | 0.998 (0.989 - 1.000) |  | 0.941 (0.838 - 0.988) | 1.000 (0.939 - 1.000) |
|  |  | Hispanic, Male | 144 | 97 | 10 | 3 | 34 | 0.919 (0.781 - 0.983) | 0.907 (0.835 - 0.954) | 0.910 (0.851 - 0.951) | 0.956 (0.908 - 1.000) |  | 0.773 (0.622 - 0.885) | 0.970 (0.915 - 0.994) |
|  |  | White, Female | 137 | 60 | 6 | 2 | 69 | 0.972 (0.902 - 0.997) | 0.909 (0.813 - 0.966) | 0.942 (0.888 - 0.974) | 0.973 (0.946 - 1.000) |  | 0.920 (0.834 - 0.970) | 0.968 (0.888 - 0.996) |
|  |  | White, Male | 233 | 108 | 18 | 6 | 101 | 0.944 (0.882 - 0.979) | 0.857 (0.784 - 0.913) | 0.897 (0.851 - 0.933) | 0.952 (0.923 - 0.982) |  | 0.849 (0.772 - 0.908) | 0.947 (0.889 - 0.980) |
|  |  | Other Race/Ethnicity, Female | 30 | 17 | 0 | 0 | 13 | 1.000 (0.753 - 1.000) | 1.000 (0.805 - 1.000) | 1.000 (0.884 - 1.000) | 1.000 (1.000 - 1.000) |  | 1.000 (0.753 - 1.000) | 1.000 (0.805 - 1.000) |
|  |  | Other Race/Ethnicity, Male | 48 | 33 | 4 | 0 | 11 | 1.000 (0.715 - 1.000) | 0.892 (0.746 - 0.970) | 0.917 (0.800 - 0.977) | 0.983 (0.928 - 1.000) |  | 0.733 (0.449 - 0.922) | 1.000 (0.894 - 1.000) |
|  | 13 to 17 | Total | 2262 | 1042 | 196 | 18 | 1006 | 0.982 (0.972 - 0.990) | 0.842 (0.820 - 0.862) | 0.905 (0.893 - 0.917) | 0.960 (0.952 - 0.969) |  | 0.837 (0.815 - 0.857) | 0.983 (0.973 - 0.990) |
|  |  | Female | 1235 | 521 | 90 | 8 | 616 | 0.987 (0.975 - 0.994) | 0.853 (0.822 - 0.880) | 0.921 (0.904 - 0.935) | 0.967 (0.957 - 0.977) |  | 0.873 (0.846 - 0.896) | 0.985 (0.970 - 0.993) |
|  |  | Male | 1027 | 521 | 106 | 10 | 390 | 0.975 (0.955 - 0.988) | 0.831 (0.799 - 0.859) | 0.887 (0.866 - 0.906) | 0.952 (0.937 - 0.967) |  | 0.786 (0.748 - 0.822) | 0.981 (0.966 - 0.991) |
|  |  | Asian | 103 | 45 | 5 | 0 | 53 | 1.000 (0.933 - 1.000) | 0.900 (0.782 - 0.967) | 0.951 (0.890 - 0.984) | 0.978 (0.950 - 1.000) |  | 0.914 (0.810 - 0.971) | 1.000 (0.921 - 1.000) |
|  |  | Black | 193 | 100 | 20 | 2 | 71 | 0.973 (0.905 - 0.997) | 0.833 (0.754 - 0.895) | 0.886 (0.833 - 0.927) | 0.943 (0.904 - 0.981) |  | 0.780 (0.681 - 0.860) | 0.980 (0.931 - 0.998) |
|  |  | Hispanic/Latino | 573 | 284 | 50 | 8 | 231 | 0.967 (0.935 - 0.985) | 0.850 (0.807 - 0.887) | 0.899 (0.871 - 0.922) | 0.955 (0.935 - 0.974) |  | 0.822 (0.772 - 0.865) | 0.973 (0.947 - 0.988) |
|  |  | White | 1178 | 507 | 102 | 8 | 561 | 0.986 (0.972 - 0.994) | 0.833 (0.800 - 0.861) | 0.907 (0.889 - 0.923) | 0.964 (0.953 - 0.975) |  | 0.846 (0.816 - 0.873) | 0.984 (0.970 - 0.993) |
|  |  | Other Race/Ethnicity | 179 | 92 | 16 | 0 | 71 | 1.000 (0.949 - 1.000) | 0.852 (0.771 - 0.913) | 0.911 (0.859 - 0.948) | 0.968 (0.938 - 0.997) |  | 0.816 (0.719 - 0.891) | 1.000 (0.961 - 1.000) |
|  |  | Asian, Female | 63 | 28 | 3 | 0 | 32 | 1.000 (0.891 - 1.000) | 0.903 (0.742 - 0.980) | 0.952 (0.867 - 0.990) | 0.984 (0.953 - 1.000) |  | 0.914 (0.769 - 0.982) | 1.000 (0.877 - 1.000) |
|  |  | Asian, Male | 40 | 17 | 2 | 0 | 21 | 1.000 (0.839 - 1.000) | 0.895 (0.669 - 0.987) | 0.950 (0.831 - 0.994) | 0.961 (0.900 - 1.000) |  | 0.913 (0.720 - 0.989) | 1.000 (0.805 - 1.000) |
|  |  | Black, Female | 113 | 50 | 12 | 2 | 49 | 0.961 (0.865 - 0.995) | 0.806 (0.686 - 0.896) | 0.876 (0.801 - 0.931) | 0.952 (0.910 - 0.995) |  | 0.803 (0.682 - 0.894) | 0.962 (0.868 - 0.995) |
|  |  | Black, Male | 80 | 50 | 8 | 0 | 22 | 1.000 (0.846 - 1.000) | 0.862 (0.746 - 0.939) | 0.900 (0.812 - 0.956) | 0.934 (0.859 - 1.000) |  | 0.733 (0.541 - 0.877) | 1.000 (0.929 - 1.000) |
|  |  | Hispanic, Female | 311 | 135 | 22 | 3 | 151 | 0.981 (0.944 - 0.996) | 0.860 (0.796 - 0.910) | 0.920 (0.884 - 0.947) | 0.964 (0.943 - 0.986) |  | 0.873 (0.814 - 0.919) | 0.978 (0.938 - 0.995) |
|  |  | Hispanic, Male | 262 | 149 | 28 | 5 | 80 | 0.941 (0.868 - 0.981) | 0.842 (0.780 - 0.892) | 0.874 (0.828 - 0.912) | 0.940 (0.904 - 0.976) |  | 0.741 (0.648 - 0.820) | 0.968 (0.926 - 0.989) |
|  |  | White, Female | 618 | 251 | 42 | 3 | 322 | 0.991 (0.973 - 0.998) | 0.857 (0.811 - 0.895) | 0.927 (0.904 - 0.946) | 0.969 (0.956 - 0.983) |  | 0.885 (0.847 - 0.916) | 0.988 (0.966 - 0.998) |
|  |  | White, Male | 560 | 256 | 60 | 5 | 239 | 0.980 (0.953 - 0.993) | 0.810 (0.762 - 0.852) | 0.884 (0.854 - 0.909) | 0.957 (0.938 - 0.975) |  | 0.799 (0.749 - 0.843) | 0.981 (0.956 - 0.994) |
|  |  | Other Race/Ethnicity, Female | 105 | 48 | 8 | 0 | 49 | 1.000 (0.927 - 1.000) | 0.857 (0.738 - 0.936) | 0.924 (0.855 - 0.967) | 0.968 (0.932 - 1.000) |  | 0.860 (0.742 - 0.937) | 1.000 (0.926 - 1.000) |
|  |  | Other Race/Ethnicity, Male | 74 | 44 | 8 | 0 | 22 | 1.000 (0.846 - 1.000) | 0.846 (0.719 - 0.931) | 0.892 (0.798 - 0.952) | 0.968 (0.915 - 1.000) |  | 0.733 (0.541 - 0.877) | 1.000 (0.920 - 1.000) |
|  |  |  |  |  |  |  |  |  |  |  |  |  |  |  |
| NLP-gen + aCS | 6 to 17 | Total | 3102 | 1361 | 369 | 20 | 1352 | 0.985 (0.978 - 0.991) | 0.787 (0.767 - 0.806) | 0.875 (0.862 - 0.886) | 0.973 (0.966 - 0.979) |  | 0.786 (0.765 - 0.805) | 0.986 (0.978 - 0.991) |
|  |  | Female | 1564 | 644 | 140 | 10 | 770 | 0.987 (0.977 - 0.994) | 0.821 (0.793 - 0.848) | 0.904 (0.888 - 0.918) | 0.980 (0.972 - 0.987) |  | 0.846 (0.821 - 0.869) | 0.985 (0.972 - 0.993) |
|  |  | Male | 1538 | 717 | 229 | 10 | 582 | 0.983 (0.969 - 0.992) | 0.758 (0.729 - 0.785) | 0.845 (0.826 - 0.862) | 0.963 (0.952 - 0.974) |  | 0.718 (0.685 - 0.748) | 0.986 (0.975 - 0.993) |
|  |  | Asian | 137 | 54 | 14 | 1 | 68 | 0.986 (0.922 - 1.000) | 0.794 (0.679 - 0.883) | 0.891 (0.826 - 0.937) | 0.982 (0.959 - 1.000) |  | 0.829 (0.730 - 0.903) | 0.982 (0.903 - 1.000) |
|  |  | Black | 288 | 130 | 43 | 1 | 114 | 0.991 (0.953 - 1.000) | 0.751 (0.680 - 0.814) | 0.847 (0.800 - 0.887) | 0.956 (0.930 - 0.983) |  | 0.726 (0.649 - 0.794) | 0.992 (0.958 - 1.000) |
|  |  | Hispanic/Latino | 827 | 425 | 78 | 6 | 318 | 0.981 (0.960 - 0.993) | 0.845 (0.810 - 0.875) | 0.898 (0.876 - 0.918) | 0.978 (0.967 - 0.990) |  | 0.803 (0.760 - 0.841) | 0.986 (0.970 - 0.995) |
|  |  | White | 1548 | 603 | 198 | 12 | 735 | 0.984 (0.972 - 0.992) | 0.753 (0.721 - 0.782) | 0.864 (0.846 - 0.881) | 0.969 (0.960 - 0.978) |  | 0.788 (0.760 - 0.814) | 0.980 (0.966 - 0.990) |
|  |  | Other Race/Ethnicity | 257 | 134 | 28 | 0 | 95 | 1.000 (0.962 - 1.000) | 0.827 (0.760 - 0.882) | 0.891 (0.846 - 0.926) | 0.983 (0.964 - 1.000) |  | 0.772 (0.688 - 0.843) | 1.000 (0.973 - 1.000) |
|  |  | Asian, Female | 79 | 32 | 5 | 0 | 42 | 1.000 (0.916 - 1.000) | 0.865 (0.712 - 0.955) | 0.937 (0.858 - 0.979) | 0.986 (0.961 - 1.000) |  | 0.894 (0.769 - 0.965) | 1.000 (0.891 - 1.000) |
|  |  | Asian, Male | 58 | 22 | 9 | 1 | 26 | 0.963 (0.810 - 0.999) | 0.710 (0.520 - 0.858) | 0.828 (0.706 - 0.914) | 0.974 (0.930 - 1.000) |  | 0.743 (0.567 - 0.875) | 0.957 (0.781 - 0.999) |
|  |  | Black, Female | 147 | 65 | 18 | 1 | 63 | 0.984 (0.916 - 1.000) | 0.783 (0.679 - 0.866) | 0.871 (0.806 - 0.920) | 0.968 (0.937 - 0.999) |  | 0.778 (0.672 - 0.863) | 0.985 (0.918 - 1.000) |
|  |  | Black, Male | 141 | 65 | 25 | 0 | 51 | 1.000 (0.930 - 1.000) | 0.722 (0.618 - 0.811) | 0.823 (0.749 - 0.882) | 0.942 (0.895 - 0.988) |  | 0.671 (0.554 - 0.775) | 1.000 (0.945 - 1.000) |
|  |  | Hispanic, Female | 421 | 188 | 31 | 3 | 199 | 0.985 (0.957 - 0.997) | 0.858 (0.805 - 0.902) | 0.919 (0.889 - 0.943) | 0.985 (0.973 - 0.997) |  | 0.865 (0.814 - 0.907) | 0.984 (0.955 - 0.997) |
|  |  | Hispanic, Male | 406 | 237 | 47 | 3 | 119 | 0.975 (0.930 - 0.995) | 0.835 (0.786 - 0.876) | 0.877 (0.841 - 0.907) | 0.967 (0.945 - 0.990) |  | 0.717 (0.642 - 0.784) | 0.988 (0.964 - 0.997) |
|  |  | White, Female | 755 | 288 | 71 | 6 | 390 | 0.985 (0.967 - 0.994) | 0.802 (0.757 - 0.842) | 0.898 (0.874 - 0.919) | 0.977 (0.967 - 0.988) |  | 0.846 (0.810 - 0.878) | 0.980 (0.956 - 0.992) |
|  |  | White, Male | 793 | 315 | 127 | 6 | 345 | 0.983 (0.963 - 0.994) | 0.713 (0.668 - 0.754) | 0.832 (0.804 - 0.858) | 0.958 (0.942 - 0.973) |  | 0.731 (0.688 - 0.770) | 0.981 (0.960 - 0.993) |
|  |  | Other Race/Ethnicity, Female | 135 | 62 | 11 | 0 | 62 | 1.000 (0.942 - 1.000) | 0.849 (0.746 - 0.922) | 0.919 (0.859 - 0.959) | 0.986 (0.964 - 1.000) |  | 0.849 (0.746 - 0.922) | 1.000 (0.942 - 1.000) |
|  |  | Other Race/Ethnicity, Male | 122 | 72 | 17 | 0 | 33 | 1.000 (0.894 - 1.000) | 0.809 (0.712 - 0.885) | 0.861 (0.786 - 0.917) | 0.978 (0.943 - 1.000) |  | 0.660 (0.512 - 0.788) | 1.000 (0.950 - 1.000) |
|  | 6 to 12 | Total | 840 | 380 | 112 | 4 | 344 | 0.989 (0.971 - 0.997) | 0.772 (0.733 - 0.809) | 0.862 (0.837 - 0.885) | 0.965 (0.951 - 0.979) |  | 0.754 (0.712 - 0.793) | 0.990 (0.974 - 0.997) |
|  |  | Female | 329 | 144 | 29 | 1 | 155 | 0.994 (0.965 - 1.000) | 0.832 (0.768 - 0.885) | 0.909 (0.872 - 0.938) | 0.982 (0.967 - 0.997) |  | 0.842 (0.782 - 0.892) | 0.993 (0.962 - 1.000) |
|  |  | Male | 511 | 236 | 83 | 3 | 189 | 0.984 (0.955 - 0.997) | 0.740 (0.688 - 0.787) | 0.832 (0.796 - 0.863) | 0.948 (0.925 - 0.970) |  | 0.695 (0.636 - 0.749) | 0.987 (0.964 - 0.997) |
|  |  | Asian | 34 | 14 | 4 | 1 | 15 | 0.938 (0.698 - 0.998) | 0.778 (0.524 - 0.936) | 0.853 (0.689 - 0.950) | 0.917 (0.814 - 1.000) |  | 0.789 (0.544 - 0.939) | 0.933 (0.681 - 0.998) |
|  |  | Black | 95 | 39 | 14 | 0 | 42 | 1.000 (0.916 - 1.000) | 0.736 (0.597 - 0.847) | 0.853 (0.765 - 0.917) | 0.938 (0.884 - 0.991) |  | 0.750 (0.616 - 0.856) | 1.000 (0.910 - 1.000) |
|  |  | Hispanic/Latino | 254 | 146 | 23 | 0 | 85 | 1.000 (0.958 - 1.000) | 0.864 (0.803 - 0.912) | 0.909 (0.867 - 0.942) | 0.991 (0.976 - 1.000) |  | 0.787 (0.698 - 0.860) | 1.000 (0.975 - 1.000) |
|  |  | White | 370 | 134 | 58 | 3 | 175 | 0.983 (0.952 - 0.997) | 0.698 (0.628 - 0.762) | 0.835 (0.793 - 0.871) | 0.946 (0.922 - 0.970) |  | 0.751 (0.690 - 0.805) | 0.978 (0.937 - 0.995) |
|  |  | Other Race/Ethnicity | 78 | 45 | 9 | 0 | 24 | 1.000 (0.858 - 1.000) | 0.833 (0.707 - 0.921) | 0.885 (0.792 - 0.946) | 0.983 (0.946 - 1.000) |  | 0.727 (0.545 - 0.867) | 1.000 (0.921 - 1.000) |
|  |  | Asian, Female | 16 | 5 | 1 | 0 | 10 | 1.000 (0.692 - 1.000) | 0.833 (0.359 - 0.996) | 0.938 (0.698 - 0.998) | 0.917 (0.775 - 1.000) |  | 0.909 (0.587 - 0.998) | 1.000 (0.478 - 1.000) |
|  |  | Asian, Male | 18 | 9 | 3 | 1 | 5 | 0.833 (0.359 - 0.996) | 0.750 (0.428 - 0.945) | 0.778 (0.524 - 0.936) | 0.875 (0.677 - 1.000) |  | 0.625 (0.245 - 0.915) | 0.900 (0.555 - 0.997) |
|  |  | Black, Female | 34 | 17 | 4 | 0 | 13 | 1.000 (0.753 - 1.000) | 0.810 (0.581 - 0.946) | 0.882 (0.725 - 0.967) | 0.974 (0.912 - 1.000) |  | 0.765 (0.501 - 0.932) | 1.000 (0.805 - 1.000) |
|  |  | Black, Male | 61 | 22 | 10 | 0 | 29 | 1.000 (0.881 - 1.000) | 0.688 (0.500 - 0.839) | 0.836 (0.719 - 0.918) | 0.918 (0.843 - 0.993) |  | 0.744 (0.579 - 0.870) | 1.000 (0.846 - 1.000) |
|  |  | Hispanic, Female | 110 | 57 | 5 | 0 | 48 | 1.000 (0.926 - 1.000) | 0.919 (0.822 - 0.973) | 0.955 (0.897 - 0.985) | 0.999 (0.994 - 1.000) |  | 0.906 (0.793 - 0.969) | 1.000 (0.937 - 1.000) |
|  |  | Hispanic, Male | 144 | 89 | 18 | 0 | 37 | 1.000 (0.905 - 1.000) | 0.832 (0.747 - 0.897) | 0.875 (0.810 - 0.924) | 0.975 (0.940 - 1.000) |  | 0.673 (0.533 - 0.793) | 1.000 (0.959 - 1.000) |
|  |  | White, Female | 137 | 50 | 16 | 1 | 70 | 0.986 (0.924 - 1.000) | 0.758 (0.636 - 0.855) | 0.876 (0.809 - 0.926) | 0.972 (0.944 - 1.000) |  | 0.814 (0.716 - 0.890) | 0.980 (0.896 - 1.000) |
|  |  | White, Male | 233 | 84 | 42 | 2 | 105 | 0.981 (0.934 - 0.998) | 0.667 (0.577 - 0.748) | 0.811 (0.755 - 0.859) | 0.926 (0.889 - 0.962) |  | 0.714 (0.634 - 0.786) | 0.977 (0.919 - 0.997) |
|  |  | Other Race/Ethnicity, Female | 30 | 15 | 2 | 0 | 13 | 1.000 (0.753 - 1.000) | 0.882 (0.636 - 0.985) | 0.933 (0.779 - 0.992) | 0.968 (0.898 - 1.000) |  | 0.867 (0.595 - 0.983) | 1.000 (0.782 - 1.000) |
|  |  | Other Race/Ethnicity, Male | 48 | 30 | 7 | 0 | 11 | 1.000 (0.715 - 1.000) | 0.811 (0.648 - 0.920) | 0.854 (0.722 - 0.939) | 0.988 (0.941 - 1.000) |  | 0.611 (0.357 - 0.827) | 1.000 (0.884 - 1.000) |
|  | 13 to 17 | Total | 2262 | 981 | 257 | 16 | 1008 | 0.984 (0.975 - 0.991) | 0.792 (0.769 - 0.815) | 0.879 (0.865 - 0.892) | 0.975 (0.968 - 0.982) |  | 0.797 (0.774 - 0.819) | 0.984 (0.974 - 0.991) |
|  |  | Female | 1235 | 500 | 111 | 9 | 615 | 0.986 (0.973 - 0.993) | 0.818 (0.785 - 0.848) | 0.903 (0.885 - 0.919) | 0.979 (0.971 - 0.987) |  | 0.847 (0.819 - 0.873) | 0.982 (0.967 - 0.992) |
|  |  | Male | 1027 | 481 | 146 | 7 | 393 | 0.983 (0.964 - 0.993) | 0.767 (0.732 - 0.800) | 0.851 (0.828 - 0.872) | 0.970 (0.958 - 0.982) |  | 0.729 (0.689 - 0.766) | 0.986 (0.971 - 0.994) |
|  |  | Asian | 103 | 40 | 10 | 0 | 53 | 1.000 (0.933 - 1.000) | 0.800 (0.663 - 0.900) | 0.903 (0.829 - 0.952) | 0.997 (0.986 - 1.000) |  | 0.841 (0.727 - 0.921) | 1.000 (0.912 - 1.000) |
|  |  | Black | 193 | 91 | 29 | 1 | 72 | 0.986 (0.926 - 1.000) | 0.758 (0.672 - 0.832) | 0.845 (0.786 - 0.893) | 0.964 (0.934 - 0.995) |  | 0.713 (0.614 - 0.799) | 0.989 (0.941 - 1.000) |
|  |  | Hispanic/Latino | 573 | 279 | 55 | 6 | 233 | 0.975 (0.946 - 0.991) | 0.835 (0.791 - 0.873) | 0.894 (0.865 - 0.918) | 0.973 (0.959 - 0.988) |  | 0.809 (0.759 - 0.853) | 0.979 (0.955 - 0.992) |
|  |  | White | 1178 | 469 | 140 | 9 | 560 | 0.984 (0.970 - 0.993) | 0.770 (0.735 - 0.803) | 0.874 (0.853 - 0.892) | 0.975 (0.965 - 0.984) |  | 0.800 (0.768 - 0.829) | 0.981 (0.965 - 0.991) |
|  |  | Other Race/Ethnicity | 179 | 89 | 19 | 0 | 71 | 1.000 (0.949 - 1.000) | 0.824 (0.739 - 0.891) | 0.894 (0.839 - 0.935) | 0.982 (0.960 - 1.000) |  | 0.789 (0.690 - 0.868) | 1.000 (0.959 - 1.000) |
|  |  | Asian, Female | 63 | 27 | 4 | 0 | 32 | 1.000 (0.891 - 1.000) | 0.871 (0.702 - 0.964) | 0.937 (0.845 - 0.982) | 0.997 (0.983 - 1.000) |  | 0.889 (0.739 - 0.969) | 1.000 (0.872 - 1.000) |
|  |  | Asian, Male | 40 | 13 | 6 | 0 | 21 | 1.000 (0.839 - 1.000) | 0.684 (0.434 - 0.874) | 0.850 (0.702 - 0.943) | 1.000 (1.000 - 1.000) |  | 0.778 (0.577 - 0.914) | 1.000 (0.753 - 1.000) |
|  |  | Black, Female | 113 | 48 | 14 | 1 | 50 | 0.980 (0.896 - 1.000) | 0.774 (0.650 - 0.871) | 0.867 (0.791 - 0.924) | 0.964 (0.927 - 1.000) |  | 0.781 (0.660 - 0.875) | 0.980 (0.891 - 0.999) |
|  |  | Black, Male | 80 | 43 | 15 | 0 | 22 | 1.000 (0.846 - 1.000) | 0.741 (0.610 - 0.847) | 0.813 (0.710 - 0.891) | 0.969 (0.917 - 1.000) |  | 0.595 (0.421 - 0.752) | 1.000 (0.918 - 1.000) |
|  |  | Hispanic, Female | 311 | 131 | 26 | 3 | 151 | 0.981 (0.944 - 0.996) | 0.834 (0.767 - 0.889) | 0.907 (0.869 - 0.937) | 0.978 (0.962 - 0.995) |  | 0.853 (0.792 - 0.902) | 0.978 (0.936 - 0.995) |
|  |  | Hispanic, Male | 262 | 148 | 29 | 3 | 82 | 0.965 (0.900 - 0.993) | 0.836 (0.773 - 0.887) | 0.878 (0.832 - 0.915) | 0.963 (0.935 - 0.992) |  | 0.739 (0.647 - 0.818) | 0.980 (0.943 - 0.996) |
|  |  | White, Female | 618 | 238 | 55 | 5 | 320 | 0.985 (0.964 - 0.995) | 0.812 (0.763 - 0.855) | 0.903 (0.877 - 0.925) | 0.978 (0.967 - 0.990) |  | 0.853 (0.813 - 0.888) | 0.979 (0.953 - 0.993) |
|  |  | White, Male | 560 | 231 | 85 | 4 | 240 | 0.984 (0.959 - 0.996) | 0.731 (0.679 - 0.779) | 0.841 (0.808 - 0.870) | 0.969 (0.954 - 0.985) |  | 0.738 (0.687 - 0.785) | 0.983 (0.957 - 0.995) |
|  |  | Other Race/Ethnicity, Female | 105 | 47 | 9 | 0 | 49 | 1.000 (0.927 - 1.000) | 0.839 (0.717 - 0.924) | 0.914 (0.844 - 0.960) | 0.989 (0.968 - 1.000) |  | 0.845 (0.726 - 0.927) | 1.000 (0.925 - 1.000) |
|  |  | Other Race/Ethnicity, Male | 74 | 42 | 10 | 0 | 22 | 1.000 (0.846 - 1.000) | 0.808 (0.675 - 0.904) | 0.865 (0.765 - 0.933) | 0.969 (0.918 - 1.000) |  | 0.688 (0.500 - 0.839) | 1.000 (0.916 - 1.000) |
|  |  |  |  |  |  |  |  |  |  |  |  |  |  |  |
| NLP-med + aCS | 6 to 17 | Total | 3102 | 1392 | 338 | 26 | 1346 | 0.981 (0.972 - 0.988) | 0.805 (0.785 - 0.823) | 0.883 (0.871 - 0.894) | 0.972 (0.965 - 0.978) |  | 0.799 (0.779 - 0.818) | 0.982 (0.973 - 0.988) |
|  |  | Female | 1564 | 657 | 127 | 13 | 767 | 0.983 (0.972 - 0.991) | 0.838 (0.810 - 0.863) | 0.910 (0.895 - 0.924) | 0.979 (0.972 - 0.986) |  | 0.858 (0.833 - 0.880) | 0.981 (0.967 - 0.990) |
|  |  | Male | 1538 | 735 | 211 | 13 | 579 | 0.978 (0.963 - 0.988) | 0.777 (0.749 - 0.803) | 0.854 (0.836 - 0.872) | 0.961 (0.950 - 0.973) |  | 0.733 (0.701 - 0.763) | 0.983 (0.970 - 0.991) |
|  |  | Asian | 137 | 56 | 12 | 2 | 67 | 0.971 (0.899 - 0.996) | 0.824 (0.712 - 0.905) | 0.898 (0.834 - 0.943) | 0.984 (0.962 - 1.000) |  | 0.848 (0.750 - 0.919) | 0.966 (0.881 - 0.996) |
|  |  | Black | 288 | 139 | 34 | 3 | 112 | 0.974 (0.926 - 0.995) | 0.803 (0.736 - 0.860) | 0.872 (0.827 - 0.908) | 0.962 (0.937 - 0.987) |  | 0.767 (0.690 - 0.833) | 0.979 (0.940 - 0.996) |
|  |  | Hispanic/Latino | 827 | 429 | 74 | 9 | 315 | 0.972 (0.948 - 0.987) | 0.853 (0.819 - 0.883) | 0.900 (0.877 - 0.919) | 0.975 (0.963 - 0.987) |  | 0.810 (0.767 - 0.848) | 0.979 (0.961 - 0.991) |
|  |  | White | 1548 | 619 | 182 | 11 | 736 | 0.985 (0.974 - 0.993) | 0.773 (0.742 - 0.801) | 0.875 (0.858 - 0.891) | 0.969 (0.960 - 0.978) |  | 0.802 (0.774 - 0.827) | 0.983 (0.969 - 0.991) |
|  |  | Other Race/Ethnicity | 257 | 133 | 29 | 1 | 94 | 0.989 (0.943 - 1.000) | 0.821 (0.753 - 0.877) | 0.883 (0.838 - 0.920) | 0.978 (0.957 - 0.999) |  | 0.764 (0.679 - 0.836) | 0.993 (0.959 - 1.000) |
|  |  | Asian, Female | 79 | 34 | 3 | 1 | 41 | 0.976 (0.874 - 0.999) | 0.919 (0.781 - 0.983) | 0.949 (0.875 - 0.986) | 0.992 (0.973 - 1.000) |  | 0.932 (0.813 - 0.986) | 0.971 (0.851 - 0.999) |
|  |  | Asian, Male | 58 | 22 | 9 | 1 | 26 | 0.963 (0.810 - 0.999) | 0.710 (0.520 - 0.858) | 0.828 (0.706 - 0.914) | 0.970 (0.923 - 1.000) |  | 0.743 (0.567 - 0.875) | 0.957 (0.781 - 0.999) |
|  |  | Black, Female | 147 | 69 | 14 | 2 | 62 | 0.969 (0.892 - 0.996) | 0.831 (0.733 - 0.905) | 0.891 (0.829 - 0.936) | 0.968 (0.937 - 0.999) |  | 0.816 (0.710 - 0.895) | 0.972 (0.902 - 0.997) |
|  |  | Black, Male | 141 | 70 | 20 | 1 | 50 | 0.980 (0.896 - 1.000) | 0.778 (0.678 - 0.859) | 0.851 (0.781 - 0.905) | 0.953 (0.911 - 0.995) |  | 0.714 (0.594 - 0.816) | 0.986 (0.924 - 1.000) |
|  |  | Hispanic, Female | 421 | 189 | 30 | 5 | 197 | 0.975 (0.943 - 0.992) | 0.863 (0.810 - 0.906) | 0.917 (0.886 - 0.941) | 0.982 (0.969 - 0.995) |  | 0.868 (0.817 - 0.909) | 0.974 (0.941 - 0.992) |
|  |  | Hispanic, Male | 406 | 240 | 44 | 4 | 118 | 0.967 (0.918 - 0.991) | 0.845 (0.798 - 0.885) | 0.882 (0.846 - 0.912) | 0.961 (0.937 - 0.986) |  | 0.728 (0.653 - 0.795) | 0.984 (0.959 - 0.996) |
|  |  | White, Female | 755 | 293 | 66 | 5 | 391 | 0.987 (0.971 - 0.996) | 0.816 (0.772 - 0.855) | 0.906 (0.883 - 0.926) | 0.977 (0.966 - 0.987) |  | 0.856 (0.820 - 0.887) | 0.983 (0.961 - 0.995) |
|  |  | White, Male | 793 | 326 | 116 | 6 | 345 | 0.983 (0.963 - 0.994) | 0.738 (0.694 - 0.778) | 0.846 (0.819 - 0.871) | 0.959 (0.943 - 0.974) |  | 0.748 (0.706 - 0.787) | 0.982 (0.961 - 0.993) |
|  |  | Other Race/Ethnicity, Female | 135 | 63 | 10 | 0 | 62 | 1.000 (0.942 - 1.000) | 0.863 (0.762 - 0.932) | 0.926 (0.868 - 0.964) | 0.985 (0.963 - 1.000) |  | 0.861 (0.759 - 0.931) | 1.000 (0.943 - 1.000) |
|  |  | Other Race/Ethnicity, Male | 122 | 70 | 19 | 1 | 32 | 0.970 (0.842 - 0.999) | 0.787 (0.687 - 0.866) | 0.836 (0.758 - 0.897) | 0.972 (0.931 - 1.000) |  | 0.627 (0.481 - 0.759) | 0.986 (0.924 - 1.000) |
|  | 6 to 12 | Total | 840 | 396 | 96 | 6 | 342 | 0.983 (0.963 - 0.994) | 0.805 (0.767 - 0.839) | 0.879 (0.855 - 0.900) | 0.966 (0.952 - 0.979) |  | 0.781 (0.739 - 0.819) | 0.985 (0.968 - 0.995) |
|  |  | Female | 329 | 148 | 25 | 1 | 155 | 0.994 (0.965 - 1.000) | 0.855 (0.794 - 0.904) | 0.921 (0.886 - 0.948) | 0.982 (0.966 - 0.997) |  | 0.861 (0.802 - 0.908) | 0.993 (0.963 - 1.000) |
|  |  | Male | 511 | 248 | 71 | 5 | 187 | 0.974 (0.940 - 0.991) | 0.777 (0.728 - 0.822) | 0.851 (0.817 - 0.881) | 0.951 (0.929 - 0.973) |  | 0.725 (0.666 - 0.778) | 0.980 (0.954 - 0.994) |
|  |  | Asian | 34 | 14 | 4 | 1 | 15 | 0.938 (0.698 - 0.998) | 0.778 (0.524 - 0.936) | 0.853 (0.689 - 0.950) | 0.934 (0.843 - 1.000) |  | 0.789 (0.544 - 0.939) | 0.933 (0.681 - 0.998) |
|  |  | Black | 95 | 43 | 10 | 1 | 41 | 0.976 (0.874 - 0.999) | 0.811 (0.680 - 0.906) | 0.884 (0.802 - 0.941) | 0.954 (0.907 - 1.000) |  | 0.804 (0.669 - 0.902) | 0.977 (0.880 - 0.999) |
|  |  | Hispanic/Latino | 254 | 148 | 21 | 0 | 85 | 1.000 (0.958 - 1.000) | 0.876 (0.816 - 0.921) | 0.917 (0.876 - 0.948) | 0.990 (0.975 - 1.000) |  | 0.802 (0.713 - 0.873) | 1.000 (0.975 - 1.000) |
|  |  | White | 370 | 143 | 49 | 3 | 175 | 0.983 (0.952 - 0.997) | 0.745 (0.677 - 0.805) | 0.859 (0.820 - 0.893) | 0.951 (0.928 - 0.974) |  | 0.781 (0.721 - 0.834) | 0.979 (0.941 - 0.996) |
|  |  | Other Race/Ethnicity | 78 | 45 | 9 | 1 | 23 | 0.958 (0.789 - 0.999) | 0.833 (0.707 - 0.921) | 0.872 (0.777 - 0.937) | 0.976 (0.933 - 1.000) |  | 0.719 (0.533 - 0.863) | 0.978 (0.885 - 0.999) |
|  |  | Asian, Female | 16 | 5 | 1 | 0 | 10 | 1.000 (0.692 - 1.000) | 0.833 (0.359 - 0.996) | 0.938 (0.698 - 0.998) | 0.950 (0.842 - 1.000) |  | 0.909 (0.587 - 0.998) | 1.000 (0.478 - 1.000) |
|  |  | Asian, Male | 18 | 9 | 3 | 1 | 5 | 0.833 (0.359 - 0.996) | 0.750 (0.428 - 0.945) | 0.778 (0.524 - 0.936) | 0.889 (0.701 - 1.000) |  | 0.625 (0.245 - 0.915) | 0.900 (0.555 - 0.997) |
|  |  | Black, Female | 34 | 19 | 2 | 0 | 13 | 1.000 (0.753 - 1.000) | 0.905 (0.696 - 0.988) | 0.941 (0.803 - 0.993) | 0.989 (0.948 - 1.000) |  | 0.867 (0.595 - 0.983) | 1.000 (0.824 - 1.000) |
|  |  | Black, Male | 61 | 24 | 8 | 1 | 28 | 0.966 (0.822 - 0.999) | 0.750 (0.566 - 0.885) | 0.852 (0.738 - 0.930) | 0.927 (0.856 - 0.998) |  | 0.778 (0.608 - 0.899) | 0.960 (0.796 - 0.999) |
|  |  | Hispanic, Female | 110 | 57 | 5 | 0 | 48 | 1.000 (0.926 - 1.000) | 0.919 (0.822 - 0.973) | 0.955 (0.897 - 0.985) | 0.998 (0.990 - 1.000) |  | 0.906 (0.793 - 0.969) | 1.000 (0.937 - 1.000) |
|  |  | Hispanic, Male | 144 | 91 | 16 | 0 | 37 | 1.000 (0.905 - 1.000) | 0.850 (0.769 - 0.912) | 0.889 (0.826 - 0.935) | 0.976 (0.940 - 1.000) |  | 0.698 (0.557 - 0.817) | 1.000 (0.960 - 1.000) |
|  |  | White, Female | 137 | 52 | 14 | 1 | 70 | 0.986 (0.924 - 1.000) | 0.788 (0.670 - 0.879) | 0.891 (0.826 - 0.937) | 0.961 (0.929 - 0.994) |  | 0.833 (0.736 - 0.906) | 0.981 (0.899 - 1.000) |
|  |  | White, Male | 233 | 91 | 35 | 2 | 105 | 0.981 (0.934 - 0.998) | 0.722 (0.635 - 0.798) | 0.841 (0.788 - 0.886) | 0.941 (0.909 - 0.974) |  | 0.750 (0.670 - 0.819) | 0.978 (0.924 - 0.997) |
|  |  | Other Race/Ethnicity, Female | 30 | 15 | 2 | 0 | 13 | 1.000 (0.753 - 1.000) | 0.882 (0.636 - 0.985) | 0.933 (0.779 - 0.992) | 0.995 (0.969 - 1.000) |  | 0.867 (0.595 - 0.983) | 1.000 (0.782 - 1.000) |
|  |  | Other Race/Ethnicity, Male | 48 | 30 | 7 | 1 | 10 | 0.909 (0.587 - 0.998) | 0.811 (0.648 - 0.920) | 0.833 (0.698 - 0.925) | 0.962 (0.881 - 1.000) |  | 0.588 (0.329 - 0.816) | 0.968 (0.833 - 0.999) |
|  | 13 to 17 | Total | 2262 | 996 | 242 | 20 | 1004 | 0.980 (0.970 - 0.988) | 0.805 (0.781 - 0.826) | 0.884 (0.870 - 0.897) | 0.974 (0.967 - 0.981) |  | 0.806 (0.783 - 0.827) | 0.980 (0.970 - 0.988) |
|  |  | Female | 1235 | 509 | 102 | 12 | 612 | 0.981 (0.967 - 0.990) | 0.833 (0.801 - 0.862) | 0.908 (0.890 - 0.923) | 0.978 (0.970 - 0.987) |  | 0.857 (0.829 - 0.882) | 0.977 (0.960 - 0.988) |
|  |  | Male | 1027 | 487 | 140 | 8 | 392 | 0.980 (0.961 - 0.991) | 0.777 (0.742 - 0.809) | 0.856 (0.833 - 0.877) | 0.966 (0.953 - 0.979) |  | 0.737 (0.697 - 0.774) | 0.984 (0.968 - 0.993) |
|  |  | Asian | 103 | 42 | 8 | 1 | 52 | 0.981 (0.899 - 1.000) | 0.840 (0.709 - 0.928) | 0.913 (0.841 - 0.959) | 0.993 (0.977 - 1.000) |  | 0.867 (0.754 - 0.941) | 0.977 (0.877 - 0.999) |
|  |  | Black | 193 | 96 | 24 | 2 | 71 | 0.973 (0.905 - 0.997) | 0.800 (0.717 - 0.867) | 0.865 (0.809 - 0.910) | 0.966 (0.937 - 0.996) |  | 0.747 (0.648 - 0.831) | 0.980 (0.928 - 0.998) |
|  |  | Hispanic/Latino | 573 | 281 | 53 | 9 | 230 | 0.962 (0.930 - 0.983) | 0.841 (0.798 - 0.879) | 0.892 (0.863 - 0.916) | 0.969 (0.953 - 0.984) |  | 0.813 (0.762 - 0.856) | 0.969 (0.942 - 0.986) |
|  |  | White | 1178 | 476 | 133 | 8 | 561 | 0.986 (0.972 - 0.994) | 0.782 (0.747 - 0.814) | 0.880 (0.860 - 0.898) | 0.973 (0.964 - 0.983) |  | 0.808 (0.777 - 0.837) | 0.983 (0.968 - 0.993) |
|  |  | Other Race/Ethnicity | 179 | 88 | 20 | 0 | 71 | 1.000 (0.949 - 1.000) | 0.815 (0.729 - 0.883) | 0.888 (0.833 - 0.930) | 0.980 (0.956 - 1.000) |  | 0.780 (0.681 - 0.860) | 1.000 (0.959 - 1.000) |
|  |  | Asian, Female | 63 | 29 | 2 | 1 | 31 | 0.969 (0.838 - 0.999) | 0.935 (0.786 - 0.992) | 0.952 (0.867 - 0.990) | 0.996 (0.980 - 1.000) |  | 0.939 (0.798 - 0.993) | 0.967 (0.828 - 0.999) |
|  |  | Asian, Male | 40 | 13 | 6 | 0 | 21 | 1.000 (0.839 - 1.000) | 0.684 (0.434 - 0.874) | 0.850 (0.702 - 0.943) | 0.997 (0.982 - 1.000) |  | 0.778 (0.577 - 0.914) | 1.000 (0.753 - 1.000) |
|  |  | Black, Female | 113 | 50 | 12 | 2 | 49 | 0.961 (0.865 - 0.995) | 0.806 (0.686 - 0.896) | 0.876 (0.801 - 0.931) | 0.962 (0.924 - 1.000) |  | 0.803 (0.682 - 0.894) | 0.962 (0.868 - 0.995) |
|  |  | Black, Male | 80 | 46 | 12 | 0 | 22 | 1.000 (0.846 - 1.000) | 0.793 (0.666 - 0.888) | 0.850 (0.753 - 0.920) | 0.970 (0.919 - 1.000) |  | 0.647 (0.465 - 0.803) | 1.000 (0.923 - 1.000) |
|  |  | Hispanic, Female | 311 | 132 | 25 | 5 | 149 | 0.968 (0.926 - 0.989) | 0.841 (0.774 - 0.894) | 0.904 (0.865 - 0.934) | 0.975 (0.957 - 0.993) |  | 0.856 (0.795 - 0.905) | 0.964 (0.917 - 0.988) |
|  |  | Hispanic, Male | 262 | 149 | 28 | 4 | 81 | 0.953 (0.884 - 0.987) | 0.842 (0.780 - 0.892) | 0.878 (0.832 - 0.915) | 0.956 (0.924 - 0.987) |  | 0.743 (0.651 - 0.822) | 0.974 (0.934 - 0.993) |
|  |  | White, Female | 618 | 241 | 52 | 4 | 321 | 0.988 (0.969 - 0.997) | 0.823 (0.774 - 0.865) | 0.909 (0.884 - 0.931) | 0.980 (0.969 - 0.991) |  | 0.861 (0.821 - 0.894) | 0.984 (0.959 - 0.996) |
|  |  | White, Male | 560 | 235 | 81 | 4 | 240 | 0.984 (0.959 - 0.996) | 0.744 (0.692 - 0.791) | 0.848 (0.816 - 0.877) | 0.964 (0.948 - 0.981) |  | 0.748 (0.696 - 0.794) | 0.983 (0.958 - 0.995) |
|  |  | Other Race/Ethnicity, Female | 105 | 48 | 8 | 0 | 49 | 1.000 (0.927 - 1.000) | 0.857 (0.738 - 0.936) | 0.924 (0.855 - 0.967) | 0.981 (0.954 - 1.000) |  | 0.860 (0.742 - 0.937) | 1.000 (0.926 - 1.000) |
|  |  | Other Race/Ethnicity, Male | 74 | 40 | 12 | 0 | 22 | 1.000 (0.846 - 1.000) | 0.769 (0.632 - 0.875) | 0.838 (0.734 - 0.913) | 0.978 (0.934 - 1.000) |  | 0.647 (0.465 - 0.803) | 1.000 (0.912 - 1.000) |
|  |  |  |  |  |  |  |  |  |  |  |  |  |  |  |
| LLM+aCS | 6 to 17 | Total | 3102 | 1479 | 251 | 33 | 1339 | 0.976 (0.966 - 0.983) | 0.855 (0.837 - 0.871) | 0.908 (0.898 - 0.918) | 0.977 (0.971 - 0.982) |  | 0.842 (0.823 - 0.860) | 0.978 (0.969 - 0.985) |
|  |  | Female | 1564 | 686 | 98 | 13 | 767 | 0.983 (0.972 - 0.991) | 0.875 (0.850 - 0.897) | 0.929 (0.915 - 0.941) | 0.982 (0.976 - 0.989) |  | 0.887 (0.864 - 0.907) | 0.981 (0.968 - 0.990) |
|  |  | Male | 1538 | 793 | 153 | 20 | 572 | 0.966 (0.948 - 0.979) | 0.838 (0.813 - 0.861) | 0.888 (0.871 - 0.903) | 0.969 (0.959 - 0.979) |  | 0.789 (0.757 - 0.818) | 0.975 (0.962 - 0.985) |
|  |  | Asian | 137 | 59 | 9 | 0 | 69 | 1.000 (0.948 - 1.000) | 0.868 (0.764 - 0.938) | 0.934 (0.879 - 0.970) | 0.985 (0.965 - 1.000) |  | 0.885 (0.792 - 0.946) | 1.000 (0.939 - 1.000) |
|  |  | Black | 288 | 145 | 28 | 2 | 113 | 0.983 (0.939 - 0.998) | 0.838 (0.775 - 0.890) | 0.896 (0.855 - 0.929) | 0.970 (0.948 - 0.992) |  | 0.801 (0.726 - 0.864) | 0.986 (0.952 - 0.998) |
|  |  | Hispanic/Latino | 827 | 433 | 70 | 12 | 312 | 0.963 (0.936 - 0.981) | 0.861 (0.827 - 0.890) | 0.901 (0.878 - 0.920) | 0.974 (0.961 - 0.986) |  | 0.817 (0.774 - 0.854) | 0.973 (0.953 - 0.986) |
|  |  | White | 1548 | 679 | 122 | 19 | 728 | 0.975 (0.961 - 0.985) | 0.848 (0.821 - 0.872) | 0.909 (0.893 - 0.923) | 0.976 (0.968 - 0.984) |  | 0.856 (0.831 - 0.879) | 0.973 (0.958 - 0.984) |
|  |  | Other Race/Ethnicity | 257 | 143 | 19 | 0 | 95 | 1.000 (0.962 - 1.000) | 0.883 (0.823 - 0.928) | 0.926 (0.887 - 0.955) | 0.988 (0.972 - 1.000) |  | 0.833 (0.752 - 0.897) | 1.000 (0.975 - 1.000) |
|  |  | Asian, Female | 79 | 33 | 4 | 0 | 42 | 1.000 (0.916 - 1.000) | 0.892 (0.746 - 0.970) | 0.949 (0.875 - 0.986) | 0.977 (0.943 - 1.000) |  | 0.913 (0.792 - 0.976) | 1.000 (0.894 - 1.000) |
|  |  | Asian, Male | 58 | 26 | 5 | 0 | 27 | 1.000 (0.872 - 1.000) | 0.839 (0.663 - 0.945) | 0.914 (0.810 - 0.971) | 0.995 (0.976 - 1.000) |  | 0.844 (0.672 - 0.947) | 1.000 (0.868 - 1.000) |
|  |  | Black, Female | 147 | 70 | 13 | 2 | 62 | 0.969 (0.892 - 0.996) | 0.843 (0.747 - 0.914) | 0.898 (0.837 - 0.942) | 0.970 (0.939 - 1.000) |  | 0.827 (0.722 - 0.904) | 0.972 (0.903 - 0.997) |
|  |  | Black, Male | 141 | 75 | 15 | 0 | 51 | 1.000 (0.930 - 1.000) | 0.833 (0.740 - 0.904) | 0.894 (0.831 - 0.939) | 0.972 (0.939 - 1.000) |  | 0.773 (0.653 - 0.867) | 1.000 (0.952 - 1.000) |
|  |  | Hispanic, Female | 421 | 192 | 27 | 4 | 198 | 0.980 (0.950 - 0.995) | 0.877 (0.826 - 0.917) | 0.926 (0.897 - 0.949) | 0.984 (0.972 - 0.997) |  | 0.880 (0.830 - 0.919) | 0.980 (0.949 - 0.994) |
|  |  | Hispanic, Male | 406 | 241 | 43 | 8 | 114 | 0.934 (0.875 - 0.971) | 0.849 (0.802 - 0.888) | 0.874 (0.838 - 0.905) | 0.958 (0.932 - 0.983) |  | 0.726 (0.649 - 0.794) | 0.968 (0.938 - 0.986) |
|  |  | White, Female | 755 | 315 | 44 | 7 | 389 | 0.982 (0.964 - 0.993) | 0.877 (0.839 - 0.910) | 0.932 (0.912 - 0.949) | 0.981 (0.971 - 0.991) |  | 0.898 (0.866 - 0.925) | 0.978 (0.956 - 0.991) |
|  |  | White, Male | 793 | 364 | 78 | 12 | 339 | 0.966 (0.941 - 0.982) | 0.824 (0.785 - 0.858) | 0.887 (0.862 - 0.908) | 0.969 (0.956 - 0.982) |  | 0.813 (0.772 - 0.849) | 0.968 (0.945 - 0.983) |
|  |  | Other Race/Ethnicity, Female | 135 | 65 | 8 | 0 | 62 | 1.000 (0.942 - 1.000) | 0.890 (0.795 - 0.951) | 0.941 (0.887 - 0.974) | 0.993 (0.978 - 1.000) |  | 0.886 (0.787 - 0.949) | 1.000 (0.945 - 1.000) |
|  |  | Other Race/Ethnicity, Male | 122 | 78 | 11 | 0 | 33 | 1.000 (0.894 - 1.000) | 0.876 (0.790 - 0.937) | 0.910 (0.844 - 0.954) | 0.981 (0.948 - 1.000) |  | 0.750 (0.597 - 0.868) | 1.000 (0.954 - 1.000) |
|  | 6 to 12 | Total | 840 | 432 | 60 | 9 | 339 | 0.974 (0.951 - 0.988) | 0.878 (0.846 - 0.906) | 0.918 (0.897 - 0.936) | 0.975 (0.964 - 0.987) |  | 0.850 (0.811 - 0.883) | 0.980 (0.962 - 0.991) |
|  |  | Female | 329 | 161 | 12 | 2 | 154 | 0.987 (0.954 - 0.998) | 0.931 (0.882 - 0.964) | 0.957 (0.930 - 0.977) | 0.983 (0.969 - 0.998) |  | 0.928 (0.877 - 0.962) | 0.988 (0.956 - 0.999) |
|  |  | Male | 511 | 271 | 48 | 7 | 185 | 0.964 (0.926 - 0.985) | 0.850 (0.805 - 0.887) | 0.892 (0.862 - 0.918) | 0.966 (0.948 - 0.985) |  | 0.794 (0.736 - 0.844) | 0.975 (0.949 - 0.990) |
|  |  | Asian | 34 | 15 | 3 | 0 | 16 | 1.000 (0.794 - 1.000) | 0.833 (0.586 - 0.964) | 0.912 (0.763 - 0.981) | 0.944 (0.861 - 1.000) |  | 0.842 (0.604 - 0.966) | 1.000 (0.782 - 1.000) |
|  |  | Black | 95 | 44 | 9 | 0 | 42 | 1.000 (0.916 - 1.000) | 0.830 (0.702 - 0.919) | 0.905 (0.828 - 0.956) | 0.959 (0.915 - 1.000) |  | 0.824 (0.691 - 0.916) | 1.000 (0.920 - 1.000) |
|  |  | Hispanic/Latino | 254 | 151 | 18 | 2 | 83 | 0.976 (0.918 - 0.997) | 0.893 (0.837 - 0.936) | 0.921 (0.881 - 0.951) | 0.985 (0.967 - 1.000) |  | 0.822 (0.733 - 0.891) | 0.987 (0.954 - 0.998) |
|  |  | White | 370 | 167 | 25 | 7 | 171 | 0.961 (0.921 - 0.984) | 0.870 (0.814 - 0.914) | 0.914 (0.880 - 0.940) | 0.971 (0.953 - 0.989) |  | 0.872 (0.817 - 0.916) | 0.960 (0.919 - 0.984) |
|  |  | Other Race/Ethnicity | 78 | 50 | 4 | 0 | 24 | 1.000 (0.858 - 1.000) | 0.926 (0.821 - 0.979) | 0.949 (0.874 - 0.986) | 0.991 (0.963 - 1.000) |  | 0.857 (0.673 - 0.960) | 1.000 (0.929 - 1.000) |
|  |  | Asian, Female | 16 | 5 | 1 | 0 | 10 | 1.000 (0.692 - 1.000) | 0.833 (0.359 - 0.996) | 0.938 (0.698 - 0.998) | 0.900 (0.745 - 1.000) |  | 0.909 (0.587 - 0.998) | 1.000 (0.478 - 1.000) |
|  |  | Asian, Male | 18 | 10 | 2 | 0 | 6 | 1.000 (0.541 - 1.000) | 0.833 (0.516 - 0.979) | 0.889 (0.653 - 0.986) | 0.972 (0.875 - 1.000) |  | 0.750 (0.349 - 0.968) | 1.000 (0.692 - 1.000) |
|  |  | Black, Female | 34 | 19 | 2 | 0 | 13 | 1.000 (0.753 - 1.000) | 0.905 (0.696 - 0.988) | 0.941 (0.803 - 0.993) | 0.963 (0.889 - 1.000) |  | 0.867 (0.595 - 0.983) | 1.000 (0.824 - 1.000) |
|  |  | Black, Male | 61 | 25 | 7 | 0 | 29 | 1.000 (0.881 - 1.000) | 0.781 (0.600 - 0.907) | 0.885 (0.778 - 0.953) | 0.958 (0.904 - 1.000) |  | 0.806 (0.640 - 0.918) | 1.000 (0.863 - 1.000) |
|  |  | Hispanic, Female | 110 | 58 | 4 | 0 | 48 | 1.000 (0.926 - 1.000) | 0.935 (0.843 - 0.982) | 0.964 (0.910 - 0.990) | 0.997 (0.985 - 1.000) |  | 0.923 (0.815 - 0.979) | 1.000 (0.938 - 1.000) |
|  |  | Hispanic, Male | 144 | 93 | 14 | 2 | 35 | 0.946 (0.818 - 0.993) | 0.869 (0.790 - 0.927) | 0.889 (0.826 - 0.935) | 0.965 (0.923 - 1.000) |  | 0.714 (0.567 - 0.834) | 0.979 (0.926 - 0.997) |
|  |  | White, Female | 137 | 61 | 5 | 2 | 69 | 0.972 (0.902 - 0.997) | 0.924 (0.832 - 0.975) | 0.949 (0.898 - 0.979) | 0.980 (0.956 - 1.000) |  | 0.932 (0.849 - 0.978) | 0.968 (0.890 - 0.996) |
|  |  | White, Male | 233 | 106 | 20 | 5 | 102 | 0.953 (0.894 - 0.985) | 0.841 (0.766 - 0.900) | 0.893 (0.846 - 0.929) | 0.963 (0.937 - 0.989) |  | 0.836 (0.758 - 0.897) | 0.955 (0.898 - 0.985) |
|  |  | Other Race/Ethnicity, Female | 30 | 17 | 0 | 0 | 13 | 1.000 (0.753 - 1.000) | 1.000 (0.805 - 1.000) | 1.000 (0.884 - 1.000) | 1.000 (1.000 - 1.000) |  | 1.000 (0.753 - 1.000) | 1.000 (0.805 - 1.000) |
|  |  | Other Race/Ethnicity, Male | 48 | 33 | 4 | 0 | 11 | 1.000 (0.715 - 1.000) | 0.892 (0.746 - 0.970) | 0.917 (0.800 - 0.977) | 0.988 (0.941 - 1.000) |  | 0.733 (0.449 - 0.922) | 1.000 (0.894 - 1.000) |
|  | 13 to 17 | Total | 2262 | 1047 | 191 | 24 | 1000 | 0.977 (0.965 - 0.985) | 0.846 (0.824 - 0.865) | 0.905 (0.892 - 0.917) | 0.978 (0.971 - 0.984) |  | 0.840 (0.818 - 0.860) | 0.978 (0.967 - 0.986) |
|  |  | Female | 1235 | 525 | 86 | 11 | 613 | 0.982 (0.969 - 0.991) | 0.859 (0.829 - 0.886) | 0.921 (0.905 - 0.936) | 0.982 (0.975 - 0.990) |  | 0.877 (0.850 - 0.900) | 0.979 (0.964 - 0.990) |
|  |  | Male | 1027 | 522 | 105 | 13 | 387 | 0.968 (0.945 - 0.983) | 0.833 (0.801 - 0.861) | 0.885 (0.864 - 0.904) | 0.971 (0.959 - 0.983) |  | 0.787 (0.748 - 0.822) | 0.976 (0.959 - 0.987) |
|  |  | Asian | 103 | 44 | 6 | 0 | 53 | 1.000 (0.933 - 1.000) | 0.880 (0.757 - 0.955) | 0.942 (0.878 - 0.978) | 0.997 (0.987 - 1.000) |  | 0.898 (0.792 - 0.962) | 1.000 (0.920 - 1.000) |
|  |  | Black | 193 | 101 | 19 | 2 | 71 | 0.973 (0.905 - 0.997) | 0.842 (0.764 - 0.902) | 0.891 (0.838 - 0.931) | 0.976 (0.950 - 1.000) |  | 0.789 (0.690 - 0.868) | 0.981 (0.932 - 0.998) |
|  |  | Hispanic/Latino | 573 | 282 | 52 | 10 | 229 | 0.958 (0.924 - 0.980) | 0.844 (0.801 - 0.881) | 0.892 (0.863 - 0.916) | 0.969 (0.953 - 0.985) |  | 0.815 (0.765 - 0.859) | 0.966 (0.938 - 0.983) |
|  |  | White | 1178 | 512 | 97 | 12 | 557 | 0.979 (0.963 - 0.989) | 0.841 (0.809 - 0.869) | 0.907 (0.889 - 0.923) | 0.977 (0.968 - 0.986) |  | 0.852 (0.822 - 0.878) | 0.977 (0.960 - 0.988) |
|  |  | Other Race/Ethnicity | 179 | 93 | 15 | 0 | 71 | 1.000 (0.949 - 1.000) | 0.861 (0.781 - 0.920) | 0.916 (0.866 - 0.952) | 0.987 (0.968 - 1.000) |  | 0.826 (0.729 - 0.899) | 1.000 (0.961 - 1.000) |
|  |  | Asian, Female | 63 | 28 | 3 | 0 | 32 | 1.000 (0.891 - 1.000) | 0.903 (0.742 - 0.980) | 0.952 (0.867 - 0.990) | 0.996 (0.980 - 1.000) |  | 0.914 (0.769 - 0.982) | 1.000 (0.877 - 1.000) |
|  |  | Asian, Male | 40 | 16 | 3 | 0 | 21 | 1.000 (0.839 - 1.000) | 0.842 (0.604 - 0.966) | 0.925 (0.796 - 0.984) | 1.000 (1.000 - 1.000) |  | 0.875 (0.676 - 0.973) | 1.000 (0.794 - 1.000) |
|  |  | Black, Female | 113 | 51 | 11 | 2 | 49 | 0.961 (0.865 - 0.995) | 0.823 (0.705 - 0.908) | 0.885 (0.811 - 0.937) | 0.973 (0.941 - 1.000) |  | 0.817 (0.696 - 0.905) | 0.962 (0.870 - 0.995) |
|  |  | Black, Male | 80 | 50 | 8 | 0 | 22 | 1.000 (0.846 - 1.000) | 0.862 (0.746 - 0.939) | 0.900 (0.812 - 0.956) | 0.985 (0.949 - 1.000) |  | 0.733 (0.541 - 0.877) | 1.000 (0.929 - 1.000) |
|  |  | Hispanic, Female | 311 | 134 | 23 | 4 | 150 | 0.974 (0.935 - 0.993) | 0.854 (0.788 - 0.905) | 0.913 (0.876 - 0.942) | 0.978 (0.962 - 0.995) |  | 0.867 (0.807 - 0.914) | 0.971 (0.927 - 0.992) |
|  |  | Hispanic, Male | 262 | 148 | 29 | 6 | 79 | 0.929 (0.853 - 0.974) | 0.836 (0.773 - 0.887) | 0.866 (0.819 - 0.905) | 0.956 (0.925 - 0.987) |  | 0.731 (0.638 - 0.812) | 0.961 (0.917 - 0.986) |
|  |  | White, Female | 618 | 254 | 39 | 5 | 320 | 0.985 (0.964 - 0.995) | 0.867 (0.823 - 0.904) | 0.929 (0.906 - 0.948) | 0.981 (0.971 - 0.992) |  | 0.891 (0.854 - 0.922) | 0.981 (0.956 - 0.994) |
|  |  | White, Male | 560 | 258 | 58 | 7 | 237 | 0.971 (0.942 - 0.988) | 0.816 (0.769 - 0.858) | 0.884 (0.854 - 0.909) | 0.971 (0.956 - 0.986) |  | 0.803 (0.753 - 0.847) | 0.974 (0.946 - 0.989) |
|  |  | Other Race/Ethnicity, Female | 105 | 48 | 8 | 0 | 49 | 1.000 (0.927 - 1.000) | 0.857 (0.738 - 0.936) | 0.924 (0.855 - 0.967) | 0.992 (0.973 - 1.000) |  | 0.860 (0.742 - 0.937) | 1.000 (0.926 - 1.000) |
|  |  | Other Race/Ethnicity, Male | 74 | 45 | 7 | 0 | 22 | 1.000 (0.846 - 1.000) | 0.865 (0.742 - 0.944) | 0.905 (0.815 - 0.961) | 0.976 (0.929 - 1.000) |  | 0.759 (0.565 - 0.897) | 1.000 (0.921 - 1.000) |

*Other Race and Ethnicity includes American Indian or Alaska Native, Native Hawaiian or Other Pacific Islander, and multiple races and ethnicities; excludes unknown.*

*The feature set notation is as follows: ICD/CC refers to a feature set based on International Classification of Diseases, Clinical Modification, Version 10, codes for non-fatal suicide attempt and intentional self-harm, as defined by the Centers for Disease Control and Prevention Case Surveillance definition list, plus suicide-related chief concern; c-SSRS+ICD/CC combines ICD/CC with c-SSRS item scores; MH dx+ICD/CC combines ICD/CC with Child and Adolescent Mental Health Disorders Classification System ICD-10-CM code categories; aCS represents all available structured data; NLP-gen and NLP-med are feature sets based on vectorized text features with embeddings derived from the Universal Sentence Encoder and MedEmbed, respectively; LLM refers to Likert-type scores generated by the open-source language model llama-3.3-70B. Feature sets denoted by (aCS+) indicate combinations of aCS with the corresponding text-based feature set (NLP-gen, NLP-med, or LLM).*
