# Supplementary material for: Detecting Pediatric Emergency Service Use for Suicide and Self-Harm: Multimodal Analysis of 3828 Encounters
Source: JMIR Ment Health. 2026 Feb 4;13:e82371. doi: 10.2196/82371 (PMC12871580; doi:10.2196/82371)
Supplement: Multimedia Appendix 16 [file mental-v13-e82371-s016.docx]

**
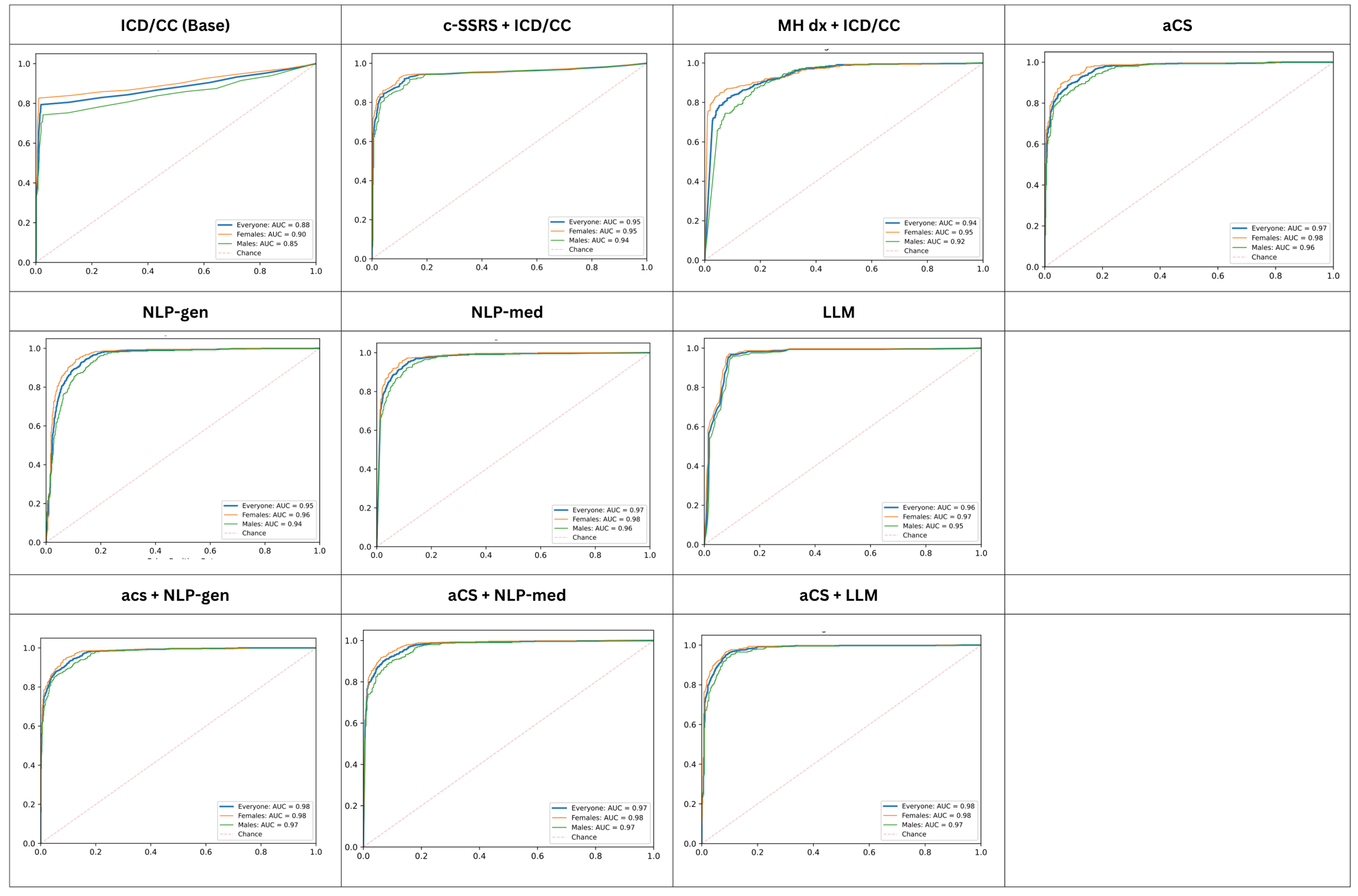
**

**Multimedia Appendix 16 (Adolescents). Area Under the Receiver Operating Characteristic Curves by Feature Set by Sex and Age Group.** The diagram is a composite of receiver operating characteristic (ROC) plots across 10 model types: 4 structured data models (ICD/CC Base, c-SSRS + ICD/CC, MH dx + ICD/CC, aCS), 3 text models (NLP-gen, NLP-med, LLM), and 3 combined models (aCS + NLP-gen, aCS + NLP-med, aCS + LLM). Each panel displays the ROC curve for a feature type, for adolescents overall (solid blue), male adolescents (green) and female adolescents (orange). *The feature set notation is as follows: ICD/CC refers to a feature set based on International Classification of Diseases, Clinical Modification, Version 10, codes for non-fatal suicide attempt and intentional self-harm, as defined by the Centers for Disease Control and Prevention Case Surveillance definition list, plus suicide-related chief concern; c-SSRS+ICD/CC combines ICD/CC with c-SSRS item scores; MH dx+ICD/CC combines ICD/CC with Child and Adolescent Mental Health Disorders Classification System ICD-10-CM code categories; aCS represents all available structured data; NLP-gen and NLP-med are feature sets based on vectorized text features with embeddings derived from the Universal Sentence Encoder and MedEmbed, respectively; LLM refers to Likert-type scores generated by the open-source language model llama-3.3-70B. Feature sets denoted by (aCS+) indicate combinations of aCS with the corresponding text-based feature set (NLP-gen, NLP-med, or LLM).*
