# Supplementary material for: Detecting Pediatric Emergency Service Use for Suicide and Self-Harm: Multimodal Analysis of 3828 Encounters
Source: JMIR Ment Health. 2026 Feb 4;13:e82371. doi: 10.2196/82371 (PMC12871580; doi:10.2196/82371)
Supplement: Multimedia Appendix 19 [file mental-v13-e82371-s019.docx]

**Multimedia Appendix 19. Detection Performance by Classifier and ED Mental Health Diagnosis**

| **Classifier** | Category | **N** | **TN** | **FP** | **FN** | **TP** |  |  |  | **ROC-AUC** |  | **PPV** | **NPV** |
| --- | --- | --- | --- | --- | --- | --- | --- | --- | --- | --- | --- | --- | --- |
| **ICD/CC** | ADHD | 700 | 101 | 242 | 38 | 319 | 0.894 (0.857 - 0.924) | 0.294 (0.247 - 0.346) | 0.600 (0.563 - 0.637) | 0.809 (0.777 - 0.841) |  | 0.569 (0.526 - 0.610) | 0.727 (0.645 - 0.799) |
|  | Anxiety disorders | 933 | 105 | 254 | 38 | 536 | 0.934 (0.910 - 0.953) | 0.292 (0.246 - 0.343) | 0.687 (0.656 - 0.717) | 0.878 (0.856 - 0.899) |  | 0.678 (0.645 - 0.711) | 0.734 (0.654 - 0.805) |
|  | Autism spectrum disorder | 395 | 61 | 164 | 26 | 144 | 0.847 (0.784 - 0.898) | 0.271 (0.214 - 0.334) | 0.519 (0.468 - 0.569) | 0.736 (0.686 - 0.787) |  | 0.468 (0.411 - 0.525) | 0.701 (0.594 - 0.795) |
|  | Bipolar and related disorders | 144 | 11 | 37 | 10 | 86 | 0.896 (0.817 - 0.949) | 0.229 (0.120 - 0.373) | 0.674 (0.591 - 0.749) | 0.804 (0.734 - 0.874) |  | 0.699 (0.610 - 0.779) | 0.524 (0.298 - 0.743) |
|  | Communication disorders | 23 | 5 | 12 | 0 | 6 | 1.000 (0.541 - 1.000) | 0.294 (0.103 - 0.560) | 0.478 (0.268 - 0.694) | 0.863 (0.662 - 1.063) |  | 0.333 (0.133 - 0.590) | 1.000 (0.478 - 1.000) |
|  | Depressive disorders | 1099 | 76 | 163 | 42 | 818 | 0.951 (0.935 - 0.965) | 0.318 (0.259 - 0.381) | 0.813 (0.789 - 0.836) | 0.896 (0.878 - 0.915) |  | 0.834 (0.809 - 0.857) | 0.644 (0.551 - 0.730) |
|  | Developmental delay or unspecified neurodevelopmental disorder | 56 | 13 | 30 | 2 | 11 | 0.846 (0.546 - 0.981) | 0.302 (0.172 - 0.461) | 0.429 (0.297 - 0.568) | 0.735 (0.566 - 0.904) |  | 0.268 (0.142 - 0.429) | 0.867 (0.595 - 0.983) |
|  | Disruptive, impulse control and conduct disorders | 215 | 27 | 69 | 20 | 99 | 0.832 (0.752 - 0.894) | 0.281 (0.194 - 0.382) | 0.586 (0.517 - 0.653) | 0.703 (0.635 - 0.772) |  | 0.589 (0.511 - 0.664) | 0.574 (0.422 - 0.717) |
|  | Feeding and eating disorders | 86 | 14 | 32 | 7 | 33 | 0.825 (0.672 - 0.927) | 0.304 (0.177 - 0.458) | 0.547 (0.435 - 0.654) | 0.774 (0.673 - 0.875) |  | 0.508 (0.381 - 0.634) | 0.667 (0.430 - 0.854) |
|  | Intellectual disability | 55 | 14 | 20 | 8 | 13 | 0.619 (0.384 - 0.819) | 0.412 (0.246 - 0.593) | 0.491 (0.354 - 0.629) | 0.568 (0.410 - 0.726) |  | 0.394 (0.229 - 0.579) | 0.636 (0.407 - 0.828) |
|  | Mental health symptom | 436 | 39 | 102 | 42 | 253 | 0.858 (0.812 - 0.895) | 0.277 (0.205 - 0.358) | 0.670 (0.623 - 0.714) | 0.745 (0.699 - 0.791) |  | 0.713 (0.663 - 0.759) | 0.481 (0.369 - 0.595) |
|  | Miscellaneous | 158 | 25 | 46 | 13 | 74 | 0.851 (0.758 - 0.918) | 0.352 (0.242 - 0.475) | 0.627 (0.546 - 0.702) | 0.763 (0.689 - 0.836) |  | 0.617 (0.524 - 0.704) | 0.658 (0.486 - 0.804) |
|  | Motor disorders | 36 | 7 | 12 | 0 | 17 | 1.000 (0.805 - 1.000) | 0.368 (0.163 - 0.616) | 0.667 (0.490 - 0.814) | 0.933 (0.845 - 1.022) |  | 0.586 (0.389 - 0.765) | 1.000 (0.590 - 1.000) |
|  | Neurocognitive disorders | 54 | 14 | 30 | 1 | 9 | 0.900 (0.555 - 0.997) | 0.318 (0.186 - 0.476) | 0.426 (0.292 - 0.568) | 0.925 (0.809 - 1.041) |  | 0.231 (0.111 - 0.393) | 0.933 (0.681 - 0.998) |
|  | Obsessive-compulsive and related disorders | 129 | 14 | 26 | 9 | 80 | 0.899 (0.817 - 0.953) | 0.350 (0.206 - 0.517) | 0.729 (0.643 - 0.803) | 0.864 (0.802 - 0.925) |  | 0.755 (0.662 - 0.833) | 0.609 (0.385 - 0.803) |
|  | Personality disorders | 23 | 0 | 5 | 3 | 15 | 0.833 (0.586 - 0.964) | 0.000 (0.000 - 0.522) | 0.652 (0.427 - 0.836) | 0.711 (0.473 - 0.949) |  | 0.750 (0.509 - 0.913) | 0.000 (0.000 - 0.708) |
|  | Schizophrenia spectrum and other psychotic disorders | 116 | 13 | 29 | 16 | 58 | 0.784 (0.673 - 0.871) | 0.310 (0.176 - 0.471) | 0.612 (0.517 - 0.701) | 0.718 (0.626 - 0.811) |  | 0.667 (0.557 - 0.764) | 0.448 (0.264 - 0.643) |
|  | Sexuality and gender identity disorders | 29 | 2 | 2 | 0 | 25 | 1.000 (0.863 - 1.000) | 0.500 (0.068 - 0.932) | 0.931 (0.772 - 0.992) | 0.940 (0.849 - 1.031) |  | 0.926 (0.757 - 0.991) | 1.000 (0.158 - 1.000) |
|  | Somatic symptom and related disorders | 23 | 7 | 11 | 0 | 5 | 1.000 (0.478 - 1.000) | 0.389 (0.173 - 0.643) | 0.522 (0.306 - 0.732) | 0.683 (0.397 - 0.969) |  | 0.313 (0.110 - 0.587) | 1.000 (0.590 - 1.000) |
|  | Specific learning disorders | 11 | 1 | 5 | 0 | 5 | 1.000 (0.478 - 1.000) | 0.167 (0.004 - 0.641) | 0.545 (0.234 - 0.833) | 0.967 (0.845 - 1.088) |  | 0.500 (0.187 - 0.813) | 1.000 (0.025 - 1.000) |
|  | Substance related and addictive disorders | 389 | 67 | 170 | 10 | 142 | 0.934 (0.882 - 0.968) | 0.283 (0.226 - 0.345) | 0.537 (0.486 - 0.588) | 0.878 (0.840 - 0.917) |  | 0.455 (0.399 - 0.512) | 0.870 (0.774 - 0.936) |
|  | Trauma and stressor-related disorders | 202 | 18 | 55 | 11 | 118 | 0.915 (0.853 - 0.957) | 0.247 (0.153 - 0.361) | 0.673 (0.604 - 0.737) | 0.842 (0.789 - 0.895) |  | 0.682 (0.607 - 0.751) | 0.621 (0.423 - 0.793) |
|  | Psychiatric comorbidity (≥ 2 CAMHD-CS diagnostic groups) | 1866 | 168 | 412 | 84 | 1202 | 0.935 (0.920 - 0.948) | 0.290 (0.253 - 0.328) | 0.734 (0.714 - 0.754) | 0.877 (0.861 - 0.892) |  | 0.745 (0.723 - 0.766) | 0.667 (0.605 - 0.725) |
|  |  |  |  |  |  |  |  |  |  |  |  |  |  |
| **c-SSRS+**  **ICD/CC** | ADHD | 700 | 274 | 69 | 47 | 310 | 0.868 (0.829 - 0.902) | 0.799 (0.752 - 0.840) | 0.834 (0.805 - 0.861) | 0.895 (0.871 - 0.919) |  | 0.818 (0.775 - 0.855) | 0.854 (0.810 - 0.890) |
|  | Anxiety disorders | 933 | 284 | 75 | 37 | 537 | 0.936 (0.912 - 0.954) | 0.791 (0.745 - 0.832) | 0.880 (0.857 - 0.900) | 0.929 (0.913 - 0.945) |  | 0.877 (0.849 - 0.902) | 0.885 (0.845 - 0.918) |
|  | Autism spectrum disorder | 395 | 177 | 48 | 38 | 132 | 0.776 (0.706 - 0.837) | 0.787 (0.727 - 0.838) | 0.782 (0.738 - 0.822) | 0.851 (0.811 - 0.891) |  | 0.733 (0.662 - 0.796) | 0.823 (0.766 - 0.872) |
|  | Bipolar and related disorders | 144 | 24 | 24 | 15 | 81 | 0.844 (0.755 - 0.910) | 0.500 (0.352 - 0.648) | 0.729 (0.649 - 0.800) | 0.819 (0.751 - 0.886) |  | 0.771 (0.679 - 0.848) | 0.615 (0.446 - 0.766) |
|  | Communication disorders | 23 | 14 | 3 | 1 | 5 | 0.833 (0.359 - 0.996) | 0.824 (0.566 - 0.962) | 0.826 (0.612 - 0.950) | 0.941 (0.804 - 1.078) |  | 0.625 (0.245 - 0.915) | 0.933 (0.681 - 0.998) |
|  | Depressive disorders | 1099 | 168 | 71 | 49 | 811 | 0.943 (0.925 - 0.958) | 0.703 (0.641 - 0.760) | 0.891 (0.871 - 0.909) | 0.931 (0.916 - 0.945) |  | 0.920 (0.900 - 0.937) | 0.774 (0.713 - 0.828) |
|  | Developmental delay or unspecified neurodevelopmental disorder | 56 | 39 | 4 | 1 | 12 | 0.923 (0.640 - 0.998) | 0.907 (0.779 - 0.974) | 0.911 (0.804 - 0.970) | 0.912 (0.802 - 1.022) |  | 0.750 (0.476 - 0.927) | 0.975 (0.868 - 0.999) |
|  | Disruptive, impulse control and conduct disorders | 215 | 51 | 45 | 32 | 87 | 0.731 (0.642 - 0.808) | 0.531 (0.427 - 0.634) | 0.642 (0.574 - 0.706) | 0.728 (0.661 - 0.794) |  | 0.659 (0.572 - 0.739) | 0.614 (0.501 - 0.719) |
|  | Feeding and eating disorders | 86 | 36 | 10 | 7 | 33 | 0.825 (0.672 - 0.927) | 0.783 (0.636 - 0.891) | 0.802 (0.702 - 0.880) | 0.849 (0.765 - 0.934) |  | 0.767 (0.614 - 0.882) | 0.837 (0.693 - 0.932) |
|  | Intellectual disability | 55 | 28 | 6 | 6 | 15 | 0.714 (0.478 - 0.887) | 0.824 (0.655 - 0.932) | 0.782 (0.650 - 0.882) | 0.756 (0.618 - 0.893) |  | 0.714 (0.478 - 0.887) | 0.824 (0.655 - 0.932) |
|  | Mental health symptom | 436 | 72 | 69 | 47 | 248 | 0.841 (0.794 - 0.881) | 0.511 (0.425 - 0.596) | 0.734 (0.690 - 0.775) | 0.822 (0.784 - 0.860) |  | 0.782 (0.733 - 0.827) | 0.605 (0.511 - 0.693) |
|  | Miscellaneous | 158 | 58 | 13 | 16 | 71 | 0.816 (0.719 - 0.891) | 0.817 (0.707 - 0.899) | 0.816 (0.747 - 0.873) | 0.864 (0.807 - 0.920) |  | 0.845 (0.750 - 0.915) | 0.784 (0.673 - 0.871) |
|  | Motor disorders | 36 | 17 | 2 | 3 | 14 | 0.824 (0.566 - 0.962) | 0.895 (0.669 - 0.987) | 0.861 (0.705 - 0.953) | 0.924 (0.829 - 1.019) |  | 0.875 (0.617 - 0.984) | 0.850 (0.621 - 0.968) |
|  | Neurocognitive disorders | 54 | 41 | 3 | 0 | 10 | 1.000 (0.692 - 1.000) | 0.932 (0.813 - 0.986) | 0.944 (0.846 - 0.988) | 1.000 (1.000 - 1.000) |  | 0.769 (0.462 - 0.950) | 1.000 (0.914 - 1.000) |
|  | Obsessive-compulsive and related disorders | 129 | 30 | 10 | 10 | 79 | 0.888 (0.803 - 0.945) | 0.750 (0.588 - 0.873) | 0.845 (0.771 - 0.903) | 0.919 (0.873 - 0.965) |  | 0.888 (0.803 - 0.945) | 0.750 (0.588 - 0.873) |
|  | Personality disorders | 23 | 4 | 1 | 2 | 16 | 0.889 (0.653 - 0.986) | 0.800 (0.284 - 0.995) | 0.870 (0.664 - 0.972) | 0.933 (0.830 - 1.036) |  | 0.941 (0.713 - 0.999) | 0.667 (0.223 - 0.957) |
|  | Schizophrenia spectrum and other psychotic disorders | 116 | 18 | 24 | 14 | 60 | 0.811 (0.703 - 0.893) | 0.429 (0.277 - 0.590) | 0.672 (0.579 - 0.757) | 0.777 (0.694 - 0.860) |  | 0.714 (0.605 - 0.808) | 0.563 (0.377 - 0.736) |
|  | Sexuality and gender identity disorders | 29 | 4 | 0 | 1 | 24 | 0.960 (0.796 - 0.999) | 1.000 (0.398 - 1.000) | 0.966 (0.822 - 0.999) | 0.970 (0.910 - 1.030) |  | 1.000 (0.858 - 1.000) | 0.800 (0.284 - 0.995) |
|  | Somatic symptom and related disorders | 23 | 17 | 1 | 3 | 2 | 0.400 (0.053 - 0.853) | 0.944 (0.727 - 0.999) | 0.826 (0.612 - 0.950) | 0.689 (0.404 - 0.974) |  | 0.667 (0.094 - 0.992) | 0.850 (0.621 - 0.968) |
|  | Specific learning disorders | 11 | 5 | 1 | 1 | 4 | 0.800 (0.284 - 0.995) | 0.833 (0.359 - 0.996) | 0.818 (0.482 - 0.977) | 0.917 (0.726 - 1.107) |  | 0.800 (0.284 - 0.995) | 0.833 (0.359 - 0.996) |
|  | Substance related and addictive disorders | 389 | 211 | 26 | 14 | 138 | 0.908 (0.850 - 0.949) | 0.890 (0.843 - 0.927) | 0.897 (0.863 - 0.926) | 0.937 (0.909 - 0.965) |  | 0.841 (0.776 - 0.894) | 0.938 (0.898 - 0.966) |
|  | Trauma and stressor-related disorders | 202 | 54 | 19 | 9 | 120 | 0.930 (0.872 - 0.968) | 0.740 (0.624 - 0.835) | 0.861 (0.806 - 0.906) | 0.914 (0.876 - 0.952) |  | 0.863 (0.795 - 0.916) | 0.857 (0.746 - 0.933) |
|  | Psychiatric comorbidity (≥ 2 CAMHD-CS diagnostic groups) | 1866 | 411 | 169 | 109 | 1177 | 0.915 (0.899 - 0.930) | 0.709 (0.670 - 0.745) | 0.851 (0.834 - 0.867) | 0.915 (0.903 - 0.927) |  | 0.874 (0.856 - 0.892) | 0.790 (0.753 - 0.825) |
|  |  |  |  |  |  |  |  |  |  |  |  |  |  |
| **MH dx+**  **ICD/CC** | ADHD | 700 | 223 | 120 | 26 | 331 | 0.927 (0.895 - 0.952) | 0.650 (0.597 - 0.701) | 0.791 (0.759 - 0.821) | 0.897 (0.873 - 0.921) |  | 0.734 (0.691 - 0.774) | 0.896 (0.851 - 0.931) |
|  | Anxiety disorders | 933 | 167 | 192 | 33 | 541 | 0.943 (0.920 - 0.960) | 0.465 (0.413 - 0.518) | 0.759 (0.730 - 0.786) | 0.903 (0.884 - 0.922) |  | 0.738 (0.705 - 0.770) | 0.835 (0.776 - 0.884) |
|  | Autism spectrum disorder | 395 | 139 | 86 | 22 | 148 | 0.871 (0.811 - 0.917) | 0.618 (0.551 - 0.682) | 0.727 (0.680 - 0.770) | 0.816 (0.772 - 0.860) |  | 0.632 (0.567 - 0.694) | 0.863 (0.800 - 0.912) |
|  | Bipolar and related disorders | 144 | 12 | 36 | 8 | 88 | 0.917 (0.842 - 0.963) | 0.250 (0.136 - 0.396) | 0.694 (0.612 - 0.768) | 0.792 (0.719 - 0.864) |  | 0.710 (0.621 - 0.788) | 0.600 (0.361 - 0.809) |
|  | Communication disorders | 23 | 4 | 13 | 0 | 6 | 1.000 (0.541 - 1.000) | 0.235 (0.068 - 0.499) | 0.435 (0.232 - 0.655) | 0.863 (0.662 - 1.063) |  | 0.316 (0.126 - 0.566) | 1.000 (0.398 - 1.000) |
|  | Depressive disorders | 1099 | 6 | 233 | 17 | 843 | 0.980 (0.969 - 0.988) | 0.025 (0.009 - 0.054) | 0.773 (0.747 - 0.797) | 0.879 (0.859 - 0.899) |  | 0.783 (0.758 - 0.808) | 0.261 (0.102 - 0.484) |
|  | Developmental delay or unspecified neurodevelopmental disorder | 56 | 38 | 5 | 2 | 11 | 0.846 (0.546 - 0.981) | 0.884 (0.749 - 0.961) | 0.875 (0.759 - 0.948) | 0.902 (0.786 - 1.017) |  | 0.688 (0.413 - 0.890) | 0.950 (0.831 - 0.994) |
|  | Disruptive, impulse control and conduct disorders | 215 | 11 | 85 | 10 | 109 | 0.916 (0.851 - 0.959) | 0.115 (0.059 - 0.196) | 0.558 (0.489 - 0.626) | 0.618 (0.544 - 0.693) |  | 0.562 (0.489 - 0.633) | 0.524 (0.298 - 0.743) |
|  | Feeding and eating disorders | 86 | 19 | 27 | 6 | 34 | 0.850 (0.702 - 0.943) | 0.413 (0.270 - 0.568) | 0.616 (0.505 - 0.719) | 0.858 (0.776 - 0.940) |  | 0.557 (0.424 - 0.685) | 0.760 (0.549 - 0.906) |
|  | Intellectual disability | 55 | 23 | 11 | 6 | 15 | 0.714 (0.478 - 0.887) | 0.676 (0.495 - 0.826) | 0.691 (0.552 - 0.809) | 0.768 (0.633 - 0.903) |  | 0.577 (0.369 - 0.766) | 0.793 (0.603 - 0.920) |
|  | Mental health symptom | 436 | 1 | 140 | 10 | 285 | 0.966 (0.939 - 0.984) | 0.007 (0.000 - 0.039) | 0.656 (0.609 - 0.701) | 0.661 (0.609 - 0.713) |  | 0.671 (0.624 - 0.715) | 0.091 (0.002 - 0.413) |
|  | Miscellaneous | 158 | 34 | 37 | 7 | 80 | 0.920 (0.841 - 0.967) | 0.479 (0.359 - 0.601) | 0.722 (0.645 - 0.790) | 0.853 (0.794 - 0.911) |  | 0.684 (0.591 - 0.767) | 0.829 (0.679 - 0.928) |
|  | Motor disorders | 36 | 8 | 11 | 1 | 16 | 0.941 (0.713 - 0.999) | 0.421 (0.203 - 0.665) | 0.667 (0.490 - 0.814) | 0.960 (0.891 - 1.029) |  | 0.593 (0.388 - 0.776) | 0.889 (0.518 - 0.997) |
|  | Neurocognitive disorders | 54 | 34 | 10 | 0 | 10 | 1.000 (0.692 - 1.000) | 0.773 (0.622 - 0.885) | 0.815 (0.686 - 0.907) | 0.991 (0.949 - 1.033) |  | 0.500 (0.272 - 0.728) | 1.000 (0.897 - 1.000) |
|  | Obsessive-compulsive and related disorders | 129 | 13 | 27 | 10 | 79 | 0.888 (0.803 - 0.945) | 0.325 (0.186 - 0.491) | 0.713 (0.627 - 0.789) | 0.839 (0.771 - 0.906) |  | 0.745 (0.651 - 0.825) | 0.565 (0.345 - 0.768) |
|  | Personality disorders | 23 | 1 | 4 | 1 | 17 | 0.944 (0.727 - 0.999) | 0.200 (0.005 - 0.716) | 0.783 (0.563 - 0.925) | 0.689 (0.442 - 0.936) |  | 0.810 (0.581 - 0.946) | 0.500 (0.013 - 0.987) |
|  | Schizophrenia spectrum and other psychotic disorders | 116 | 6 | 36 | 6 | 68 | 0.919 (0.832 - 0.970) | 0.143 (0.054 - 0.285) | 0.638 (0.544 - 0.725) | 0.648 (0.547 - 0.749) |  | 0.654 (0.554 - 0.744) | 0.500 (0.211 - 0.789) |
|  | Sexuality and gender identity disorders | 29 | 1 | 3 | 1 | 24 | 0.960 (0.796 - 0.999) | 0.250 (0.006 - 0.806) | 0.862 (0.683 - 0.961) | 0.910 (0.792 - 1.028) |  | 0.889 (0.708 - 0.976) | 0.500 (0.013 - 0.987) |
|  | Somatic symptom and related disorders | 23 | 5 | 13 | 1 | 4 | 0.800 (0.284 - 0.995) | 0.278 (0.097 - 0.535) | 0.391 (0.197 - 0.615) | 0.422 (0.143 - 0.702) |  | 0.235 (0.068 - 0.499) | 0.833 (0.359 - 0.996) |
|  | Specific learning disorders | 11 | 2 | 4 | 0 | 5 | 1.000 (0.478 - 1.000) | 0.333 (0.043 - 0.777) | 0.636 (0.308 - 0.891) | 0.933 (0.762 - 1.104) |  | 0.556 (0.212 - 0.863) | 1.000 (0.158 - 1.000) |
|  | Substance related and addictive disorders | 389 | 159 | 78 | 10 | 142 | 0.934 (0.882 - 0.968) | 0.671 (0.607 - 0.730) | 0.774 (0.729 - 0.814) | 0.921 (0.890 - 0.953) |  | 0.645 (0.578 - 0.709) | 0.941 (0.894 - 0.971) |
|  | Trauma and stressor-related disorders | 202 | 18 | 55 | 11 | 118 | 0.915 (0.853 - 0.957) | 0.247 (0.153 - 0.361) | 0.673 (0.604 - 0.737) | 0.840 (0.787 - 0.893) |  | 0.682 (0.607 - 0.751) | 0.621 (0.423 - 0.793) |
|  | Psychiatric comorbidity (≥ 2 CAMHD-CS diagnostic groups) | 1866 | 150 | 430 | 48 | 1238 | 0.963 (0.951 - 0.972) | 0.259 (0.223 - 0.296) | 0.744 (0.723 - 0.764) | 0.869 (0.854 - 0.885) |  | 0.742 (0.721 - 0.763) | 0.758 (0.692 - 0.816) |
|  |  |  |  |  |  |  |  |  |  |  |  |  |  |
| **aCS** | ADHD | 700 | 221 | 122 | 1 | 356 | 0.997 (0.984 - 1.000) | 0.644 (0.591 - 0.695) | 0.824 (0.794 - 0.852) | 0.934 (0.915 - 0.953) |  | 0.745 (0.703 - 0.783) | 0.995 (0.975 - 1.000) |
|  | Anxiety disorders | 933 | 214 | 145 | 8 | 566 | 0.986 (0.973 - 0.994) | 0.596 (0.543 - 0.647) | 0.836 (0.811 - 0.859) | 0.950 (0.937 - 0.963) |  | 0.796 (0.765 - 0.825) | 0.964 (0.930 - 0.984) |
|  | Autism spectrum disorder | 395 | 139 | 86 | 7 | 163 | 0.959 (0.917 - 0.983) | 0.618 (0.551 - 0.682) | 0.765 (0.720 - 0.806) | 0.902 (0.869 - 0.935) |  | 0.655 (0.592 - 0.714) | 0.952 (0.904 - 0.981) |
|  | Bipolar and related disorders | 144 | 8 | 40 | 2 | 94 | 0.979 (0.927 - 0.997) | 0.167 (0.075 - 0.302) | 0.708 (0.627 - 0.781) | 0.828 (0.762 - 0.893) |  | 0.701 (0.616 - 0.777) | 0.800 (0.444 - 0.975) |
|  | Communication disorders | 23 | 11 | 6 | 0 | 6 | 1.000 (0.541 - 1.000) | 0.647 (0.383 - 0.858) | 0.739 (0.516 - 0.898) | 0.961 (0.848 - 1.074) |  | 0.500 (0.211 - 0.789) | 1.000 (0.715 - 1.000) |
|  | Depressive disorders | 1099 | 123 | 116 | 5 | 855 | 0.994 (0.986 - 0.998) | 0.515 (0.449 - 0.580) | 0.890 (0.870 - 0.908) | 0.942 (0.929 - 0.955) |  | 0.881 (0.858 - 0.900) | 0.961 (0.911 - 0.987) |
|  | Developmental delay or unspecified neurodevelopmental disorder | 56 | 39 | 4 | 0 | 13 | 1.000 (0.753 - 1.000) | 0.907 (0.779 - 0.974) | 0.929 (0.827 - 0.980) | 0.968 (0.899 - 1.036) |  | 0.765 (0.501 - 0.932) | 1.000 (0.910 - 1.000) |
|  | Disruptive, impulse control and conduct disorders | 215 | 13 | 83 | 1 | 118 | 0.992 (0.954 - 1.000) | 0.135 (0.074 - 0.220) | 0.609 (0.541 - 0.675) | 0.743 (0.679 - 0.808) |  | 0.587 (0.516 - 0.656) | 0.929 (0.661 - 0.998) |
|  | Feeding and eating disorders | 86 | 26 | 20 | 0 | 40 | 1.000 (0.912 - 1.000) | 0.565 (0.411 - 0.711) | 0.767 (0.664 - 0.852) | 0.909 (0.843 - 0.976) |  | 0.667 (0.533 - 0.783) | 1.000 (0.868 - 1.000) |
|  | Intellectual disability | 55 | 22 | 12 | 1 | 20 | 0.952 (0.762 - 0.999) | 0.647 (0.465 - 0.803) | 0.764 (0.630 - 0.868) | 0.902 (0.808 - 0.996) |  | 0.625 (0.437 - 0.789) | 0.957 (0.781 - 0.999) |
|  | Mental health symptom | 436 | 21 | 120 | 4 | 291 | 0.986 (0.966 - 0.996) | 0.149 (0.095 - 0.219) | 0.716 (0.671 - 0.758) | 0.841 (0.805 - 0.877) |  | 0.708 (0.661 - 0.752) | 0.840 (0.639 - 0.955) |
|  | Miscellaneous | 158 | 41 | 30 | 4 | 83 | 0.954 (0.886 - 0.987) | 0.577 (0.454 - 0.694) | 0.785 (0.712 - 0.846) | 0.909 (0.862 - 0.955) |  | 0.735 (0.643 - 0.813) | 0.911 (0.788 - 0.975) |
|  | Motor disorders | 36 | 10 | 9 | 2 | 15 | 0.882 (0.636 - 0.985) | 0.526 (0.289 - 0.756) | 0.694 (0.519 - 0.837) | 0.904 (0.798 - 1.010) |  | 0.625 (0.406 - 0.812) | 0.833 (0.516 - 0.979) |
|  | Neurocognitive disorders | 54 | 39 | 5 | 0 | 10 | 1.000 (0.692 - 1.000) | 0.886 (0.754 - 0.962) | 0.907 (0.797 - 0.969) | 0.984 (0.929 - 1.040) |  | 0.667 (0.384 - 0.882) | 1.000 (0.910 - 1.000) |
|  | Obsessive-compulsive and related disorders | 129 | 19 | 21 | 2 | 87 | 0.978 (0.921 - 0.997) | 0.475 (0.315 - 0.639) | 0.822 (0.745 - 0.883) | 0.958 (0.925 - 0.990) |  | 0.806 (0.718 - 0.875) | 0.905 (0.696 - 0.988) |
|  | Personality disorders | 23 | 1 | 4 | 0 | 18 | 1.000 (0.815 - 1.000) | 0.200 (0.005 - 0.716) | 0.826 (0.612 - 0.950) | 0.933 (0.830 - 1.036) |  | 0.818 (0.597 - 0.948) | 1.000 (0.025 - 1.000) |
|  | Schizophrenia spectrum and other psychotic disorders | 116 | 3 | 39 | 1 | 73 | 0.986 (0.927 - 1.000) | 0.071 (0.015 - 0.195) | 0.655 (0.561 - 0.741) | 0.759 (0.672 - 0.845) |  | 0.652 (0.556 - 0.739) | 0.750 (0.194 - 0.994) |
|  | Sexuality and gender identity disorders | 29 | 2 | 2 | 1 | 24 | 0.960 (0.796 - 0.999) | 0.500 (0.068 - 0.932) | 0.897 (0.726 - 0.978) | 0.960 (0.889 - 1.031) |  | 0.923 (0.749 - 0.991) | 0.667 (0.094 - 0.992) |
|  | Somatic symptom and related disorders | 23 | 15 | 3 | 2 | 3 | 0.600 (0.147 - 0.947) | 0.833 (0.586 - 0.964) | 0.783 (0.563 - 0.925) | 0.700 (0.417 - 0.983) |  | 0.500 (0.118 - 0.882) | 0.882 (0.636 - 0.985) |
|  | Specific learning disorders | 11 | 4 | 2 | 0 | 5 | 1.000 (0.478 - 1.000) | 0.667 (0.223 - 0.957) | 0.818 (0.482 - 0.977) | 0.967 (0.845 - 1.088) |  | 0.714 (0.290 - 0.963) | 1.000 (0.398 - 1.000) |
|  | Substance related and addictive disorders | 389 | 189 | 48 | 2 | 150 | 0.987 (0.953 - 0.998) | 0.797 (0.741 - 0.847) | 0.871 (0.834 - 0.903) | 0.971 (0.952 - 0.990) |  | 0.758 (0.692 - 0.816) | 0.990 (0.963 - 0.999) |
|  | Trauma and stressor-related disorders | 202 | 40 | 33 | 0 | 129 | 1.000 (0.972 - 1.000) | 0.548 (0.427 - 0.665) | 0.837 (0.778 - 0.885) | 0.934 (0.901 - 0.967) |  | 0.796 (0.726 - 0.855) | 1.000 (0.912 - 1.000) |
|  | Psychiatric comorbidity (≥ 2 CAMHD-CS diagnostic groups) | 1866 | 262 | 318 | 11 | 1275 | 0.991 (0.985 - 0.996) | 0.452 (0.411 - 0.493) | 0.824 (0.806 - 0.841) | 0.931 (0.920 - 0.942) |  | 0.800 (0.780 - 0.820) | 0.960 (0.929 - 0.980) |
|  |  |  |  |  |  |  |  |  |  |  |  |  |  |
| **NLP-gen** | ADHD | 700 | 228 | 115 | 3 | 354 | 0.992 (0.976 - 0.998) | 0.665 (0.612 - 0.715) | 0.831 (0.802 - 0.858) | 0.932 (0.913 - 0.951) |  | 0.755 (0.713 - 0.793) | 0.987 (0.963 - 0.997) |
|  | Anxiety disorders | 933 | 230 | 129 | 9 | 565 | 0.984 (0.970 - 0.993) | 0.641 (0.589 - 0.690) | 0.852 (0.828 - 0.874) | 0.953 (0.940 - 0.966) |  | 0.814 (0.783 - 0.842) | 0.962 (0.930 - 0.983) |
|  | Autism spectrum disorder | 395 | 147 | 78 | 7 | 163 | 0.959 (0.917 - 0.983) | 0.653 (0.587 - 0.715) | 0.785 (0.741 - 0.824) | 0.899 (0.866 - 0.933) |  | 0.676 (0.613 - 0.735) | 0.955 (0.909 - 0.982) |
|  | Bipolar and related disorders | 144 | 13 | 35 | 2 | 94 | 0.979 (0.927 - 0.997) | 0.271 (0.153 - 0.418) | 0.743 (0.664 - 0.812) | 0.791 (0.719 - 0.864) |  | 0.729 (0.643 - 0.803) | 0.867 (0.595 - 0.983) |
|  | Communication disorders | 23 | 11 | 6 | 1 | 5 | 0.833 (0.359 - 0.996) | 0.647 (0.383 - 0.858) | 0.696 (0.471 - 0.868) | 0.873 (0.678 - 1.067) |  | 0.455 (0.167 - 0.766) | 0.917 (0.615 - 0.998) |
|  | Depressive disorders | 1099 | 143 | 96 | 7 | 853 | 0.992 (0.983 - 0.997) | 0.598 (0.533 - 0.661) | 0.906 (0.887 - 0.923) | 0.934 (0.920 - 0.948) |  | 0.899 (0.878 - 0.917) | 0.953 (0.906 - 0.981) |
|  | Developmental delay or unspecified neurodevelopmental disorder | 56 | 40 | 3 | 1 | 12 | 0.923 (0.640 - 0.998) | 0.930 (0.809 - 0.985) | 0.929 (0.827 - 0.980) | 0.952 (0.868 - 1.035) |  | 0.800 (0.519 - 0.957) | 0.976 (0.871 - 0.999) |
|  | Disruptive, impulse control and conduct disorders | 215 | 17 | 79 | 5 | 114 | 0.958 (0.905 - 0.986) | 0.177 (0.107 - 0.268) | 0.609 (0.541 - 0.675) | 0.730 (0.663 - 0.796) |  | 0.591 (0.518 - 0.661) | 0.773 (0.546 - 0.922) |
|  | Feeding and eating disorders | 86 | 29 | 17 | 4 | 36 | 0.900 (0.763 - 0.972) | 0.630 (0.475 - 0.768) | 0.756 (0.651 - 0.842) | 0.885 (0.811 - 0.960) |  | 0.679 (0.537 - 0.801) | 0.879 (0.718 - 0.966) |
|  | Intellectual disability | 55 | 23 | 11 | 0 | 21 | 1.000 (0.839 - 1.000) | 0.676 (0.495 - 0.826) | 0.800 (0.670 - 0.896) | 0.898 (0.802 - 0.993) |  | 0.656 (0.468 - 0.814) | 1.000 (0.852 - 1.000) |
|  | Mental health symptom | 436 | 26 | 115 | 4 | 291 | 0.986 (0.966 - 0.996) | 0.184 (0.124 - 0.258) | 0.727 (0.683 - 0.768) | 0.791 (0.749 - 0.833) |  | 0.717 (0.670 - 0.760) | 0.867 (0.693 - 0.962) |
|  | Miscellaneous | 158 | 42 | 29 | 2 | 85 | 0.977 (0.919 - 0.997) | 0.592 (0.468 - 0.707) | 0.804 (0.733 - 0.863) | 0.913 (0.868 - 0.958) |  | 0.746 (0.656 - 0.823) | 0.955 (0.845 - 0.994) |
|  | Motor disorders | 36 | 11 | 8 | 1 | 16 | 0.941 (0.713 - 0.999) | 0.579 (0.335 - 0.797) | 0.750 (0.578 - 0.879) | 0.879 (0.761 - 0.998) |  | 0.667 (0.447 - 0.844) | 0.917 (0.615 - 0.998) |
|  | Neurocognitive disorders | 54 | 38 | 6 | 1 | 9 | 0.900 (0.555 - 0.997) | 0.864 (0.726 - 0.948) | 0.870 (0.751 - 0.946) | 0.911 (0.786 - 1.037) |  | 0.600 (0.323 - 0.837) | 0.974 (0.865 - 0.999) |
|  | Obsessive-compulsive and related disorders | 129 | 19 | 21 | 2 | 87 | 0.978 (0.921 - 0.997) | 0.475 (0.315 - 0.639) | 0.822 (0.745 - 0.883) | 0.924 (0.880 - 0.969) |  | 0.806 (0.718 - 0.875) | 0.905 (0.696 - 0.988) |
|  | Personality disorders | 23 | 0 | 5 | 0 | 18 | 1.000 (0.815 - 1.000) | 0.000 (0.000 - 0.522) | 0.783 (0.563 - 0.925) | 0.889 (0.750 - 1.028) |  | 0.783 (0.563 - 0.925) | nan (0.000 - 1.000) |
|  | Schizophrenia spectrum and other psychotic disorders | 116 | 7 | 35 | 2 | 72 | 0.973 (0.906 - 0.997) | 0.167 (0.070 - 0.314) | 0.681 (0.588 - 0.764) | 0.750 (0.663 - 0.838) |  | 0.673 (0.575 - 0.760) | 0.778 (0.400 - 0.972) |
|  | Sexuality and gender identity disorders | 29 | 2 | 2 | 1 | 24 | 0.960 (0.796 - 0.999) | 0.500 (0.068 - 0.932) | 0.897 (0.726 - 0.978) | 0.940 (0.849 - 1.031) |  | 0.923 (0.749 - 0.991) | 0.667 (0.094 - 0.992) |
|  | Somatic symptom and related disorders | 23 | 16 | 2 | 2 | 3 | 0.600 (0.147 - 0.947) | 0.889 (0.653 - 0.986) | 0.826 (0.612 - 0.950) | 0.856 (0.634 - 1.078) |  | 0.600 (0.147 - 0.947) | 0.889 (0.653 - 0.986) |
|  | Specific learning disorders | 11 | 4 | 2 | 0 | 5 | 1.000 (0.478 - 1.000) | 0.667 (0.223 - 0.957) | 0.818 (0.482 - 0.977) | 0.967 (0.845 - 1.088) |  | 0.714 (0.290 - 0.963) | 1.000 (0.398 - 1.000) |
|  | Substance related and addictive disorders | 389 | 177 | 60 | 1 | 151 | 0.993 (0.964 - 1.000) | 0.747 (0.686 - 0.801) | 0.843 (0.803 - 0.878) | 0.972 (0.953 - 0.991) |  | 0.716 (0.650 - 0.775) | 0.994 (0.969 - 1.000) |
|  | Trauma and stressor-related disorders | 202 | 41 | 32 | 3 | 126 | 0.977 (0.934 - 0.995) | 0.562 (0.441 - 0.678) | 0.827 (0.767 - 0.876) | 0.896 (0.853 - 0.938) |  | 0.797 (0.726 - 0.857) | 0.932 (0.813 - 0.986) |
|  | Psychiatric comorbidity (≥ 2 CAMHD-CS diagnostic groups) | 1866 | 306 | 274 | 16 | 1270 | 0.988 (0.980 - 0.993) | 0.528 (0.486 - 0.569) | 0.845 (0.827 - 0.861) | 0.919 (0.907 - 0.931) |  | 0.823 (0.803 - 0.841) | 0.950 (0.921 - 0.971) |
|  |  |  |  |  |  |  |  |  |  |  |  |  |  |
| **NLP-med** | ADHD | 700 | 241 | 102 | 11 | 346 | 0.969 (0.946 - 0.985) | 0.703 (0.651 - 0.751) | 0.839 (0.809 - 0.865) | 0.949 (0.932 - 0.966) |  | 0.772 (0.731 - 0.810) | 0.956 (0.923 - 0.978) |
|  | Anxiety disorders | 933 | 253 | 106 | 13 | 561 | 0.977 (0.962 - 0.988) | 0.705 (0.655 - 0.751) | 0.872 (0.849 - 0.893) | 0.963 (0.951 - 0.974) |  | 0.841 (0.811 - 0.868) | 0.951 (0.918 - 0.974) |
|  | Autism spectrum disorder | 395 | 157 | 68 | 8 | 162 | 0.953 (0.909 - 0.979) | 0.698 (0.633 - 0.757) | 0.808 (0.765 - 0.845) | 0.927 (0.898 - 0.955) |  | 0.704 (0.641 - 0.763) | 0.952 (0.907 - 0.979) |
|  | Bipolar and related disorders | 144 | 17 | 31 | 3 | 93 | 0.969 (0.911 - 0.994) | 0.354 (0.222 - 0.505) | 0.764 (0.686 - 0.831) | 0.911 (0.865 - 0.957) |  | 0.750 (0.664 - 0.823) | 0.850 (0.621 - 0.968) |
|  | Communication disorders | 23 | 13 | 4 | 0 | 6 | 1.000 (0.541 - 1.000) | 0.765 (0.501 - 0.932) | 0.826 (0.612 - 0.950) | 0.936 (0.794 - 1.079) |  | 0.600 (0.262 - 0.878) | 1.000 (0.753 - 1.000) |
|  | Depressive disorders | 1099 | 155 | 84 | 13 | 847 | 0.985 (0.974 - 0.992) | 0.649 (0.584 - 0.709) | 0.912 (0.893 - 0.928) | 0.957 (0.946 - 0.968) |  | 0.910 (0.890 - 0.927) | 0.923 (0.871 - 0.958) |
|  | Developmental delay or unspecified neurodevelopmental disorder | 56 | 40 | 3 | 0 | 13 | 1.000 (0.753 - 1.000) | 0.930 (0.809 - 0.985) | 0.946 (0.851 - 0.989) | 0.986 (0.939 - 1.032) |  | 0.813 (0.544 - 0.960) | 1.000 (0.912 - 1.000) |
|  | Disruptive, impulse control and conduct disorders | 215 | 24 | 72 | 7 | 112 | 0.941 (0.883 - 0.976) | 0.250 (0.167 - 0.349) | 0.633 (0.564 - 0.697) | 0.839 (0.787 - 0.892) |  | 0.609 (0.534 - 0.680) | 0.774 (0.589 - 0.904) |
|  | Feeding and eating disorders | 86 | 30 | 16 | 4 | 36 | 0.900 (0.763 - 0.972) | 0.652 (0.498 - 0.786) | 0.767 (0.664 - 0.852) | 0.910 (0.844 - 0.976) |  | 0.692 (0.549 - 0.813) | 0.882 (0.725 - 0.967) |
|  | Intellectual disability | 55 | 23 | 11 | 0 | 21 | 1.000 (0.839 - 1.000) | 0.676 (0.495 - 0.826) | 0.800 (0.670 - 0.896) | 0.902 (0.808 - 0.996) |  | 0.656 (0.468 - 0.814) | 1.000 (0.852 - 1.000) |
|  | Mental health symptom | 436 | 42 | 99 | 7 | 288 | 0.976 (0.952 - 0.990) | 0.298 (0.224 - 0.381) | 0.757 (0.714 - 0.796) | 0.846 (0.811 - 0.881) |  | 0.744 (0.698 - 0.787) | 0.857 (0.728 - 0.941) |
|  | Miscellaneous | 158 | 46 | 25 | 5 | 82 | 0.943 (0.871 - 0.981) | 0.648 (0.525 - 0.758) | 0.810 (0.740 - 0.868) | 0.938 (0.900 - 0.976) |  | 0.766 (0.675 - 0.843) | 0.902 (0.786 - 0.967) |
|  | Motor disorders | 36 | 11 | 8 | 1 | 16 | 0.941 (0.713 - 0.999) | 0.579 (0.335 - 0.797) | 0.750 (0.578 - 0.879) | 0.940 (0.855 - 1.024) |  | 0.667 (0.447 - 0.844) | 0.917 (0.615 - 0.998) |
|  | Neurocognitive disorders | 54 | 40 | 4 | 1 | 9 | 0.900 (0.555 - 0.997) | 0.909 (0.783 - 0.975) | 0.907 (0.797 - 0.969) | 0.941 (0.837 - 1.045) |  | 0.692 (0.386 - 0.909) | 0.976 (0.871 - 0.999) |
|  | Obsessive-compulsive and related disorders | 129 | 23 | 17 | 3 | 86 | 0.966 (0.905 - 0.993) | 0.575 (0.409 - 0.730) | 0.845 (0.771 - 0.903) | 0.947 (0.911 - 0.983) |  | 0.835 (0.749 - 0.901) | 0.885 (0.698 - 0.976) |
|  | Personality disorders | 23 | 1 | 4 | 0 | 18 | 1.000 (0.815 - 1.000) | 0.200 (0.005 - 0.716) | 0.826 (0.612 - 0.950) | 0.861 (0.703 - 1.020) |  | 0.818 (0.597 - 0.948) | 1.000 (0.025 - 1.000) |
|  | Schizophrenia spectrum and other psychotic disorders | 116 | 11 | 31 | 2 | 72 | 0.973 (0.906 - 0.997) | 0.262 (0.139 - 0.420) | 0.716 (0.624 - 0.795) | 0.823 (0.749 - 0.897) |  | 0.699 (0.601 - 0.785) | 0.846 (0.546 - 0.981) |
|  | Sexuality and gender identity disorders | 29 | 3 | 1 | 1 | 24 | 0.960 (0.796 - 0.999) | 0.750 (0.194 - 0.994) | 0.931 (0.772 - 0.992) | 0.970 (0.910 - 1.030) |  | 0.960 (0.796 - 0.999) | 0.750 (0.194 - 0.994) |
|  | Somatic symptom and related disorders | 23 | 16 | 2 | 2 | 3 | 0.600 (0.147 - 0.947) | 0.889 (0.653 - 0.986) | 0.826 (0.612 - 0.950) | 0.822 (0.582 - 1.063) |  | 0.600 (0.147 - 0.947) | 0.889 (0.653 - 0.986) |
|  | Specific learning disorders | 11 | 4 | 2 | 0 | 5 | 1.000 (0.478 - 1.000) | 0.667 (0.223 - 0.957) | 0.818 (0.482 - 0.977) | 0.933 (0.762 - 1.104) |  | 0.714 (0.290 - 0.963) | 1.000 (0.398 - 1.000) |
|  | Substance related and addictive disorders | 389 | 191 | 46 | 1 | 151 | 0.993 (0.964 - 1.000) | 0.806 (0.750 - 0.854) | 0.879 (0.843 - 0.910) | 0.975 (0.958 - 0.993) |  | 0.766 (0.701 - 0.824) | 0.995 (0.971 - 1.000) |
|  | Trauma and stressor-related disorders | 202 | 43 | 30 | 3 | 126 | 0.977 (0.934 - 0.995) | 0.589 (0.468 - 0.703) | 0.837 (0.778 - 0.885) | 0.944 (0.913 - 0.974) |  | 0.808 (0.737 - 0.866) | 0.935 (0.821 - 0.986) |
|  | Psychiatric comorbidity (≥ 2 CAMHD-CS diagnostic groups) | 1866 | 345 | 235 | 24 | 1262 | 0.981 (0.972 - 0.988) | 0.595 (0.554 - 0.635) | 0.861 (0.845 - 0.877) | 0.946 (0.937 - 0.956) |  | 0.843 (0.824 - 0.861) | 0.935 (0.905 - 0.958) |
|  |  |  |  |  |  |  |  |  |  |  |  |  |  |
| **LLM** | ADHD | 700 | 269 | 74 | 10 | 347 | 0.972 (0.949 - 0.986) | 0.784 (0.737 - 0.827) | 0.880 (0.854 - 0.903) | 0.941 (0.922 - 0.959) |  | 0.824 (0.784 - 0.859) | 0.964 (0.935 - 0.983) |
|  | Anxiety disorders | 933 | 278 | 81 | 8 | 566 | 0.986 (0.973 - 0.994) | 0.774 (0.728 - 0.817) | 0.905 (0.884 - 0.923) | 0.953 (0.940 - 0.966) |  | 0.875 (0.847 - 0.899) | 0.972 (0.946 - 0.988) |
|  | Autism spectrum disorder | 395 | 172 | 53 | 9 | 161 | 0.947 (0.902 - 0.976) | 0.764 (0.703 - 0.818) | 0.843 (0.803 - 0.877) | 0.924 (0.895 - 0.953) |  | 0.752 (0.689 - 0.809) | 0.950 (0.908 - 0.977) |
|  | Bipolar and related disorders | 144 | 16 | 32 | 5 | 91 | 0.948 (0.883 - 0.983) | 0.333 (0.204 - 0.484) | 0.743 (0.664 - 0.812) | 0.867 (0.810 - 0.924) |  | 0.740 (0.653 - 0.815) | 0.762 (0.528 - 0.918) |
|  | Communication disorders | 23 | 13 | 4 | 1 | 5 | 0.833 (0.359 - 0.996) | 0.765 (0.501 - 0.932) | 0.783 (0.563 - 0.925) | 0.936 (0.794 - 1.079) |  | 0.556 (0.212 - 0.863) | 0.929 (0.661 - 0.998) |
|  | Depressive disorders | 1099 | 160 | 79 | 8 | 852 | 0.991 (0.982 - 0.996) | 0.669 (0.606 - 0.729) | 0.921 (0.903 - 0.936) | 0.942 (0.929 - 0.955) |  | 0.915 (0.895 - 0.932) | 0.952 (0.908 - 0.979) |
|  | Developmental delay or unspecified neurodevelopmental disorder | 56 | 40 | 3 | 1 | 12 | 0.923 (0.640 - 0.998) | 0.930 (0.809 - 0.985) | 0.929 (0.827 - 0.980) | 0.927 (0.825 - 1.028) |  | 0.800 (0.519 - 0.957) | 0.976 (0.871 - 0.999) |
|  | Disruptive, impulse control and conduct disorders | 215 | 49 | 47 | 3 | 116 | 0.975 (0.928 - 0.995) | 0.510 (0.406 - 0.614) | 0.767 (0.705 - 0.822) | 0.859 (0.810 - 0.908) |  | 0.712 (0.636 - 0.780) | 0.942 (0.841 - 0.988) |
|  | Feeding and eating disorders | 86 | 35 | 11 | 3 | 37 | 0.925 (0.796 - 0.984) | 0.761 (0.612 - 0.874) | 0.837 (0.742 - 0.908) | 0.898 (0.828 - 0.969) |  | 0.771 (0.627 - 0.880) | 0.921 (0.786 - 0.983) |
|  | Intellectual disability | 55 | 28 | 6 | 1 | 20 | 0.952 (0.762 - 0.999) | 0.824 (0.655 - 0.932) | 0.873 (0.755 - 0.947) | 0.945 (0.875 - 1.016) |  | 0.769 (0.564 - 0.910) | 0.966 (0.822 - 0.999) |
|  | Mental health symptom | 436 | 66 | 75 | 8 | 287 | 0.973 (0.947 - 0.988) | 0.468 (0.384 - 0.554) | 0.810 (0.770 - 0.845) | 0.856 (0.822 - 0.890) |  | 0.793 (0.747 - 0.833) | 0.892 (0.798 - 0.952) |
|  | Miscellaneous | 158 | 52 | 19 | 4 | 83 | 0.954 (0.886 - 0.987) | 0.732 (0.614 - 0.831) | 0.854 (0.790 - 0.905) | 0.918 (0.874 - 0.962) |  | 0.814 (0.724 - 0.884) | 0.929 (0.827 - 0.980) |
|  | Motor disorders | 36 | 17 | 2 | 1 | 16 | 0.941 (0.713 - 0.999) | 0.895 (0.669 - 0.987) | 0.917 (0.775 - 0.982) | 0.980 (0.931 - 1.029) |  | 0.889 (0.653 - 0.986) | 0.944 (0.727 - 0.999) |
|  | Neurocognitive disorders | 54 | 41 | 3 | 1 | 9 | 0.900 (0.555 - 0.997) | 0.932 (0.813 - 0.986) | 0.926 (0.821 - 0.979) | 0.900 (0.768 - 1.032) |  | 0.750 (0.428 - 0.945) | 0.976 (0.874 - 0.999) |
|  | Obsessive-compulsive and related disorders | 129 | 30 | 10 | 3 | 86 | 0.966 (0.905 - 0.993) | 0.750 (0.588 - 0.873) | 0.899 (0.834 - 0.945) | 0.949 (0.913 - 0.984) |  | 0.896 (0.817 - 0.949) | 0.909 (0.757 - 0.981) |
|  | Personality disorders | 23 | 2 | 3 | 0 | 18 | 1.000 (0.815 - 1.000) | 0.400 (0.053 - 0.853) | 0.870 (0.664 - 0.972) | 1.000 (1.000 - 1.000) |  | 0.857 (0.637 - 0.970) | 1.000 (0.158 - 1.000) |
|  | Schizophrenia spectrum and other psychotic disorders | 116 | 14 | 28 | 1 | 73 | 0.986 (0.927 - 1.000) | 0.333 (0.196 - 0.495) | 0.750 (0.661 - 0.826) | 0.829 (0.756 - 0.902) |  | 0.723 (0.625 - 0.807) | 0.933 (0.681 - 0.998) |
|  | Sexuality and gender identity disorders | 29 | 3 | 1 | 0 | 25 | 1.000 (0.863 - 1.000) | 0.750 (0.194 - 0.994) | 0.966 (0.822 - 0.999) | 0.960 (0.889 - 1.031) |  | 0.962 (0.804 - 0.999) | 1.000 (0.292 - 1.000) |
|  | Somatic symptom and related disorders | 23 | 17 | 1 | 1 | 4 | 0.800 (0.284 - 0.995) | 0.944 (0.727 - 0.999) | 0.913 (0.720 - 0.989) | 0.783 (0.525 - 1.041) |  | 0.800 (0.284 - 0.995) | 0.944 (0.727 - 0.999) |
|  | Specific learning disorders | 11 | 6 | 0 | 1 | 4 | 0.800 (0.284 - 0.995) | 1.000 (0.541 - 1.000) | 0.909 (0.587 - 0.998) | 0.950 (0.801 - 1.099) |  | 1.000 (0.398 - 1.000) | 0.857 (0.421 - 0.996) |
|  | Substance related and addictive disorders | 389 | 192 | 45 | 2 | 150 | 0.987 (0.953 - 0.998) | 0.810 (0.754 - 0.858) | 0.879 (0.843 - 0.910) | 0.958 (0.935 - 0.981) |  | 0.769 (0.704 - 0.826) | 0.990 (0.963 - 0.999) |
|  | Trauma and stressor-related disorders | 202 | 45 | 28 | 3 | 126 | 0.977 (0.934 - 0.995) | 0.616 (0.495 - 0.728) | 0.847 (0.789 - 0.893) | 0.917 (0.880 - 0.955) |  | 0.818 (0.748 - 0.876) | 0.938 (0.828 - 0.987) |
|  | Psychiatric comorbidity (≥ 2 CAMHD-CS diagnostic groups) | 1866 | 400 | 180 | 19 | 1267 | 0.985 (0.977 - 0.991) | 0.690 (0.650 - 0.727) | 0.893 (0.878 - 0.907) | 0.931 (0.921 - 0.942) |  | 0.876 (0.857 - 0.892) | 0.955 (0.930 - 0.972) |
|  |  |  |  |  |  |  |  |  |  |  |  |  |  |
| **NLP-gen+aCS** | ADHD | 700 | 228 | 115 | 1 | 356 | 0.997 (0.984 - 1.000) | 0.665 (0.612 - 0.715) | 0.834 (0.805 - 0.861) | 0.950 (0.933 - 0.966) |  | 0.756 (0.714 - 0.794) | 0.996 (0.976 - 1.000) |
|  | Anxiety disorders | 933 | 231 | 128 | 6 | 568 | 0.990 (0.977 - 0.996) | 0.643 (0.591 - 0.693) | 0.856 (0.832 - 0.878) | 0.964 (0.953 - 0.975) |  | 0.816 (0.785 - 0.844) | 0.975 (0.946 - 0.991) |
|  | Autism spectrum disorder | 395 | 144 | 81 | 6 | 164 | 0.965 (0.925 - 0.987) | 0.640 (0.574 - 0.703) | 0.780 (0.736 - 0.820) | 0.915 (0.885 - 0.946) |  | 0.669 (0.607 - 0.728) | 0.960 (0.915 - 0.985) |
|  | Bipolar and related disorders | 144 | 9 | 39 | 1 | 95 | 0.990 (0.943 - 1.000) | 0.188 (0.089 - 0.326) | 0.722 (0.642 - 0.794) | 0.844 (0.782 - 0.907) |  | 0.709 (0.624 - 0.784) | 0.900 (0.555 - 0.997) |
|  | Communication disorders | 23 | 11 | 6 | 0 | 6 | 1.000 (0.541 - 1.000) | 0.647 (0.383 - 0.858) | 0.739 (0.516 - 0.898) | 0.912 (0.746 - 1.077) |  | 0.500 (0.211 - 0.789) | 1.000 (0.715 - 1.000) |
|  | Depressive disorders | 1099 | 142 | 97 | 5 | 855 | 0.994 (0.986 - 0.998) | 0.594 (0.529 - 0.657) | 0.907 (0.888 - 0.924) | 0.960 (0.949 - 0.970) |  | 0.898 (0.877 - 0.917) | 0.966 (0.922 - 0.989) |
|  | Developmental delay or unspecified neurodevelopmental disorder | 56 | 40 | 3 | 0 | 13 | 1.000 (0.753 - 1.000) | 0.930 (0.809 - 0.985) | 0.946 (0.851 - 0.989) | 0.966 (0.896 - 1.037) |  | 0.813 (0.544 - 0.960) | 1.000 (0.912 - 1.000) |
|  | Disruptive, impulse control and conduct disorders | 215 | 15 | 81 | 2 | 117 | 0.983 (0.941 - 0.998) | 0.156 (0.090 - 0.245) | 0.614 (0.545 - 0.679) | 0.752 (0.688 - 0.816) |  | 0.591 (0.519 - 0.660) | 0.882 (0.636 - 0.985) |
|  | Feeding and eating disorders | 86 | 28 | 18 | 2 | 38 | 0.950 (0.831 - 0.994) | 0.609 (0.454 - 0.749) | 0.767 (0.664 - 0.852) | 0.921 (0.859 - 0.983) |  | 0.679 (0.540 - 0.797) | 0.933 (0.779 - 0.992) |
|  | Intellectual disability | 55 | 23 | 11 | 0 | 21 | 1.000 (0.839 - 1.000) | 0.676 (0.495 - 0.826) | 0.800 (0.670 - 0.896) | 0.899 (0.804 - 0.994) |  | 0.656 (0.468 - 0.814) | 1.000 (0.852 - 1.000) |
|  | Mental health symptom | 436 | 27 | 114 | 3 | 292 | 0.990 (0.971 - 0.998) | 0.191 (0.130 - 0.266) | 0.732 (0.687 - 0.773) | 0.862 (0.829 - 0.895) |  | 0.719 (0.673 - 0.762) | 0.900 (0.735 - 0.979) |
|  | Miscellaneous | 158 | 41 | 30 | 2 | 85 | 0.977 (0.919 - 0.997) | 0.577 (0.454 - 0.694) | 0.797 (0.726 - 0.857) | 0.937 (0.898 - 0.975) |  | 0.739 (0.649 - 0.817) | 0.953 (0.842 - 0.994) |
|  | Motor disorders | 36 | 12 | 7 | 1 | 16 | 0.941 (0.713 - 0.999) | 0.632 (0.384 - 0.837) | 0.778 (0.608 - 0.899) | 0.941 (0.858 - 1.025) |  | 0.696 (0.471 - 0.868) | 0.923 (0.640 - 0.998) |
|  | Neurocognitive disorders | 54 | 39 | 5 | 1 | 9 | 0.900 (0.555 - 0.997) | 0.886 (0.754 - 0.962) | 0.889 (0.774 - 0.958) | 0.986 (0.935 - 1.038) |  | 0.643 (0.351 - 0.872) | 0.975 (0.868 - 0.999) |
|  | Obsessive-compulsive and related disorders | 129 | 20 | 20 | 2 | 87 | 0.978 (0.921 - 0.997) | 0.500 (0.338 - 0.662) | 0.829 (0.753 - 0.890) | 0.948 (0.912 - 0.984) |  | 0.813 (0.726 - 0.882) | 0.909 (0.708 - 0.989) |
|  | Personality disorders | 23 | 1 | 4 | 0 | 18 | 1.000 (0.815 - 1.000) | 0.200 (0.005 - 0.716) | 0.826 (0.612 - 0.950) | 0.889 (0.750 - 1.028) |  | 0.818 (0.597 - 0.948) | 1.000 (0.025 - 1.000) |
|  | Schizophrenia spectrum and other psychotic disorders | 116 | 6 | 36 | 1 | 73 | 0.986 (0.927 - 1.000) | 0.143 (0.054 - 0.285) | 0.681 (0.588 - 0.764) | 0.801 (0.722 - 0.879) |  | 0.670 (0.573 - 0.757) | 0.857 (0.421 - 0.996) |
|  | Sexuality and gender identity disorders | 29 | 3 | 1 | 1 | 24 | 0.960 (0.796 - 0.999) | 0.750 (0.194 - 0.994) | 0.931 (0.772 - 0.992) | 0.980 (0.933 - 1.027) |  | 0.960 (0.796 - 0.999) | 0.750 (0.194 - 0.994) |
|  | Somatic symptom and related disorders | 23 | 16 | 2 | 2 | 3 | 0.600 (0.147 - 0.947) | 0.889 (0.653 - 0.986) | 0.826 (0.612 - 0.950) | 0.678 (0.391 - 0.965) |  | 0.600 (0.147 - 0.947) | 0.889 (0.653 - 0.986) |
|  | Specific learning disorders | 11 | 4 | 2 | 0 | 5 | 1.000 (0.478 - 1.000) | 0.667 (0.223 - 0.957) | 0.818 (0.482 - 0.977) | 0.967 (0.845 - 1.088) |  | 0.714 (0.290 - 0.963) | 1.000 (0.398 - 1.000) |
|  | Substance related and addictive disorders | 389 | 190 | 47 | 1 | 151 | 0.993 (0.964 - 1.000) | 0.802 (0.745 - 0.850) | 0.877 (0.840 - 0.908) | 0.978 (0.961 - 0.995) |  | 0.763 (0.697 - 0.820) | 0.995 (0.971 - 1.000) |
|  | Trauma and stressor-related disorders | 202 | 40 | 33 | 2 | 127 | 0.984 (0.945 - 0.998) | 0.548 (0.427 - 0.665) | 0.827 (0.767 - 0.876) | 0.939 (0.908 - 0.971) |  | 0.794 (0.723 - 0.854) | 0.952 (0.838 - 0.994) |
|  | Psychiatric comorbidity (≥ 2 CAMHD-CS diagnostic groups) | 1866 | 302 | 278 | 9 | 1277 | 0.993 (0.987 - 0.997) | 0.521 (0.479 - 0.562) | 0.846 (0.829 - 0.862) | 0.946 (0.937 - 0.956) |  | 0.821 (0.801 - 0.840) | 0.971 (0.946 - 0.987) |
|  |  |  |  |  |  |  |  |  |  |  |  |  |  |
| **NLP-med+aCS** | ADHD | 700 | 235 | 108 | 6 | 351 | 0.983 (0.964 - 0.994) | 0.685 (0.633 - 0.734) | 0.837 (0.808 - 0.864) | 0.950 (0.933 - 0.967) |  | 0.765 (0.723 - 0.803) | 0.975 (0.947 - 0.991) |
|  | Anxiety disorders | 933 | 244 | 115 | 11 | 563 | 0.981 (0.966 - 0.990) | 0.680 (0.629 - 0.728) | 0.865 (0.841 - 0.886) | 0.962 (0.951 - 0.974) |  | 0.830 (0.800 - 0.858) | 0.957 (0.924 - 0.978) |
|  | Autism spectrum disorder | 395 | 153 | 72 | 8 | 162 | 0.953 (0.909 - 0.979) | 0.680 (0.615 - 0.740) | 0.797 (0.754 - 0.836) | 0.912 (0.881 - 0.943) |  | 0.692 (0.629 - 0.751) | 0.950 (0.904 - 0.978) |
|  | Bipolar and related disorders | 144 | 15 | 33 | 2 | 94 | 0.979 (0.927 - 0.997) | 0.313 (0.187 - 0.463) | 0.757 (0.679 - 0.824) | 0.876 (0.821 - 0.931) |  | 0.740 (0.655 - 0.814) | 0.882 (0.636 - 0.985) |
|  | Communication disorders | 23 | 13 | 4 | 0 | 6 | 1.000 (0.541 - 1.000) | 0.765 (0.501 - 0.932) | 0.826 (0.612 - 0.950) | 0.922 (0.765 - 1.078) |  | 0.600 (0.262 - 0.878) | 1.000 (0.753 - 1.000) |
|  | Depressive disorders | 1099 | 152 | 87 | 9 | 851 | 0.990 (0.980 - 0.995) | 0.636 (0.572 - 0.697) | 0.913 (0.894 - 0.929) | 0.964 (0.954 - 0.974) |  | 0.907 (0.887 - 0.925) | 0.944 (0.897 - 0.974) |
|  | Developmental delay or unspecified neurodevelopmental disorder | 56 | 40 | 3 | 0 | 13 | 1.000 (0.753 - 1.000) | 0.930 (0.809 - 0.985) | 0.946 (0.851 - 0.989) | 0.950 (0.865 - 1.035) |  | 0.813 (0.544 - 0.960) | 1.000 (0.912 - 1.000) |
|  | Disruptive, impulse control and conduct disorders | 215 | 26 | 70 | 4 | 115 | 0.966 (0.916 - 0.991) | 0.271 (0.185 - 0.371) | 0.656 (0.588 - 0.719) | 0.816 (0.760 - 0.872) |  | 0.622 (0.548 - 0.692) | 0.867 (0.693 - 0.962) |
|  | Feeding and eating disorders | 86 | 31 | 15 | 2 | 38 | 0.950 (0.831 - 0.994) | 0.674 (0.520 - 0.805) | 0.802 (0.702 - 0.880) | 0.921 (0.858 - 0.983) |  | 0.717 (0.577 - 0.832) | 0.939 (0.798 - 0.993) |
|  | Intellectual disability | 55 | 26 | 8 | 1 | 20 | 0.952 (0.762 - 0.999) | 0.765 (0.588 - 0.893) | 0.836 (0.712 - 0.922) | 0.913 (0.825 - 1.002) |  | 0.714 (0.513 - 0.868) | 0.963 (0.810 - 0.999) |
|  | Mental health symptom | 436 | 40 | 101 | 4 | 291 | 0.986 (0.966 - 0.996) | 0.284 (0.211 - 0.366) | 0.759 (0.716 - 0.799) | 0.875 (0.843 - 0.906) |  | 0.742 (0.696 - 0.785) | 0.909 (0.783 - 0.975) |
|  | Miscellaneous | 158 | 43 | 28 | 4 | 83 | 0.954 (0.886 - 0.987) | 0.606 (0.483 - 0.720) | 0.797 (0.726 - 0.857) | 0.940 (0.903 - 0.978) |  | 0.748 (0.656 - 0.825) | 0.915 (0.796 - 0.976) |
|  | Motor disorders | 36 | 11 | 8 | 1 | 16 | 0.941 (0.713 - 0.999) | 0.579 (0.335 - 0.797) | 0.750 (0.578 - 0.879) | 0.944 (0.863 - 1.026) |  | 0.667 (0.447 - 0.844) | 0.917 (0.615 - 0.998) |
|  | Neurocognitive disorders | 54 | 41 | 3 | 1 | 9 | 0.900 (0.555 - 0.997) | 0.932 (0.813 - 0.986) | 0.926 (0.821 - 0.979) | 0.993 (0.957 - 1.030) |  | 0.750 (0.428 - 0.945) | 0.976 (0.874 - 0.999) |
|  | Obsessive-compulsive and related disorders | 129 | 21 | 19 | 2 | 87 | 0.978 (0.921 - 0.997) | 0.525 (0.361 - 0.685) | 0.837 (0.762 - 0.896) | 0.942 (0.903 - 0.980) |  | 0.821 (0.734 - 0.888) | 0.913 (0.720 - 0.989) |
|  | Personality disorders | 23 | 1 | 4 | 0 | 18 | 1.000 (0.815 - 1.000) | 0.200 (0.005 - 0.716) | 0.826 (0.612 - 0.950) | 0.944 (0.852 - 1.037) |  | 0.818 (0.597 - 0.948) | 1.000 (0.025 - 1.000) |
|  | Schizophrenia spectrum and other psychotic disorders | 116 | 8 | 34 | 1 | 73 | 0.986 (0.927 - 1.000) | 0.190 (0.086 - 0.341) | 0.698 (0.606 - 0.780) | 0.809 (0.732 - 0.886) |  | 0.682 (0.585 - 0.769) | 0.889 (0.518 - 0.997) |
|  | Sexuality and gender identity disorders | 29 | 3 | 1 | 1 | 24 | 0.960 (0.796 - 0.999) | 0.750 (0.194 - 0.994) | 0.931 (0.772 - 0.992) | 0.970 (0.910 - 1.030) |  | 0.960 (0.796 - 0.999) | 0.750 (0.194 - 0.994) |
|  | Somatic symptom and related disorders | 23 | 17 | 1 | 2 | 3 | 0.600 (0.147 - 0.947) | 0.944 (0.727 - 0.999) | 0.870 (0.664 - 0.972) | 0.733 (0.459 - 1.008) |  | 0.750 (0.194 - 0.994) | 0.895 (0.669 - 0.987) |
|  | Specific learning disorders | 11 | 5 | 1 | 0 | 5 | 1.000 (0.478 - 1.000) | 0.833 (0.359 - 0.996) | 0.909 (0.587 - 0.998) | 0.967 (0.845 - 1.088) |  | 0.833 (0.359 - 0.996) | 1.000 (0.478 - 1.000) |
|  | Substance related and addictive disorders | 389 | 194 | 43 | 2 | 150 | 0.987 (0.953 - 0.998) | 0.819 (0.763 - 0.865) | 0.884 (0.848 - 0.914) | 0.976 (0.959 - 0.994) |  | 0.777 (0.712 - 0.834) | 0.990 (0.964 - 0.999) |
|  | Trauma and stressor-related disorders | 202 | 42 | 31 | 2 | 127 | 0.984 (0.945 - 0.998) | 0.575 (0.454 - 0.690) | 0.837 (0.778 - 0.885) | 0.948 (0.919 - 0.977) |  | 0.804 (0.733 - 0.863) | 0.955 (0.845 - 0.994) |
|  | Psychiatric comorbidity (≥ 2 CAMHD-CS diagnostic groups) | 1866 | 331 | 249 | 15 | 1271 | 0.988 (0.981 - 0.993) | 0.571 (0.529 - 0.611) | 0.859 (0.842 - 0.874) | 0.948 (0.938 - 0.957) |  | 0.836 (0.817 - 0.854) | 0.957 (0.930 - 0.976) |
|  |  |  |  |  |  |  |  |  |  |  |  |  |  |
| **LLM+aCS** | ADHD | 700 | 268 | 75 | 9 | 348 | 0.975 (0.953 - 0.988) | 0.781 (0.734 - 0.824) | 0.880 (0.854 - 0.903) | 0.956 (0.941 - 0.972) |  | 0.823 (0.783 - 0.858) | 0.968 (0.939 - 0.985) |
|  | Anxiety disorders | 933 | 278 | 81 | 11 | 563 | 0.981 (0.966 - 0.990) | 0.774 (0.728 - 0.817) | 0.901 (0.880 - 0.920) | 0.970 (0.960 - 0.980) |  | 0.874 (0.846 - 0.899) | 0.962 (0.933 - 0.981) |
|  | Autism spectrum disorder | 395 | 164 | 61 | 8 | 162 | 0.953 (0.909 - 0.979) | 0.729 (0.666 - 0.786) | 0.825 (0.784 - 0.861) | 0.936 (0.909 - 0.963) |  | 0.726 (0.663 - 0.784) | 0.953 (0.910 - 0.980) |
|  | Bipolar and related disorders | 144 | 17 | 31 | 3 | 93 | 0.969 (0.911 - 0.994) | 0.354 (0.222 - 0.505) | 0.764 (0.686 - 0.831) | 0.891 (0.840 - 0.943) |  | 0.750 (0.664 - 0.823) | 0.850 (0.621 - 0.968) |
|  | Communication disorders | 23 | 13 | 4 | 0 | 6 | 1.000 (0.541 - 1.000) | 0.765 (0.501 - 0.932) | 0.826 (0.612 - 0.950) | 0.961 (0.848 - 1.074) |  | 0.600 (0.262 - 0.878) | 1.000 (0.753 - 1.000) |
|  | Depressive disorders | 1099 | 162 | 77 | 11 | 849 | 0.987 (0.977 - 0.994) | 0.678 (0.615 - 0.737) | 0.920 (0.902 - 0.935) | 0.963 (0.953 - 0.973) |  | 0.917 (0.897 - 0.934) | 0.936 (0.889 - 0.968) |
|  | Developmental delay or unspecified neurodevelopmental disorder | 56 | 40 | 3 | 1 | 12 | 0.923 (0.640 - 0.998) | 0.930 (0.809 - 0.985) | 0.929 (0.827 - 0.980) | 0.973 (0.910 - 1.036) |  | 0.800 (0.519 - 0.957) | 0.976 (0.871 - 0.999) |
|  | Disruptive, impulse control and conduct disorders | 215 | 49 | 47 | 2 | 117 | 0.983 (0.941 - 0.998) | 0.510 (0.406 - 0.614) | 0.772 (0.710 - 0.826) | 0.858 (0.809 - 0.908) |  | 0.713 (0.638 - 0.781) | 0.961 (0.865 - 0.995) |
|  | Feeding and eating disorders | 86 | 36 | 10 | 3 | 37 | 0.925 (0.796 - 0.984) | 0.783 (0.636 - 0.891) | 0.849 (0.755 - 0.917) | 0.952 (0.903 - 1.000) |  | 0.787 (0.643 - 0.893) | 0.923 (0.791 - 0.984) |
|  | Intellectual disability | 55 | 27 | 7 | 1 | 20 | 0.952 (0.762 - 0.999) | 0.794 (0.621 - 0.913) | 0.855 (0.733 - 0.935) | 0.933 (0.854 - 1.011) |  | 0.741 (0.537 - 0.889) | 0.964 (0.817 - 0.999) |
|  | Mental health symptom | 436 | 55 | 86 | 7 | 288 | 0.976 (0.952 - 0.990) | 0.390 (0.309 - 0.476) | 0.787 (0.745 - 0.824) | 0.880 (0.849 - 0.911) |  | 0.770 (0.724 - 0.812) | 0.887 (0.781 - 0.953) |
|  | Miscellaneous | 158 | 54 | 17 | 5 | 82 | 0.943 (0.871 - 0.981) | 0.761 (0.645 - 0.854) | 0.861 (0.797 - 0.911) | 0.939 (0.901 - 0.976) |  | 0.828 (0.739 - 0.897) | 0.915 (0.813 - 0.972) |
|  | Motor disorders | 36 | 18 | 1 | 1 | 16 | 0.941 (0.713 - 0.999) | 0.947 (0.740 - 0.999) | 0.944 (0.813 - 0.993) | 0.991 (0.957 - 1.024) |  | 0.941 (0.713 - 0.999) | 0.947 (0.740 - 0.999) |
|  | Neurocognitive disorders | 54 | 42 | 2 | 1 | 9 | 0.900 (0.555 - 0.997) | 0.955 (0.845 - 0.994) | 0.944 (0.846 - 0.988) | 0.973 (0.901 - 1.045) |  | 0.818 (0.482 - 0.977) | 0.977 (0.877 - 0.999) |
|  | Obsessive-compulsive and related disorders | 129 | 30 | 10 | 3 | 86 | 0.966 (0.905 - 0.993) | 0.750 (0.588 - 0.873) | 0.899 (0.834 - 0.945) | 0.967 (0.939 - 0.995) |  | 0.896 (0.817 - 0.949) | 0.909 (0.757 - 0.981) |
|  | Personality disorders | 23 | 2 | 3 | 0 | 18 | 1.000 (0.815 - 1.000) | 0.400 (0.053 - 0.853) | 0.870 (0.664 - 0.972) | 1.000 (1.000 - 1.000) |  | 0.857 (0.637 - 0.970) | 1.000 (0.158 - 1.000) |
|  | Schizophrenia spectrum and other psychotic disorders | 116 | 14 | 28 | 1 | 73 | 0.986 (0.927 - 1.000) | 0.333 (0.196 - 0.495) | 0.750 (0.661 - 0.826) | 0.867 (0.803 - 0.931) |  | 0.723 (0.625 - 0.807) | 0.933 (0.681 - 0.998) |
|  | Sexuality and gender identity disorders | 29 | 3 | 1 | 0 | 25 | 1.000 (0.863 - 1.000) | 0.750 (0.194 - 0.994) | 0.966 (0.822 - 0.999) | 0.970 (0.910 - 1.030) |  | 0.962 (0.804 - 0.999) | 1.000 (0.292 - 1.000) |
|  | Somatic symptom and related disorders | 23 | 17 | 1 | 2 | 3 | 0.600 (0.147 - 0.947) | 0.944 (0.727 - 0.999) | 0.870 (0.664 - 0.972) | 0.767 (0.503 - 1.031) |  | 0.750 (0.194 - 0.994) | 0.895 (0.669 - 0.987) |
|  | Specific learning disorders | 11 | 6 | 0 | 0 | 5 | 1.000 (0.478 - 1.000) | 1.000 (0.541 - 1.000) | 1.000 (0.715 - 1.000) | 1.000 (1.000 - 1.000) |  | 1.000 (0.478 - 1.000) | 1.000 (0.541 - 1.000) |
|  | Substance related and addictive disorders | 389 | 197 | 40 | 4 | 148 | 0.974 (0.934 - 0.993) | 0.831 (0.777 - 0.877) | 0.887 (0.851 - 0.917) | 0.974 (0.956 - 0.992) |  | 0.787 (0.722 - 0.843) | 0.980 (0.950 - 0.995) |
|  | Trauma and stressor-related disorders | 202 | 45 | 28 | 5 | 124 | 0.961 (0.912 - 0.987) | 0.616 (0.495 - 0.728) | 0.837 (0.778 - 0.885) | 0.936 (0.903 - 0.969) |  | 0.816 (0.745 - 0.874) | 0.900 (0.782 - 0.967) |
|  | Psychiatric comorbidity (≥ 2 CAMHD-CS diagnostic groups) | 1866 | 394 | 186 | 20 | 1266 | 0.984 (0.976 - 0.990) | 0.679 (0.640 - 0.717) | 0.890 (0.874 - 0.903) | 0.957 (0.948 - 0.965) |  | 0.872 (0.854 - 0.889) | 0.952 (0.926 - 0.970) |

*Abbreviations: ADHD: Attention Deficit/Hyperactivity Disorder; CAMHD-CS: Child and Adolescent Mental Health Disorders Classification System.*

*The feature set notation is as follows: ICD/CC refers to a feature set based on International Classification of Diseases, Clinical Modification, Version 10, codes for non-fatal suicide attempt and intentional self-harm, as defined by the Centers for Disease Control and Prevention Case Surveillance definition list, plus suicide-related chief concern; c-SSRS+ICD/CC combines ICD/CC with c-SSRS item scores; MH dx+ICD/CC combines ICD/CC with Child and Adolescent Mental Health Disorders Classification System ICD-10-CM code categories; aCS represents all available structured data; NLP-gen and NLP-med are feature sets based on vectorized text features with embeddings derived from the Universal Sentence Encoder and MedEmbed, respectively; LLM refers to Likert-type scores generated by the open-source language model llama-3.3-70B. Feature sets denoted by (aCS+) indicate combinations of aCS with the corresponding text-based feature set (NLP-gen, NLP-med, or LLM).*
