# Supplementary material for: Detecting Pediatric Emergency Service Use for Suicide and Self-Harm: Multimodal Analysis of 3828 Encounters
Source: JMIR Ment Health. 2026 Feb 4;13:e82371. doi: 10.2196/82371 (PMC12871580; doi:10.2196/82371)
Supplement: Checklist 1 [file mental-v13-e82371-s020.docx]

# **Checklist 1 –** STROBE and TRIPOD Checklists

# **STROBE Checklist**

# *Von Elm, E., Altman, D. G., Egger, M., Pocock, S. J., Gøtzsche, P. C., & Vandenbroucke, J. P. (2007). The Strengthening the Reporting of Observational Studies in Epidemiology (STROBE) statement: guidelines for reporting observational studies. The Lancet, 370(9596), 1453-1457.*

|  | Item No | Recommendation |
| --- | --- | --- |
| **Title and abstract** | 1 | (*a*) Title includes design (cross-sectional) |
|  |  | (*b*) Abstract |
| Introduction | | |
| Background/rationale | 2 | Introduction, paragraphs 1-2 |
| Objectives | 3 | Introduction, paragraph 3 |
| Methods | | |
| Study design | 4 | Method (Study Design, paragraph 1) |
| Setting | 5 | Method (Study Design, paragraph 1); Flow Diagram: eFigure 1 |
| Participants | 6 | Method (Study Design, paragraph 1); Flow Diagram: eFigure 1 |
| Variables | 7 | Method, Data Sources & Variables. Supplemental content: on variable construction in eMethod 2, on human annotator coding in eMethod 3 chart annotation guide, on variables comprising feature sets in eTable 3 |
| Data sources/ measurement | 8* | Method, Data Sources & Variables. |
| Bias | 9 | Prevention of data leakage or overfitting described in Methods, Encounter Classification; subgroup variation for assessment of bias in classification described in Methods, Statistical Analysis; community advisory board review of LLM output described in Supplement eMethod 5. |
| Study size | 10 | Method (Study Design, paragraph 1); Flow Diagram: eFigure 1 |
| Quantitative variables | 11 | Explain how quantitative variables were handled in the analyses. If applicable, describe which groupings were chosen and why (Methods; Study Design & Population, paragraphs 2-3; eMethod 2 Variable Construction; eTable 3 Variables Comprising Feature Sets) |
| Statistical methods | 12 | (*a*) Describe all statistical methods, including those used to control for confounding (Methods, Text Processing Approaches; Encounter Classification; Statistical Analysis; eMethod Software Implementation) |
|  |  | (*b*) Methods used to examine subgroups and interactions described in: Method (Statistical Analyses, paragraph 1) |
|  |  | (*c*) Missing data described in: Method (Data Sources, paragraph 2); eMethod 2 Missingness and eMethod 2 Software Implementation |
|  |  | (*d*) If applicable, describe analytical methods taking account of sampling strategy |
|  |  | (*e*) Describe any sensitivity analyses: NA |
| Results | | |
| Participants | 13* | (a) Method (Study Design & Population, paragraph 1); eFigure 1 |
|  |  | (b) Method (Study Design & Population, paragraph 1); eFigure 1 |
|  |  | (c) Flow Diagram: eFigure 1 |
| Descriptive data | 14* | Results Table 1 |
|  |  | Method (Data Sources, paragraph 2) |
| Outcome data | 15* | Results (Sample Characteristics; Table 1; Figures 2-4) |
| Main results | 16 | (*a*) Give unadjusted estimates and, if applicable, confounder-adjusted estimates and their precision (eg, 95% confidence interval). Make clear which confounders were adjusted for and why they were included: Results |
|  |  | (*b*) Report category boundaries when continuous variables were categorized: not applicable |
|  |  | (*c*) If relevant, consider translating estimates of relative risk into absolute risk for a meaningful time period: not applicable |
| Other analyses | 17 | Results (Demographic Comparison; Diagnostic Comparisons) |
| Discussion | | |
| Key results | 18 | Discussion, paragraph 1 |
| Limitations | 19 | Discussion, paragraph 4. |
| Interpretation | 20 | Discussion: overall interpretation of results considering objectives (paragraph 1), limitations (paragraph 4), multiplicity of analyses (paragraphs 2-3), results from similar studies (paragraphs 2-3), and other relevant evidence (paragraphs 2-3). |
| Generalisability | 21 | Discussion, paragraph 4 |
| Other information | | |
| Funding | 22 | Funding Section |

# **TRIPOD+AI Checklist**

# *Collins GS, Moons KGM, Dhiman P, Riley RD, Beam AL, Van Calster B, Ghassemi M, et al. TRIPOD+ AI statement: updated guidance for reporting clinical prediction models that use regression or machine learning methods. BMJ. 2024;385.*

| Item | Checklist Description | Manuscript Location |
| --- | --- | --- |
| 1 | Title identifying model, population, outcome | Title |
| 2 | Abstract structured per TRIPOD+AI | Abstract |
| 3a | Healthcare context & rationale | Introduction, paragraphs 1-2 |
| 3b | Target population & intended users | Introduction, paragraph 3 |
| 3c | Health inequalities between groups | Introduction, paragraphs 2-3 |
| 4 | Study objectives clearly specified | Introduction, paragraph 3 |
| 5a | Data sources & representativeness | Methods (Study Design & Population, paragraph 1) |
| 5b | Data collection dates | Methods (Study Design & Population, paragraph 1) |
| 6a | Setting & location | Methods (Study Design & Population, paragraph 1) |
| 6b | Participant eligibility criteria | Methods (Study Design & Population, paragraph 1); eFigure 1 |
| 7 | Data preprocessing & quality | Methods (Data Sources & Variables), paragraphs 1-3; eMethod 2-4; eTable 3 |
| 8a | Outcome defined clearly & rationale | Methods (Data Sources & Variables, paragraph 3); eMethod 3 |
| 8b | Subjective outcome assessors qualifications | Methods (Text Processing Approaches, paragraph 1); eMethod 4 |
| 9a | Choice of predictors | Methods (Data Sources & Variables, paragraphs 1-2) |
| 9b | Predictors clearly defined | Methods (Data Sources & Variables); eMethod 2; eMethod 4; eTable 1; eTable 2 |
| 10 | Sample size rationale | Methods (Study Design & Population), paragraph 1; eFigure 1 |
| 11 | Missing data handling | Methods (Data Sources & Variables, paragraph 2) |
| 12a | Data partitioning | Methods (Statistical Analysis, paragraph 1) |
| 12c | Model type & validation | Methods (Statistical Analysis, paragraph 1) |
| 13 | Class imbalance handling | Methods (Statistical Analysis, paragraph 1) |
| 14 | Fairness approaches & rationale | Methods (Statistical Analysis, paragraph 1); Results (Demographic Comparison, paragraph 1) |
| 15 | Model output & thresholds | Results (Figure 1; eFigures 2-4) |
| 16 | Development vs evaluation data differences | Methods (Text Processing Approach, paragraph 1; Encounter Classification, paragraph 1) |
| 17 | Ethical approval | Methods (Study Design & Population, paragraph 3) |
| 18a | Funding & role | Funding section |
| 18b | Conflicts of interest | None reported |
| 18c | Protocol availability | Not applicable |
| 18d | Registration | Not applicable |
| 18e | Data sharing | Data availability statement |
| 18f | Code sharing | Methods (Statistical Analysis; paragraph 1) |
| 19 | Patient/public involvement | Methods (Text Processing Approaches, paragraph 1) |
| 20a | Participant flow | Methods (Study Design & Population, paragraph 1); eFigure 1 |

# **TRIPOD-LLM Checklist**

# *Gallifant J, Afshar M, Ameen S, Aphinyanaphongs Y, Chen S, Cacciamani G, Demner-Fushman D, Dligach D, Daneshjou R, Fernandes C, Hansen LH, et al. The TRIPOD-LLM reporting guideline for studies using large language models. Nat Med. 2025:1-10.*

| **Item** | **Checklist Description** | **Manuscript Location** |
| --- | --- | --- |
| 1 | Title identifying LLM, task, population, outcome | Title |
| 2 | Abstract structured per TRIPOD-LLM | Abstract |
| 3a | Healthcare context/use case & rationale | Introduction, paragraphs 1-2 |
| 3b | Target population & intended use | Introduction, paragraph 3 |
| 4 | Clearly specified objectives | Introduction, paragraph 3 |
| 5a | Data sources described separately | Methods (Study Design & Population, paragraph 1) |
| 5b | Quantitative & qualitative dataset description | Methods (Data Sources & Variables, paragraphs 1-3); eMethod 2-4 |
| 5c | Dates of oldest/newest text | Methods (Study Design & Population, paragraph 1) |
| 5d | Data preprocessing & quality checking | Methods (Data Sources & Variables), paragraphs 1-3; eMethod 2-4; eTable 3 |
| 5e | Handling missing/imbalanced data | Methods (Data Sources & Variables, paragraph 2); Methods (Statistical Analysis, paragraph 1) |
| 6a | LLM name, version, training date | Methods (Text Processing Approaches, paragraph 1); eMethod 4 |
| 6b | LLM architecture & fine-tuning details | Methods (Text Processing Approaches, paragraph 1); eMethod 4 |
| 6c | Text generation & prompt engineering details | Methods (Text Processing Approaches, paragraph 1); eMethod 4 |
| 6d | LLM output details | Methods (Text Processing Approaches, paragraph 1); eMethod 4 |
| 6e | Classification details & threshold identification | Methods (Statistical Analysis, paragraph 1) |
| 7a | Generative output quality metrics | Methods (Statistical Analysis, paragraph 1) |
| 7b | Metric relevance & human evaluation correlation | Methods (Data Sources & Variables, paragraph 3); Results (Figure 1; eFigures 2-4) |
| 7c | Outcome calculation details clearly defined | Methods (Statistical Analysis, paragraph 1) |
| 7d | Subjective outcome interpretation & assessors qualifications | Methods (Text Processing Approaches, paragraph 1) |
| 7e | Performance comparisons | Results (Figure 1; Tables 2-4; eTables 3-7 eFigures 2-4) |
| 8a | Annotation guidelines with examples | eMethod 3 |
| 8b | Annotators & agreement | Methods (Data Sources & Variables, paragraphs 3); eMethod 3 |
| 8c | Annotators' experience/background | Methods (Data Sources & Variables, paragraphs 3) |
| 9a | Prompt design, curation, selection | Methods (Text Processing Approaches, paragraph 1); eMethod 4 |
| 9b | Prompt development data | Methods (Text Processing Approaches, paragraph 1) |
| 10 | Preprocessing before summarization | Methods (Text Processing Approaches); eMethod 4 |
| 11 | Instruction tuning/alignment evaluation | Not applicable |
| 12 | Compute resources described | Methods (Statistical Analysis, Paragraph 1) |
| 13 | Ethical approval & consent | Methods (Study Design & Population, paragraph 3) |
| 14a | Funding sources & roles | Funding section |
| 14b | Conflicts of interest | None reported |
| 14c | Protocol availability | Not applicable |
| 14d | Study registration | Not applicable |
| 14e | Data availability | Data availability statement |
| 14f | Code availability | Methods (Statistical Analysis; paragraph 1) |
| 15 | Patient/public involvement | Methods (Text Processing Approaches, paragraph 1) |
| 16a | Data flow description | Methods (Data Sources) and Results, Tables 1-2 |
| 16b | Participant characteristics | Results, Table 1 |
| 16c | Clinical variables comparison | Methods (Data Sources & Variables, paragraphs 1-2) |
| 16d | Participant/event numbers per analysis | Figures 3-4; eTables 4, 6-7 |
| 17 | LLM performance metrics | Results, Figure 1; Figures 2-4; eTables 4-7. |
| 18 | LLM updating results | Not applicable |
| 19a | Main results interpretation including fairness | Discussion, paragraph 1 |
| 19b | Study limitations | Discussion, paragraph 4 |
| 19c | Challenges with data/task/domain | Discussion, paragraph 4 |
| 19d | Intended implementation details | Discussion, paragraph 3; eMethod 4 |
| 19e | Usability assessment of poor quality input | Not applicable |
| 19f | User interaction & required expertise | eMethod 4 |
| 19g | Next research steps & applicability | Discussion, paragraph 4 and Conclusion |
